# The Genome and Linkage Map of the Northern Pike (*Esox lucius*): Conserved Synteny Revealed between the Salmonid Sister Group and the Neoteleostei

**DOI:** 10.1371/journal.pone.0102089

**Published:** 2014-07-28

**Authors:** Eric B. Rondeau, David R. Minkley, Jong S. Leong, Amber M. Messmer, Johanna R. Jantzen, Kristian R. von Schalburg, Craig Lemon, Nathan H. Bird, Ben F. Koop

**Affiliations:** 1 Department of Biology, Centre for Biomedical Research, University of Victoria, Victoria, British Columbia, Canada; 2 The Charles O. Hayford Hackettstown State Fish Hatchery, Hackettstown, New Jersey, United States of America; Nanjing Forestry University, China

## Abstract

The northern pike is the most frequently studied member of the Esociformes, the closest order to the diverse and economically important Salmoniformes. The ancestor of all salmonids purportedly experienced a whole-genome duplication (WGD) event, making salmonid species ideal for studying the early impacts of genome duplication while complicating their use in wider analyses of teleost evolution. Studies suggest that the Esociformes diverged from the salmonid lineage prior to the WGD, supporting the use of northern pike as a pre-duplication outgroup. Here we present the first genome assembly, reference transcriptome and linkage map for northern pike, and evaluate the suitability of this species to provide a representative pre-duplication genome for future studies of salmonid and teleost evolution. The northern pike genome sequence is composed of 94,267 contigs (N50 = 16,909 bp) contained in 5,688 scaffolds (N50 = 700,535 bp); the total scaffolded genome size is 878 million bases. Multiple lines of evidence suggest that over 96% of the protein-coding genome is present in the genome assembly. The reference transcriptome was constructed from 13 tissues and contains 38,696 transcripts, which are accompanied by normalized expression data in all tissues. Gene-prediction analysis produced a total of 19,601 northern pike-specific gene models. The first-generation linkage map identifies 25 linkage groups, in agreement with northern pike's diploid karyotype of 2N = 50, and facilitates the placement of 46% of assembled bases onto linkage groups. Analyses reveal a high degree of conserved synteny between northern pike and other model teleost genomes. While conservation of gene order is limited to smaller syntenic blocks, the wider conservation of genome organization implies the northern pike exhibits a suitable approximation of a non-duplicated Protacanthopterygiian genome. This dataset will facilitate future studies of esocid biology and empower ongoing examinations of the Atlantic salmon and rainbow trout genomes by facilitating their comparison with other major teleost groups.

## Background

**Figure 1:**
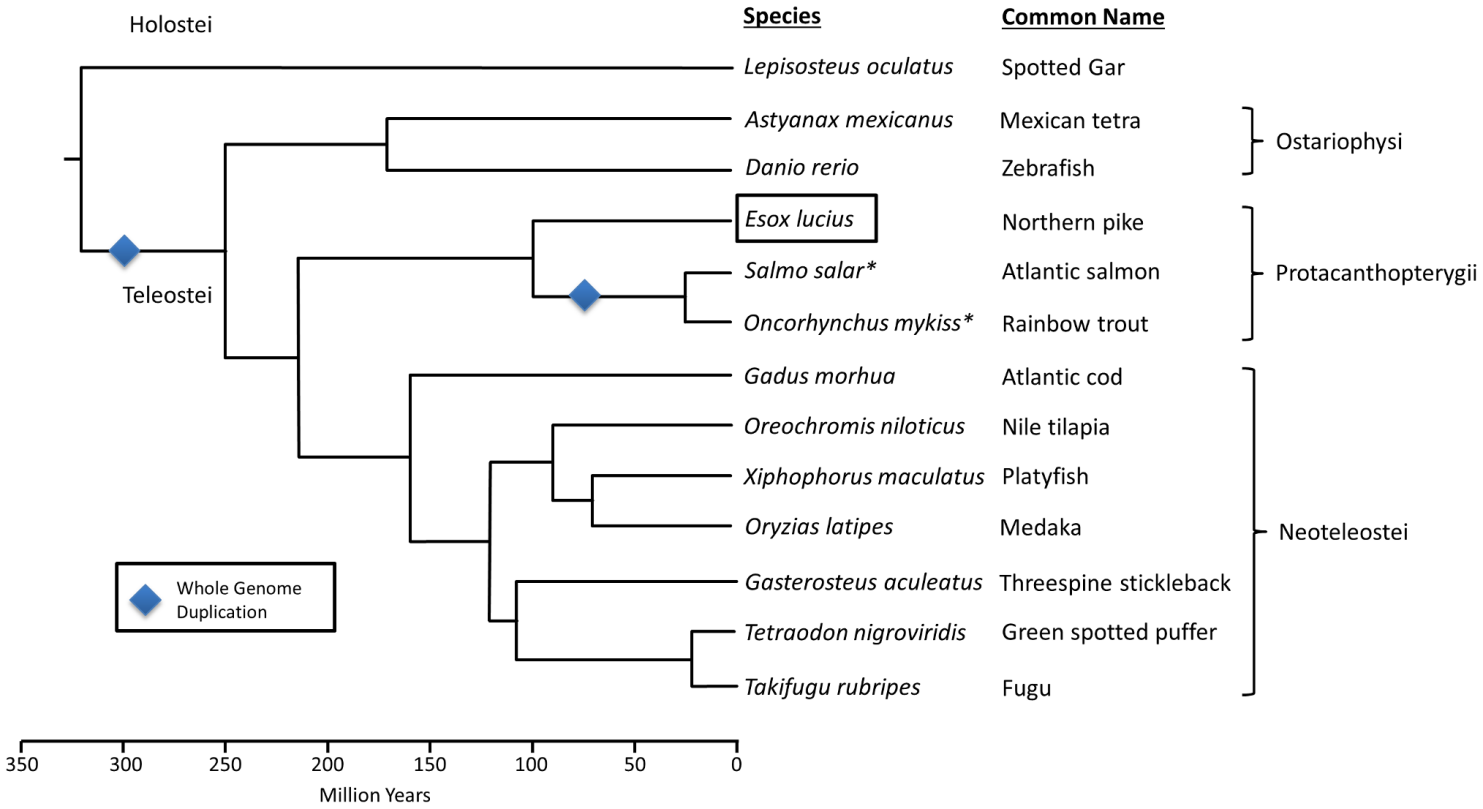
The phylogenetic relationship of teleosts with publicly available genome assemblies, as well as select species with ongoing genome projects (*). Divergence times and Subcohort naming based on Betancur et al. [1]. Diamond shapes represents presumed whole-genome duplications. *Lepisosteus oculatus* (Spotted gar) used as an outgroup.

Of the nine currently available teleost genome assemblies (www.ensembl.org), all fall into either the Neoteleostei or the Ostariophysi [1]. This is not necessarily surprising as combined these two clades comprise more than 70% of the approximately 26,840 extant teleost species [2]. While not as large, other groups contain a number of commercially important species and thus receive a significant amount of scientific attention. This observation is particularly true of the Protacanthopterygii (the salmonids, esocids and marine smelts) [2]. The Protacanthopterygii are placed in an interesting evolutionary position, having diverged from the Neoteleostei approximately 200 million years ago (MYA) [1] following an even more ancestral split from the lineage containing the Ostariophysi (250 MYA; see Figure 1). Thus, the generation of a genome sequence representing the Protacanthopterygii provides an intermediate phylogenetic branch of significant scientific interest.

While not conclusively resolved [1], [3]–[6] the Protacanthopterygii generally include the orders Salmoniformes, Esociformes (pikes and mudminnows) and Argentiniformes (marine smelts). The Salmoniformes (grayling, ciscoes, whitefish, trout, char, and salmon) are the most studied of the Protacanthopterygii and nearly all constituent species have significant economic, ecological, conservation, environmental and societal value [7], [8]. In the last 60 years, over 70,000 publications [7] have examined fundamental and applied questions relating to their fisheries, aquaculture, physiology, ecology, evolution, disease, resistance, reproduction, growth, tolerance to physical factors, and general immunity.

The Salmoniformes are a rapidly evolving order, with most species exhibiting in excess of 92% similarity in the coding DNA [9], [10]. The biological diversity and complexity observed within salmonids is undoubtedly a reflection of their complex genomes. While two rounds of whole-genome duplication (WGD) are thought to have preceded the origin of vertebrates and a third round (3R) is believed to have occurred in basal teleosts [11]–[15], the common ancestor of salmonids has purportedly experienced an additional, fourth round (4R) WGD approximately 60–100 MYA [7], [8], [16]–[18]. Modern salmonids are considered pseudotetraploid and various independent rediploidizations and genome restabilizations have occurred in different salmonid lineages as they revert to a stable diploid state [7], [8], [16], [17]. Although this recent duplication makes the salmonids difficult to use in the analysis of major teleost lineage evolution, it does make them ideal organisms for examining some of the early impacts of gene and genome duplications. These processes are thought to have played pivotal roles in promoting increased genetic diversity, the development of novel functions and functional specialization. The way in which a genome is reorganized to cope with duplicated chromosomes and the importance of gene duplications for evolution and adaptation represent long­standing biological questions that remain unresolved [7], [19]–[22]. Additionally, the role of repetitive DNA in facilitating post-duplication genomic reorganization is also of increasing interest in light of the recent expansions of transposable elements which have been identified within salmonids [23]. The salmonid-specific WGD, the resulting pseudotetraploid genomic state, and recent waves of transposable element activity all contribute to the increased complexity of relating genetic mechanisms to physical characteristics in salmonid species. The presence of duplicated genes has made it more difficult to clearly identify genetic mechanisms underlying physiological, immunological, ecological and evolutionary traits within the commercially and culturally valuable salmonids. Presently, the International Collaboration to Sequence the Atlantic Salmon Genome (ICSASG) represents researchers, funding agencies and industry from Canada, Chile and Norway. It has the goal of generating a robust salmonid reference genome sequence that facilitates the study of all salmonids [7]. This work in Atlantic salmon (*Salmo salar*) [7] is ongoing. During review for this work, the genome assembly for the rainbow trout (*Oncorhynchus mykiss*) was released [24] providing an additional dataset with which to analyse the results of genome duplication in the salmonids.

The genetic diversity in the Protacanthopterygii is reflected in their variable genome sizes and highly variable karyotypes that frequently differ from the presumed ancestral karyotype of 48 or 50 chromosomes [25]. This primitive karyotype is seen in a few members, including within two genera of the Esociformes. Total DNA content comparisons [26] and several gene studies [9], [10] suggest that Esociformes diverged from Salmoniformes prior to the salmonid WGD and that a genome of the Esociformes may closely approximate the ancestral genomic state. Therefore, it is important to determine how closely the organization of a representative Esociformes genome may reflect that of the ancestral Protacanthopterygiian. This information will help determine if comparisons to the Esociformes are appropriate in helping to resolve patterns of genome recombination and changes in gene expression that are implicated in the rediploidization of the salmonids.

Esociformes, an old lineage with fossils dating back to 70 MYA, contains a limited number of species (∼13) of which most literature concerns the northern pike (*Esox lucius*). The karyotype for the *Esox* genus is 2N = 50 (NF = 50) [27], [28], similar to the presumed karyotype of the teleost ancestor (2N = 48–50) [25]. This compares to the larger Salmoniformes karyotypes, which range from 2N = 52 (*Oncorhynchus gorbuscha*) to 2N = 102 (*Thymallus thymallus*) [26]. Esociformes outside the *Esox* genus have varying karyotypes of 2N = 22–78, with only *Novumbra hubbsi* exhibiting an ancestral-like diploid chromosome number of 2N = 48 (although the fundamental number indicates there are 62 chromosomal arms, suggesting significant rearrangement may have occurred [29]). A previous assessment of the suitability of northern pike as an outgroup for the study of duplicated genes in the salmonids examined 408 genes in Atlantic salmon (*Salmo salar*) and *E. Lucius* [10] and obtained results consistent with northern pike having a pre-WGD genome and being much more closely related to salmon than to other teleost species (zebrafish, medaka, fugu or stickleback). Such data suggest that the esocids provide an important intermediate comparison between salmonids and other more distantly-related fish genome sequences. For the present study *E. lucius* was chosen to represent Esociformes because of its availability, general interest and literature support.

The northern pike is a northern hemisphere, circumpolar, freshwater species that is typically the top predator in many lakes [30], [31]. It is an important northern recreational fishery and forms the basis of small commercial fisheries in some locations [32]. Northern pike are morphologically diverse and highly adaptable, they play a major role in structuring freshwater communities and are used in stocking programs to improve water quality [32]. Despite their wide distribution, northern pike populations have very low levels of polymorphism and divergence [31], indicative of a recent common ancestor. Genetic studies suggest that 1–3 post-glacial refugia in North America and several significant refugia in Europe [30], [33] contributed to the reduced levels of genetic diversity within this group.

In order to complement ongoing genome studies of Atlantic salmon and rainbow trout we sought to develop an extensive set of genomic resources for northern pike. To this end we have obtained a comprehensive set of northern pike transcripts and characterized their expression levels in various tissues. Further, we have produced a genome assembly that establishes the structure of genes, gene order, and repeat element content in northern pike. We have constructed a first-generation linkage map using highly variable microsatellite markers and mapped the relative position of both genomic scaffolds and expressed transcripts to linkage groups. Finally, we have examined the syntenic relationships between this representative esocid and distantly-related model organisms within the Neoteleostei and Ostariophysi in order to determine whether or not northern pike represent an appropriate model of a pre-duplication genome for use in the analysis of the salmonids. This work presents a significant resource for future studies of northern pike and provides the basis for invaluable comparisons in future salmonid studies, particularly once salmonid genomes become available.

## Results and Discussion

### A high quality genome assembly for *Esox lucius*

Libraries from 180 bp DNA fragments were Illumina paired-end sequenced (465.9 million 100 bp overlapping reads, ∼50X genome coverage) (Michael Smith Genome Sciences Centre, Vancouver, BC, CANADA). Libraries from 2 kb (448 million reads, ∼45X coverage) and 5 kb fragments (59.7 million reads, ∼6X coverage) were mate-pair sequenced (BGI). Assembly using ALLPATHS-LG [34] produced 94,275 contigs with 823,910,316 bp. Subsequent filtering (vector, duplicates, mitochondrial sequences) produced a finalized dataset comprising 823,673,596 bp in 94,267 contigs (mean length  = 8,738, median length  = 4,729, contig N50 = 16,909). Contigs were further joined by ALLPATHS-LG to include the estimated gaps between contigs based on mate-pair data, which produced 5,688 scaffolds with a total scaffolded genome size of 877,777,613 bp. The longest scaffold was 5.1 Mb, and the overall scaffold assembly had an N50 of 700,535 bp. In all, 199 scaffolds were over 1 Mb in length and 1,600 scaffolds were over 100,000 bp. These sequences have been uploaded to NCBI under bioproject PRJNA221548, accession GenBank:AZJR00000000, as well as to web.uvic.ca/grasp/pike in FASTA format. As the genome assembly improves, updates to the assembly will continue to be provided on the website. Major genome assembly statistics are summarized in Table 1.

**Table 1:** *Esox lucius* genome assembly statistics. Minimum contig and scaffold size ≥200 bp.

	Number (≥200 bp)	Bases (Mb)	N50 (Sequence length)	N50 (number of sequences)	Maximum Length (bp)
**Contigs**	94,267	824	16,909	13,483	232,364
**Scaffolds**	5,688	878	700,535	318	5,140,982

The most recent figures based on the c-value [35]–[37] estimate the *Esox lucius* haploid genome size to be approximately 1.09–1.12 Gb; if correct, our scaffold assembly covers approximately 78–79% of the genome. As there are known or suspected biases in next-generation sequencing (NGS) library construction (see [38] for example), it is possible that a small portion of the genome is missing from the sequencing libraries constructed. Published genome size estimates, however, range from 0.83–1.37 Gb [26], implying scaffold coverage could range anywhere from 64–100% of the genome. Therefore, because of the wide range of genome size estimates, a further estimate of genome completeness based on published EST transcript coverage [10] was performed. Of 11,662 EST contig sequences considered, 11,263 (96.6%) were successfully mapped to the scaffold assembly. Further, ALLPATHS-LG reported an estimated genome size of 911,413,085 bp based on the kmer distribution of raw genomic reads (k = 25), or 96.3% coverage. Together, these results suggest that the assembly represents a very large percentage of the genome.

While we cautiously expect that some chimeric scaffolds occur within our assembly, none were observed in our analysis of the largest 50 scaffolds (see synteny section below). Therefore, this initial version of the *E. lucius* genomic sequence provides an excellent framework to assess gene structure, gene content and general genome organization. The assembly statistics for both contigs and scaffolds are in-line with recently published fish genomes sequenced using NGS technologies, such as Atlantic cod (*Gadus morhua*; Contig N50 = 2,311 bp, Scaffold N50 = 393,166 bp) [39] and African coelacanth (*Latimeria chalumnae*; Contig N50 = 12,671 bp, Scaffold N50 = 924,513 bp) [40]. This assembly is the first genome assembly for a non-salmonid member of the Protacanthopterygii and represents an important evolutionary bridge between the two largest teleost subcohorts, Neoteleostei and Ostariophysi.

The genome sequence was examined for low-complexity repeat and transposable element content. This analysis allowed for the identification of major transposable element families present in the genome, and more importantly, provides a consensus library of transposable element sequences for use in filtering non-genic RNA sequences. An estimated 18.1% of the genome sequence is derived from transposable elements. This repetitive fraction is dominated by class II elements of the Tc1-Mariner superfamily (at least 9.42% of the genome is derived from Tc1-Mariner elements). The genomic abundance of observed transposable element taxa are reported in Table S1. An additional 2.0% of the genome consists of low-complexity sequences for a total of 20.1% of the genome annotated as repetitive sequence. Additional studies are currently underway to more thoroughly profile the transposable element sequences in *E. lucius* and to compare them to those of the Atlantic salmon. As a relative observation, 20% repeat-derived genome content is within the range represented in other teleost genomes; while repeat elements represent ≥50% of the well-characterized zebrafish genome [41], only 25% of the stickleback [42] and Atlantic cod genomes [39], 17.5% of the medaka genome [43], and <5% fugu genome [44] are TE-derived.

### Transcriptome assembly and Gene Identification

A total of 677,321,182 100 bp reads were obtained from 13 different tissues of a one year old individual and assembled into 413,679 contigs using the Trinity assembler [45]. Potential gene candidates were identified from the Trinity raw assembly. Those that were potential full-length annotated genes, had homology to a UniprotKB/Swiss-Prot or Gene Ontology protein, or those that possessed an unannotated ORF ≥300 bp were retained. All potential gene candidates were further filtered for the longest unique, non-overlapping location in the genome sequence. To reduce isoforms, alleles, recent duplicates, and possible sequencing errors, the potential gene candidates were screened for redundancy (≥98%, ≥300 bp). 38,696 potential gene candidates are represented in this curated non-redundant set, comprised of full-length annotations (9,553), UniprotKB/Swiss-Prot homology (14,538), Gene Ontology homology (604), and unannotated ORFs ≥300 bp (14,001). These gene candidates represent our *E. lucius* reference transcriptome set. These sequences have been submitted to NCBI under project accession GenBank: GATF00000000; the assembly described in this work is the first version, GenBank: GATF01000000.

An additional dataset incorporating all available transcriptome (including ESTs) and genome sequence data was prepared using the gene-modelling program MAKER2 to produce an *ab initio* dataset of 19,601 putative transcripts. It can be noted that the number of transcripts predicted by modelling is less than those from the de novo assembly. In some instances, this is a result of predicting a single gene model that spans multiple, non-overlapping, RNAseq contigs. In other cases, it appears to be due to limits in the data used to predict the models such as the size of the genomic contigs used; the short sequences limit the ability to successfully model a transcript. As the genome assembly is improved, so should the gene-modelling results. Datasets have thus been made available for download at web.uvic.ca/grasp/pike, and any future updates will be distributed here as well; the current MAKER2 dataset is additionally presented as File S1.

With previous EST studies and the current RNAseq data, we attempt to provide a comprehensive view of the number and identity of genes that make up the genome of *Esox lucius*. In combination with transcriptome data from other fish species, these gene lists will help facilitate future estimates of the identity and number of genes in the ancestral Protacanthopterygii lineage, before the whole-genome duplication occurred in the salmonid ancestor.

### A snapshot of tissue-specific gene expression

To determine the expression levels of hypothesized genes in each of the 13 tissues examined, reads from each tissue library were mapped back to the reference transcriptome and normalized expression values (fragments per kilobase of transcript per million mapped reads  =  FPKM) were determined for each sequence. All FPKM values from the non-redundant protein-coding transcriptome are presented in Table S2, while results based on the MAKER2 transcriptome can be found in Table S3. The primary intent of this initial characterization of gene expression is to provide a basic resource that can empower future work in *E. lucius* while also providing a baseline comparison for research in salmonids that seeks to understand the impact of gene duplication on transcriptional regulation and gene network organization.

To illustrate the general utility of gene expression values, the genetic complexity of the different tissues was investigated by analysing the magnitude and specialization of expression in each tissue. We discuss results based on the Trinity-assembled transcriptome, although findings using the MAKER2 transcriptome generally reflect the same trends. Unsurprisingly, the most common highly-expressed genes are NADH-ubiquinone oxidoreductase, actin, cytochrome c and the ribosomal protein-coding genes (Table S2); these occur within the top 10 highest-expressed genes in almost all tissues. We also looked at specialized expression patterns for each gene by identifying any tissues in which FPKM values were more than three standard deviations greater than the average across all tissues. The number of specialized transcripts was counted for each tissue (Table S4) and the order of tissues by decreasing complexity is: brain (5,408 transcripts), testis (2,784 transcripts), spleen (1,100 transcripts), eye (1,055 transcripts), nose (804 transcripts), gut (659 transcripts), heart (630 transcripts), muscle (430 transcripts), kidney (406 transcripts), gill (374 transcripts), head kidney (338 transcripts), liver (332 transcripts) and stomach (140 transcripts). The brain represents the most complex/specialized tissue with the largest number of genes expressed and the largest number of specialized genes. We found that the testis were the second-most complex and specialized tissue, a result that is in accord with the work of others [46]. The spleen, eye and nose regions have the next highest number of specialized genes expressed.

In examining the ten most specialized genes in each tissue (Table S4) it was found that chymosin, gastricsin, and pepsin were highly expressed in the stomach tissue, rhodopsin and gamma-crystallin in the eye, immunoglobulins in the head kidney and complement in the spleen, genes often associated with these tissues. The muscle was the least complex tissue in terms of the number of transcripts expressed, however the stomach tissue expressed the smallest number of specialized genes. Perhaps one surprise was the high level of ice-structuring protein expressed in the gut. Though antifreeze protein (AFP) gene expression is known to occur in many tissues, it is typically produced in the liver and transferred to the blood [47]. However, as the gut is a possible route whereby nucleating ice might enter the fish, the finding of high transcription of AFP in the gut is informative and may help to explain some of the protective physiology of survival in high northern climates.

Documenting the expression values for each of the transcripts from the different tissues provides a single snapshot of expression in one set of conditions and is therefore bound to vary in different circumstances. This snapshot does, however, provide a basal atlas of gene expression estimates that can be used by the fish community for many experimental and comparative purposes.

### *E. lucius* homologues suggest a pre-duplication genome

**Figure 2:**
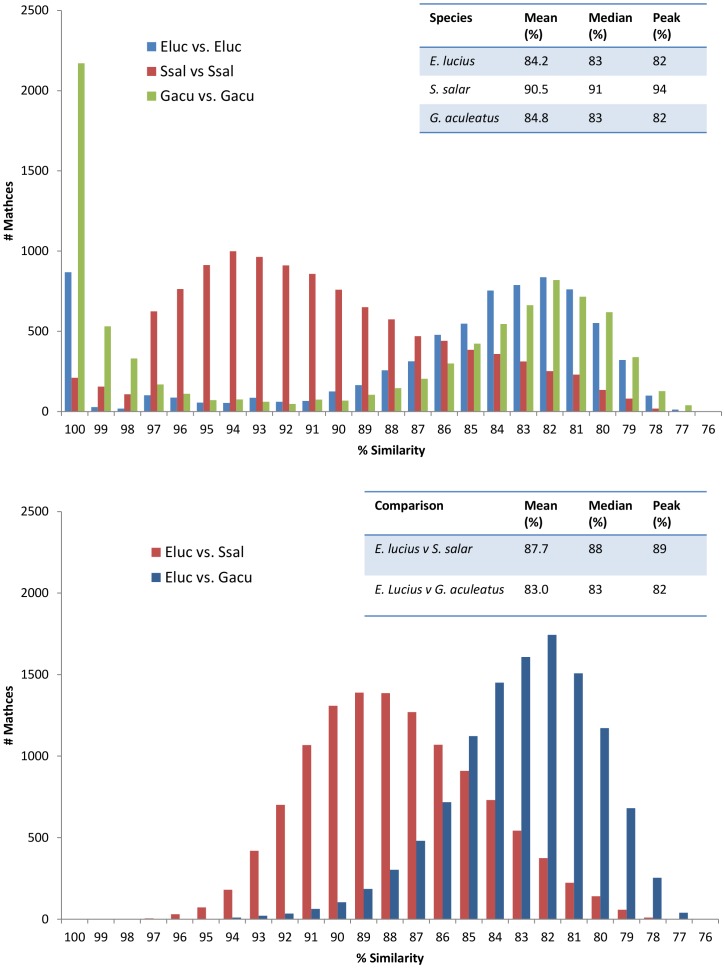
The percent similarities identified through transcriptome blastn comparisons. Blast results from non-redundant, repeat mask transcripts >300 bp with minimum 100 bp alignment. Results plotted by 1% intervals, grouped into bins. Results are graphed as A) within species (paralogues) and B) between species (Orthologues). Eluc  =  *Esox lucius*, Ssal  =  *Salmo salar*, Gacu  =  *Gasterosteus aculeatus*.

To investigate patterns of gene duplication in *E. lucius*, we plotted the percent similarity of transcript homologues between and within northern pike, Atlantic salmon and threespine stickleback (*Gasterosteus aculeatus*) (Figure 2). To facilitate this analysis three transcript datasets were used: 1) 38,696 reference transcripts identified within our RNA-seq assembly; 2) previously published Atlantic salmon EST data [9], [10], and; 3) *G. aculeatus* transcripts available from the UCSC genome browser. Transcript homologues were identified within and between datasets using reciprocal best BLASTN hits following a similar approach previously used to analyse Atlantic salmon homologues [9]. In this analysis percent similarity was used as a proxy for evolutionary time; lower percent similarity between two paralogues suggests more time has passed since their origin by duplication.

Two peaks of percent similarity are apparent in the northern pike paralogue comparisons (Figure 2A): one above 97% and one centred at 82%. The analogous plot of stickleback paralogue similarity exhibits a similar profile (with a nearly identical peak at 82% identity). If a similar rate of mutation is assumed within the northern pike and stickleback lineages, the overlapping peaks occurring between 77% and 89% suggest a common origin. These peaks may represent the remnants of the 3R basal teleost genome duplication, which would have occurred in a distant common ancestor of both lineages. Atlantic salmon paralogue comparisons, in contrast, show much higher percent similarities and exhibit a notable peak at 94% identity, a region in which very few northern pike or stickleback paralogues appear. This upward shift in the peak of percent similarity is most easily explained by the fourth-round (4R), salmonid-specific WGD. While Atlantic salmon does not exhibit a distinct peak in the region identified in the other two fish species (77–89% similarity), the right tail of its percent distribution is considerably drawn out. The notably extended tail may be a result of a) paralogues produced following the 4R WGD with a much increased rate of sequence divergence relative to the average, or b) remnants of the 3R duplication in which one copy of each paralogous gene pair produced following a salmon-specific 4R duplication has been lost. Together, these observations support the hypothesis that an Atlantic salmon ancestor experienced a comparatively recent whole-genome duplication event that was not experienced in the pike or stickleback lineages, and supports the use of the northern pike as a WGD outgroup.

The peaks occurring above 97% similarity in all within-species comparisons imply very recent gene duplication events that are unique to each species. Although gene duplication is known to occur at very high rates, the vast majority of those duplicate pairs that are fixed in a lineage are thought to experience rapid loss of one or both copies, occurring within a few million years [48], [49]. The high-similarity peaks observed in our within-species comparisons likely result from this dynamic of creation and loss; many paralogues are almost identical, indicating recent duplication, however fewer paralogues are observed as similarity decreases. These young paralogues may have been created by a recent series of segmental duplications [50], or may simply be an artefact of sequencing errors; we cannot with the current data distinguish between the two possibilities.

The most divergent peak observed in the northern pike within-species comparisons occurs from 77% to 89% similarity and contains 5,885 total transcripts. The annotations of these transcripts were examined in order to identify significantly over-represented functional categories (Table S5). Many of the terms identified fall into the development, signalling and regulatory classifications Brunet et al. [51] found to be enriched in *Tetrodon nigroviridis* gene duplicates likely to be from the 3R duplication. Of the Gene Ontology (GO) terms identified in the presumed 3R orthologues in northern pike however, the most striking are phosphate metabolism biological processes and nucleoside-binding and nucleotide-binding molecular functions. Interestingly, nucleotide-binding molecular function category was significantly under-represented in 3R-duplicated genes examined in the *Tetraodon* pufferfish. Examining more recent duplicates in carp, Wang et al. [52] identified immune-related terms and pathways as the predominantly enriched biological functions relative to in the recently duplicated common carp (*Cyprinus carpio*) genome, relative to the Zebrafish; immune-related terms were not a significant component of the GO terms identified in this study. The diversity of enriched GO categories implied by these studies suggests that different taxonomic groups may retain gene duplicate pairs from different functional categories, thus contributing to overall taxonomic diversity. Alternatively, the observed differences could indicate that different functional categories of gene duplicates are retained over different lengths of time.

Utilizing the same approach as that used for within-species comparisons, northern pike transcripts were compared with their orthologues in both the Atlantic salmon EST dataset and the threespine stickleback transcriptome (Figure 2B). Unsurprisingly, Atlantic salmon and northern pike orthologues are on average more similar (distribution maxima at 89%) than northern pike transcripts are to their respective orthologues in threespine stickleback (distribution maxima at 82%). These data are consistent with the expectation that the pike are more closely related to the salmonids than to species in the Neoteleostei, including stickleback. As northern pike and Atlantic salmon are estimated to have diverged from one another 100–130 million years ago (MYA) [1], [4], the value of 89% provides a very broad reference point with which to estimate the timing of the orthologue divergence in the transcriptomes of the Salmoniformes and Esociformes.

### RAD-tag based linkage mapping inefficient in pike

Our attempts to produce a RAD-tag based genetic map from a single full-sib family of *E. lucius* were unsuccessful. From 33,588 *Sbf*I-associated sequence loci across all family members, 351 informative polymorphic SNP markers were identified, or about 175 informative SNPs per sex. Further, the combination of very few useful markers and the bi-allelic nature of the SNPs did not allow for an efficient merge of the two sex-specific maps. Attempts to produce a useful linkage map from this data proved fruitless. Our initial decision to pursue this approach was based on its comparative success in other fish species. Other studies using the same protocol and enzyme produced 8,406 mapped SNPs in the spotted gar [53], 6,458 mapped SNPs in Atlantic salmon [54] and 5,703 mapped SNPs in Atlantic halibut [55]. The poor performance of this method in our study is almost certainly due to the very low degree of polymorphism observed in the northern pike data; the ALLPATHS-LG genome assembler reported an estimated polymorphism rate of 1 SNP for every 10,830 bp and an independent analysis of our RAD-tag dataset indicated an average polymorphism level of approximately 1 SNP for every 6,000 bp. The polymorphism rate estimates from our data are consistent with the very low levels of genetic polymorphism and heterozygosity noted in previous genetic studies of North American pike populations [33]. It is possible that future attempts utilizing a restriction enzyme which cuts more frequently could restore the usefulness of this RAD-tag approach. Such changes, however, would significantly increase the number of sequencing lanes required, thereby reducing some of the cost-advantages of the RAD-tag technique.

### A first-generation, Microsatellite based linkage map

In an attempt to increase the detection and integration levels of polymorphism, we utilized a half-sibling family (48 progeny from one father and 44 from a second) in combination with microsatellite markers. Utilizing 32,833 previously published northern pike EST sequences [9], [10] we identified long near-perfect di- and tri-nucleotide repeats from which we generated 776 microsatellite primer pairs, of which 187 (24%) were found to be polymorphic in at least one of the parents. Of these polymorphic primer pairs, 180 were successfully mapped back to a single *E. lucius* genomic scaffold. To further extend our database of polymorphic primer pairs we similarly screened the largest 900 genomic scaffolds for long near-perfect di- and tri-nucleotide microsatellites. This approach allowed us to screen an additional 835 scaffold-designed primer pairs against the parents of our half-sib family. We identified 378 primer pairs that were polymorphic in at least one of the parents with between 1 and 6 markers identified per scaffold. From a total of 1,611 screened primer pairs we identified 565 polymorphic markers that cover 429 genomic scaffolds; 7 such markers could not be mapped to a scaffold.

The 565 polymorphic primer pairs we have identified represent a very significant increase in the available marker data for *E. lucius*; prior to this work only 70 markers were available [56]–[60]. Given the low level of polymorphism observed in northern pike, particularly in North American populations, the additional marker availability should be useful to future studies that seek to delineate population structure or trace unauthorized introductions of this fish species.

**Figure 3:**
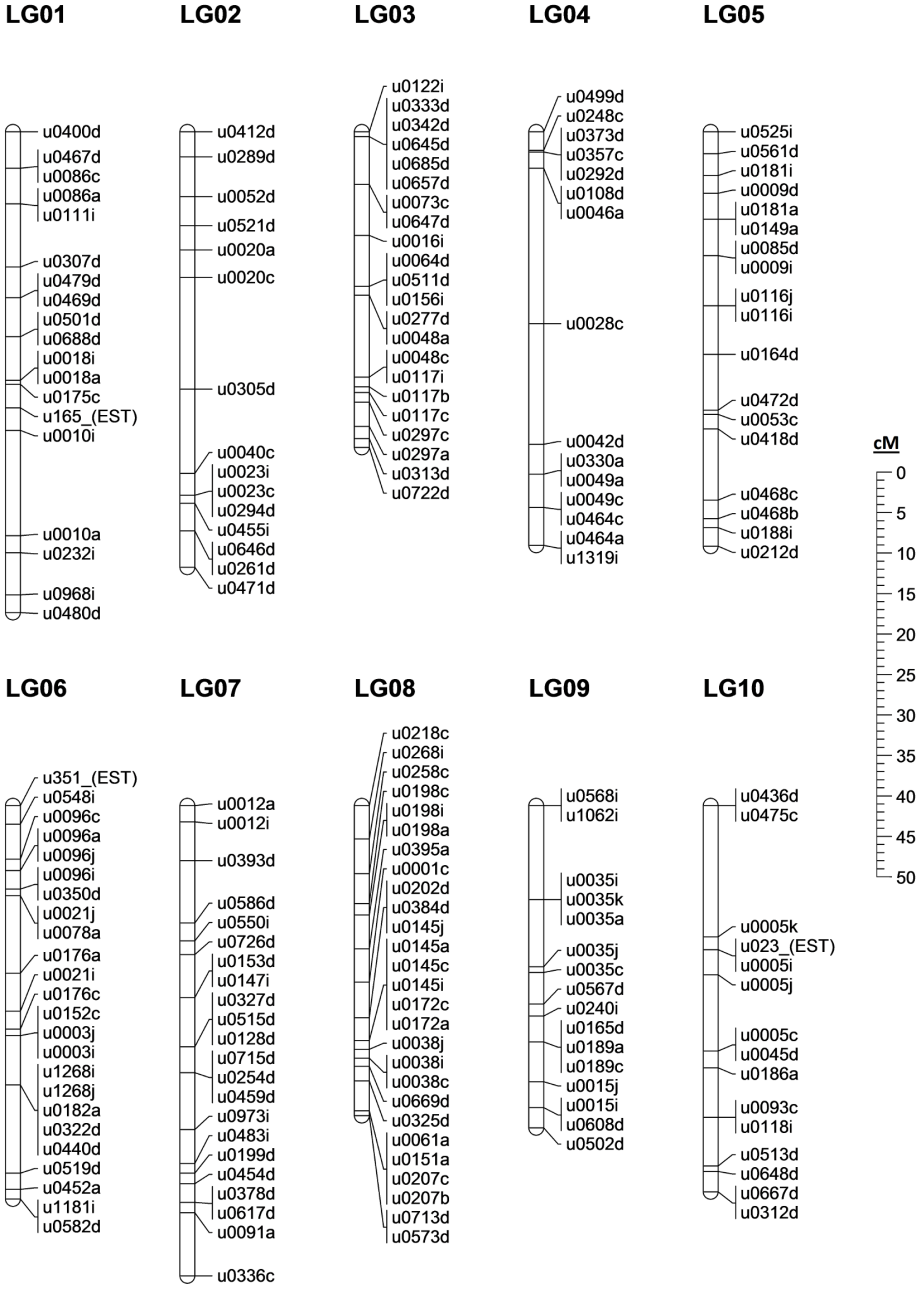
The northern pike merged linkage map: LG-01 to LG-10. Genomic scaffold identified by first 4 digit number, with multiple scaffold markers distinguished by final letter. Markers not mapped to a scaffold identified by “(EST)”; sex-specific linkage maps in Figure S1. Marker information in Table S7.

**Figure 4:**
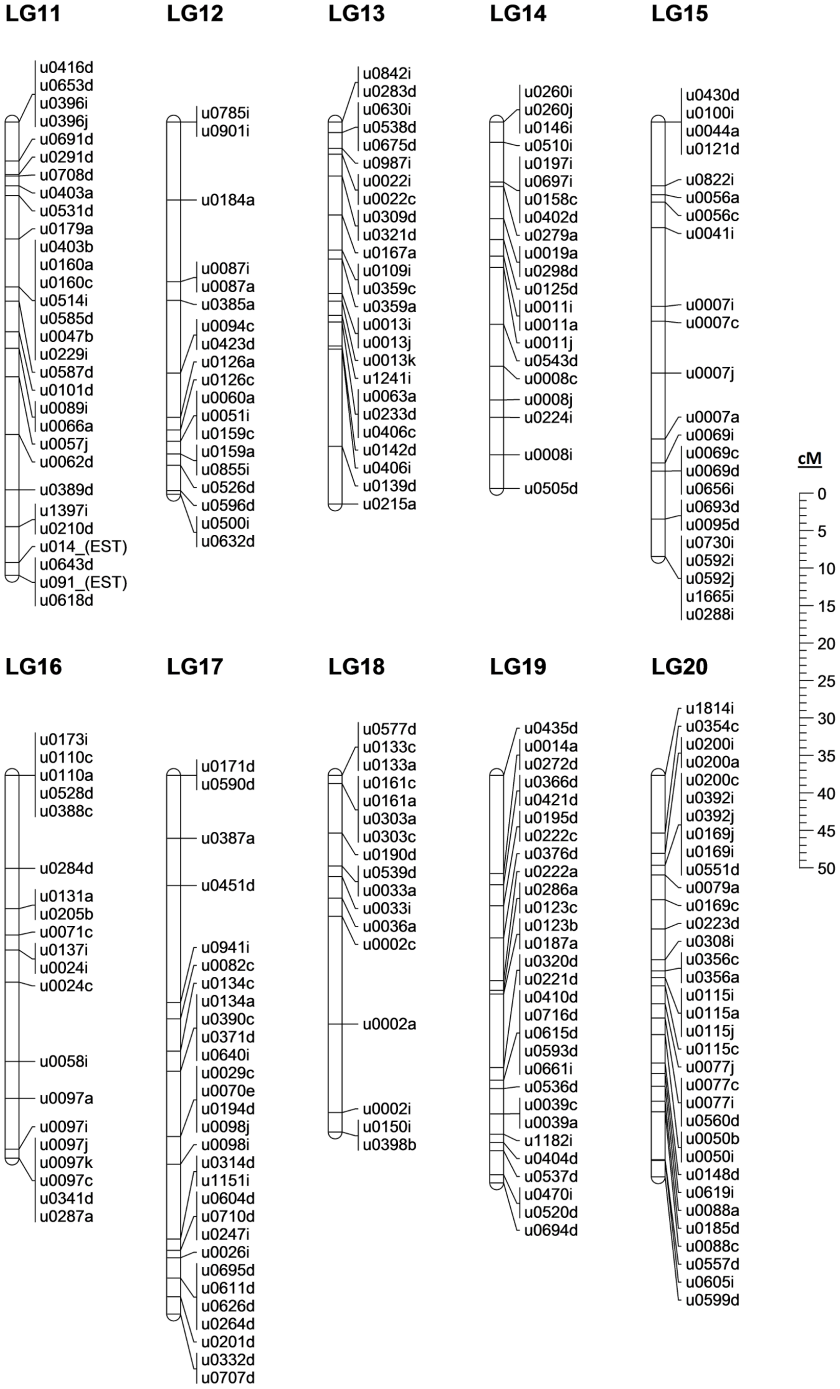
The northern pike merged linkage map: LG-11 to LG-20. Genomic scaffold identified by first 4 digit number, with multiple scaffold markers distinguished by final letter. Markers not mapped to a scaffold identified by “(EST)”; sex-specific linkage maps in Figure S1. Marker information in Table S7.

**Figure 5:**
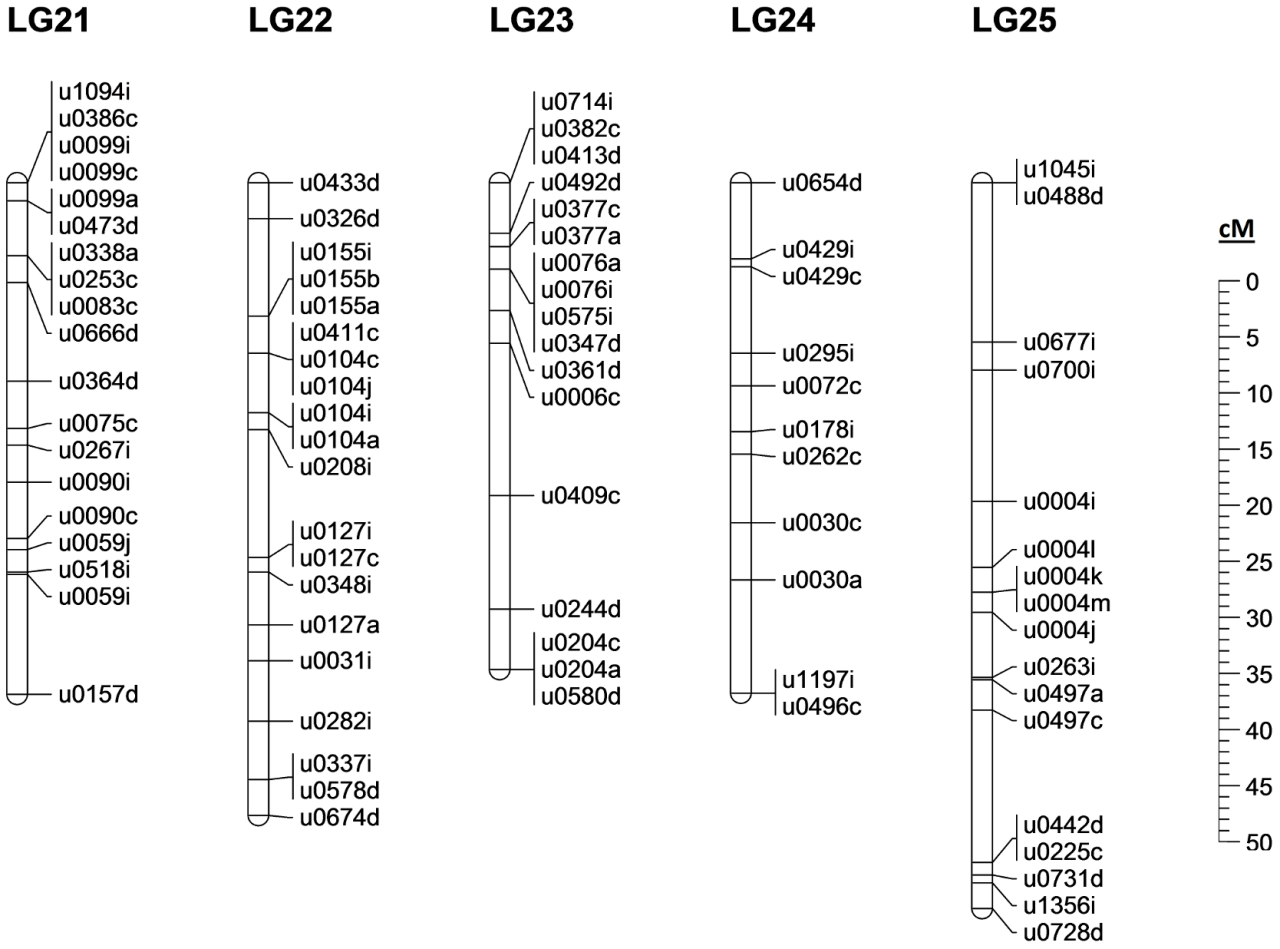
The northern pike merged linkage map: LG-21 to LG-25. Genomic scaffold identified by first 4 digit number, with multiple scaffold markers distinguished by final letter. Markers not mapped to a scaffold identified by “(EST)”; sex-specific linkage maps in Figure S1. Marker information in Table S7.

Analysis of the genotypes found within our half-sib family allowed 526 of the 565 polymorphic primer pairs (and 406 scaffolds) to be mapped across 25 linkage groups spanning 1289.3 cM, the expected number of linkage groups based on previous *E. lucius* karyotyping [27]. The final merged linkage map is presented in Figures 3–5. An additional 12 markers were polymorphic but remained unlinked at the LOD cutoff of 3.0. Of the 28 markers with more than 15% missing data, all but one were dropped from the linkage analysis: u0028c was maintained in the data set to join two groups of markers into the single linkage group LG-04. This join was further verified at LOD 3.0 using u0046a, u0028c and u0330a in an unrelated family of northern pike (Whiteshell hatchery, MB, Canada). While most of the mapping procedure was straightforward, some difficulty was observed in the merging of the map due to a low level of polymorphism across multiple individuals. Of the 526 mapped markers, 301 were only informative in a single parent, leaving relatively few common markers with which to merge the maps. Therefore, true marker order can only be determined from the individual maps in Figure S1; the merged map is presented as a best interpretation of the available data.

Sex-specific maps spanned 1245.4 cM in the female and 1166.0 cM in the male, an average recombination ratio of 1.07∶1 between females and males. Regional recombination differed across and within particular linkage groups between the sexes, with one region in particular standing out. In the male maps, the top of LG14 shows strongly repressed recombination in the males relative to the females, with ∼11X greater rate of recombination between scaffold 260 and scaffold 11 in the female map relative to the male. While the average recombination rates diverge slightly from even between the two sexes in pike, the recombination ratio is not nearly as skewed as in other teleost species; in Atlantic salmon, recently published ratios ranging from 1.3–1.7∶1 (female:male) [54], [61], in rainbow trout (*Oncorhynchus mykiss*) 1.68∶1 [62], and in zebrafish (*Danio rerio*) 2.74∶1 [63].

In one of the fathers we noted the unusual observation that of the 29 markers mapped to LG-19, none were found to be polymorphic (individual male-1 in Figure S1). In early karyotyping of northern pike various chromosome counts were observed [27], particularly in the range of 48–50 chromosomes; in concert with our data these observations suggest that the father was possibly missing a single chromosome, or distributed only a single chromosome to its progeny. It is also possible, depending on the degree of inbreeding within our sampled population, that this particular father inherited a separate but nearly identical chromosome from each of its parents; no observable polymorphism would be displayed in this situation. We are unable to determine the biological source of this unusual observation, as the available tissues are insufficient for karyotyping.

The low level of polymorphism implied by our data produces an interesting trade-off: genome assembly proved relatively straightforward due to a lack of sequence ambiguity introduced by polymorphic sites, while this same lack of polymorphic diversity ultimately limited the number of scaffolds that could be placed and oriented on the linkage map. Originally, we identified and targeted at least two microsatellite markers on each of the longest genome scaffolds; however, very few scaffolds ultimately possessed more than one such marker that was usefully polymorphic. Only 41 marker pairs derived from the same scaffold were mapped in such a way as to imply definitive scaffold orientation in at least one of the sex-specific linkage maps. Generally, such marker pairs were from the largest scaffolds and almost all were found on scaffolds greater than 1 Mb in length. The 406 scaffolds mapped by at least one microsatellite marker to this first-generation linkage map contribute to a total of 46% of scaffold assembly bases being associated with a linkage group. Additional northern pike families, further polymorphic marker identification, directed sequencing of scaffold gaps, and/or additional whole-genome sequencing will be required in order to improve this value in future work.

### Synteny analyses suggest Neoteleostei-like genome in northern pike

Given that the majority of teleost species fall into either the Ostariophysi or the Neoteleostei subcohorts, it is understandable that all of the nine teleost genome sequences available in the Ensembl genome browser (December 2013) fall into one of these two clades. While the Ostariophysi are thought to have diverged from the Euteleosteomorpha approximately 250 MYA, the next branch point possessing a representative genome assembly occurs within the Neoteleostei, approximately 160 MYA when the Paracanthomorphacea (represented by the Atlantic cod) diverged from the Euacanthomorphacea [1]. Usefully, the Protacanthopterygii are thought to have branched off from other Euteleosteomorphs approximately 200 MYA, and as such they represent a phylogenetically intermediate branch point between the fish species with currently available genomic data. When considering the reconstruction of ancestral genomes for evolutionary studies, the addition of branch points and the decreasing of branch lengths can only improve the quality of the results. If a phylogenetically useful Protacanthoptyerygiian genome is desired, the selection of an order that did not experience the salmonid-specific genome duplication is desirable in order to limit complications; such an order could be reasonably expected to provide the most straightforward and ancestral-like genome that can be obtained from this group. The Esociformes, represented in the present study by northern pike, are such a suitably placed order; the northern pike genome will be an excellent intermediate resource for use in phylogenetic studies of teleosts.

**Figure 6:**
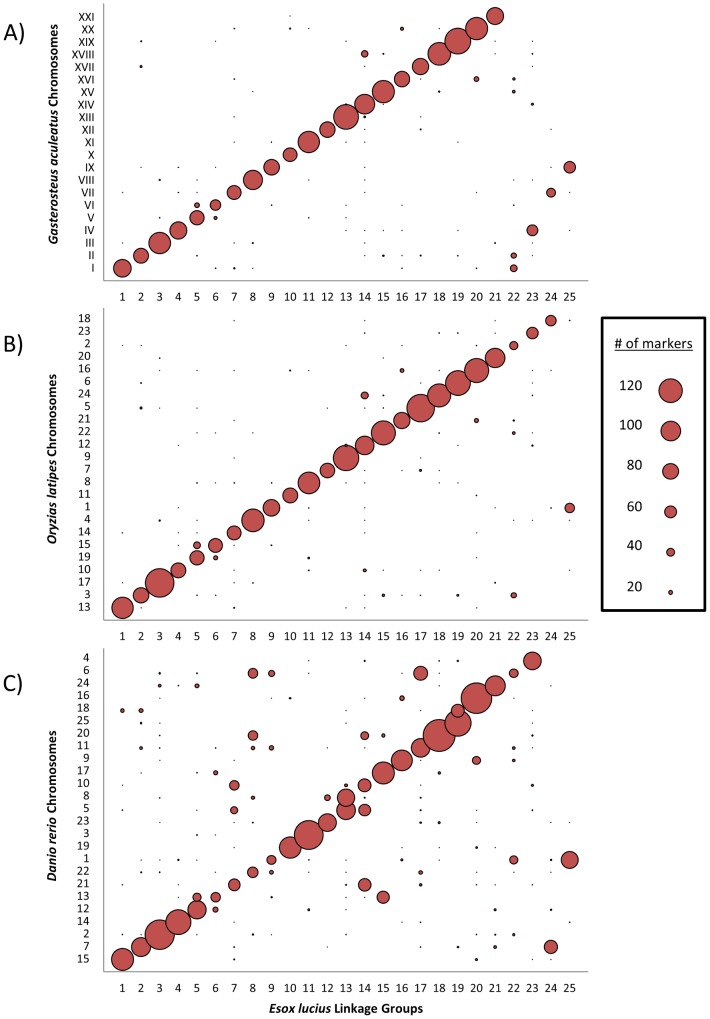
The synteny between the northern pike and model teleosts. Reciprocal best hit results (≥50% of total length, ≤1e-5) displaying synteny between the 25 pike linkage groups and three model teleost genomes, threespine stickleback (*Gasterosteus aculeatus*), medaka (*Oryzias latipes*), and zebrafish (*Danio rerio*). Sphere diameter reflects the number of matched transcripts. For numerical results, see Table S6.

Using the genetic map we set out to determine whether or not the gene distribution and genome structure of northern pike imply synteny with the hypothesized genome of the ancestral teleost, as is tentatively implied by karyotype morphology. To this end, the syntenic relationships were examined between four species representing three major subcohorts of the infraclass Teleostei: northern pike (*Esox lucius*) was chosen to represent the Protacanthopterygii, zebrafish (*Danio rerio*) was chosen to represent the Ostariophysi, and both threespine stickleback (*Gasterosteus aculeatus*) and medaka (*Oryzias latipes*) were selected to represent the Neoteleostei. First, transcripts from northern pike were mapped to those northern pike genomic scaffolds that had previously been assigned to a group on the linkage map. Putative orthologues for these mapped transcripts were then identified in the transcriptomes of the three other representative teleosts using a reciprocal BLAST approach. A linkage group was subsequently inferred for each mapped transcript based on that of its parent scaffold. Finally, this *E. lucius* transcript linkage group was compared to the chromosomal location of the transcript's orthologue in stickleback (Figure 6A), medaka (Figure 6B) and zebrafish (Figure 6C); see Table S6 for specific numbers.

Considering the time since their last common ancestor there exists a clear association between linkage groups in *E. lucius* and chromosomes in *G. aculeatus* and *O. latipes*. In general, each northern pike linkage group predominantly associated with a single stickleback chromosome. In four cases there were two *E. lucius* linkage groups that associated with only a single stickleback chromosome. Of these four cases, only one was analogously observed in *Oryzias latipes* (Figure 6B), a species that possesses a karyotype equivalent to the presumed ancestral karyotype in the Neoteleostei [25]. Specifically, only chr. 1 of *O. latipes* (orthologous to *G. aculeatus* chr. IX) shows a strong association with two separate *E. lucius* linkage groups. This observation suggests that the other three instances in which a single stickleback chromosome corresponds to two northern pike linkage groups (ie. *G. aculeatus* chrs. I, IV and VII) are best described as stickleback lineage-specific fusions of three pairs of ancestral-like chromosomes. This interpretation is further supported by recent work using sablefish (*Anoplopoma fimbria*), a species which is much more closely related to stickleback than is northern pike or medaka [64].

Two *E. lucius* linkage groups consistently associate with only a single chromosome of the other representative teleosts. As previously discussed, transcripts from northern pike linkage groups LG-09 and LG-25 predominantly possess orthologues on stickleback chr. IX and medaka chr. 1; an analogous situation is observed with zebrafish chr. 1. These observations suggest that the common ancestor of the Neoteleostei and the Protacanthopterygii most likely possessed a haploid karyotype of 24 chromosomes; this is in contrast to the haploid karyotype of 25 chromosomes observed in members of the *Esox* genus [27] and in the majority of the members of the Ostariophysi [25]. This analysis further implies that the two *E. lucius* chromosomes represented by LG-09 and LG-25 were formed as a result of the fission of a single ancestral chromosome, and that this fission occurred after the divergence of the Protacanthopterygii lineage. Syntenic analyses of orthologous sequence shared by stickleback and Atlantic salmon [61] suggest that the ancestral chromosome that eventually evolved into stickleback chromosome IX had already split in two prior to the salmonid-specific genome duplication. Therefore, this chromosomal fission likely occurred prior to the Esociformes/Salmoniformes divergence and it can be reasonably concluded that the common ancestor of the Esociformes and Salmoniformes possessed a 2N = 50 karyotype which was secondarily derived from the more ancestral karyotype of 2N = 48.

While the distribution of orthologous genes to chromosomes was particularly well conserved between the Protacanthopterygii and the Neoteleostei, the gene order along chromosomes was not as consistently similar. The positions of mapped genes in northern pike and the three model teleost genomes were plotted for the largest 50 scaffolds (Figure S2). In some instances, such as scaffolds 4 and 6, syntenic blocks in excess of three 3 million bp can be found where gene order appears to be conserved between medaka, stickleback and northern pike. In other cases, such as those observed in scaffolds 13 and 15, there are only small blocks of 500,000 bp or less where conservation of gene order can be observed. There are qualitatively many fewer occurrences of conserved gene order between zebrafish and pike, however there do exist large tracts of conserved order in certain scaffolds; one such scaffold is scaffold 1, where sections of 1–2 million bp maintain consistent ordering. In fact, gene order appears to be more consistently similar between zebrafish and pike than between pike and medaka in scaffold 1.

While the relatively small number of reciprocal blast hits per scaffold makes quantifying the degree of gene order conservation difficult, the number of consistently ordered transcripts from a single syntenic chromosome lends confidence to the quality of the assembled scaffolds. In three of the largest 50 scaffolds (scaffolds 8, 9 and 31), however, northern pike transcripts are consistently mapped across two or three chromosomes in all three model teleosts; this raises the possibility of chimeric scaffold assembly. An examination of the scaffolds mapped to either side of scaffold 9 in LG-05 implies that the discrepancy in this particular scaffold represents a genuine chromosomal translocation, in which the break point falls in the middle of the scaffold. Scaffolds to one side of LG-05 exhibit blast hits predominately to *G. aculeatus* chr. V, while scaffolds on the other show blast hits to *G. aculeatus* chr. VI. Similarly, scaffold 8 (mapped to LG-14) also appears to contain a genuine chromosomal translocation rather than a chimeric assembly artefact. This is inferred because a) the majority of other scaffolds on LG-14 possess best blast hits to *G. aculeatus* chr. XIV and b) scaffold 224, mapped adjacent to the chr. XVIII ‘end’ of scaffold 8 in LG-14, exhibits blast results dominated by hits to *G. aculeatus* chr. XVIII. Given the pronounced split between these regions it seems likely these translocations are quite recent and that the genes involved have not yet had time to be re-distributed to other regions across the chromosome. The third scaffold exhibiting potential assembly chimerism, scaffold 31 (located on LG-22), is not flanked by scaffolds that support a similarly distinct breakpoint. However, scaffolds mapped to LG-22 possess genes with significant numbers of orthologues on at least four different chromosomes in *O. latipes* and *G. aculeatus* (Figure 6A and B; Table S6); genes from different chromosomal sources are also mixed and distributed over the entire length of this linkage group. Together, these observations suggest that LG-22 possesses an old collection of pieces from multiple ancestral chromosomes that have had significant time to redistribute themselves throughout the linkage group. This characterization of LG-22 supports the idea that scaffold 8 reflects the biological reality and is not the result of inappropriately linked assembly contigs. While we are unable to rule out the possibility of chimeric joins having occurred in our genomic assembly, the three large scaffolds deemed most likely to have been chimeric show evidence suggesting that they are indeed real.

Although gene order is highly variable, chromosomal synteny remains high between the northern pike and the Neoteleostei representatives. While small sections appear to have undergone chromosomal translocations, and LG-22 may be highly reorganized, there is generally a 1∶1 relationship between linkage groups identified in northern pike and the chromosomes in the Neoteleostei. This implies the genome of *E. lucius* is indeed ancestral-like and will provide a good overall representative of the pre-duplication Protacanthopterygii genome structure in wider studies of genome evolution.

### The usefulness in salmonid duplication analysis

While inferring ancestral states is made easier with an ancestral-like genome, the examination of more recent events, such as the WGD event and subsequent rediploidization in the salmonid lineage, can be made much easier using this genetic dataset. Although numerous rearrangements have undoubtedly had a substantial effect on genome organization, many of the greatest genomic changes must be seen as the consequence of the formation and evolution of paralogous genes resulting from a WGD (such gene pairs are termed ohnologues [65]). As the genome attempts to revert to a stable diploid state, ohnologues are presumed to evolve like individual gene duplicates through subfunctionalization, neofunctionalization or loss of one of the gene copies. Determining the mode and rate of gene and genome evolution requires an understanding of where the two copies began. Gaining this understanding would be most easily accomplished using the ancestral, pre-duplicated sequence of the gene or genome segment; since this sequence is unavailable it is most readily inferred using an appropriate pre-duplication sister group. Even given the significant amount of time which has passed since their divergence from each other over 100 MYA [1] the Esociformes remain the most closely related order to the Salmoniformes and therefore represent the best system of species from which a pre-duplication genomic state can be inferred.

Nucleotide BLASTN alignments between the flanking sequence of 5,918 mapped genomic markers from the Atlantic salmon linkage map [61] and the northern pike genome scaffolds produced 3,241 high-quality alignments. Of these alignments, 1,803 involved scaffolds assigned a linkage group on the northern pike genetic map. An analysis of these alignments clearly indicates that each *E. lucius* linkage group possesses significant conserved synteny with two Atlantic salmon chromosomes (Table 2). This observation is consistent with a historical duplication of each chromosome in the salmonid lineage that did not occur in an esocid ancestor. These results corroborate previous analyses which compared Atlantic salmon to stickleback [61]. Indeed, the increased relatedness and shared Protacanthopterygiian-specific rearrangements between Atlantic salmon and northern pike demonstrate an even greater example of shared synteny between these two species that are separated by a genome duplication.

**Table 2:** Sequences from mapped Atlantic salmon SNPs [61] blasted against the *Esox lucius* genome (≤1e-10).

		*Esox lucius* Linkage Groups																								
		1	2	3	4	5	6	7	8	9	10	11	12	13	14	15	16	17	18	19	20	21	22	23	24	25
***Salmo salar*** ** Chromosomes**	**Ssa01**					8	**28**								**44**	**41**										
	**Ssa02**									**45**							13				**40**					
	**Ssa03**			**37**								**43**														
	**Ssa04**							**28**																		**24**
	**Ssa05**				**28**												10				**47**					
	**Ssa06**											**52**							**43**							
	**Ssa07**																		3					**17**	**10**	
	**Ssa08**																									**15**
	**Ssa09**	**32**			**34**											**29**										
	**Ssa10**								**68**	3										**36**						
	**Ssa11**		**35**					10							**31**											
	**Ssa12**		3							**18**								**55**								
	**Ssa13**							**20**					**28**		11											
	**Ssa14**			**18**							**25**															
	**Ssa15**												**13**	5	8				**33**							
	**Ssa16**	3							22											**20**			**23**			
	**Ssa17**																						**22**	**23**		
	**Ssa18**					4	**29**																		**16**	
	**Ssa19**					**17**	3															**21**				
	**Ssa20**	**18**												**36**												
	**Ssa21**																**25**				6					
	**Ssa22**		4															**56**								
	**Ssa23**								**33**											12						
	**Ssa24**													**39**												
	**Ssa25**																**25**									
	**Ssa26**		**46**									3								3						
	**Ssa27**										**41**															
	**Ssa28**					**24**	8																			
	**Ssa29**																					**24**				
**Total blast hits**		**57**	**89**	**58**	**65**	**59**	**71**	**63**	**131**	**67**	**72**	**108**	**43**	**87**	**103**	**73**	**77**	**118**	**84**	**74**	**97**	**46**	**49**	**45**	**27**	**40**

The Atlantic salmon chromosome linked to each mapped SNP was plotted against the linkage group of the top *E. lucius* scaffold blast match. Results <3 for a given LG/chromosome comparison are removed for clarity.

This work in northern pike provides a core dataset with which to examine genome evolution following whole-genome duplication. The reference transcriptome allows for the functional analysis of ohnologue evolution while the genome assemblies facilitate the study of intron and promoter evolution as well as post-WGD gene and genome rearrangements. The role of transposable elements in both esocid and salmonid genome evolution and speciation can be further explored by inferring pre-duplication genomic repeat content, by dating periods of transposable element activity and by characterizing the abundance and character of transposable elements in this pre-duplication genome.

## Conclusions

This work represents the largest currently available collection of genomic data for a member of the Esociformes and the first genome assembly for a non-salmonid member of the Protacanthopterygii, an economically important superorder which bridges the evolutionary gap between the Ostariophysi and Neoteleostei. We present a genome assembly of 824 Mbp across 94,267 contigs and 878 Mbp across 5,688 scaffolds, representing an estimated 96% of the genome; 50% of the assembly is represented in the largest 318 scaffolds. Approximately 20% of the genome sequence is made up of repeat elements. Two transcriptome assemblies from multi-tissue RNA-seq are presented and FPKM values provide a baseline dataset of expression levels across 13 individual tissues. A microsatellite-based linkage map places 526 markers across 25 linkage groups, the expected number of linkage groups based on previous karyotypes. These markers further facilitate the mapping of 46% of the scaffold assembly sequence onto linkage groups. Comparisons between linkage-mapped scaffolds and three teleost genomes show that northern pike has a chromosomal distribution of genes very similar to that of the Neoteleostei, suggesting relatively low chromosomal translocation activity and an ancestral-like gene distribution. Gene order along individual chromosomes, however, exhibits significant reorganization. This dataset represents an important resource for future ecological and evolutionary work in northern pike and will assist in understanding the effects of whole-genome duplication on genome evolution, especially once the upcoming salmonid genomes become available.

## Methods and Materials

### Animal Care

In accordance with the Canadian Council on Animal Care Guidelines, the Animal Care Committee at the University of Victoria did not require ethical review as only archival tissue or tissue from fish that were harvested by various government agencies for their own purposes, was used in the course of the experiments.

### Whole-Genome Sequencing

For whole-genome sequencing, genomic DNA was extracted from the spleen of a single male pike individual from an introduced population to Charlie Lake, British Columbia, CANADA [66], using pooled DNA from both DNeasy (QIAGEN) and phenol [67] extractions. DNA libraries of 180 bp were prepared and sequenced (paired-end) by the Michael Smith Genome Sciences Centre; libraries of 2 kb and 5 kb fragments were constructed and sequenced by BGI (http://www.genomics.cn/en/index); all sequences were produced using Illumina HiSeq2000 instruments (San Diego, CA).

Following ALLPATHS-LG genome assembly, contigs were checked for long runs of ambiguous bases and trimmed/split if necessary. We removed contigs that were primarily vector sequence (BLASTN 1e-50). Furthermore, all contigs were trimmed internally and at terminal ends for any vector sequence according to NCBI's VecScreen search recommendations (www.ncbi.nlm.nih.gov/tools/vecscreen/). Duplicate contigs were removed and mitochondrial contigs were removed (BLASTN 1e-5). This resulting contig set was compared to GenBank's non-redundant nucleotide database for contamination (BLASTN 1e-25). Potential non-vertebrate sequence matches were flagged and manually inspected to determine classification. These contig sequences including scaffold information have been uploaded to NCBI under BioProject ID PRJNA221548, accession AZJR00000000.

### Transposable element library creation and genomic repeat annotation

The process used to create the northern pike transposable element (TE) library was based on that used by Wegrzyn et al. [68] to identify TE sequences in the Loblolly Pine genome. First, a custom repeat ‘seed’ library (CSL) containing 33,304 TE sequences was assembled from three sources: 1) a quality-checked repeat consensus library produced by the *de novo* repeat-finding program REPET (version 1.3.9.1; default settings; [69]); 2) the RepBase database of TE sequences (REPET-formatted v18.08; [70]); and 3) TE sequences previously identified in Esociformes or Salmoniformes and deposited in Genbank.

Following CSL creation, the CENSOR program [71] was used to identify all non-redundant instances of CSL sequences in the northern pike genome contigs. Subsequent filtering removed all CENSOR hits that were smaller than 80% of the length of the respective CSL query sequence. Sequences from long CENSOR hits were clustered using the uclust.global program of USEARCH [72]. USEARCH parameters were such that a sequence was only added to an existing cluster if it possessed at least 80% nucleotide similarity over 80% of its length to the cluster's representative ‘centroid’. Following clustering, a representative sequence was obtained for each cluster; if there were fewer than three sequences in the cluster the longest sequence was chosen as a representative, otherwise a consensus sequence was generated from a multiple sequence alignment (MSA). MSAs were built using the T-COFFEE program [73], which used alignment data from two sources: global alignment information from MUSCLE [74], [75] and local alignment information from USEARCH.

The representative sequences from all clusters were combined and sequences with strong BLASTX alignments (E-value ≤1e-10) to the UniprotKB/SwissProt protein database (July 2013 release) were manually examined and, if appropriate, removed as non-TE host genes. The remaining library sequences were classified in accordance with the Wicker et al. classification system [76] based on the occurrence of structural motifs and/or similarity to previously identified TEs. The classification process relied on both manual and automated processing of output from NCBI's BLAST+ software and the PASTEClassifier.py script included with REPET. The final northern pike TE library contained 26,628 sequences of which 14,820 were classified to at least the subclass level under Wicker's taxonomic system. Many of the in-house scripts used to create and validate the TE library utilized modules included in the BioPython library [77], [78].

The total amount of low-complexity DNA and TE-derived sequence within the genome was estimated using RepeatMasker (v4.0.3 [79]); RepeatMasker was configured to use RMBlast (version 2.2.23+) as its internal search engine. The abundance estimations for individual TE taxa were aggregated from RepeatMasker's annotation output file.

### RNA-seq and assembly

All tissues for RNA-seq were extracted from a single, juvenile, male pike caught November 1, 2011 in the Portage Diversion/Assiniboine River Floodway near Portage la Prairie, MB, Canada (N50.00747 W98.38181) as the water was beginning to freeze. The animal was euthanized and then frozen on dry ice until sampling, and subsequently at −80°C. RNA from all 13 tissues – brain, eye, gill, hind gut, head kidney, heart, kidney, liver, muscle, nose, stomach, spleen and testis - were extracted by mixer-mill (Retsch) homogenization in Trizol (Invitrogen), followed by column clean-up using the RNeasy kit (QIAGEN). Total RNA was submitted directly to BGI for Illumina sequencing; indexed samples were pooled (4 per RNA-seq lane, pooled along with other projects) and sequenced on an Illumina HiSeq2000.

Paired-end raw reads were quality-trimmed using Trimmomatic [80]. Trimmed reads from all tissues were combined and a *de novo* transcriptome assembly was created using the Trinity assembler [45]. Trinity used a minimum kmer count of 3 in order to reduce noise introduced by sequencing errors.

In order to generate a non-redundant reference set of transcripts, the resulting set of Trinity transcripts were filtered. These raw putative transcripts were reduced by retaining those that were characterized as full-length [10], those that had a significant BLASTX [81] match to the UniProtKB/Swiss-Prot or Gene Ontology protein databases (≤1e-5) without being transposable element annotations, and those that did not show any sequence homology to a known protein but had a predicted open reading frame ≥300 bp. This reduced set was mapped to our genome assembly using BLAT [82]. Transcripts mapping uniquely to a single loci were retained. In cases where multiple transcripts (from alternatively-spliced mRNAs) were mapped to the same genomic location, only the longest transcript was retained. We masked repeats using RepeatMasker [79] and our *Esox lucius* repeat library, removing open reading frames that were no longer ≥300 bp. To remove possible alleles, recent duplicates, and sequencing errors in our de novo assembly, we took a single representative of transcripts that were ≥98% similar over a minimum length of 300 bp, as determined by BLASTN. This curated set represents our RNA-seq reference transcriptome.

We produced an *ab initio* transcript set from our genome contig assembly using MAKER2 [83]. An Augustus [84] gene model specific for *Esox lucius* was generated using contigs from the RNA-seq assembly and the previously published EST assembly as evidence. MAKER2 was run for two rounds to produce an *Esox lucius* SNAP model. A final round of MAKER2 was run using the Augustus gene model, SNAP model, and the mRNA evidence.

Homology analysis of protein-coding mRNA data utilized the non-redundant protein-coding northern pike transcriptome (repeat-masked), Atlantic salmon EST data [9] and *Gasterosteus aculeatus* transcripts obtained from the UCSC genome browser. Intra-transcriptome comparisons for northern pike, Atlantic salmon and threespine stickleback, as well as between-species comparisons (northern pike vs. Atlantic salmon and northern pike vs. threespine stickleback) were performed using a reciprocal best BLASTN approach. Only BLASTN alignments longer than 100 bp and possessing an E-value ≤1e-5 were considered in establishing homology. DAVID analyses [85] were performed using paralogues from *Esox lucius* that exhibited 77–89% similarity in the aforementioned BLASTN alignments. Results were categorized by molecular function and biological process, and sorted by EASE-Score (p-value).

### RAD-tag based linkage mapping

A RAD-tag-based approach for linkage map construction was attempted following the protocol of Amores et al. [53] using the *Sbf*I restriction enzyme. Tissues from a family consisting of a single father and mother (fin clips) and 94 progeny (yolk-sac larvae) from the Hackettstown Fish Hatchery, New Jersey, USA were extracted and used to construct three barcoded Illumina libraries, each containing data from 32 individuals. These libraries were paired-end sequenced using an Illumina HiSeq-2000 sequencer (Michael Smith Genome Sciences Centre, Vancouver, BC, CANADA). Subsequent analysis to identify and score SNPs was performed with Stacks [86].

### Microsatellite Primer Design

A second family was obtained from the Hackettstown Fish Hatchery, New Jersey. Tissues stored in 95% ethanol from 103 progeny (either yolk-sac larvae or fin clips from 4-month old juveniles) of a half-sibling family produced from 3 fathers and a single mother were extracted by placing 2 mm^3^ of tissue in 5% Chelex 100 (Biorad), 0.2% SDS and 0.27 mg/ml proteinase K (Invitrogen) for 2 hours at 55°C followed by 95°C for 10 minutes [87]. Extractions were diluted 1/100 in water for use in PCR. Offspring were screened using microsatellite primers to determine parentage (see below for primer design); progeny were distributed 106∶45∶0 between the three fathers. The family was therefore used as a two-father/single mother half-sibling family in further analysis, split 48∶44 progeny per father to fit a 96-well format.

776 primer pairs were designed to target di- and tri-nucleotide repeats identified by Tandem Repeats finder [88] from *Esox* EST sequences [10], with an additional 835 primer pairs designed to target microsatellite repeats identified from genome scaffolds (1–3 primer pairs evaluated per scaffold). The unlabelled EST-designed primer pairs, u001-u296 were evaluated on a 5% polyacrylamide gel prior to re-ordering HEX or 6-FAM directly-labelled forward primers. The forward primer from each of the remaining primer pairs (u297–u776) and all scaffold primers were ordered with one of three tails, Tail-A, Tail-B or Tail-C for fluorescently labelling products with a labelled universal third primer, adapted from Blackett et al. protocol [89]. The universal primers were labelled: Tail-A [90] with 6-FAM, Tail-B [90] with HEX, Tail-C [89] with NED, 6-FAM and HEX.

For directly labelled primers, each PCR reaction contained: ∼2.5 ng template DNA, 200 µM each dNTP (Promega), 0.5 µM each forward (HEX or 6-FAM labelled) and reverse primer (IDT), 0.25 U Hot Start *Taq* DNA polymerase (Promega), 1X GoTaq Flexi Colorless PCR buffer (Promega), 2.0 mM MgCl_2_, to a final volume of 10 µl with DNase/RNase free H_2_O (Gibco). Reactions were cycled on a TC-412 thermocycler (Techne) following the profile: 3 min at 95°C followed by 35 cycles of 30 s at 95°C, 30 s at 52°C and 30 s at 72°C, and a final extension of 72°C for 10 min with a hold at 4°C. For “Tailed” reactions: ∼2.5 ng template DNA, 200 µM each dNTP (Promega), 0.15 µM forward primer (IDT), 0.5 µM reverse primer (IDT), 0.2 µM labelled universal tail primer (IDT), 0.25 U Hot Start *Taq* DNA polymerase (Promega), 1X GoTaq Flexi Colorless PCR buffer (Promega), 2.0 mM MgCl_2_, and final volume of 10 µl with DNase/RNase free H_2_O (Gibco). Tailed reactions were cycled on the same profile as above, except the 52°C annealing step was replaced with 56°C for 1 minute 30 seconds.

Following PCR, genotyping reactions consisted of 1.0 µl each PCR product, 9.9 µl Hi-Di Formamide (Life Technologies) and 0.1 µl GeneScan -500 ROXSize Standard (Life Technologies); two or three PCR reactions with different dyes were pooled into a single genotyping reaction. Following denaturation for 3–5 minutes at 95°C, samples were cooled on ice for 5–10 minutes before analysis on a 3730 DNA analyzer (Life Technologies). The generated electropherograms were analysed using GeneMapper V4.0 (Life Technologies), with automated scoring reviewed manually for errors in all samples. Samples for which clear peaks were not identified were removed from the dataset in subsequent analyses. All primer pairs were evaluated on the mother and two fathers of the family. Markers identified as polymorphic in the parents were used to amplify and score the distribution of alleles in the progeny. Polymorphic marker information is summarized in Table S7.

### Linkage Mapping

Linkage mapping was performed using the LINKMFEX package, v 2.3 (R. Danzmann, University of Guelph, http://www.uoguelph.ca/~rdanzman/software.htm) following the standard protocol; markers with more than 15% of genotypes missing were omitted from the analysis (apart from u0028c; see below). Linkage groups were identified at an LOD threshold of 4.0; linkage groups in the individual maps were further joined at an LOD of 3 if in another individual the linkage was recognized using the stricter cut-off. Further estimation of distances below LOD = 3 was used to create merged, sex-specific maps; these are represented by dotted lines in Figure S1. Merged sex-specific and final merged maps were produced using the MERGE programs in the LINKMFEX package. Maps were visualized using the program MAPCHART [91]. The linkage of u0028c (with more than 15% missing data in original family) to adjacent markers in LG-04 was confirmed using a separate, unrelated family from the Whiteshell fish hatchery (West Hawk Lake, Manitoba, CANADA), extracted, amplified and analysed using the previously described procedures.

### Synteny analyses

Four datasets were used for synteny analyses: 1) the reference *Esox lucius* transcriptome (see ‘RNA-seq and assembly’ section of Methods); 2) stickleback protein sequences from the Feb. 2006 Broad/gasAcu1 release; 3) zebrafish protein sequences from the Jul. 2010 Zv9/danRer7 release and; 4) medaka protein sequences from the Oct. 2005 NIG/UT MEDAKA1/oryLat 2 release. Stickleback, zebrafish and medaka sequences and their associated genomic location information were obtained from the UCSC Genome Browser [92]. Scaffold locations for northern pike transcripts were obtained through mapping using GMAP [93]; linkage group assignments followed if the host scaffold had been previously mapped to a group in the genetic map. Using the BLASTX and TBLASTN programs, BLAST alignments (E-value ≤1e-5) were obtained between the northern pike transcripts and the proteins of each other fish species. Orthology between northern pike transcripts and other fish protein sequences was determined using the reciprocal best hit (RBH) paradigm requiring at least 50% of each sequence was covered in non-overlapping BLAST alignments (HSPs) from the other. Synteny between two species (Figure 6) and scaffold continuity (Figure S2) were examined by plotting the genomic locations of each sequence in a relevant orthologue pair.

The analysis of synteny between northern pike and Atlantic salmon (Table 2) was performed by obtaining the flanking sequence of chromosome-associated SNPs in Atlantic salmon and identifying the strongest BLASTN hits (E-value ≤1e-10) between these sequences and northern pike scaffolds with a known linkage group.

## Supporting Information

Figure S1 **Individual male and sex-specific linkage maps for the northern pike.** Sex-specific maps and how markers are merged into the final, merged linkage map are presented. Individual linkage maps produced from each of the two fathers are also presented, as well as positions for which a distance was estimated below an LOD of 3.0 for efficient merging. Star symbol denotes markers designed from the same scaffold that appear disrupted by one or more additional scaffolds in the merged linkage map, due to lack of shared informative markers in merging sex-specific maps.
(PDF)

Figure S2 **Conservation of gene order within the largest 50 northern pike genomic scaffolds relative to** ***Gasterosteus aculeatus*** **,** ***Oryzias latipes*** **, and** ***Danio rerio*** **.** Reciprocal best hit results (≥50% of total length, ≤1e-5) and mapped scaffold position plotted. Average position ((Start position + End position)/2) taken to represent a point position for each mapped transcript.
(PDF)

Table S1 **Contribution of major Transposable Element categories to the** ***Esox lucius*** **genome.** Produced by parsing results from Repeat masker output; variation between the direct Repeat masker output (18.1% of genome) and the sum of the annotated categories is likely due to overlap in the masked elements.
(XLSX)

Table S2 **Trinity-assembly FPKM results for 13** ***Esox lucius*** **tissues.** All values submitted to NCBI GEO profile database.
(XLSX)

Table S3 **MAKER2-assembly FPKM results for 13** ***Esox lucius*** **tissues.**
(XLSX)

Table S4 **Top 10 expressed specialized transcripts from each tissue in** ***Esox lucius*** **.** Sorted by highest FPKM for transcripts >3 standard deviations above average across all tissues; based on results from 38K Trinity-assembled transcriptome.
(XLSX)

Table S5 **Pathways Identified in duplicated transcripts.** Significant DAVID results (sorted by p-value) for duplicated transcripts between 77–89% similarity in northern pike. Gene_Ontology terms presented from the molecular function (GOTERM_MF_FAT) and biological process (GOTERM_BP_FAT) categories.
(XLSX)

Table S6 **Comparative synteny between** ***Esox lucius*** **and three model organisms.** Reciprocal blast results (≥50% of total length, ≤1e-5) between scaffold-linked *E. lucius* transcripts and published genome assemblies of A) *Gasterosteus aculeatus* (gasAcu1 – Feb 2006), B) *Oryzias latipes* (oryLat2 – Oct 2005) and C) *Danio rerio* (ZV9 - Jul 2010). The table provides numerical results used to produce Figure 6.
(XLSX)

Table S7 **Microsatellite marker information and primer sequences used in construction of first-generation linkage map.** Includes relevant information regarding the location of the microsatellite marker within the scaffolds, the primers used to amplify the repeats, and the results from tandem repeats finder representing information on the repeats, based on the EST/scaffold sequence used to design the primer pairs targeting the repeats.
(XLSX)

File S1 **19K MAKER2-assembled transcriptome in FASTA format.**
(GZ) Click here for additional data file.
