# Supplementary material for: The Genome and Linkage Map of the Northern Pike (Esox lucius): Conserved Synteny Revealed between the Salmonid Sister Group and the Neoteleostei
Source: PLoS One. 2014 Jul 28;9(7):e102089. doi: 10.1371/journal.pone.0102089 (PMC4113312; doi:10.1371/journal.pone.0102089)
Supplement: Figure S1 — Individual male and sex-specific linkage maps for the northern pike. Sex-specific maps and how markers are merged into the final, merged linkage map are presented. Individual linkage maps produced from each of the two fathers are also presented, as well as positions for which a distance was estimated below an LOD of 3.0 for efficient merging. Star symbol denotes markers designed from the same scaffold that appear disrupted by one or more additional scaffolds in the merged linkage map, due to lack of shared informative markers in merging sex-specific maps. (PDF) [file pone.0102089.s001.pdf]

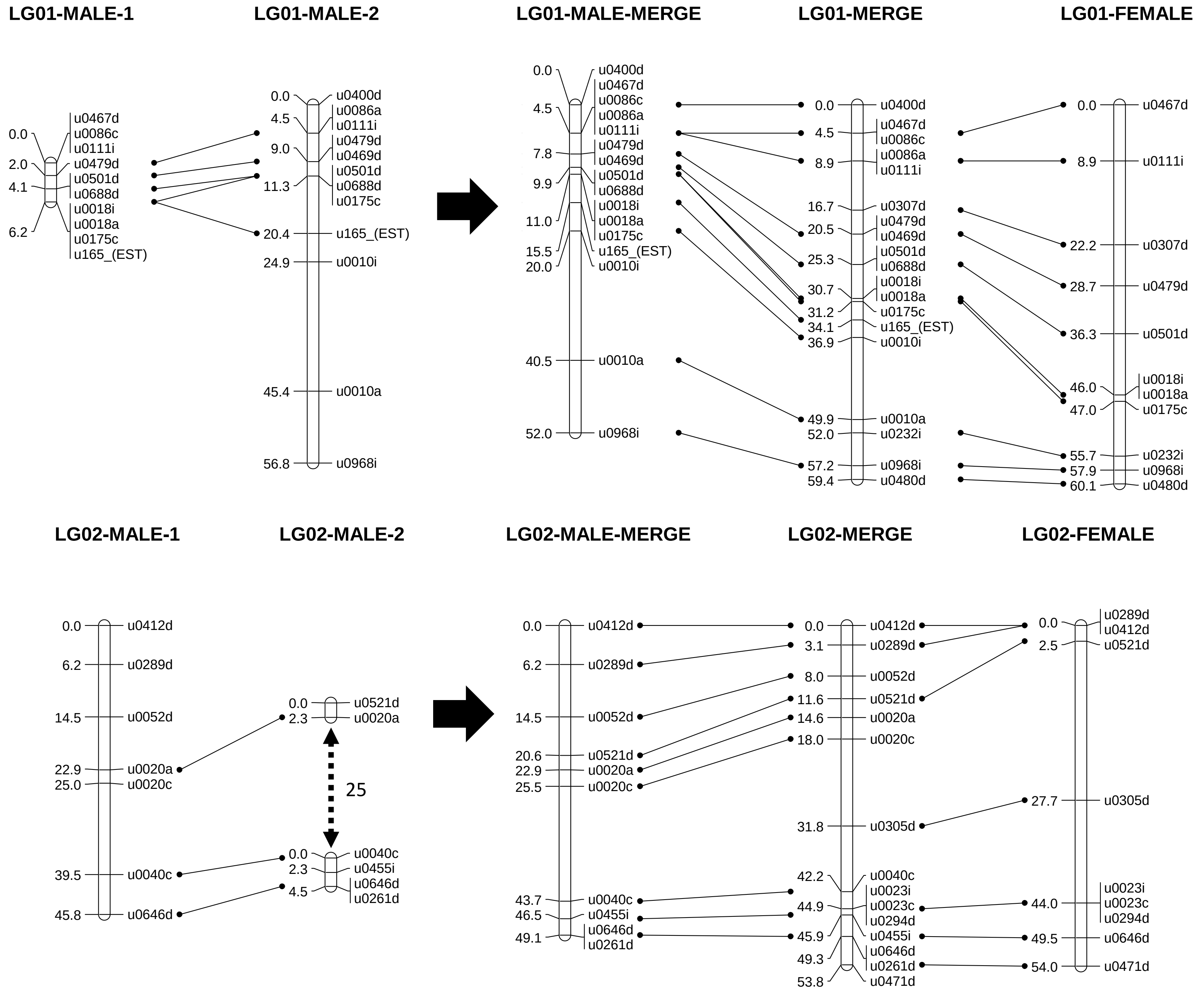

LG01-MALE-1
0.0 u0467d u0086c u0111i
2.0 u0479d
4.1 u0501d u0688d
6.2 u0018i u0018a u0175c u165_(EST)
LG01-MALE-2
0.0 u0400d
4.5 u0086a u0111i
9.0 u0479d u0469d
11.3 u0501d u0688d u0175c
20.4 u165_(EST)
24.9 u0010i
45.4 u0010a
56.8 u0968i
LG01-MALE-MERGE
0.0 u0400d
4.5 u0467d u0086c u0086a u0111i
7.8 u0479d u0469d
9.9 u0501d u0688d
11.0 u0018i u0018a u0175c
15.5 u165_(EST)
20.0 u0010i
40.5 u0010a
52.0 u0968i
LG01-MERGE
0.0 u0400d
4.5 u0467d u0086c
8.9 u0086a u0111i
16.7 u0307d
20.5 u0479d u0469d
25.3 u0501d u0688d
30.7 u0018i u0018a
31.2 u0175c
34.1 u165_(EST)
36.9 u0010i
49.9 u0010a
52.0 u0232i
57.2 u0968i
59.4 u0480d
LG01-FEMALE
0.0 u0467d
8.9 u0111i
22.2 u0307d
28.7 u0479d
36.3 u0501d
46.0 u0018i u0018a
47.0 u0175c
55.7 u0232i
57.9 u0968i
60.1 u0480d
LG02-MALE-1
0.0 u0412d
6.2 u0289d
14.5 u0052d
22.9 u0020a
25.0 u0020c
39.5 u0040c
45.8 u0646d
LG02-MALE-2
0.0 u0521d
2.3 u0020a
25
0.0 u0040c
2.3 u0455i
4.5 u0646d u0261d
LG02-MALE-MERGE
0.0 u0412d
6.2 u0289d
14.5 u0052d
20.6 u0521d
22.9 u0020a
25.5 u0020c
43.7 u0040c
46.5 u0455i
49.1 u0646d u0261d
LG02-MERGE
0.0 u0412d
3.1 u0289d
8.0 u0052d
11.6 u0521d
14.6 u0020a
18.0 u0020c
31.8 u0305d
42.2 u0040c
44.9 u0023i u0023c u0294d
45.9 u0455i
49.3 u0646d u0261d
53.8 u0471d
LG02-FEMALE
0.0 u0289d u0412d
2.5 u0521d
27.7 u0305d
44.0 u0023i u0023c u0294d
49.5 u0646d
54.0 u0471d

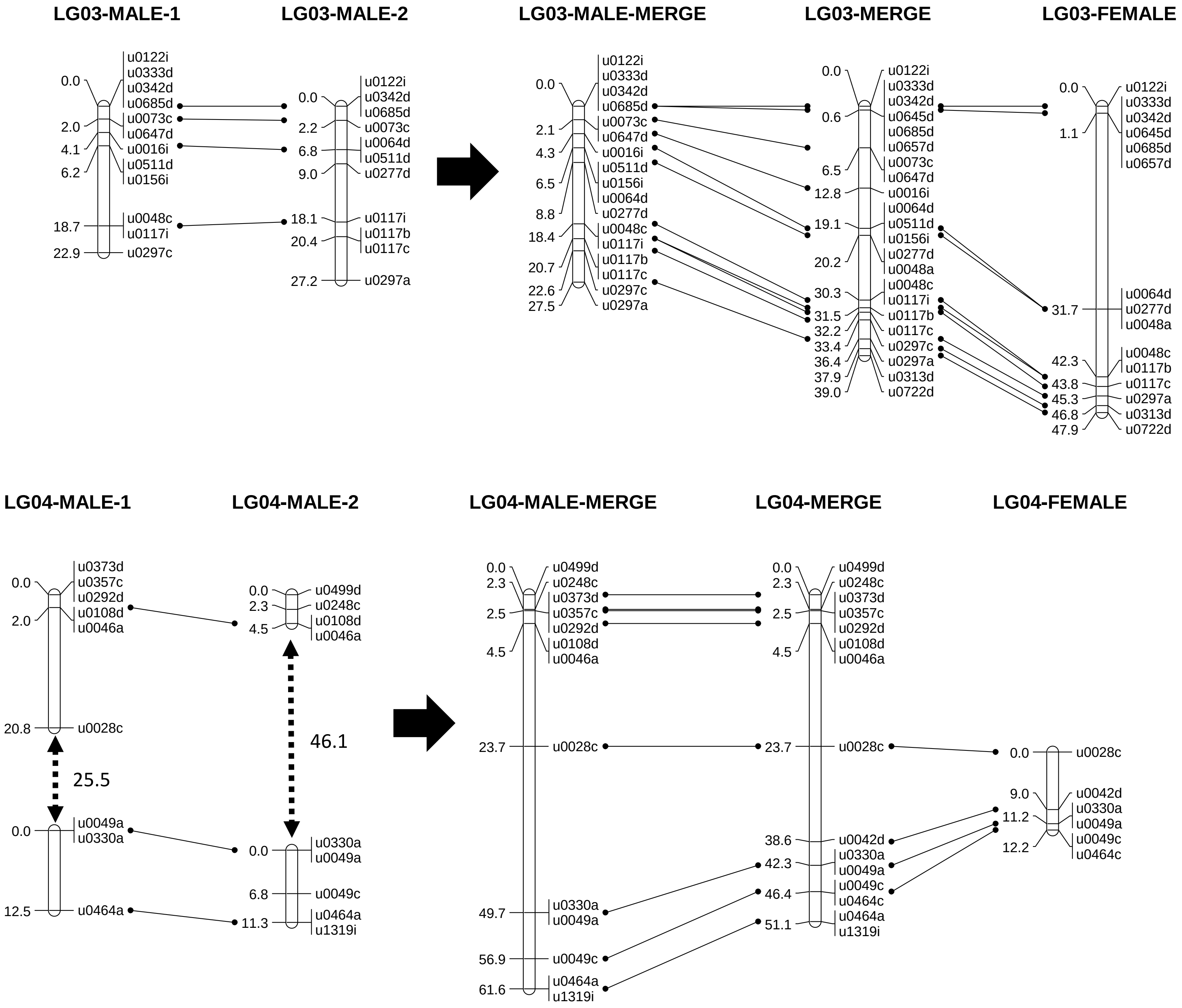

LG03-MALE-1
0.0 u0122i u0333d u0342d u0685d
2.0 u0073c u0647d
4.1 u0016i
6.2 u0511d u0156i
18.7 u0048c u0117i
22.9 u0297c
LG03-MALE-2
0.0 u0122i u0342d u0685d
2.2 u0073c
6.8 u0064d u0511d
9.0 u0277d
18.1 u0117i
20.4 u0117b u0117c
27.2 u0297a
LG03-MALE-MERGE
0.0 u0122i u0333d u0342d u0685d
2.1 u0073c
4.3 u0647d
6.5 u0016i
8.8 u0511d u0156i u0064d u0277d
18.4 u0048c
20.7 u0117i
22.6 u0117b u0117c u0297c
27.5 u0297a
LG03-MERGE
0.0 u0122i u0333d u0342d
0.6 u0645d u0685d u0657d
6.5 u0073c u0647d
12.8 u0016i
19.1 u0064d u0511d u0156i
20.2 u0277d u0048a
30.3 u0048c
31.5 u0117i
32.2 u0117b
33.4 u0117c
36.4 u0297c
37.9 u0297a
39.0 u0313d u0722d
LG03-FEMALE
0.0 u0122i
1.1 u0333d u0342d u0645d u0685d u0657d
31.7 u0064d u0277d u0048a
42.3 u0048c u0117b
43.8 u0117c
45.3 u0297a
46.8 u0313d
47.9 u0722d
LG04-MALE-1
0.0 u0373d u0357c u0292d
2.0 u0108d u0046a
20.8 u0028c
25.5
0.0 u0049a u0330a
12.5 u0464a
LG04-MALE-2
0.0 u0499d
2.3 u0248c
4.5 u0108d u0046a
46.1
0.0 u0330a u0049a
6.8 u0049c
11.3 u0464a u1319i
LG04-MALE-MERGE
0.0 u0499d
2.3 u0248c
2.5 u0373d u0357c u0292d
4.5 u0108d u0046a
23.7 u0028c
49.7 u0330a u0049a
56.9 u0049c
61.6 u0464a u1319i
LG04-MERGE
0.0 u0499d
2.3 u0248c
2.5 u0373d u0357c u0292d
4.5 u0108d u0046a
23.7 u0028c
38.6 u0042d
42.3 u0330a u0049a
46.4 u0049c u0464c
51.1 u0464a u1319i
LG04-FEMALE
0.0 u0028c
9.0 u0042d
11.2 u0330a u0049a
12.2 u0049c u0464c

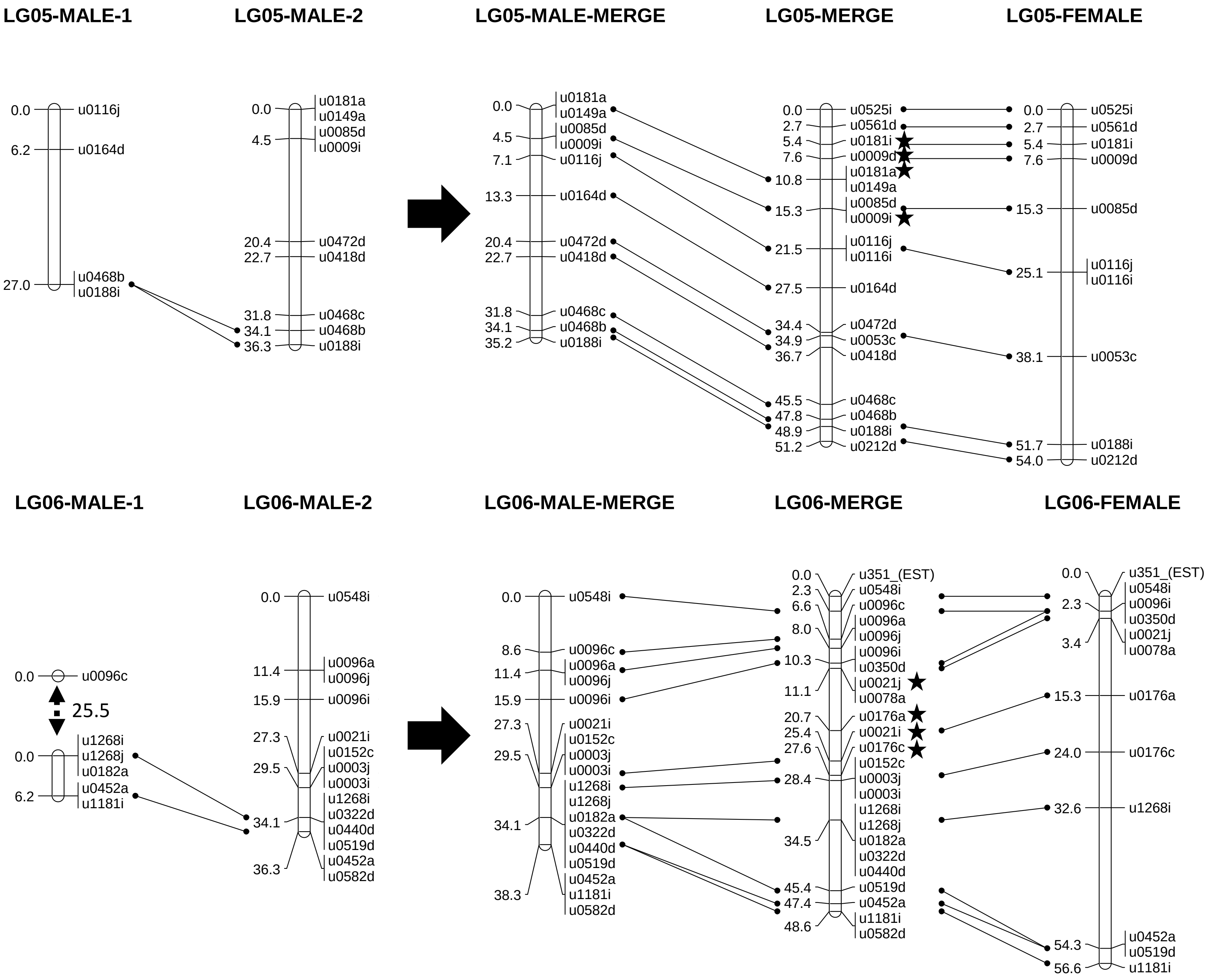
LG05-MALE-1
LG05-MALE-2
LG05-MALE-MERGE
LG05-MERGE
LG05-FEMALE
0.0 u0116j
6.2 u0164d
27.0 u0468b u0188i
0.0 u0181a u0149a
4.5 u0085d u0009i
20.4 u0472d
22.7 u0418d
31.8 u0468c
34.1 u0468b
36.3 u0188i
0.0 u0181a u0149a
4.5 u0085d u0009i
7.1 u0116j
13.3 u0164d
20.4 u0472d
22.7 u0418d
31.8 u0468c
34.1 u0468b
35.2 u0188i
0.0 u0525i
2.7 u0561d
5.4 u0181i
7.6 u0009d
10.8 u0181a u0149a
15.3 u0085d u0009i
21.5 u0116j u0116i
27.5 u0164d
34.4 u0472d
34.9 u0053c
36.7 u0418d
45.5 u0468c
47.8 u0468b
48.9 u0188i
51.2 u0212d
0.0 u0525i
2.7 u0561d
5.4 u0181i
7.6 u0009d
15.3 u0085d
25.1 u0116j u0116i
38.1 u0053c
51.7 u0188i
54.0 u0212d
LG06-MALE-1
LG06-MALE-2
LG06-MALE-MERGE
LG06-MERGE
LG06-FEMALE
0.0 u0096c
25.5
0.0 u1268i u1268j u0182a
6.2 u0452a u1181i
0.0 u0548i
11.4 u0096a u0096j
15.9 u0096i
27.3 u0021i u0152c
29.5 u0003j u0003i
34.1 u1268i u0322d u0440d u0519d
36.3 u0452a u0582d
0.0 u0548i
8.6 u0096c
11.4 u0096a u0096j
15.9 u0096i
27.3 u0021i u0152c u0003j
29.5 u0003i u1268i u1268j
34.1 u0182a u0322d u0440d u0519d
38.3 u0452a u1181i u0582d
0.0 u351_(EST)
2.3 u0548i
6.6 u0096c
8.0 u0096a u0096j
10.3 u0096i u0350d
11.1 u0021j u0078a
20.7 u0176a
25.4 u0021i
27.6 u0176c
28.4 u0152c u0003j u0003i
34.5 u1268i u1268j u0182a u0322d u0440d
45.4 u0519d
47.4 u0452a
48.6 u1181i u0582d
0.0 u351_(EST)
2.3 u0548i u0096i u0350d
3.4 u0021j u0078a
15.3 u0176a
24.0 u0176c
32.6 u1268i
54.3 u0452a u0519d
56.6 u1181i

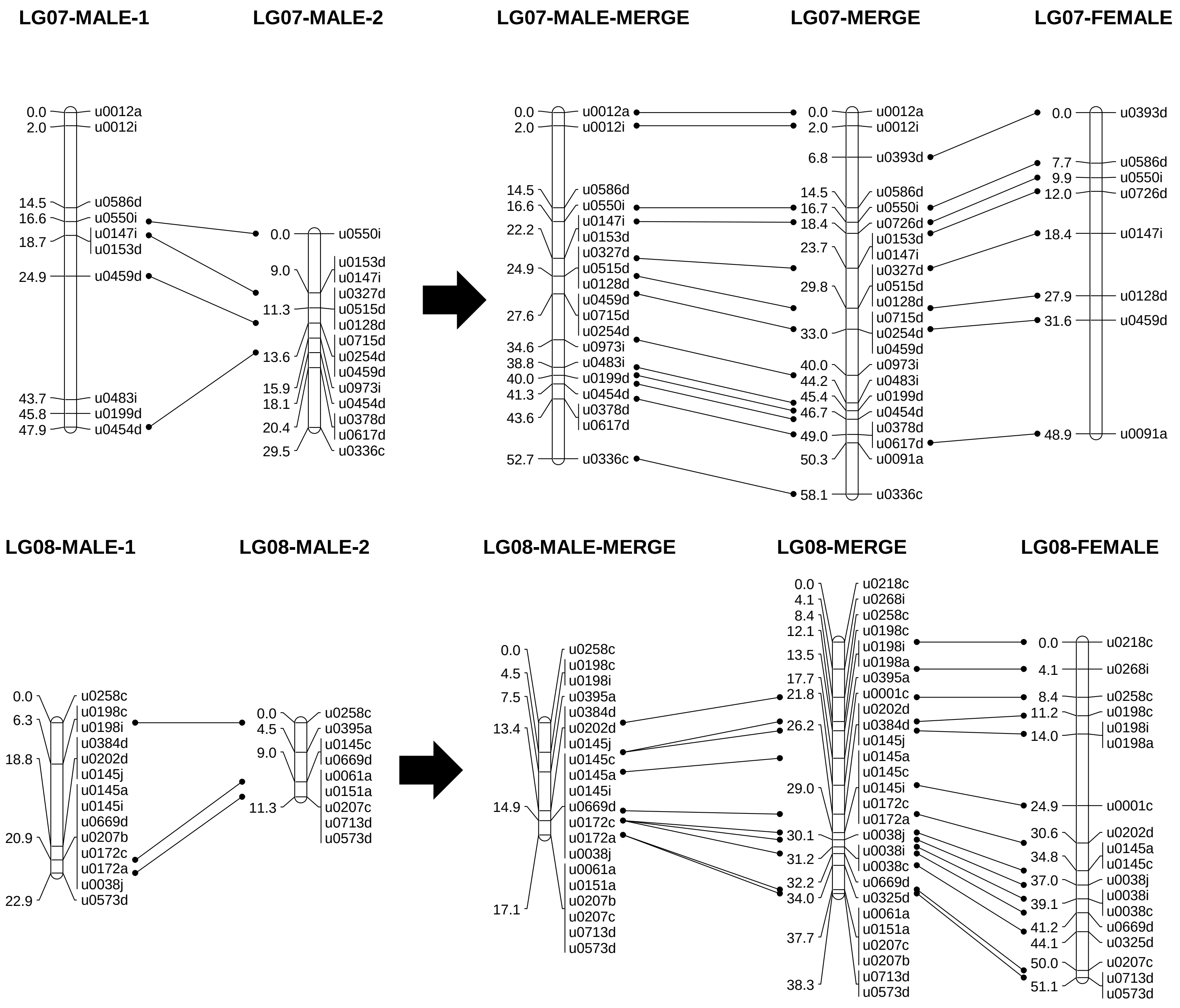
LG07-MALE-1
0.0 u0012a
2.0 u0012i
14.5 u0586d
16.6 u0550i
18.7 u0147i u0153d
24.9 u0459d
43.7 u0483i
45.8 u0199d
47.9 u0454d
LG07-MALE-2
0.0 u0550i
9.0 11.3 13.6 15.9 18.1 20.4 29.5
u0153d u0147i u0327d u0515d u0128d u0715d u0254d u0459d u0973i u0454d u0378d u0617d u0336c
LG07-MALE-MERGE
0.0 u0012a
2.0 u0012i
14.5 16.6 22.2 24.9 27.6 34.6 38.8 40.0 41.3 43.6 52.7
u0586d u0550i u0147i u0153d u0327d u0515d u0128d u0459d u0715d u0254d u0973i u0483i u0199d u0454d u0378d u0617d u0336c
LG07-MERGE
0.0 u0012a
2.0 u0012i
6.8 u0393d
14.5 16.7 18.4 23.7 29.8 33.0 40.0 44.2 45.4 46.7 49.0 50.3 58.1
u0586d u0550i u0726d u0153d u0147i u0327d u0515d u0128d u0715d u0254d u0459d u0973i u0483i u0199d u0454d u0378d u0617d u0091a u0336c
LG07-FEMALE
0.0 u0393d
7.7 u0586d
9.9 u0550i
12.0 u0726d
18.4 u0147i
27.9 u0128d
31.6 u0459d
48.9 u0091a
LG08-MALE-1
0.0 6.3 18.8 20.9 22.9
u0258c u0198c u0198i u0384d u0202d u0145j u0145a u0145i u0669d u0207b u0172c u0172a u0038j u0573d
LG08-MALE-2
0.0 4.5 9.0 11.3
u0258c u0395a u0145c u0669d u0061a u0151a u0207c u0713d u0573d
LG08-MALE-MERGE
0.0 4.5 7.5 13.4 14.9 17.1
u0258c u0198c u0198i u0395a u0384d u0202d u0145j u0145c u0145a u0145i u0669d u0172c u0172a u0038j u0061a u0151a u0207b u0207c u0713d u0573d
LG08-MERGE
0.0 4.1 8.4 12.1 13.5 17.7 21.8 26.2 29.0 30.1 31.2 32.2 34.0 37.7 38.3
u0218c u0268i u0258c u0198c u0198i u0198a u0395a u0001c u0202d u0384d u0145j u0145a u0145c u0145i u0172c u0172a u0038j u0038i u0038c u0669d u0325d u0061a u0151a u0207c u0207b u0713d u0573d
LG08-FEMALE
0.0 u0218c
4.1 u0268i
8.4 u0258c
11.2 u0198c
14.0 u0198i u0198a
24.9 u0001c
30.6 u0202d
34.8 u0145a u0145c
37.0 u0038j
39.1 u0038i u0038c
41.2 u0669d
44.1 u0325d
50.0 u0207c
51.1 u0713d u0573d

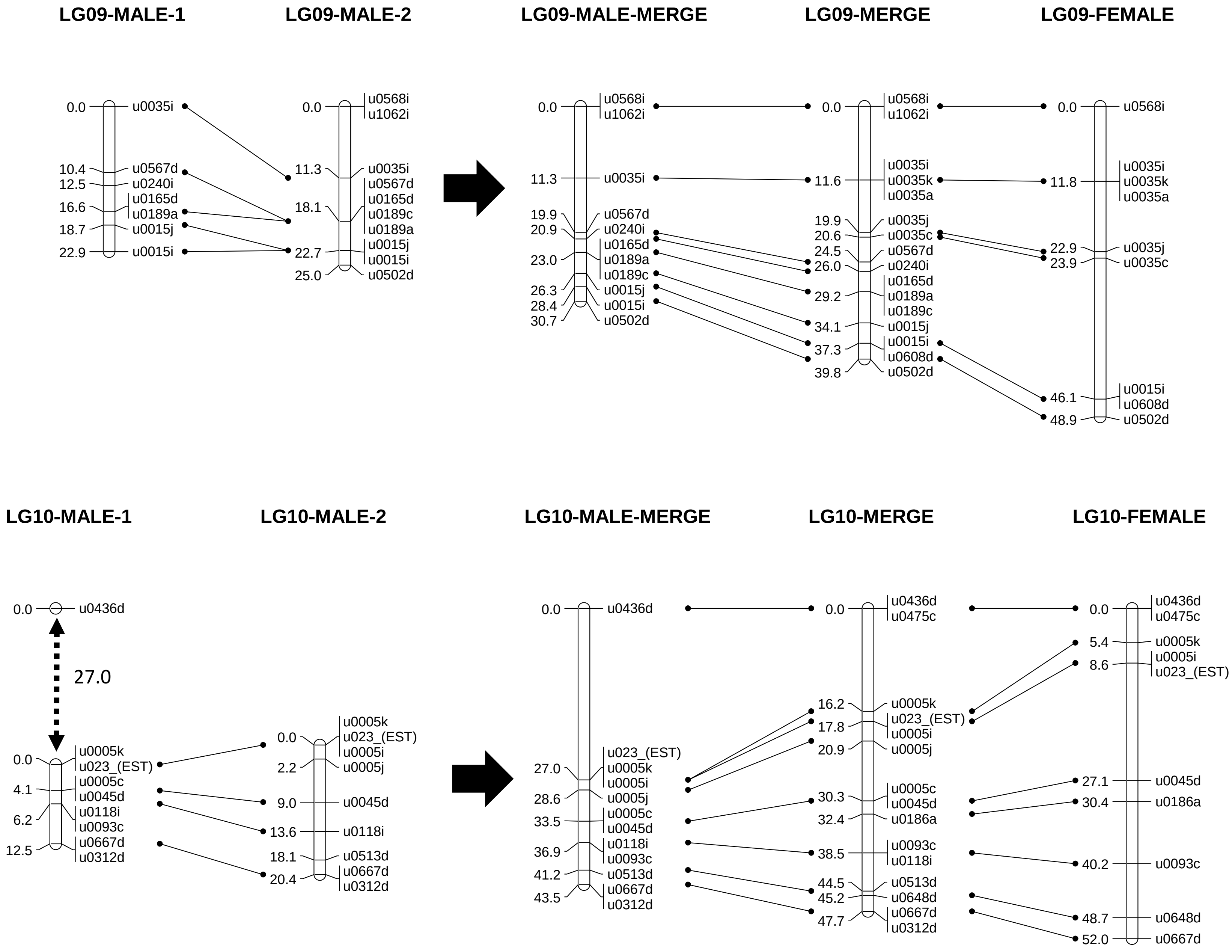
LG09-MALE-1
LG09-MALE-2
LG09-MALE-MERGE
LG09-MERGE
LG09-FEMALE
0.0 u0035i
10.4 u0567d
12.5 u0240i
16.6 u0165d u0189a
18.7 u0015j
22.9 u0015i
0.0 u0568i u1062i
11.3 u0035i
18.1 u0567d u0165d u0189c u0189a
22.7 u0015j u0015i
25.0 u0502d
0.0 u0568i u1062i
11.3 u0035i
19.9 u0567d
20.9 u0240i
23.0 u0165d u0189a u0189c
26.3 u0015j
28.4 u0015i
30.7 u0502d
0.0 u0568i u1062i
11.6 u0035i u0035k u0035a
19.9 u0035j
20.6 u0035c
24.5 u0567d
26.0 u0240i
29.2 u0165d u0189a u0189c
34.1 u0015j
37.3 u0015i u0608d
39.8 u0502d
0.0 u0568i
11.8 u0035i u0035k u0035a
22.9 u0035j
23.9 u0035c
46.1 u0015i u0608d
48.9 u0502d
LG10-MALE-1
LG10-MALE-2
LG10-MALE-MERGE
LG10-MERGE
LG10-FEMALE
0.0 u0436d
27.0
0.0 u0005k u023_(EST)
4.1 u0005c u0045d
6.2 u0118i u0093c
12.5 u0667d u0312d
0.0 u0005k u023_(EST) u0005i
2.2 u0005j
9.0 u0045d
13.6 u0118i
18.1 u0513d
20.4 u0667d u0312d
0.0 u0436d
27.0 u023_(EST) u0005k u0005i
28.6 u0005j
33.5 u0005c u0045d
36.9 u0118i u0093c
41.2 u0513d
43.5 u0667d u0312d
0.0 u0436d u0475c
16.2 u0005k
17.8 u023_(EST) u0005i
20.9 u0005j
30.3 u0005c u0045d
32.4 u0186a
38.5 u0093c u0118i
44.5 u0513d
45.2 u0648d
47.7 u0667d u0312d
0.0 u0436d u0475c
5.4 u0005k
8.6 u0005i u023_(EST)
27.1 u0045d
30.4 u0186a
40.2 u0093c
48.7 u0648d
52.0 u0667d

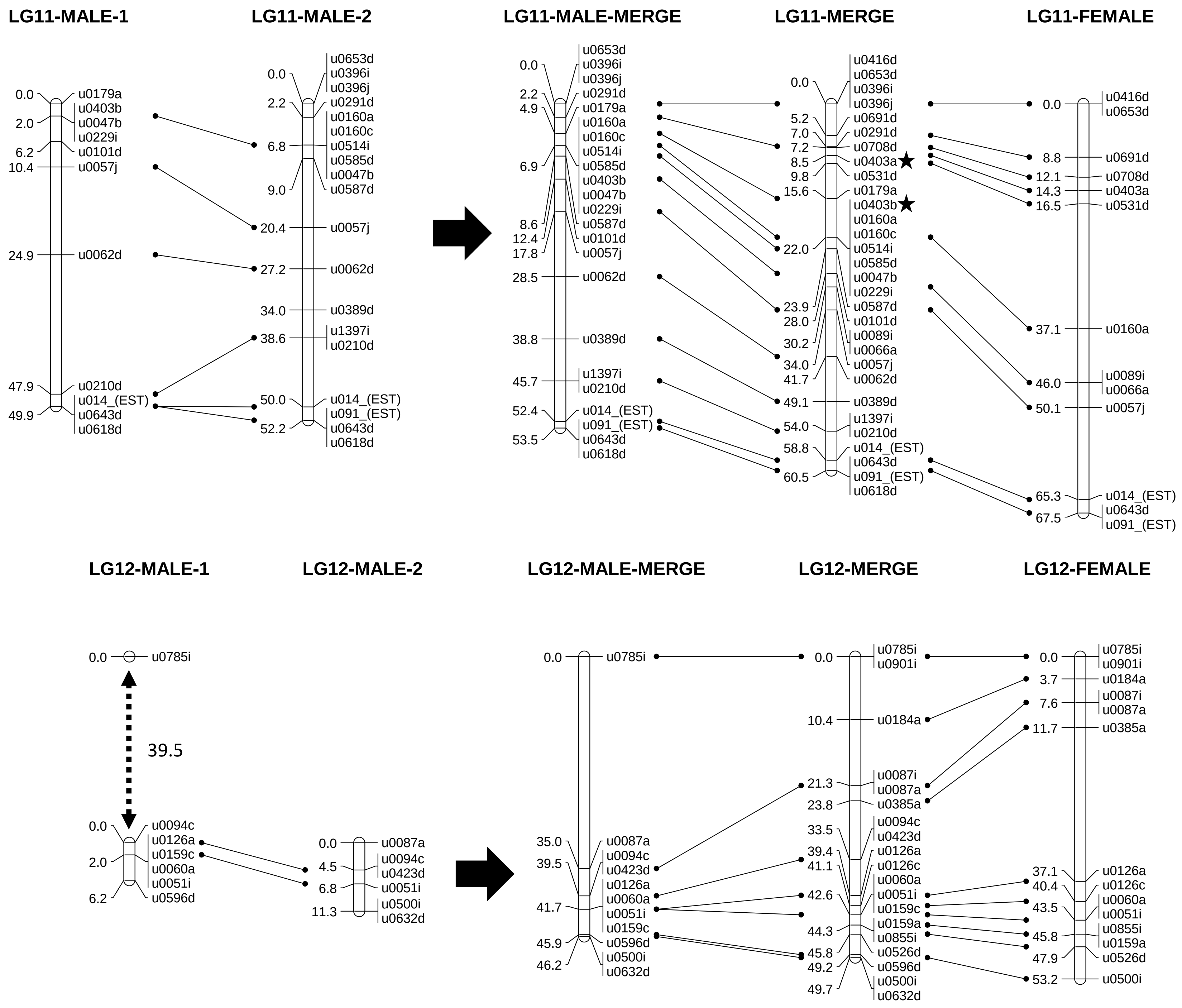

LG11-MALE-1
0.0 u0179a
2.0 u0403b u0047b u0229i
6.2 u0101d
10.4 u0057j
24.9 u0062d
47.9 u0210d
49.9 u014_(EST) u0643d u0618d
LG11-MALE-2
0.0 u0653d u0396i u0396j
2.2 u0291d
6.8 u0160a u0160c u0514i u0585d u0047b
9.0 u0587d
20.4 u0057j
27.2 u0062d
34.0 u0389d
38.6 u1397i u0210d
50.0 u014_(EST)
52.2 u091_(EST) u0643d u0618d
LG11-MALE-MERGE
0.0 u0653d u0396i u0396j
2.2 u0291d
4.9 u0179a
6.9 u0160a u0160c u0514i u0585d u0403b u0047b u0229i
8.6 u0587d
12.4 u0101d
17.8 u0057j
28.5 u0062d
38.8 u0389d
45.7 u1397i u0210d
52.4 u014_(EST)
53.5 u091_(EST) u0643d u0618d
LG11-MERGE
0.0 u0416d u0653d u0396i u0396j
5.2 u0691d
7.0 u0291d
7.2 u0708d
8.5 u0403a★
9.8 u0531d
15.6 u0179a
22.0 u0403b★ u0160a u0160c u0514i u0585d u0047b u0229i
23.9 u0587d
28.0 u0101d
30.2 u0089i u0066a
34.0 u0057j
41.7 u0062d
49.1 u0389d
54.0 u1397i u0210d
58.8 u014_(EST)
60.5 u0643d u091_(EST) u0618d
LG11-FEMALE
0.0 u0416d u0653d
8.8 u0691d
12.1 u0708d
14.3 u0403a
16.5 u0531d
37.1 u0160a
46.0 u0089i u0066a
50.1 u0057j
65.3 u014_(EST)
67.5 u0643d u091_(EST)
LG12-MALE-1
0.0 u0785i
39.5
0.0 u0094c
2.0 u0126a u0159c u0060a u0051i
6.2 u0596d
LG12-MALE-2
0.0 u0087a
4.5 u0094c u0423d
6.8 u0051i
11.3 u0500i u0632d
LG12-MALE-MERGE
0.0 u0785i
35.0 u0087a
39.5 u0094c u0423d
41.7 u0126a u0060a u0051i u0159c
45.9 u0596d
46.2 u0500i u0632d
LG12-MERGE
0.0 u0785i u0901i
10.4 u0184a
21.3 u0087i u0087a
23.8 u0385a
33.5 u0094c u0423d
39.4 u0126a
41.1 u0126c u0060a
42.6 u0051i u0159c
44.3 u0159a u0855i
45.8 u0526d
49.2 u0596d
49.7 u0500i u0632d
LG12-FEMALE
0.0 u0785i u0901i
3.7 u0184a
7.6 u0087i u0087a
11.7 u0385a
37.1 u0126a
40.4 u0126c
43.5 u0060a u0051i
45.8 u0855i u0159a
47.9 u0526d
53.2 u0500i

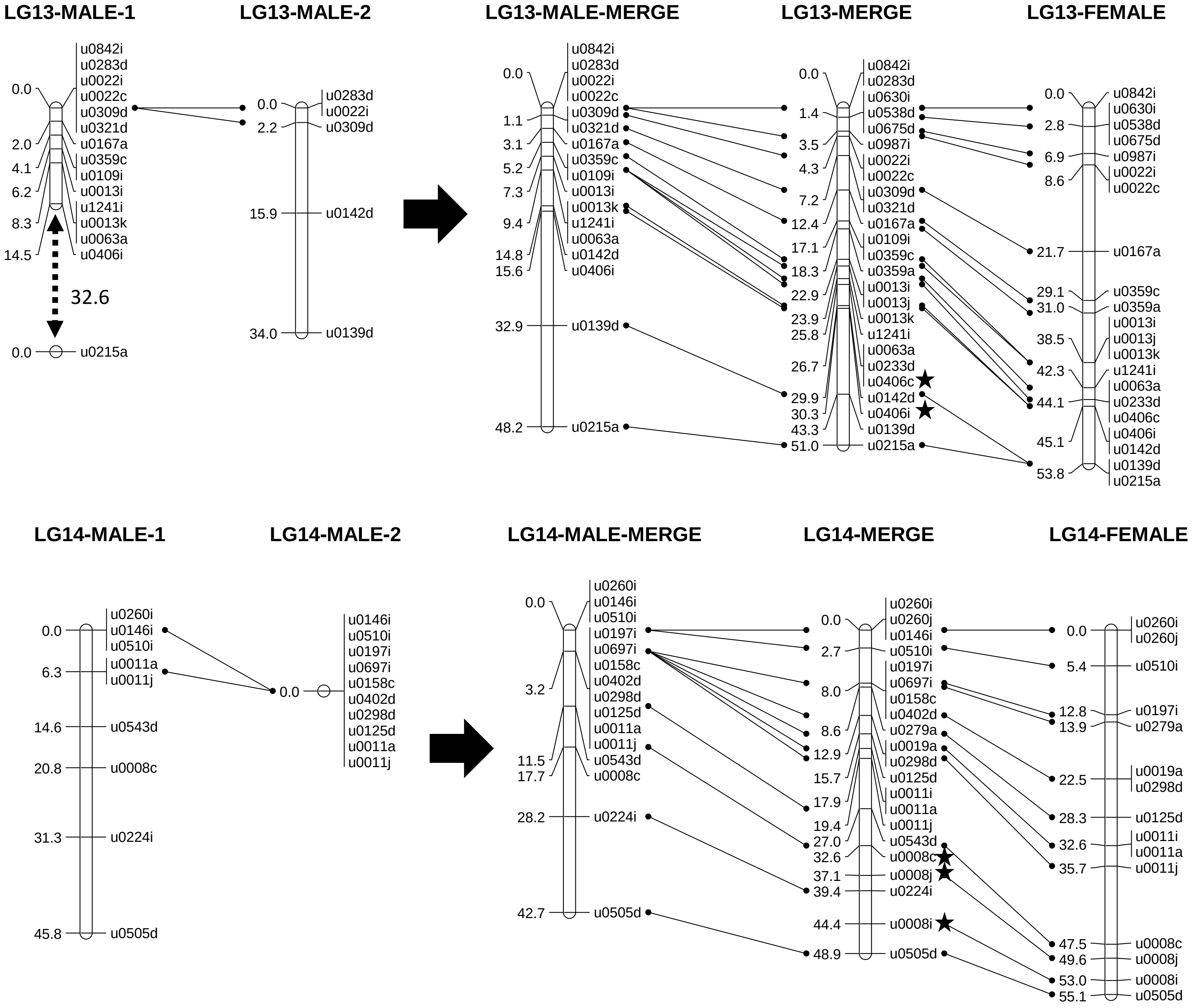
LG13-MALE-1
0.0
2.0
4.1
6.2
8.3
14.5
u0842i
u0283d
u0022i
u0022c
u0309d
u0321d
u0167a
u0359c
u0109i
u0013i
u1241i
u0013k
u0063a
u0406i
32.6
0.0 u0215a
LG13-MALE-2
0.0 u0283d
u0022i
2.2 u0309d
15.9 u0142d
34.0 u0139d
LG13-MALE-MERGE
0.0
1.1
3.1
5.2
7.3
9.4
14.8
15.6
u0842i
u0283d
u0022i
u0022c
u0309d
u0321d
u0167a
u0359c
u0109i
u0013i
u0013k
u1241i
u0063a
u0142d
u0406i
32.9 u0139d
48.2 u0215a
LG13-MERGE
0.0
1.4
3.5
4.3
7.2
12.4
17.1
18.3
22.9
23.9
25.8
26.7
29.9
30.3
43.3
51.0
u0842i
u0283d
u0630i
u0538d
u0675d
u0987i
u0022i
u0022c
u0309d
u0321d
u0167a
u0109i
u0359c
u0359a
u0013i
u0013j
u0013k
u1241i
u0063a
u0233d
u0406c
u0142d
u0406i
u0139d
u0215a
LG13-FEMALE
0.0
2.8
6.9
8.6
u0842i
u0630i
u0538d
u0675d
u0987i
u0022i
u0022c
21.7 u0167a
29.1 u0359c
31.0 u0359a
38.5
42.3
44.1
45.1
53.8
u0013i
u0013j
u0013k
u1241i
u0063a
u0233d
u0406c
u0406i
u0142d
u0139d
u0215a
LG14-MALE-1
0.0 u0260i
u0146i
u0510i
6.3 u0011a
u0011j
14.6 u0543d
20.8 u0008c
31.3 u0224i
45.8 u0505d
LG14-MALE-2
0.0
u0146i
u0510i
u0197i
u0697i
u0158c
u0402d
u0298d
u0125d
u0011a
u0011j
LG14-MALE-MERGE
0.0
3.2
11.5
17.7
u0260i
u0146i
u0510i
u0197i
u0697i
u0158c
u0402d
u0298d
u0125d
u0011a
u0011j
u0543d
u0008c
28.2 u0224i
42.7 u0505d
LG14-MERGE
0.0
2.7
8.0
8.6
12.9
15.7
17.9
19.4
27.0
32.6
37.1
39.4
44.4
48.9
u0260i
u0260j
u0146i
u0510i
u0197i
u0697i
u0158c
u0402d
u0279a
u0019a
u0298d
u0125d
u0011i
u0011a
u0011j
u0543d
u0008c
u0008j
u0224i
u0008i
u0505d
LG14-FEMALE
0.0 u0260i
u0260j
5.4 u0510i
12.8 u0197i
13.9 u0279a
22.5 u0019a
u0298d
28.3 u0125d
32.6 u0011i
u0011a
35.7 u0011j
47.5 u0008c
49.6 u0008j
53.0 u0008i
55.1 u0505d

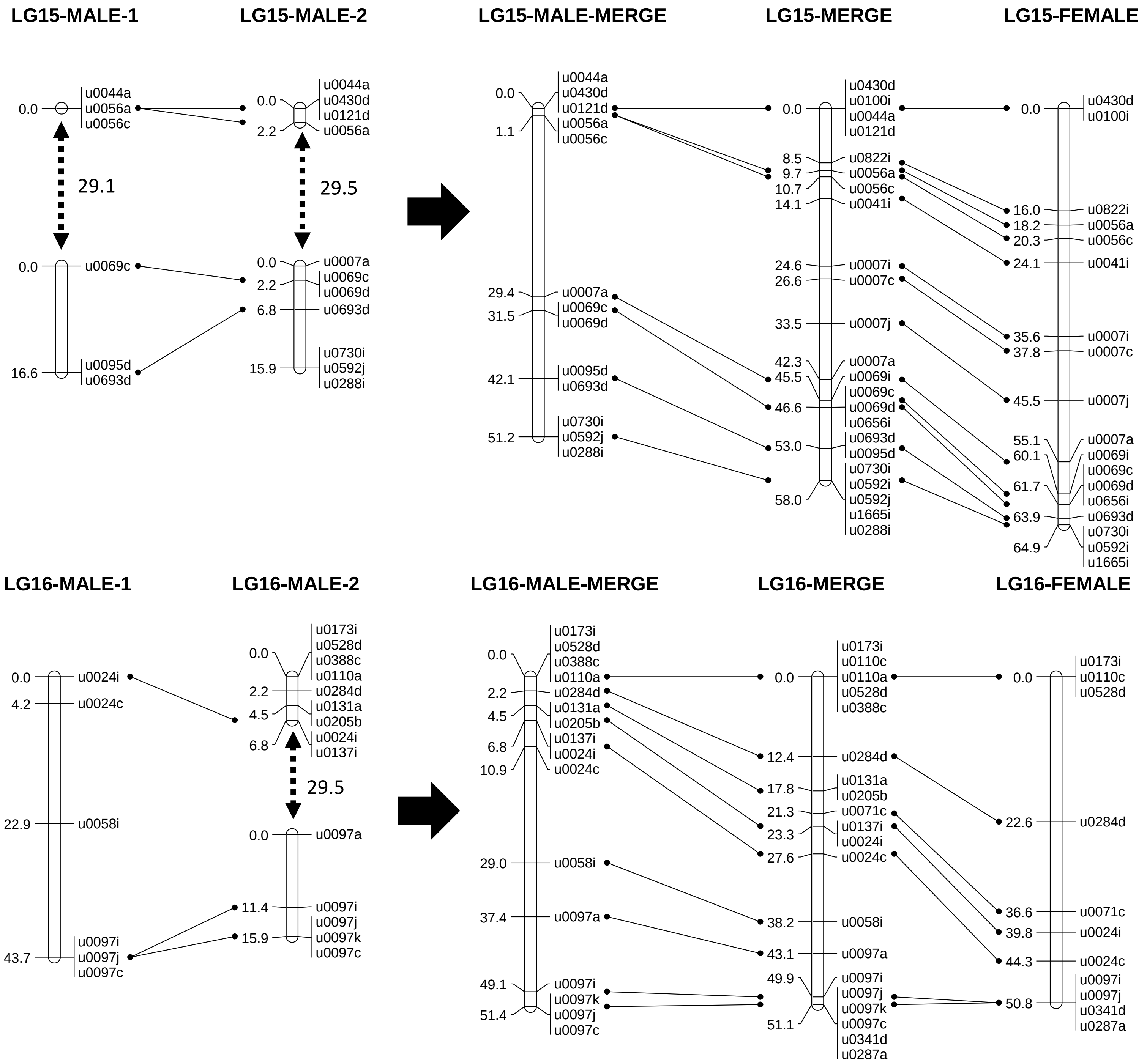

LG15-MALE-1
LG15-MALE-2
LG15-MALE-MERGE
LG15-MERGE
LG15-FEMALE
29.1
29.5
LG16-MALE-1
LG16-MALE-2
LG16-MALE-MERGE
LG16-MERGE
LG16-FEMALE
29.5

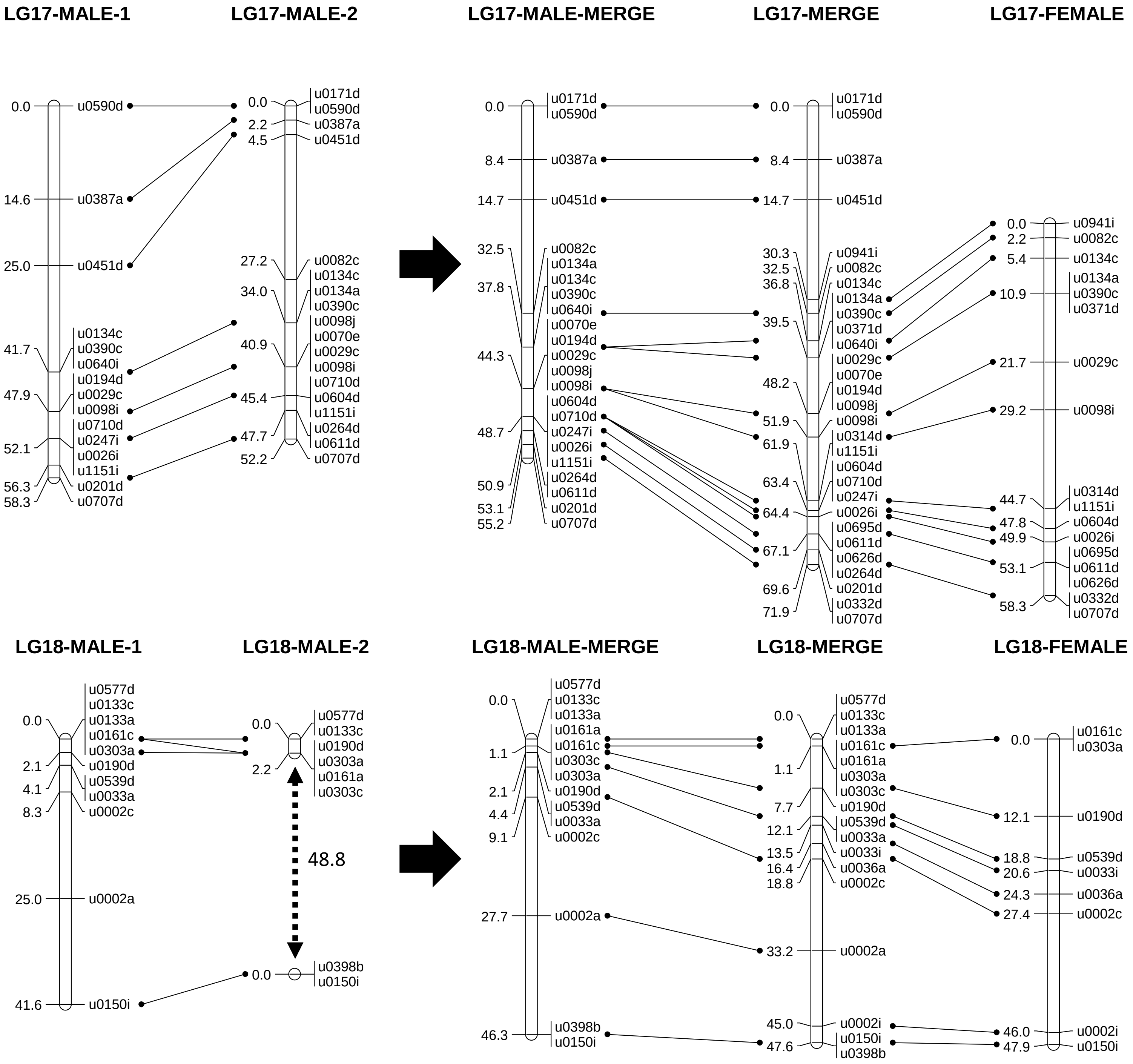

LG17-MALE-1
0.0 u0590d
14.6 u0387a
25.0 u0451d
41.7 u0134c u0390c u0640i
47.9 u0194d u0029c u0098i
52.1 u0710d u0247i u0026i
56.3 u1151i u0201d
58.3 u0707d
LG17-MALE-2
0.0 u0171d u0590d
2.2 u0387a
4.5 u0451d
27.2 u0082c u0134c
34.0 u0134a u0390c u0098j
40.9 u0070e u0029c u0098i
45.4 u0710d u0604d u1151i
47.7 u0264d u0611d
52.2 u0707d
LG17-MALE-MERGE
0.0 u0171d u0590d
8.4 u0387a
14.7 u0451d
32.5 u0082c u0134a
37.8 u0134c u0390c u0640i
44.3 u0070e u0194d u0029c u0098j u0098i
48.7 u0604d u0710d u0247i u0026i u1151i
50.9 u0264d
53.1 u0611d u0201d
55.2 u0707d
LG17-MERGE
0.0 u0171d u0590d
8.4 u0387a
14.7 u0451d
30.3 u0941i
32.5 u0082c
36.8 u0134c u0134a u0390c
39.5 u0371d u0640i u0029c
48.2 u0070e u0194d u0098j
51.9 u0098i u0314d
61.9 u1151i u0604d
63.4 u0710d u0247i
64.4 u0026i u0695d
67.1 u0611d u0626d u0264d
69.6 u0201d u0332d
71.9 u0707d
LG17-FEMALE
0.0 u0941i
2.2 u0082c
5.4 u0134c
10.9 u0134a u0390c u0371d
21.7 u0029c
29.2 u0098i
44.7 u0314d u1151i
47.8 u0604d
49.9 u0026i
53.1 u0695d u0611d u0626d
58.3 u0332d u0707d
LG18-MALE-1
0.0 u0577d u0133c u0133a u0161c u0303a
2.1 u0190d
4.1 u0539d u0033a
8.3 u0002c
25.0 u0002a
41.6 u0150i
LG18-MALE-2
0.0 u0577d u0133c
2.2 u0190d u0303a u0161a u0303c
48.8
0.0 u0398b u0150i
LG18-MALE-MERGE
0.0 u0577d u0133c u0133a u0161a
1.1 u0161c u0303c u0303a
2.1 u0190d
4.4 u0539d u0033a
9.1 u0002c
27.7 u0002a
46.3 u0398b u0150i
LG18-MERGE
0.0 u0577d u0133c u0133a u0161c
1.1 u0161a u0303a u0303c
7.7 u0190d
12.1 u0539d u0033a
13.5 u0033i
16.4 u0036a
18.8 u0002c
33.2 u0002a
45.0 u0002i
47.6 u0150i u0398b
LG18-FEMALE
0.0 u0161c u0303a
12.1 u0190d
18.8 u0539d
20.6 u0033i
24.3 u0036a
27.4 u0002c
46.0 u0002i
47.9 u0150i

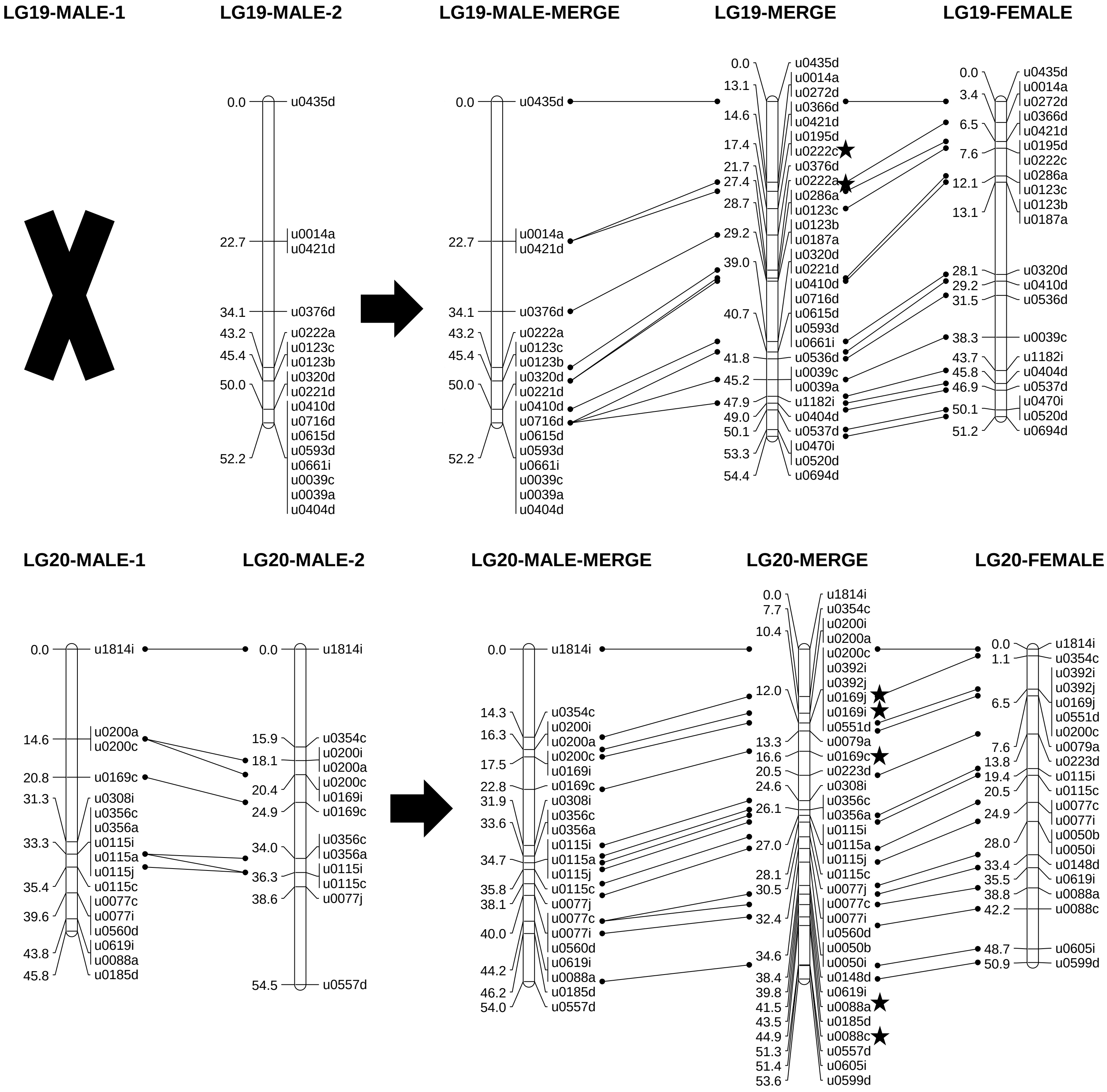

LG19-MALE-1
LG19-MALE-2
0.0 u0435d
22.7 u0014a u0421d
34.1 u0376d
43.2 u0222a
45.4 u0123c u0123b
50.0 u0320d u0221d
52.2 u0410d u0716d u0615d u0593d u0661i u0039c u0039a u0404d
LG19-MALE-MERGE
0.0 u0435d
22.7 u0014a u0421d
34.1 u0376d
43.2 u0222a
45.4 u0123c u0123b
50.0 u0320d u0221d
52.2 u0410d u0716d u0615d u0593d u0661i u0039c u0039a u0404d
LG19-MERGE
0.0 u0435d
13.1 u0014a u0272d
14.6 u0366d u0421d
17.4 u0195d u0222c
21.7 u0376d
27.4 u0222a
28.7 u0286a u0123c
29.2 u0123b u0187a
39.0 u0320d u0221d
40.7 u0410d u0716d u0615d u0593d u0661i
41.8 u0536d
45.2 u0039c u0039a
47.9 u1182i
49.0 u0404d
50.1 u0537d
53.3 u0470i u0520d
54.4 u0694d
LG19-FEMALE
0.0 u0435d
3.4 u0014a u0272d
6.5 u0366d u0421d
7.6 u0195d u0222c
12.1 u0286a u0123c
13.1 u0123b u0187a
28.1 u0320d
29.2 u0410d
31.5 u0536d
38.3 u0039c
43.7 u1182i
45.8 u0404d
46.9 u0537d
50.1 u0470i u0520d
51.2 u0694d
LG20-MALE-1
0.0 u1814i
14.6 u0200a u0200c
20.8 u0169c
31.3 u0308i u0356c u0356a
33.3 u0115i u0115a u0115j
35.4 u0115c
39.6 u0077c u0077i u0560d
43.8 u0619i u0088a
45.8 u0185d
LG20-MALE-2
0.0 u1814i
15.9 u0354c
18.1 u0200i u0200a
20.4 u0200c u0169i
24.9 u0169c
34.0 u0356c u0356a
36.3 u0115i u0115c
38.6 u0077j
54.5 u0557d
LG20-MALE-MERGE
0.0 u1814i
14.3 u0354c
16.3 u0200i u0200a
17.5 u0200c u0169i
22.8 u0169c
31.9 u0308i
33.6 u0356c u0356a
34.7 u0115i u0115a u0115j
35.8 u0115c
38.1 u0077j
40.0 u0077c u0077i u0560d
44.2 u0619i u0088a
46.2 u0185d
54.0 u0557d
LG20-MERGE
0.0 u1814i
7.7 u0354c
10.4 u0200i u0200a u0200c u0392i u0392j
12.0 u0169j u0169i u0551d
13.3 u0079a
16.6 u0169c
20.5 u0223d
24.6 u0308i
26.1 u0356c u0356a u0115i
27.0 u0115a u0115j u0115c
28.1 u0077j
30.5 u0077c
32.4 u0077i u0560d
34.6 u0050b u0050i
38.4 u0148d
39.8 u0619i
41.5 u0088a
43.5 u0185d
44.9 u0088c
51.3 u0557d
51.4 u0605i
53.6 u0599d
LG20-FEMALE
0.0 u1814i
1.1 u0354c
6.5 u0392i u0392j u0169j u0551d u0200c
7.6 u0079a
13.8 u0223d
19.4 u0115i
20.5 u0115c
24.9 u0077c u0077i
28.0 u0050b u0050i
33.4 u0148d
35.5 u0619i
38.8 u0088a
42.2 u0088c
48.7 u0605i
50.9 u0599d

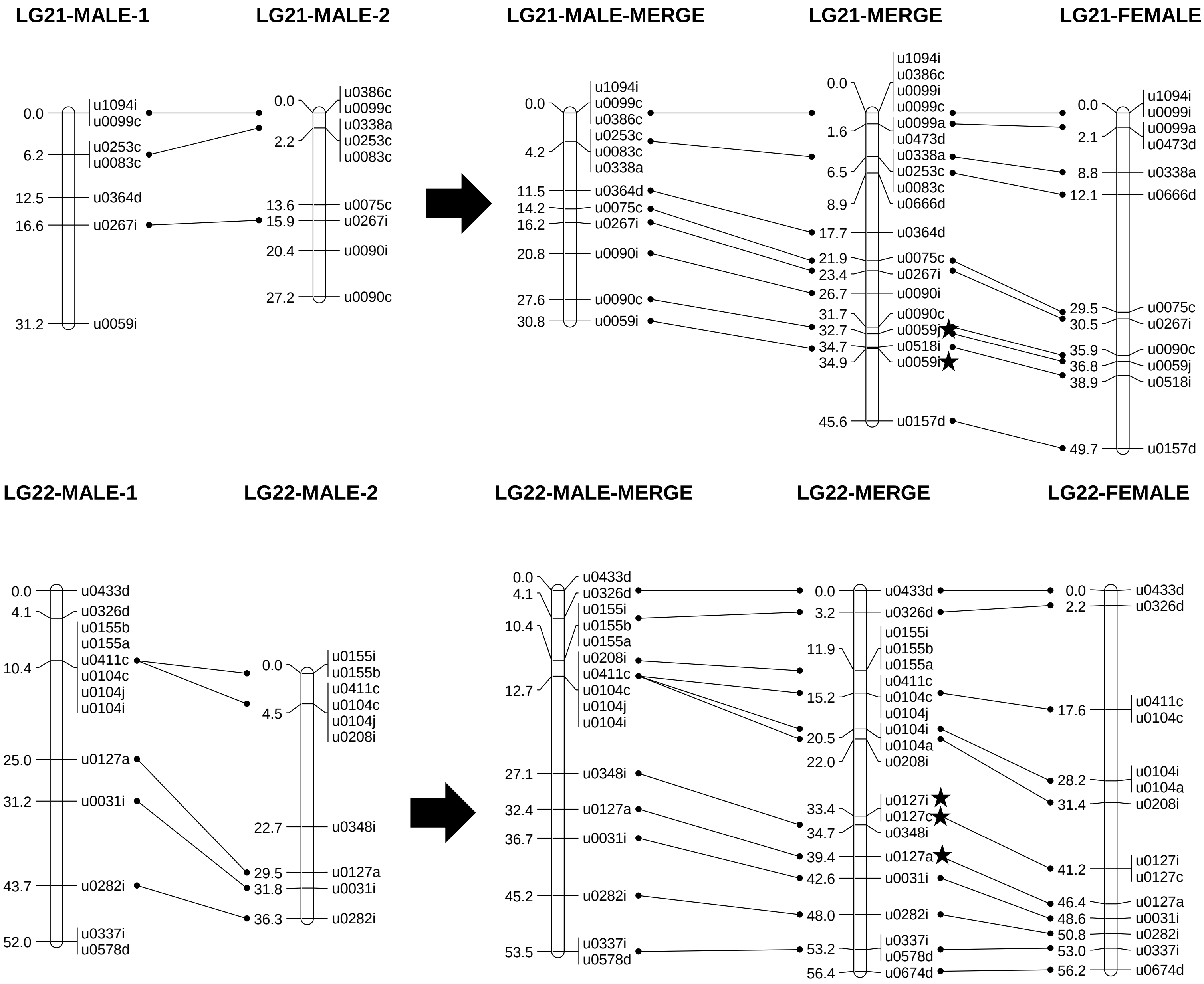
LG21-MALE-1
0.0 u1094i u0099c
6.2 u0253c u0083c
12.5 u0364d
16.6 u0267i
31.2 u0059i
LG21-MALE-2
0.0 u0386c u0099c
2.2 u0338a u0253c u0083c
13.6 u0075c
15.9 u0267i
20.4 u0090i
27.2 u0090c
LG21-MALE-MERGE
0.0 u1094i u0099c u0386c
4.2 u0253c u0083c u0338a
11.5 u0364d
14.2 u0075c
16.2 u0267i
20.8 u0090i
27.6 u0090c
30.8 u0059i
LG21-MERGE
0.0 u1094i u0386c u0099i u0099c
1.6 u0099a u0473d
6.5 u0338a u0253c u0083c
8.9 u0666d
17.7 u0364d
21.9 u0075c
23.4 u0267i
26.7 u0090i
31.7 u0090c
32.7 u0059j
34.7 u0518i
34.9 u0059i
45.6 u0157d
LG21-FEMALE
0.0 u1094i u0099i
2.1 u0099a u0473d
8.8 u0338a
12.1 u0666d
29.5 u0075c
30.5 u0267i
35.9 u0090c
36.8 u0059j
38.9 u0518i
49.7 u0157d
LG22-MALE-1
0.0 u0433d
4.1 u0326d
10.4 u0155b u0155a u0411c u0104c u0104j u0104i
25.0 u0127a
31.2 u0031i
43.7 u0282i
52.0 u0337i u0578d
LG22-MALE-2
0.0 u0155i u0155b
4.5 u0411c u0104c u0104j u0208i
22.7 u0348i
29.5 u0127a
31.8 u0031i
36.3 u0282i
LG22-MALE-MERGE
0.0 u0433d
4.1 u0326d
10.4 u0155i u0155b u0155a
12.7 u0208i u0411c u0104c u0104j u0104i
27.1 u0348i
32.4 u0127a
36.7 u0031i
45.2 u0282i
53.5 u0337i u0578d
LG22-MERGE
0.0 u0433d
3.2 u0326d
11.9 u0155i u0155b u0155a
15.2 u0411c u0104c u0104j
20.5 u0104i u0104a
22.0 u0208i
33.4 u0127i u0127c
34.7 u0348i
39.4 u0127a
42.6 u0031i
48.0 u0282i
53.2 u0337i u0578d
56.4 u0674d
LG22-FEMALE
0.0 u0433d
2.2 u0326d
17.6 u0411c u0104c
28.2 u0104i u0104a
31.4 u0208i
41.2 u0127i u0127c
46.4 u0127a
48.6 u0031i
50.8 u0282i
53.0 u0337i
56.2 u0674d

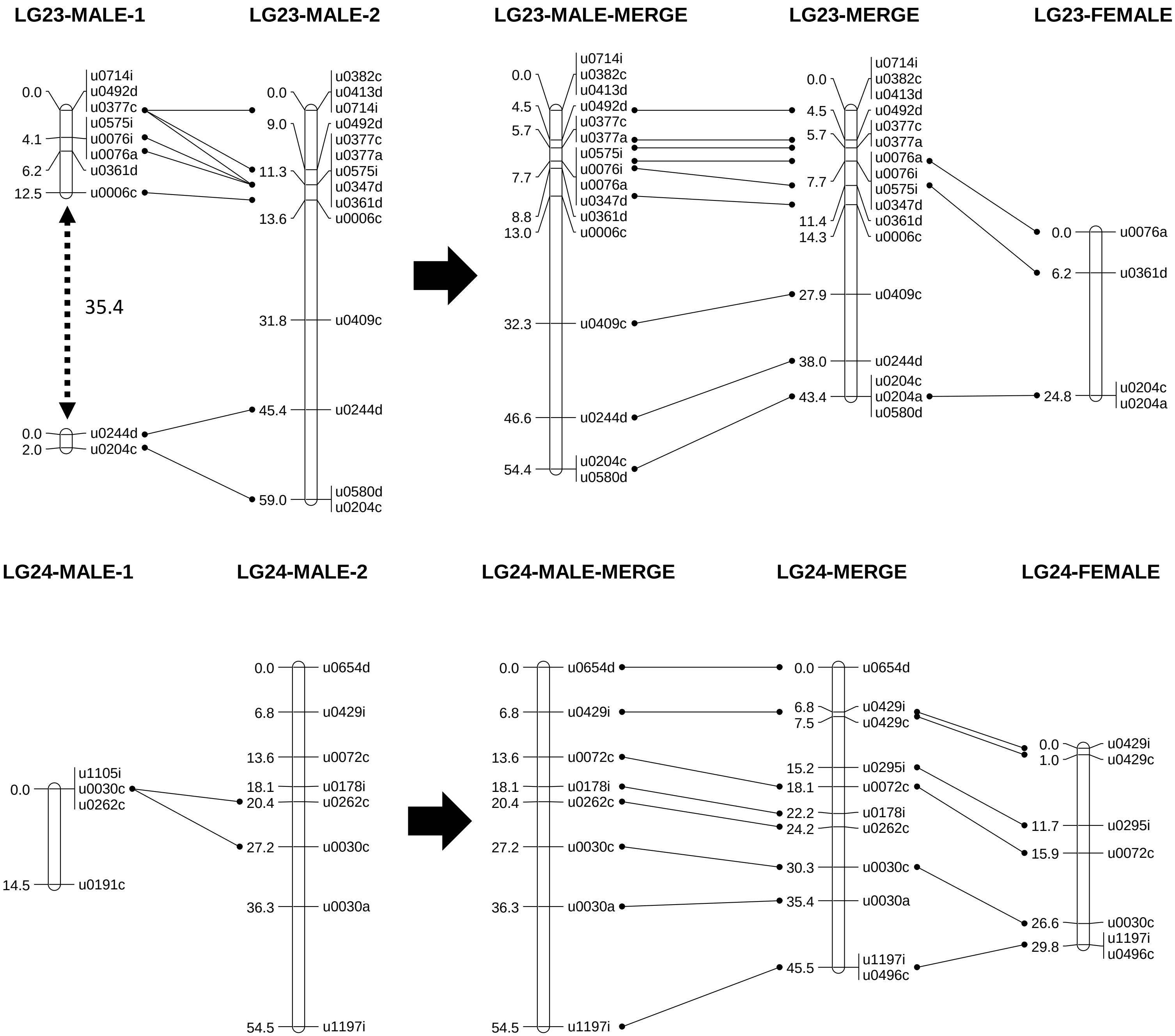

LG23-MALE-1
0.0 u0714i u0492d u0377c
4.1 u0575i u0076i
6.2 u0076a u0361d
12.5 u0006c
35.4
0.0 u0244d
2.0 u0204c
LG23-MALE-2
0.0 u0382c u0413d u0714i
9.0 u0492d u0377c u0377a
11.3 u0575i u0347d
13.6 u0361d u0006c
31.8 u0409c
45.4 u0244d
59.0 u0580d u0204c
LG23-MALE-MERGE
0.0 u0714i u0382c u0413d
4.5 u0492d
5.7 u0377c u0377a
7.7 u0575i u0076i u0076a
8.8 u0347d
13.0 u0361d u0006c
32.3 u0409c
46.6 u0244d
54.4 u0204c u0580d
LG23-MERGE
0.0 u0714i u0382c u0413d
4.5 u0492d
5.7 u0377c u0377a
7.7 u0076a u0076i u0575i u0347d
11.4 u0361d
14.3 u0006c
27.9 u0409c
38.0 u0244d
43.4 u0204c u0204a u0580d
LG23-FEMALE
0.0 u0076a
6.2 u0361d
24.8 u0204c u0204a
LG24-MALE-1
0.0 u1105i u0030c u0262c
14.5 u0191c
LG24-MALE-2
0.0 u0654d
6.8 u0429i
13.6 u0072c
18.1 u0178i
20.4 u0262c
27.2 u0030c
36.3 u0030a
54.5 u1197i
LG24-MALE-MERGE
0.0 u0654d
6.8 u0429i
13.6 u0072c
18.1 u0178i
20.4 u0262c
27.2 u0030c
36.3 u0030a
54.5 u1197i
LG24-MERGE
0.0 u0654d
6.8 u0429i
7.5 u0429c
15.2 u0295i
18.1 u0072c
22.2 u0178i
24.2 u0262c
30.3 u0030c
35.4 u0030a
45.5 u1197i u0496c
LG24-FEMALE
0.0 u0429i
1.0 u0429c
11.7 u0295i
15.9 u0072c
26.6 u0030c
29.8 u1197i u0496c

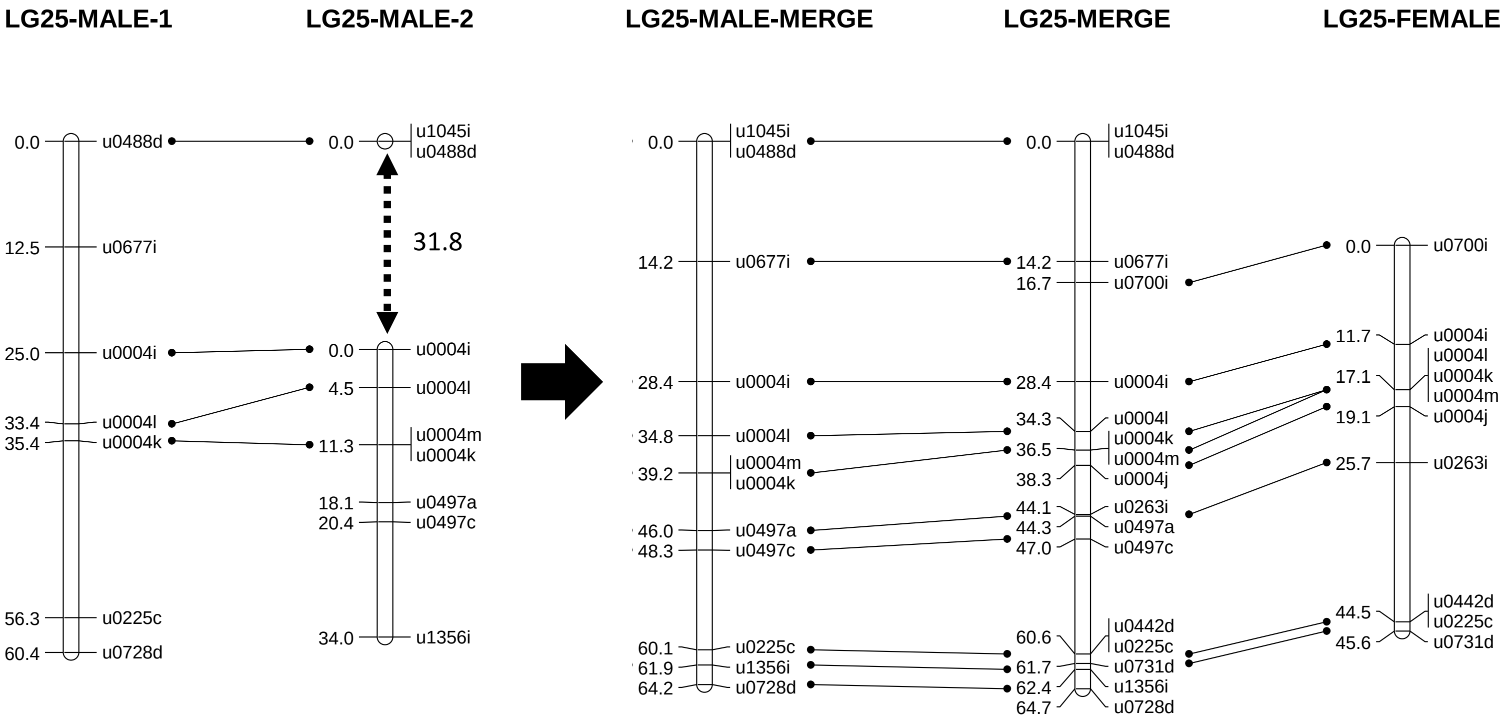

Denotes the estimated distance (Below LOD 3.0) in Centimorgans. Used to create Merged Maps.

★ Denotes Inconsistent placement of scaffolds in merged map.
