# Supplementary material for: The Genome and Linkage Map of the Northern Pike (Esox lucius): Conserved Synteny Revealed between the Salmonid Sister Group and the Neoteleostei
Source: PLoS One. 2014 Jul 28;9(7):e102089. doi: 10.1371/journal.pone.0102089 (PMC4113312; doi:10.1371/journal.pone.0102089)
Supplement: Figure S2 — Conservation of gene order within the largest 50 northern pike genomic scaffolds relative to Gasterosteus aculeatus , Oryzias latipes , and Danio rerio . Reciprocal best hit results (≥50% of total length, ≤1e-5) and mapped scaffold position plotted. Average position ((Start position + End position)/2) taken to represent a point position for each mapped transcript. (PDF) [file pone.0102089.s002.pdf]

PLOT OF TRANSCRIPT ORDER IN SCAFFOLD VERSUS MODEL GENOMES

- Includes largest 50 scaffolds
- 3 Genomes: Threespine Stickleback (*Gasterosteus aculeatus*), Japanese Medaka (*Oryzias latipes*), and Zebrafish (*Danio rerio*)
- Included when ≥2 Blast hits are to a given chromosome
- Each point is plotted at the midway point between the start and end of the transcript in both the Scaffold and the Chromosomes
- Each chromosome is given a different colour in the plot, and chromosomes are overlayed on the same graph
- Raw Blast results used to construct figures can be found in the second tab of Supplementary file 5

Scaffold 1

Scaffold 1 - *G. aculeatus* v. *E. lucius*

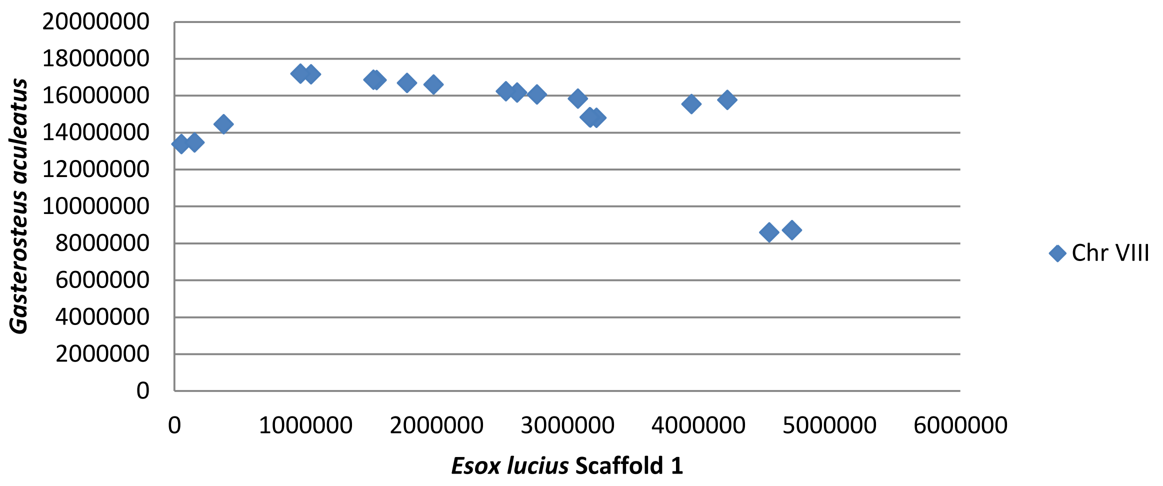

Scaffold 1 - *O. latipes* v. *E. lucius*

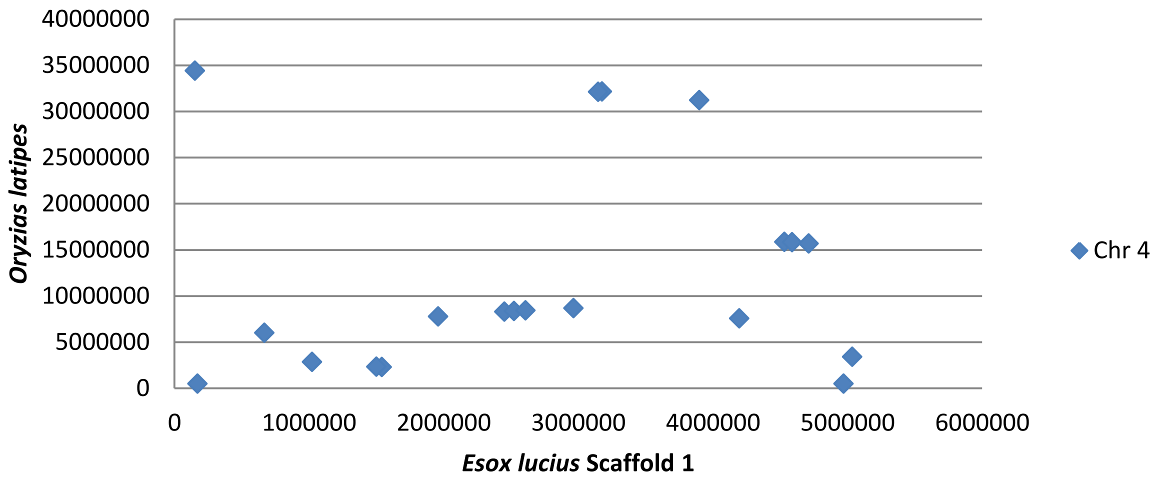

Scaffold 1 - *D. rerio* v. *E. lucius*

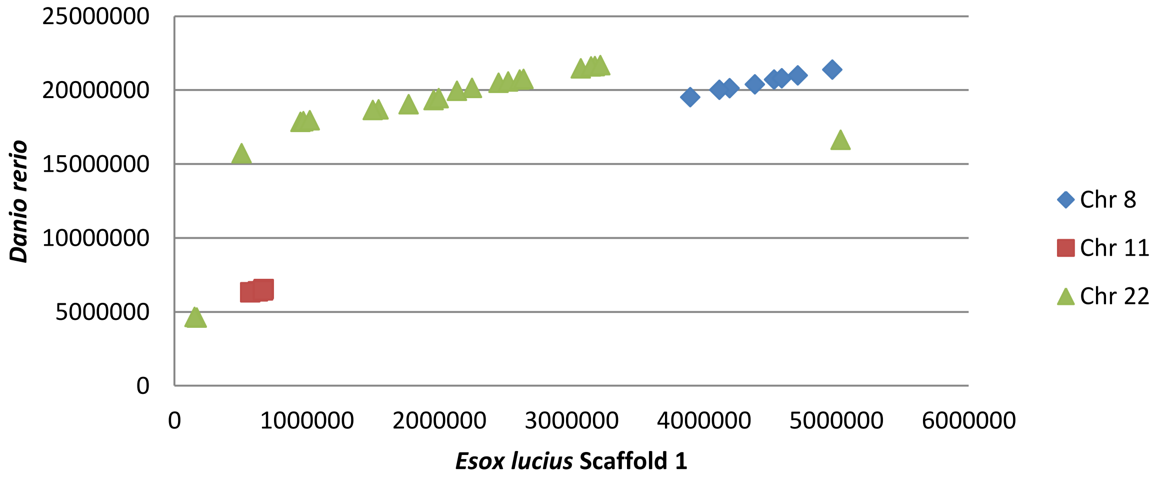

Scaffold 2

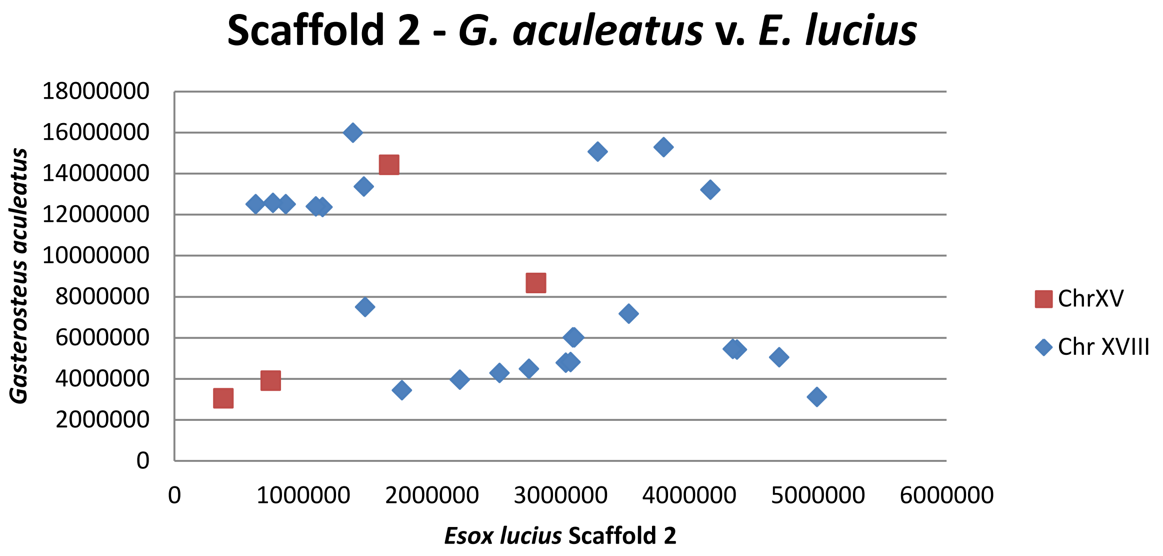
Scaffold 2 - G. aculeatus v. E. lucius
18000000
16000000
14000000
12000000
10000000
8000000
6000000
4000000
2000000
0
Gasterosteus aculeatus
0
1000000
2000000
3000000
4000000
5000000
6000000
Esox lucius Scaffold 2
ChrXV
Chr XVIII

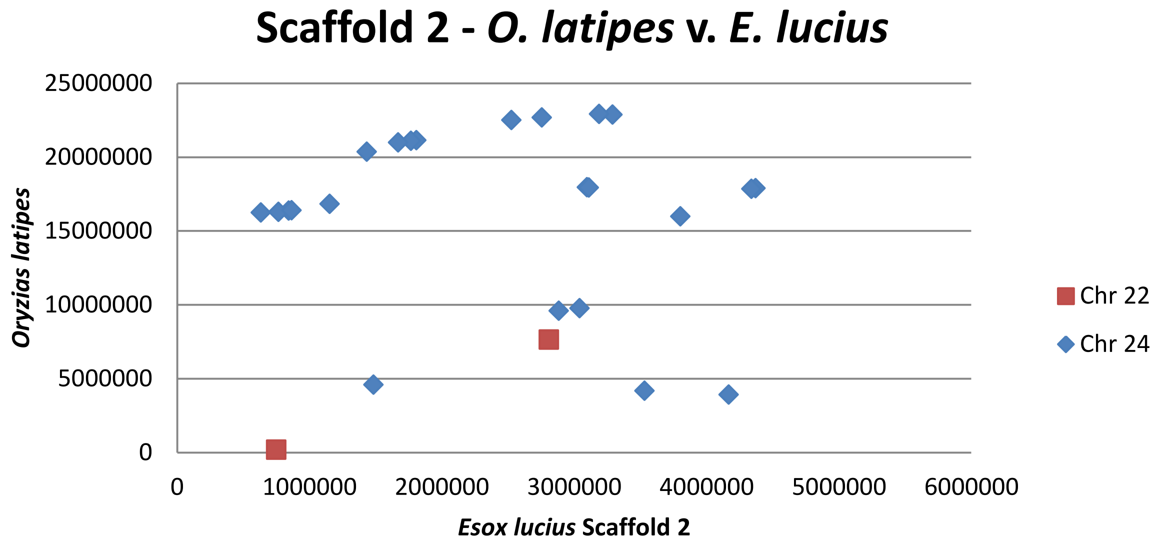
Scaffold 2 - O. latipes v. E. lucius
25000000
20000000
15000000
10000000
5000000
0
Oryzias latipes
0
1000000
2000000
3000000
4000000
5000000
6000000
Esox lucius Scaffold 2
Chr 22
Chr 24

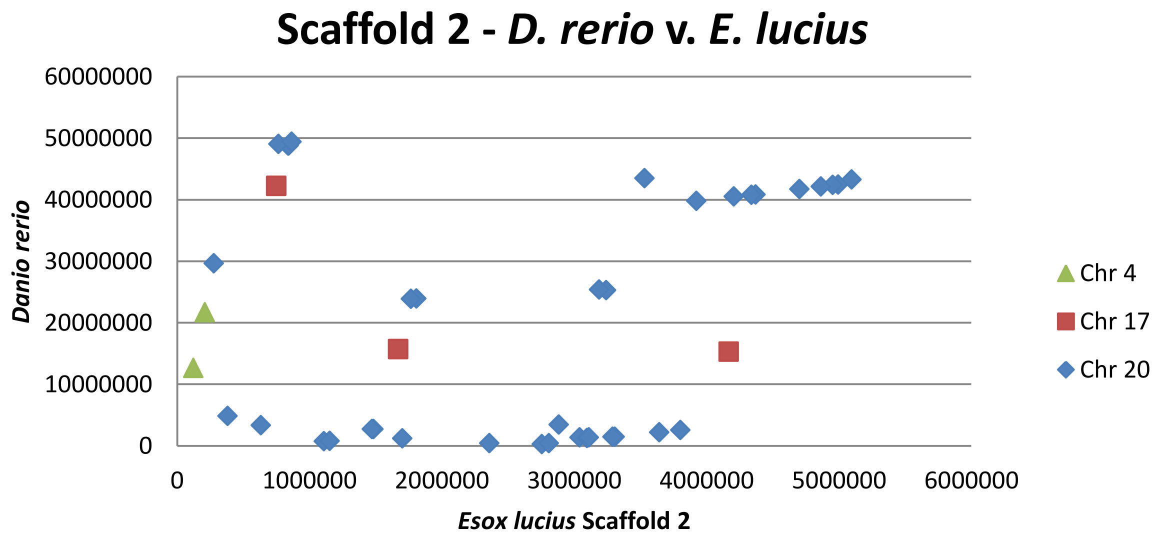
Scaffold 2 - D. rerio v. E. lucius
60000000
50000000
40000000
30000000
20000000
10000000
0
Danio rerio
0
1000000
2000000
3000000
4000000
5000000
6000000
Esox lucius Scaffold 2
Chr 4
Chr 17
Chr 20

Scaffold 3

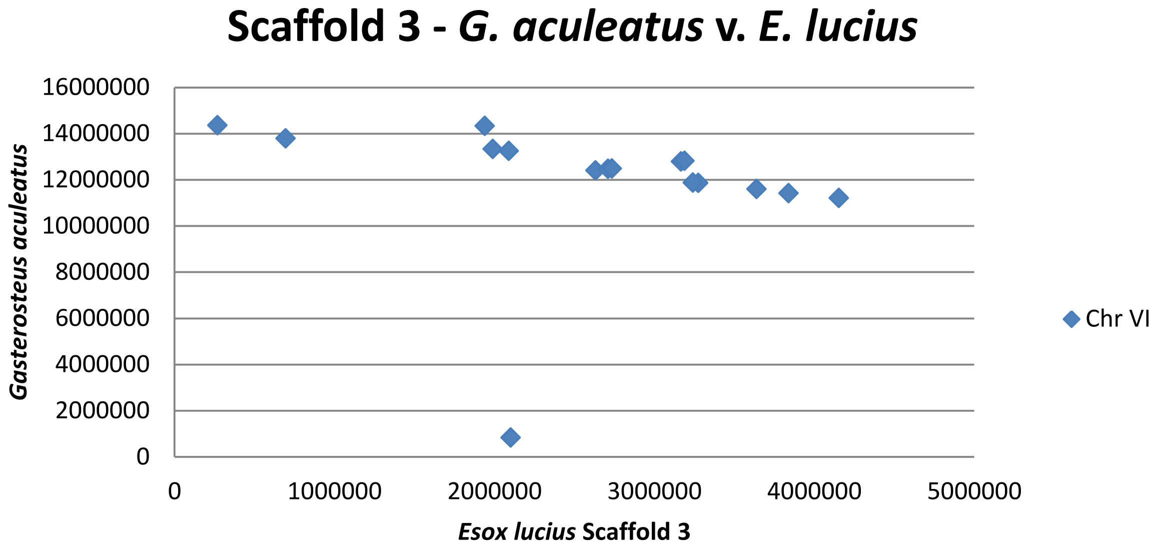
Scaffold 3 - G. aculeatus v. E. lucius
Gasterosteus aculeatus
16000000
14000000
12000000
10000000
8000000
6000000
4000000
2000000
0
0
1000000
2000000
3000000
4000000
5000000
Esox lucius Scaffold 3
Chr VI

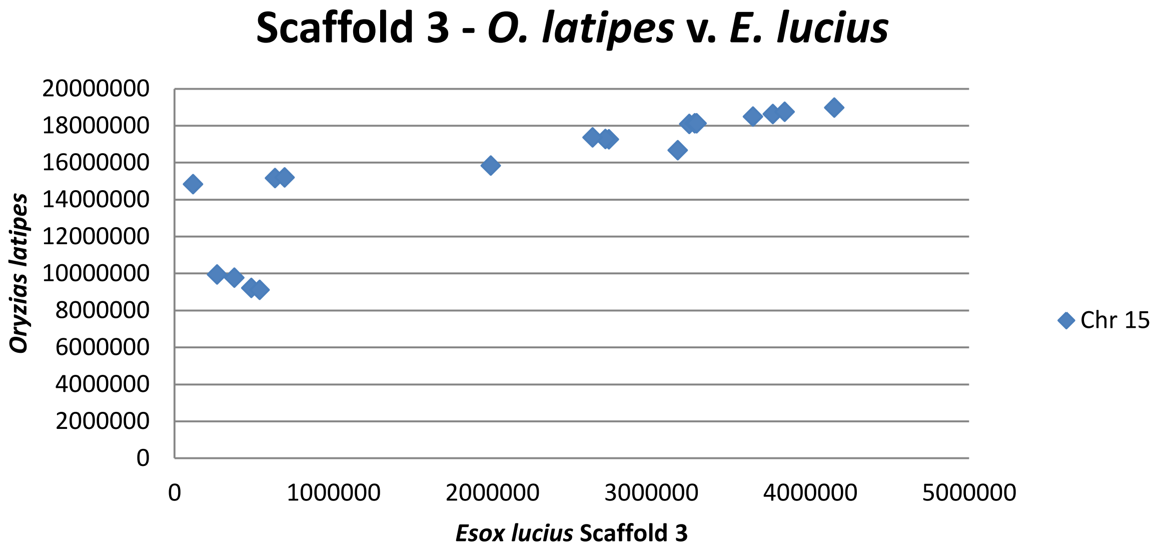
Scaffold 3 - O. latipes v. E. lucius
Oryzias latipes
20000000
18000000
16000000
14000000
12000000
10000000
8000000
6000000
4000000
2000000
0
0
1000000
2000000
3000000
4000000
5000000
Esox lucius Scaffold 3
Chr 15

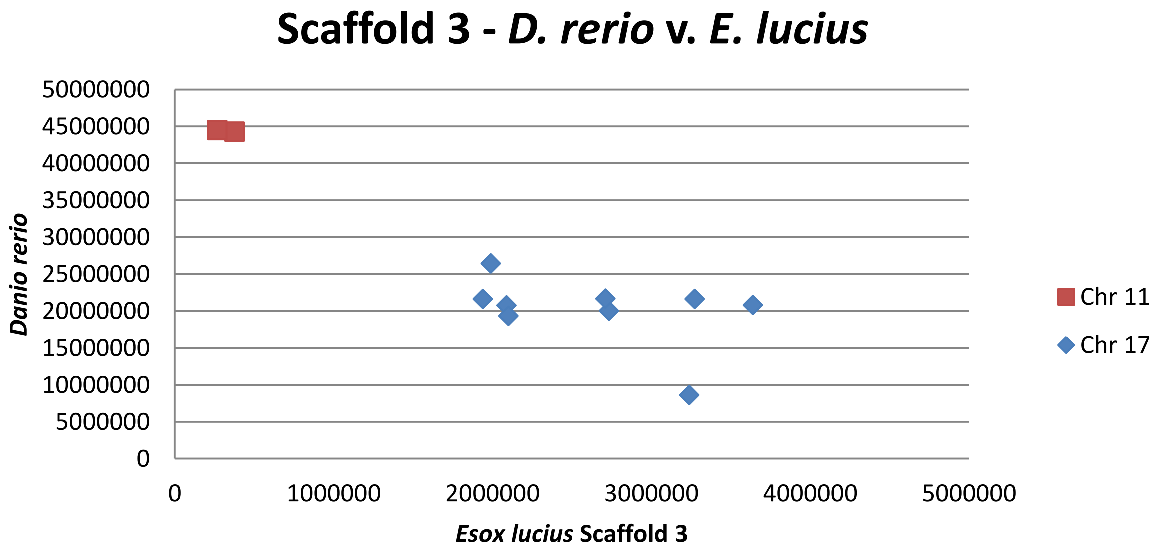
Scaffold 3 - D. rerio v. E. lucius
Danio rerio
50000000
45000000
40000000
35000000
30000000
25000000
20000000
15000000
10000000
5000000
0
0
1000000
2000000
3000000
4000000
5000000
Esox lucius Scaffold 3
Chr 11
Chr 17

Scaffold 4

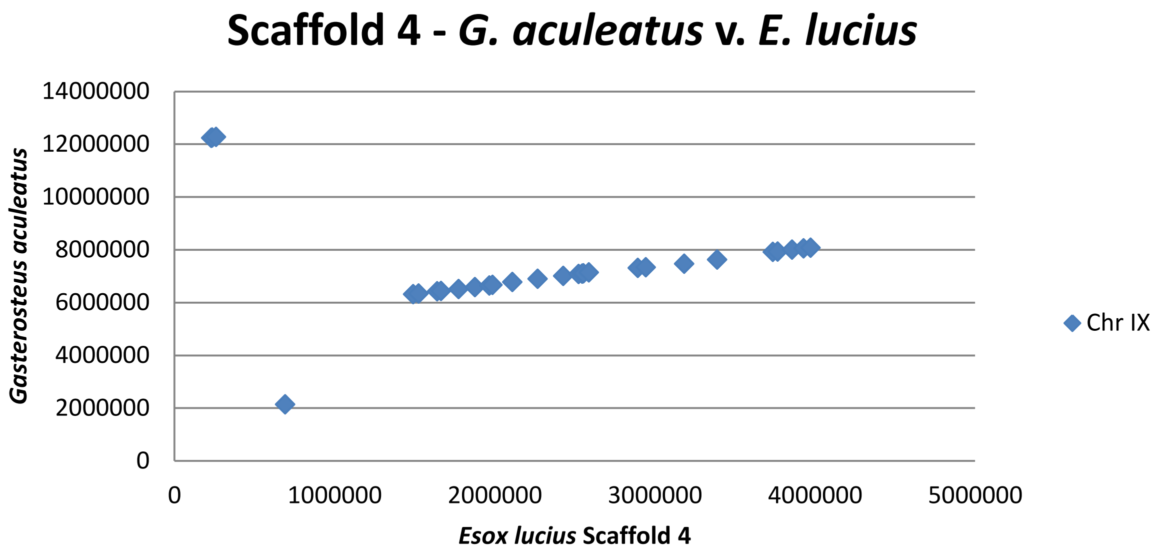
Scaffold 4 - G. aculeatus v. E. lucius
Gasterosteus aculeatus
14000000
12000000
10000000
8000000
6000000
4000000
2000000
0
1000000
2000000
3000000
4000000
5000000
Esox lucius Scaffold 4
Chr IX

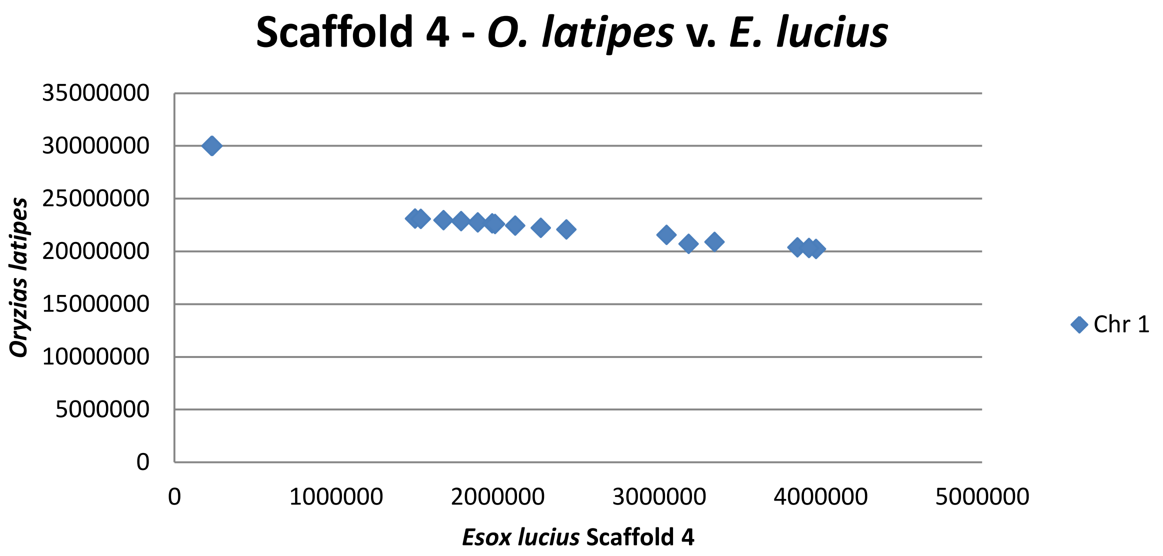
Scaffold 4 - O. latipes v. E. lucius
Oryzias latipes
35000000
30000000
25000000
20000000
15000000
10000000
5000000
0
1000000
2000000
3000000
4000000
5000000
Esox lucius Scaffold 4
Chr 1

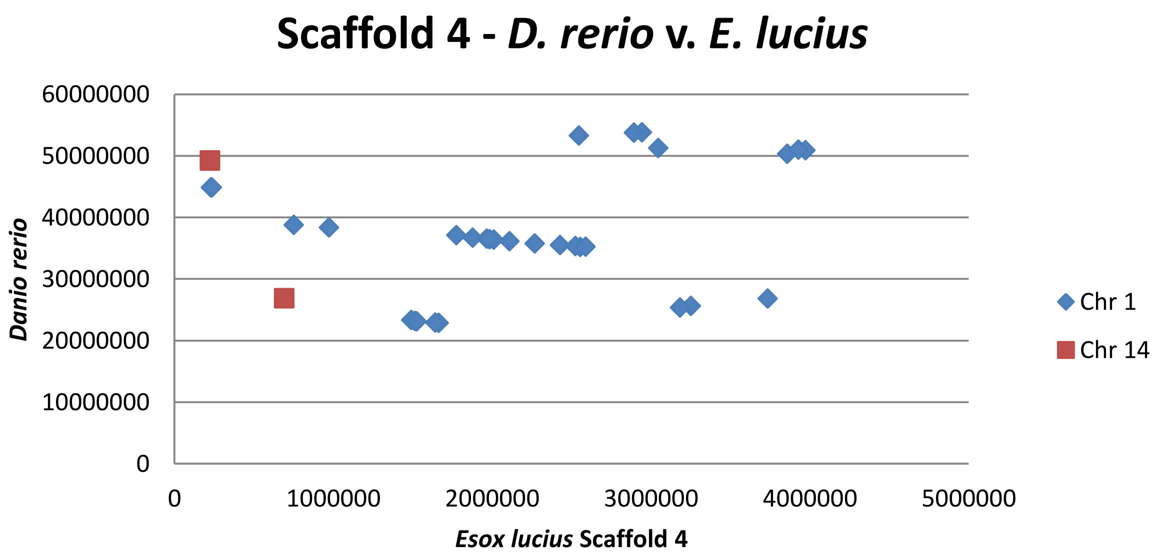
Scaffold 4 - D. rerio v. E. lucius
Danio rerio
60000000
50000000
40000000
30000000
20000000
10000000
0
1000000
2000000
3000000
4000000
5000000
Esox lucius Scaffold 4
Chr 1
Chr 14

Scaffold 5

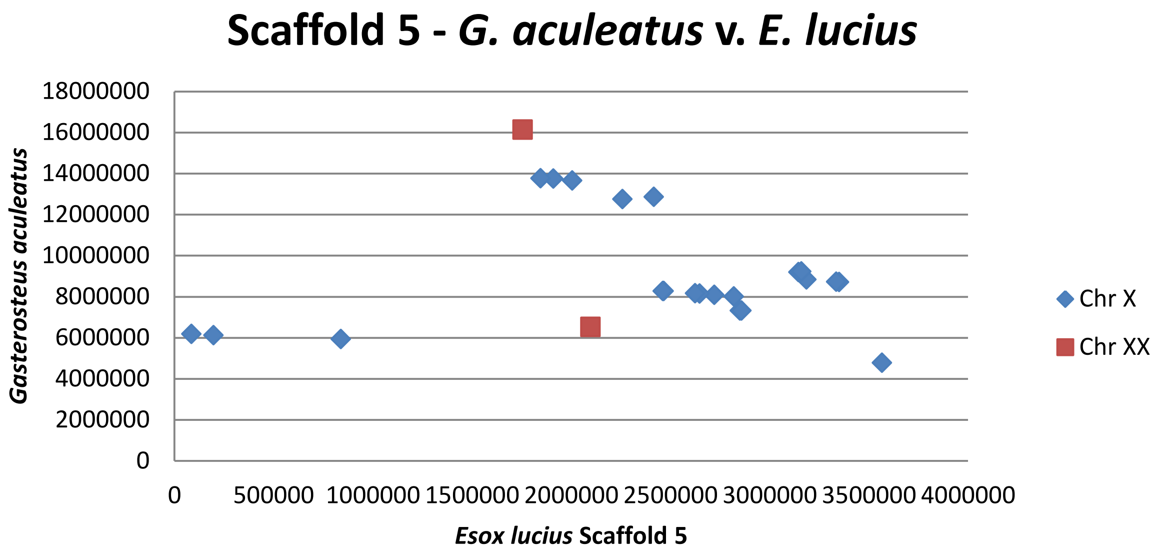
Scaffold 5 - *G. aculeatus* v. *E. lucius*
18000000
16000000
14000000
12000000
10000000
8000000
6000000
4000000
2000000
0
Gasterosteus aculeatus
0
500000
1000000
1500000
2000000
2500000
3000000
3500000
4000000
Esox lucius Scaffold 5
Chr X
Chr XX

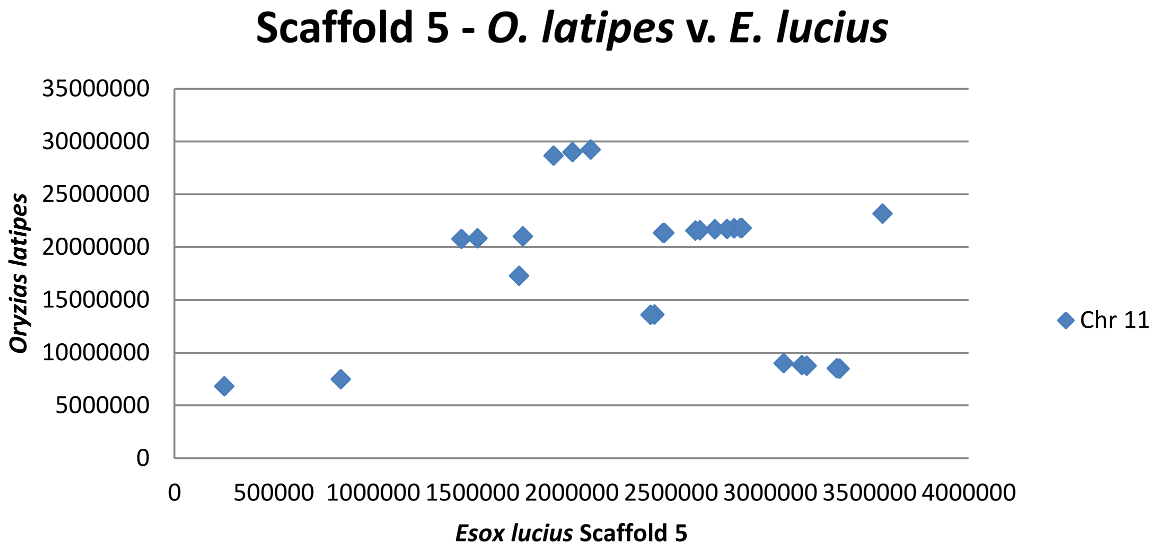
Scaffold 5 - *O. latipes* v. *E. lucius*
35000000
30000000
25000000
20000000
15000000
10000000
5000000
0
Oryzias latipes
0
500000
1000000
1500000
2000000
2500000
3000000
3500000
4000000
Esox lucius Scaffold 5
Chr 11

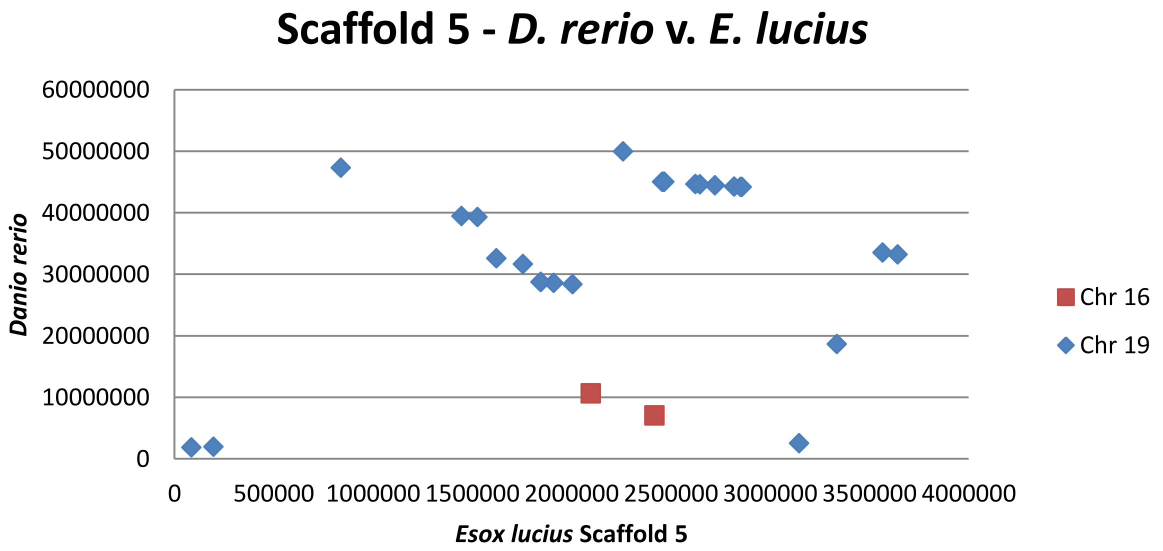
Scaffold 5 - *D. rerio* v. *E. lucius*
60000000
50000000
40000000
30000000
20000000
10000000
0
Danio rerio
0
500000
1000000
1500000
2000000
2500000
3000000
3500000
4000000
Esox lucius Scaffold 5
Chr 16
Chr 19

Scaffold 6

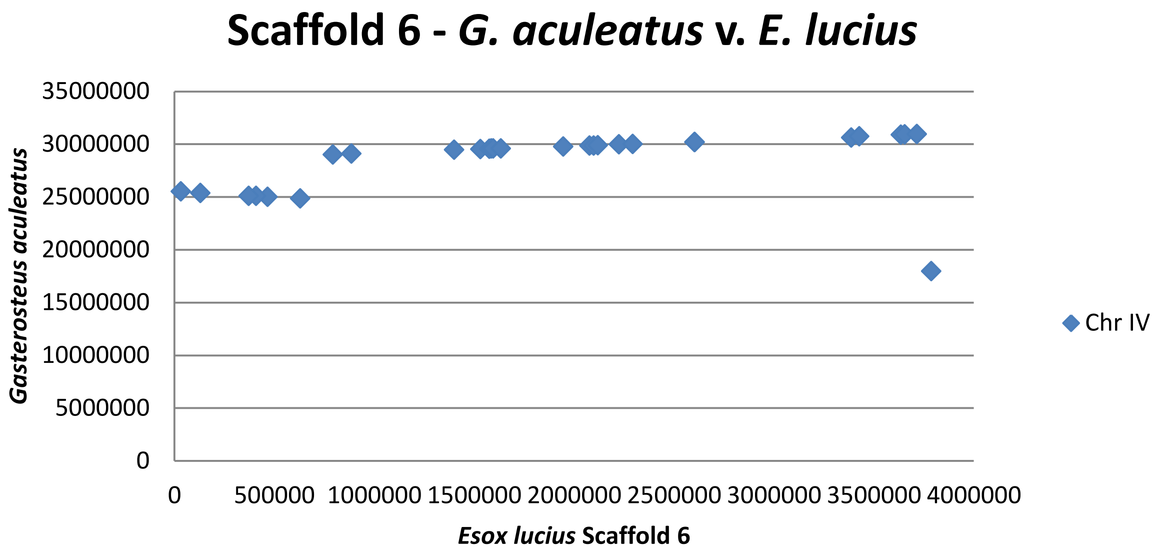
Scaffold 6 - G. aculeatus v. E. lucius
Gasterosteus aculeatus
35000000
30000000
25000000
20000000
15000000
10000000
5000000
0
0 500000 1000000 1500000 2000000 2500000 3000000 3500000 4000000
Esox lucius Scaffold 6
Chr IV

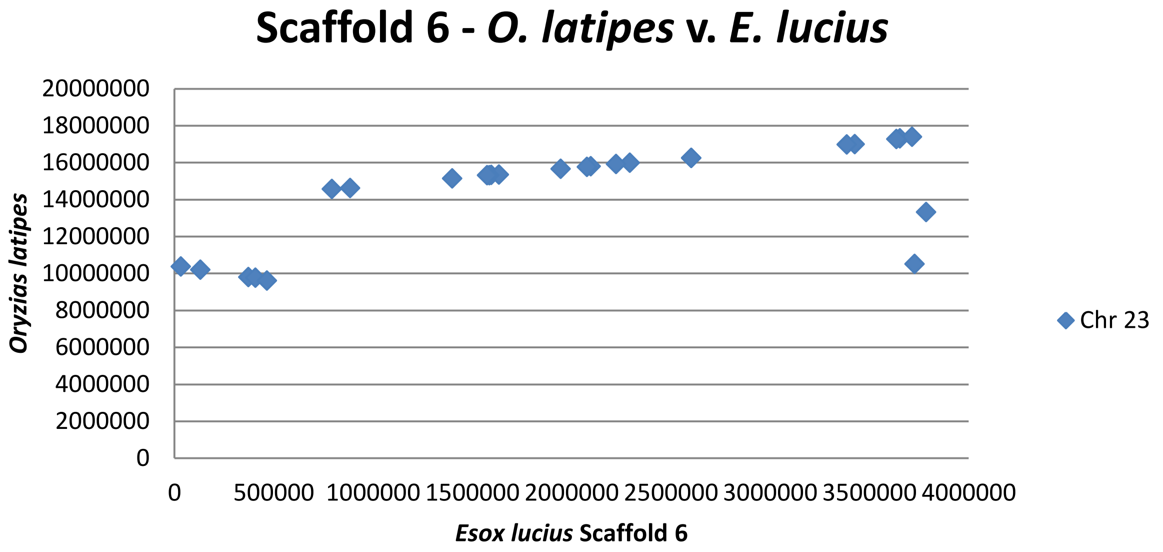
Scaffold 6 - O. latipes v. E. lucius
Oryzias latipes
20000000
18000000
16000000
14000000
12000000
10000000
8000000
6000000
4000000
2000000
0
0 500000 1000000 1500000 2000000 2500000 3000000 3500000 4000000
Esox lucius Scaffold 6
Chr 23

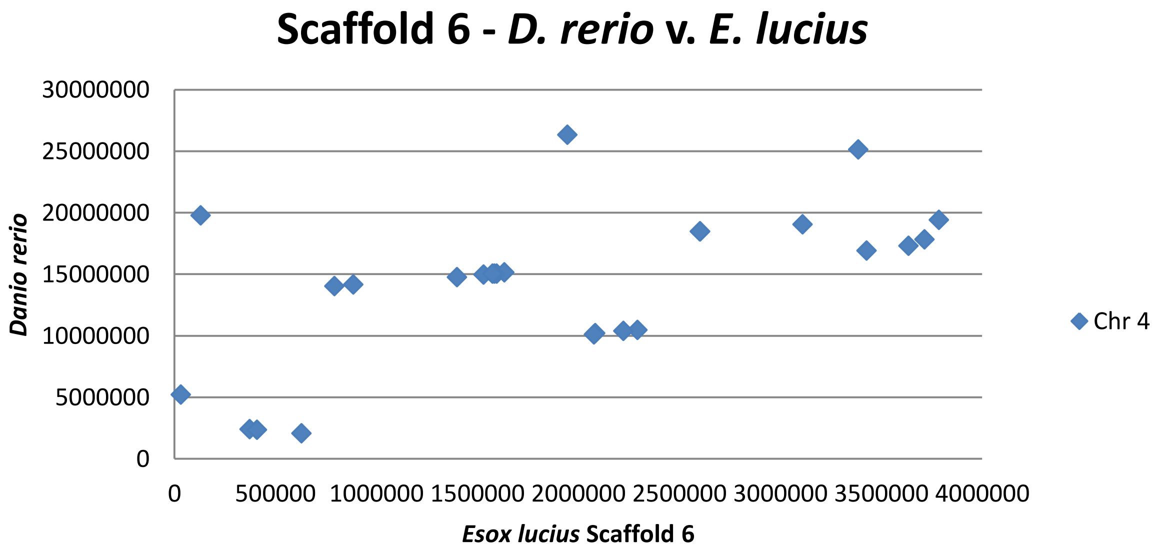
Scaffold 6 - D. rerio v. E. lucius
Danio rerio
30000000
25000000
20000000
15000000
10000000
5000000
0
0 500000 1000000 1500000 2000000 2500000 3000000 3500000 4000000
Esox lucius Scaffold 6
Chr 4

Scaffold 7

Scaffold 7 - *G. aculeatus* v. *E. lucius*

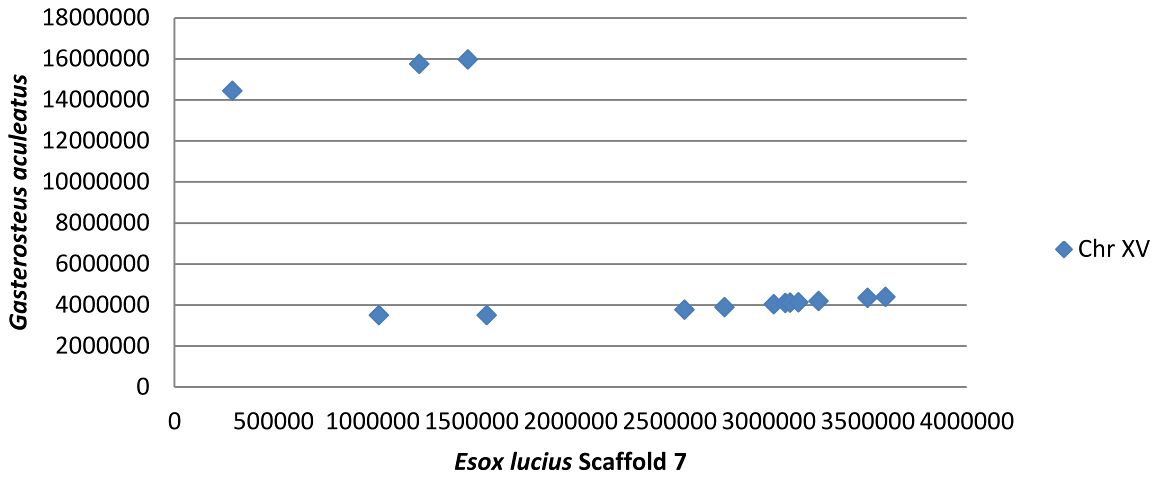

Scaffold 7 - *O. latipes* v. *E. lucius*

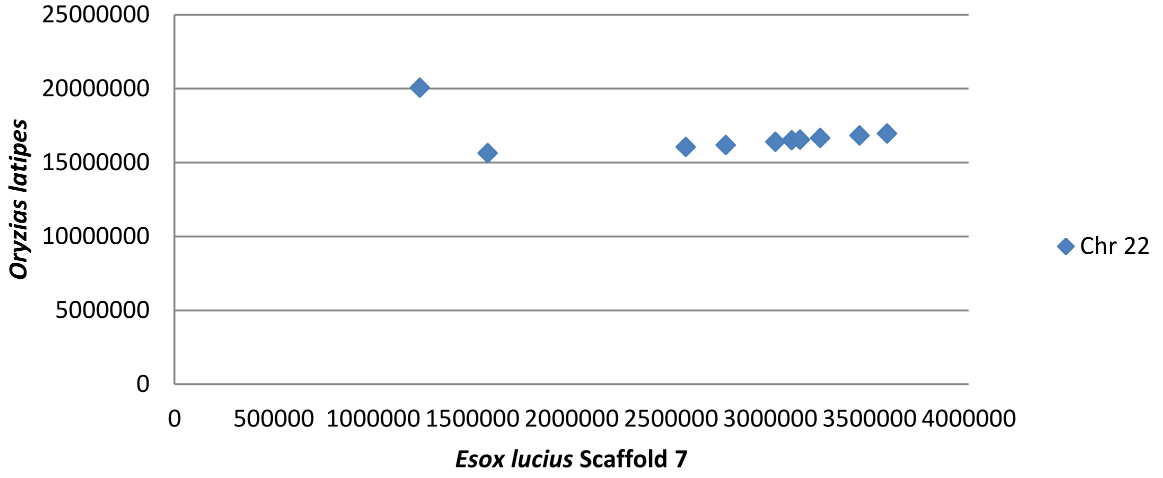

Scaffold 7 - *D. rerio* v. *E. lucius*

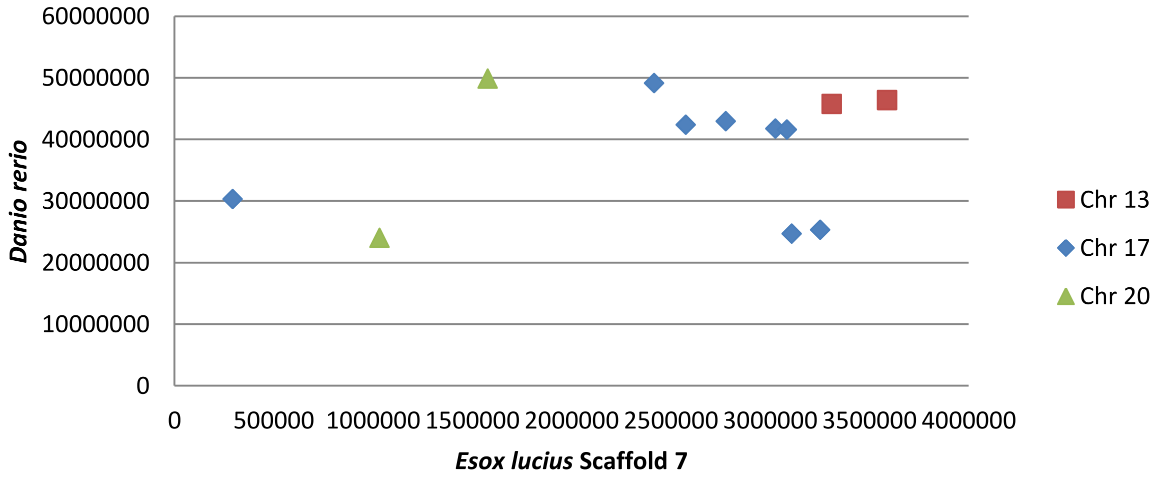

Scaffold 8

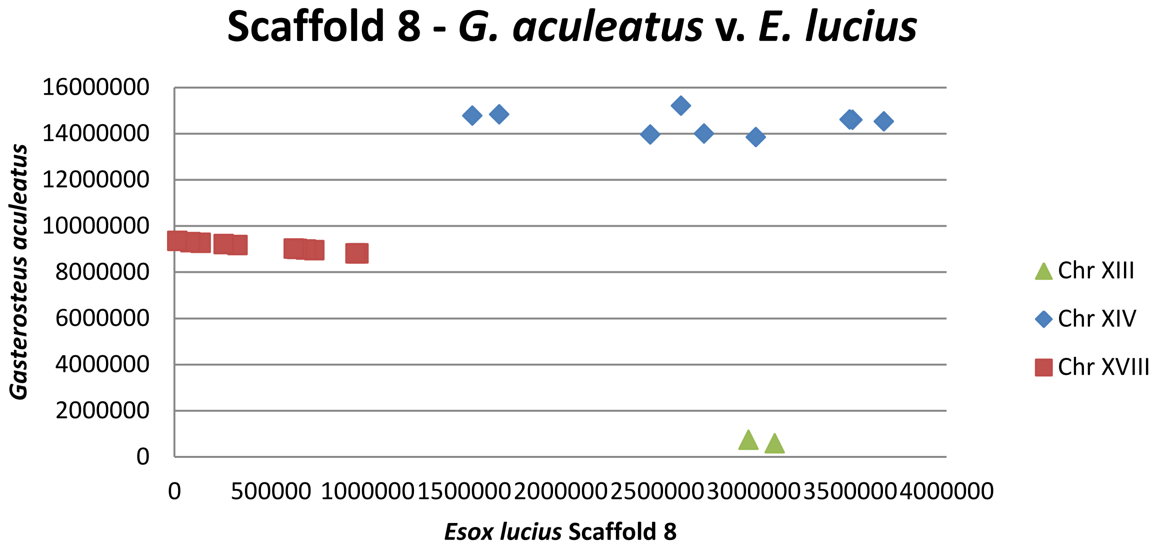
Scaffold 8 - G. aculeatus v. E. lucius
Gasterosteus aculeatus
16000000
14000000
12000000
10000000
8000000
6000000
4000000
2000000
0
0
500000
1000000
1500000
2000000
2500000
3000000
3500000
4000000
Esox lucius Scaffold 8
Chr XIII
Chr XIV
Chr XVIII

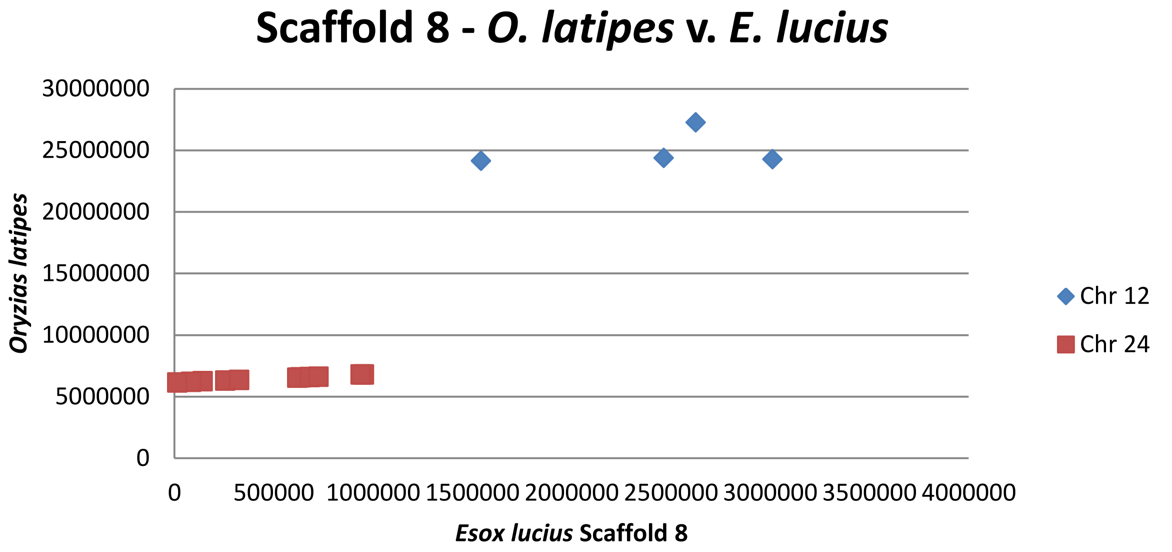
Scaffold 8 - O. latipes v. E. lucius
Oryzias latipes
30000000
25000000
20000000
15000000
10000000
5000000
0
0
500000
1000000
1500000
2000000
2500000
3000000
3500000
4000000
Esox lucius Scaffold 8
Chr 12
Chr 24

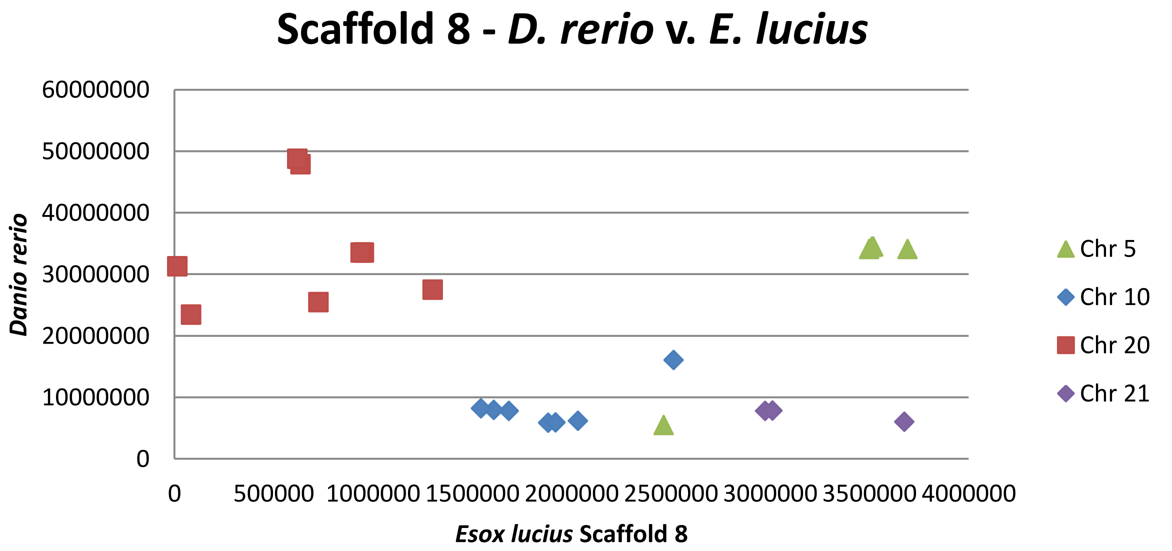
Scaffold 8 - D. rerio v. E. lucius
Danio rerio
60000000
50000000
40000000
30000000
20000000
10000000
0
0
500000
1000000
1500000
2000000
2500000
3000000
3500000
4000000
Esox lucius Scaffold 8
Chr 5
Chr 10
Chr 20
Chr 21

Scaffold 9

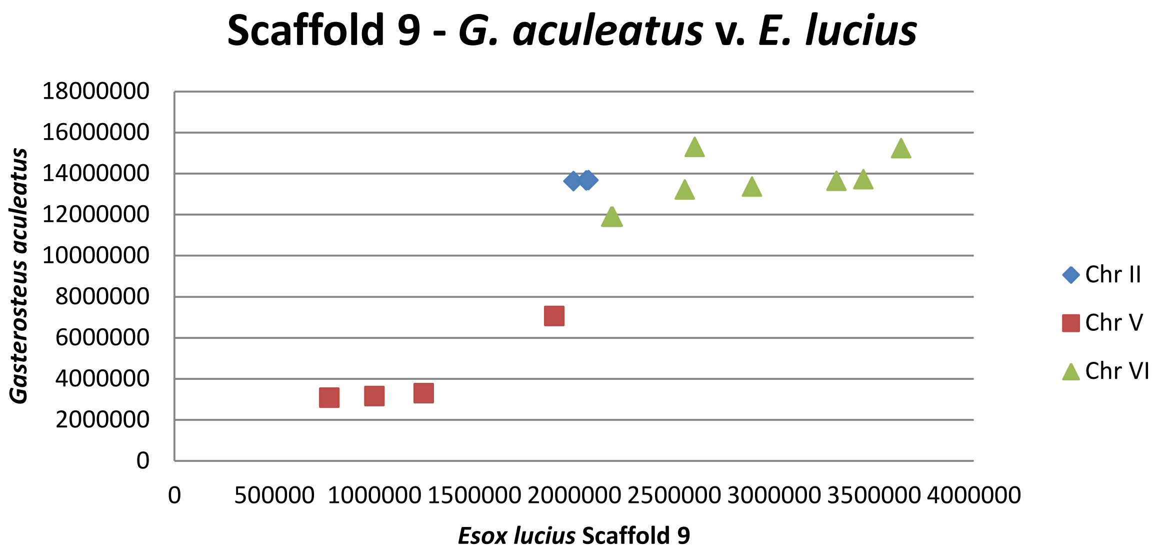
Scaffold 9 - G. aculeatus v. E. lucius
Gasterosteus aculeatus
Esox lucius Scaffold 9
Chr II
Chr V
Chr VI
18000000
16000000
14000000
12000000
10000000
8000000
6000000
4000000
2000000
0
500000 1000000 1500000 2000000 2500000 3000000 3500000 4000000

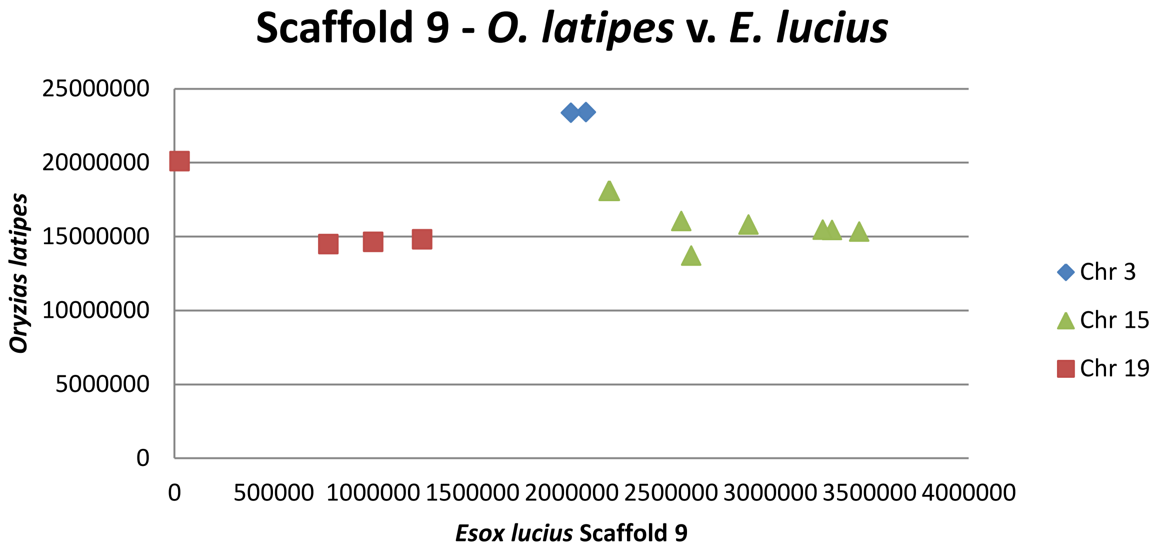
Scaffold 9 - O. latipes v. E. lucius
Oryzias latipes
Esox lucius Scaffold 9
Chr 3
Chr 15
Chr 19
25000000
20000000
15000000
10000000
5000000
0
500000 1000000 1500000 2000000 2500000 3000000 3500000 4000000

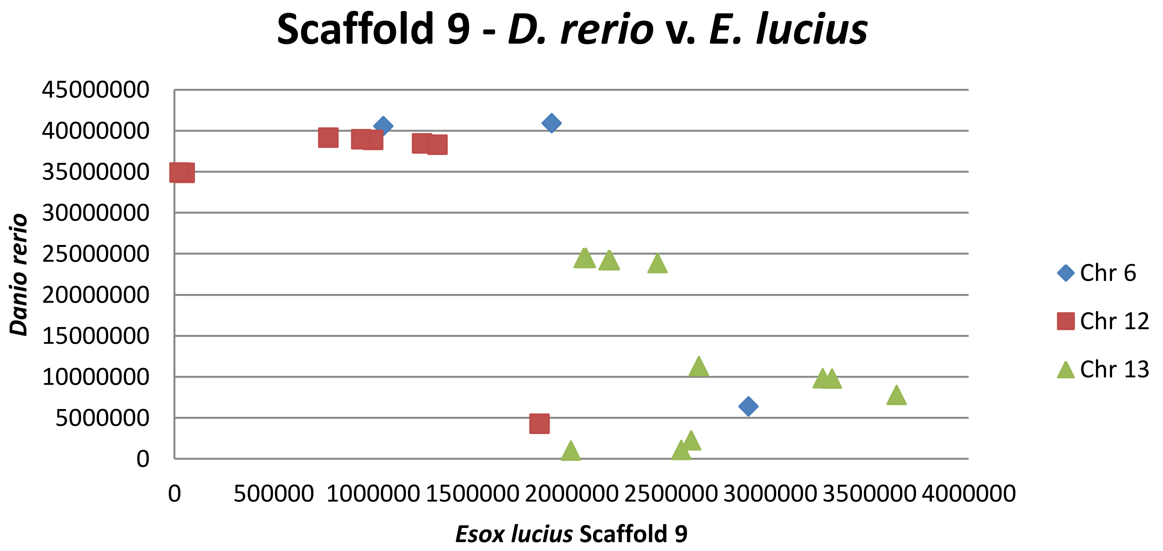
Scaffold 9 - D. rerio v. E. lucius
Danio rerio
Esox lucius Scaffold 9
Chr 6
Chr 12
Chr 13
45000000
40000000
35000000
30000000
25000000
20000000
15000000
10000000
5000000
0
500000 1000000 1500000 2000000 2500000 3000000 3500000 4000000

Scaffold 10

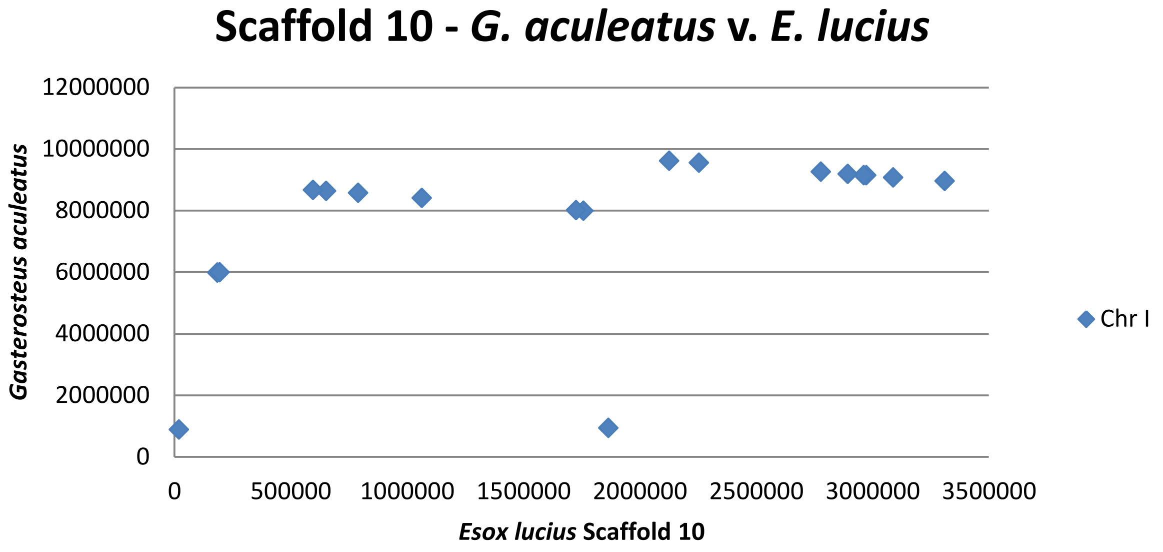
Scaffold 10 - G. aculeatus v. E. lucius
12000000
10000000
8000000
6000000
4000000
2000000
0
Gasterosteus aculeatus
0
500000
1000000
1500000
2000000
2500000
3000000
3500000
Esox lucius Scaffold 10
Chr I

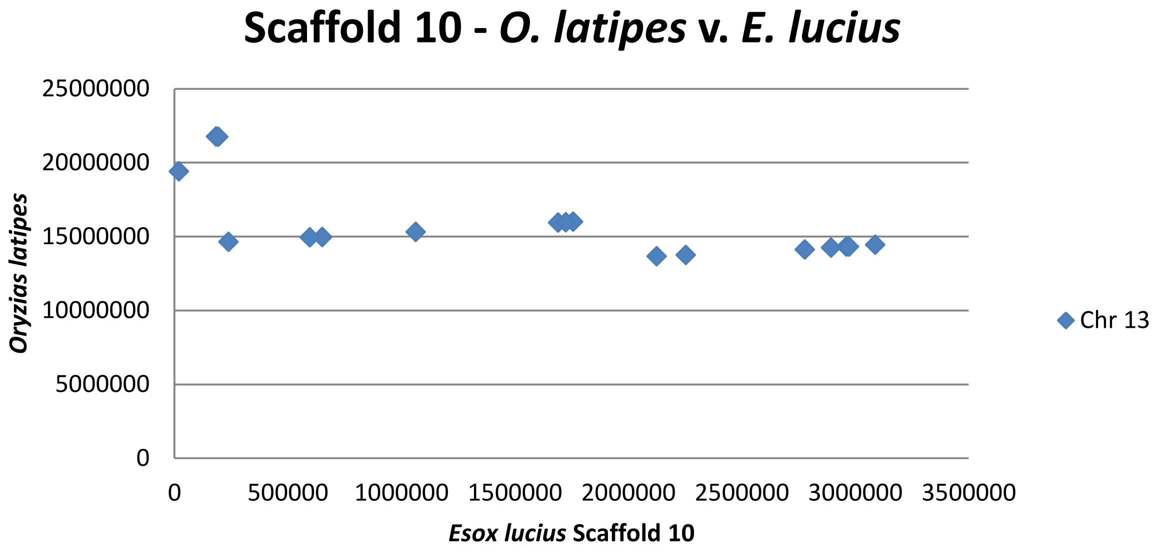
Scaffold 10 - O. latipes v. E. lucius
25000000
20000000
15000000
10000000
5000000
0
Oryzias latipes
0
500000
1000000
1500000
2000000
2500000
3000000
3500000
Esox lucius Scaffold 10
Chr 13

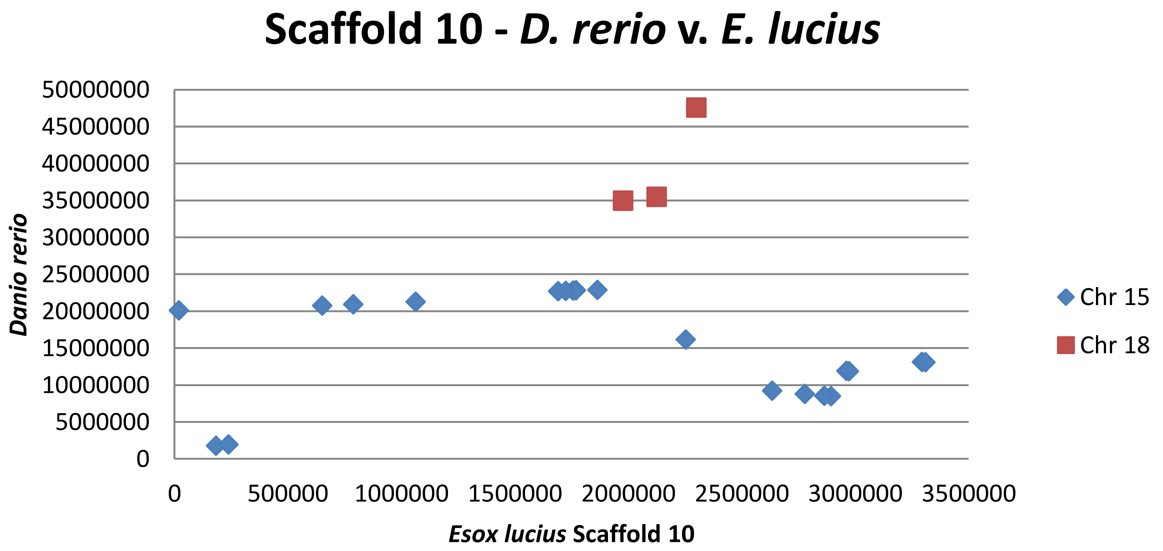
Scaffold 10 - D. rerio v. E. lucius
50000000
45000000
40000000
35000000
30000000
25000000
20000000
15000000
10000000
5000000
0
Danio rerio
0
500000
1000000
1500000
2000000
2500000
3000000
3500000
Esox lucius Scaffold 10
Chr 15
Chr 18

Scaffold 11

Scaffold 11 - *G. aculeatus* v. *E. lucius*

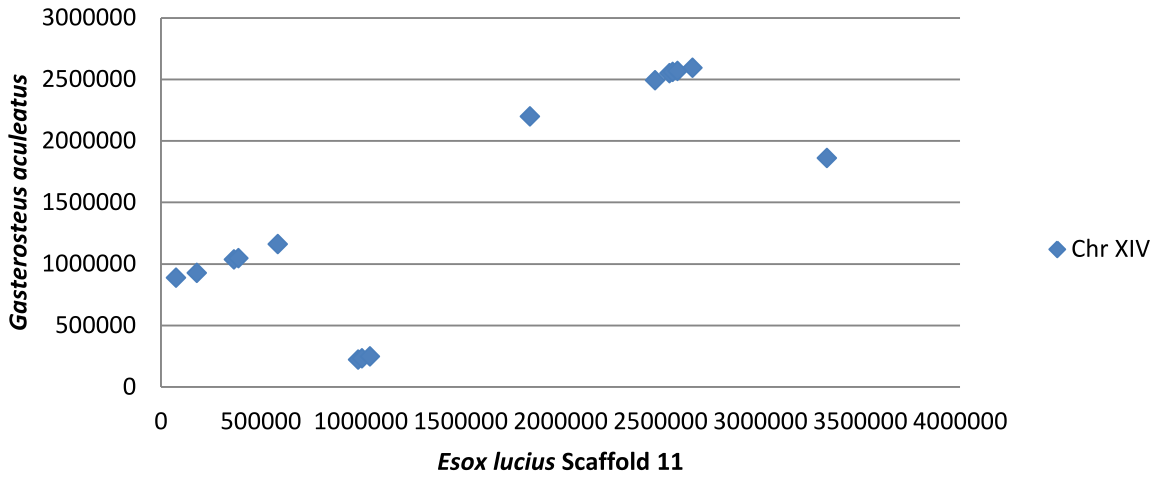

Scaffold 11 - *O. latipes* v. *E. lucius*

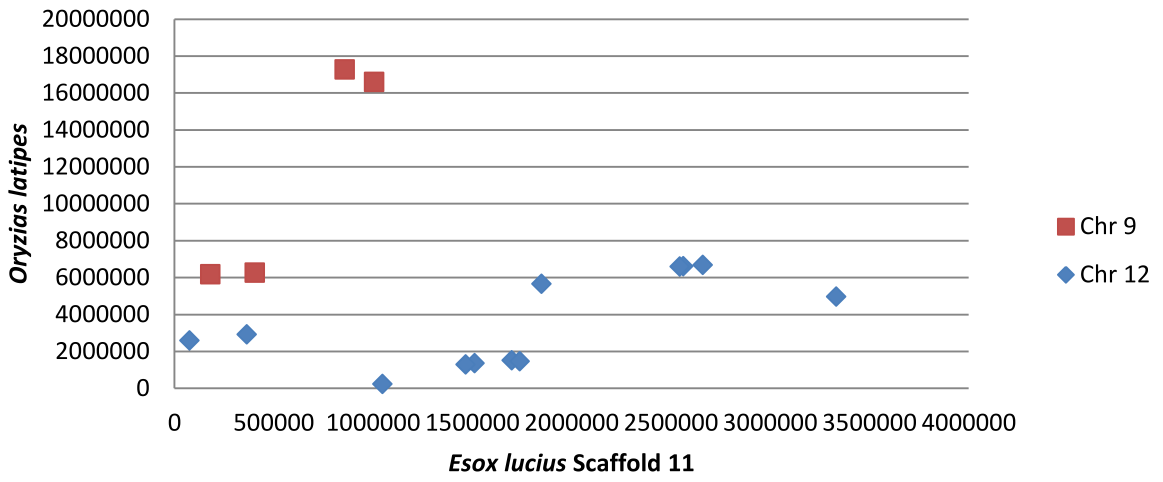

Scaffold 11 - *D. rerio* v. *E. lucius*

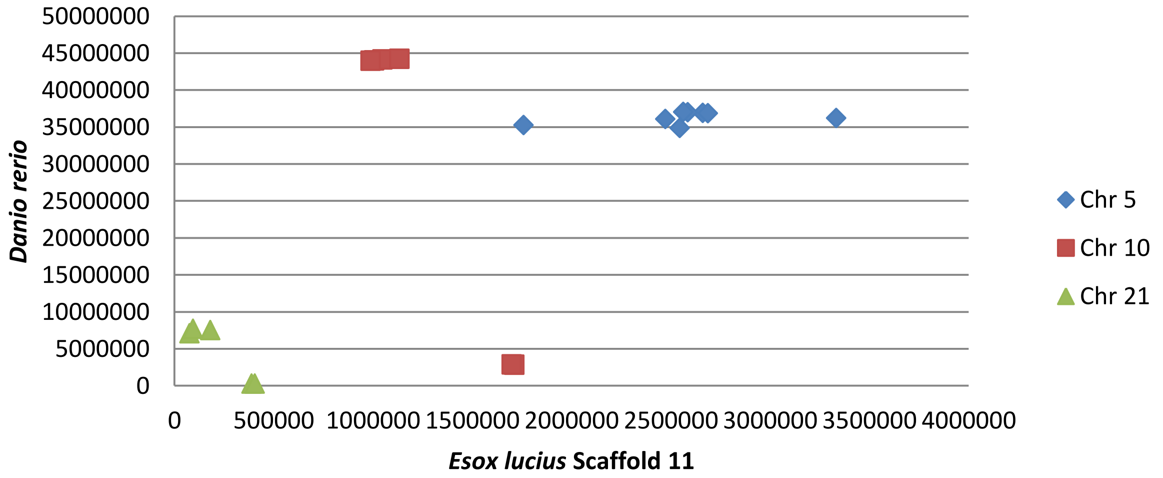

Scaffold 12

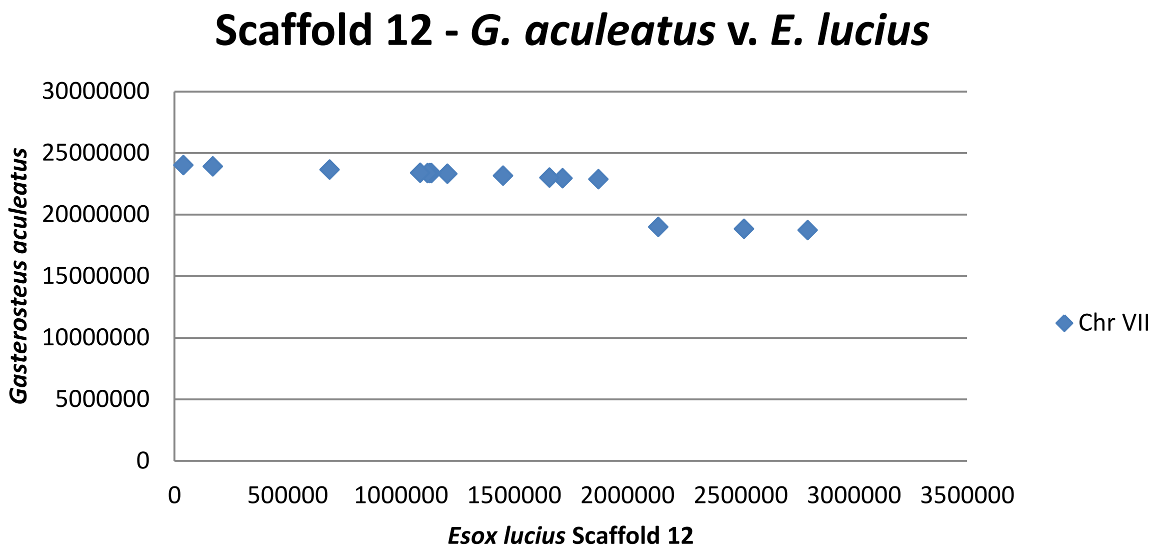

Scaffold 12 - *G. aculeatus* v. *E. lucius*
30000000
25000000
20000000
15000000
10000000
5000000
0
0
500000
1000000
1500000
2000000
2500000
3000000
3500000
Gasterosteus aculeatus
Esox lucius Scaffold 12
Chr VII

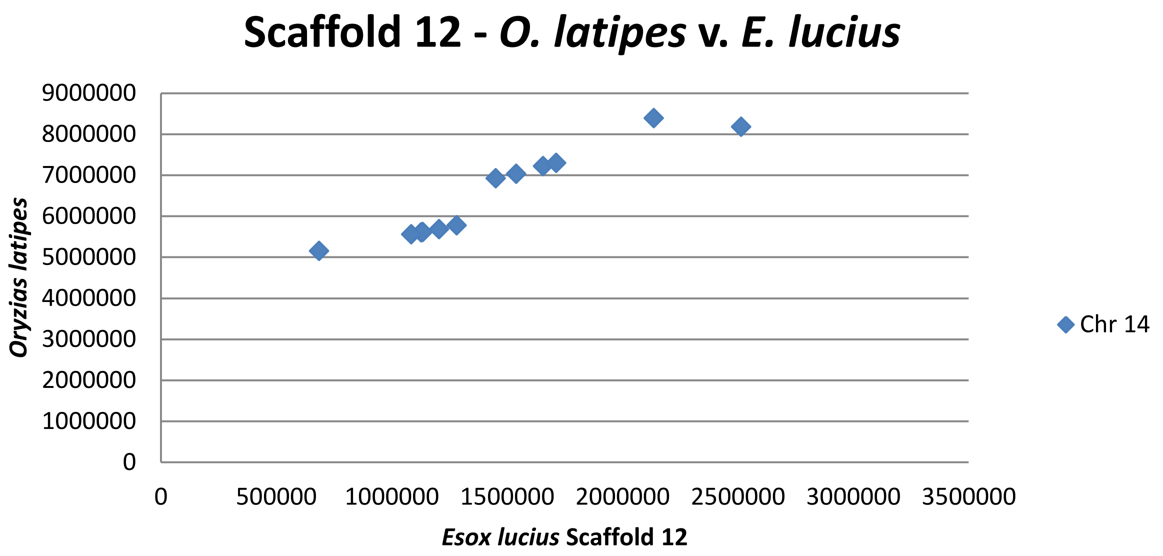

Scaffold 12 - *O. latipes* v. *E. lucius*
9000000
8000000
7000000
6000000
5000000
4000000
3000000
2000000
1000000
0
0
500000
1000000
1500000
2000000
2500000
3000000
3500000
Oryzias latipes
Esox lucius Scaffold 12
Chr 14

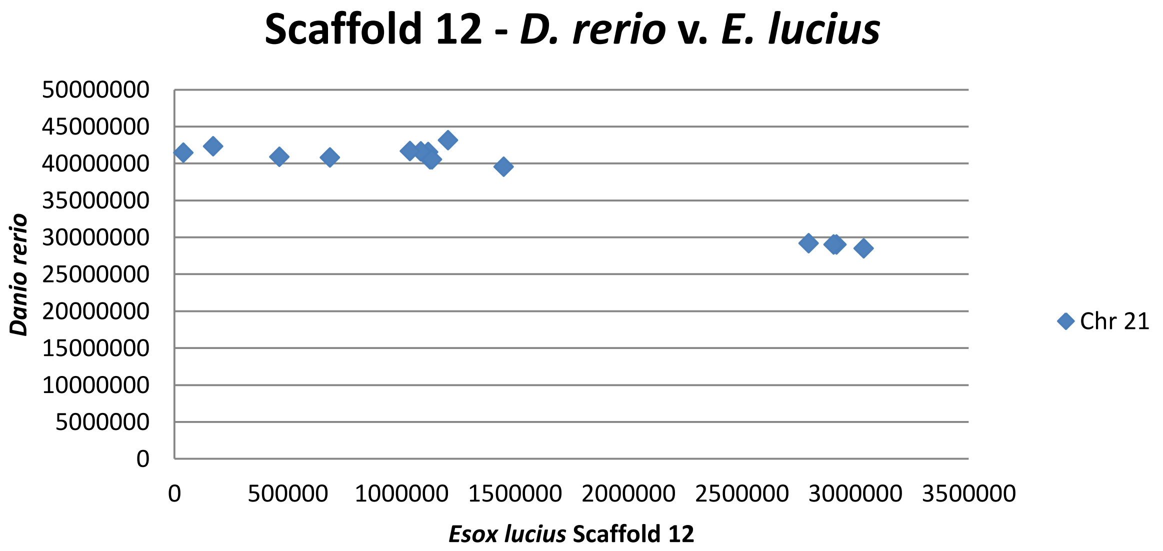

Scaffold 12 - *D. rerio* v. *E. lucius*
50000000
45000000
40000000
35000000
30000000
25000000
20000000
15000000
10000000
5000000
0
0
500000
1000000
1500000
2000000
2500000
3000000
3500000
Danio rerio
Esox lucius Scaffold 12
Chr 21

Scaffold 13

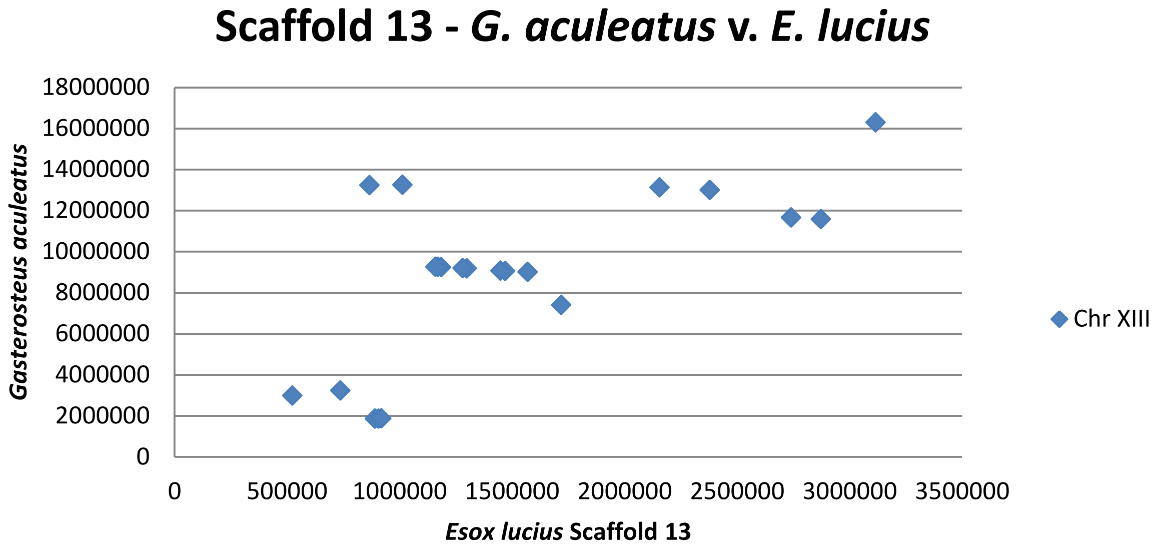
Scaffold 13 - *G. aculeatus* v. *E. lucius*
18000000
16000000
14000000
12000000
10000000
8000000
6000000
4000000
2000000
0
Gasterosteus aculeatus
0
500000
1000000
1500000
2000000
2500000
3000000
3500000
Esox lucius Scaffold 13
Chr XIII

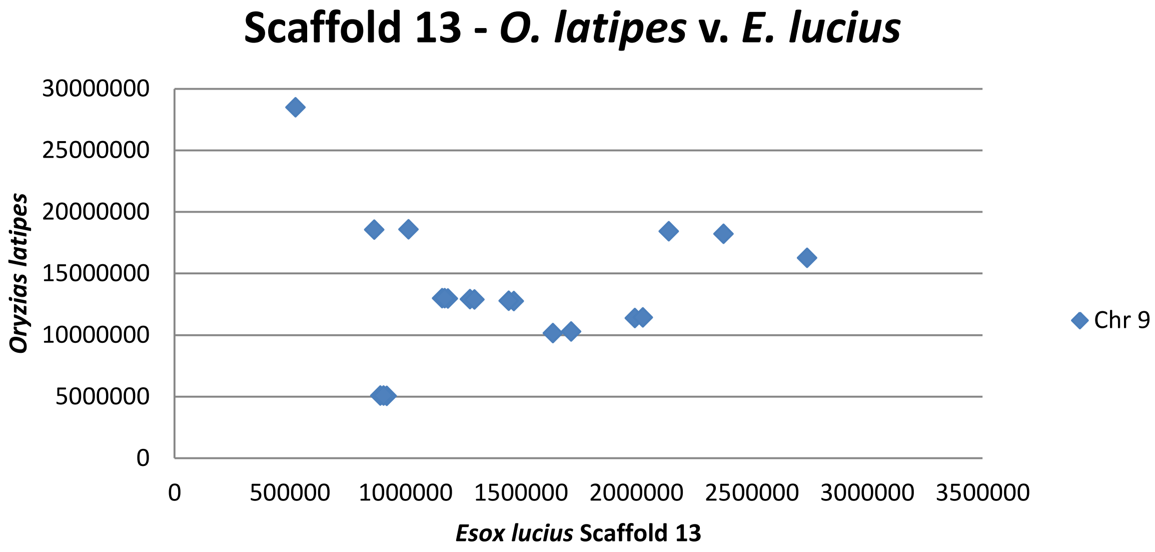
Scaffold 13 - *O. latipes* v. *E. lucius*
30000000
25000000
20000000
15000000
10000000
5000000
0
Oryzias latipes
0
500000
1000000
1500000
2000000
2500000
3000000
3500000
Esox lucius Scaffold 13
Chr 9

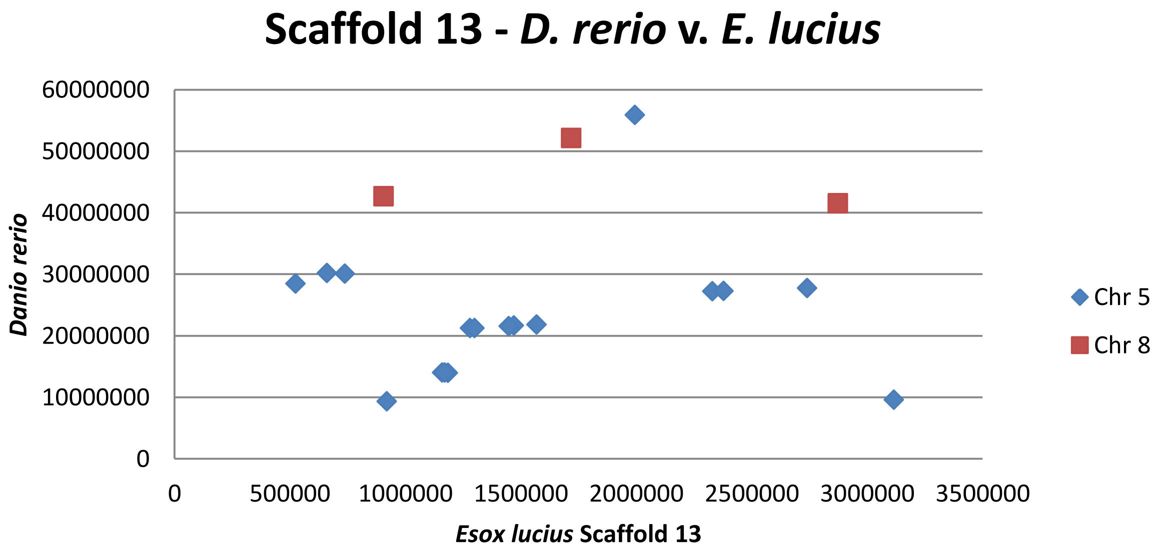
Scaffold 13 - *D. rerio* v. *E. lucius*
60000000
50000000
40000000
30000000
20000000
10000000
0
Danio rerio
0
500000
1000000
1500000
2000000
2500000
3000000
3500000
Esox lucius Scaffold 13
Chr 5
Chr 8

Scaffold 14

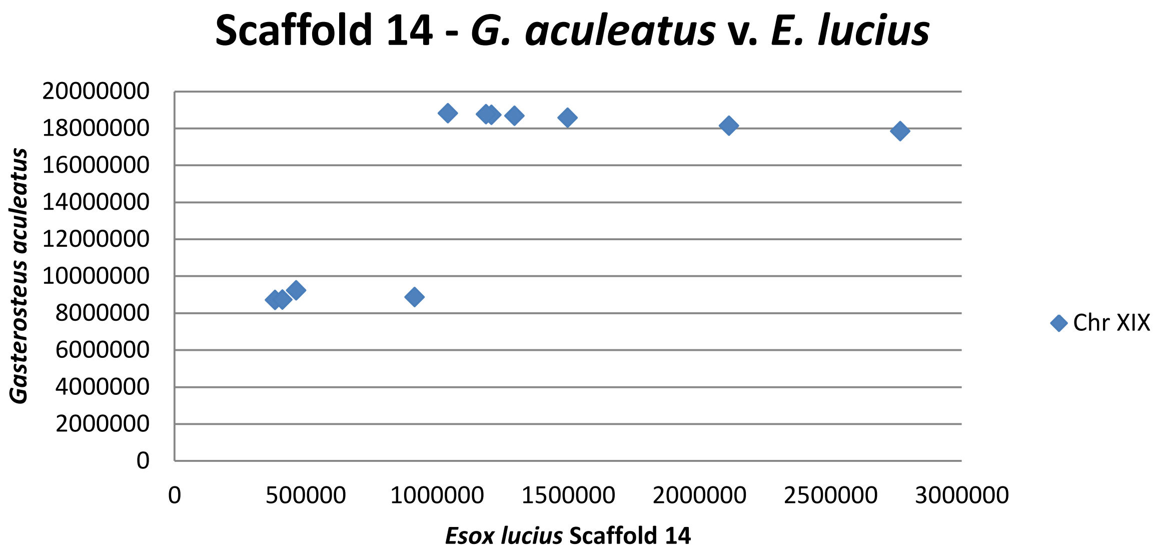

Scaffold 14 - *G. aculeatus* v. *E. lucius*
Gasterosteus aculeatus
20000000
18000000
16000000
14000000
12000000
10000000
8000000
6000000
4000000
2000000
0
0
500000
1000000
1500000
2000000
2500000
3000000
Esox lucius Scaffold 14
Chr XIX

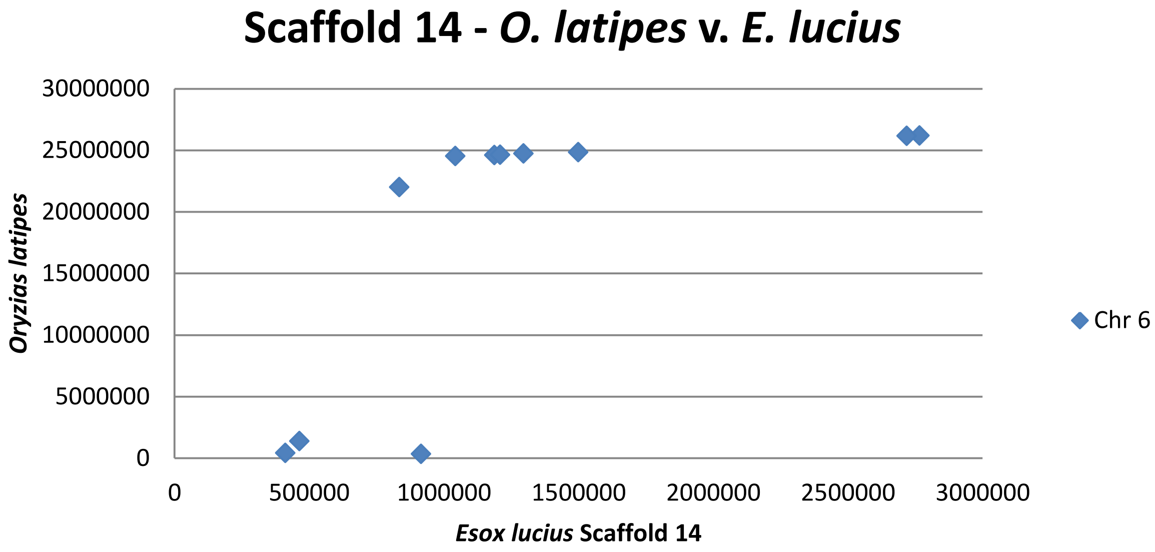

Scaffold 14 - *O. latipes* v. *E. lucius*
Oryzias latipes
30000000
25000000
20000000
15000000
10000000
5000000
0
0
500000
1000000
1500000
2000000
2500000
3000000
Esox lucius Scaffold 14
Chr 6

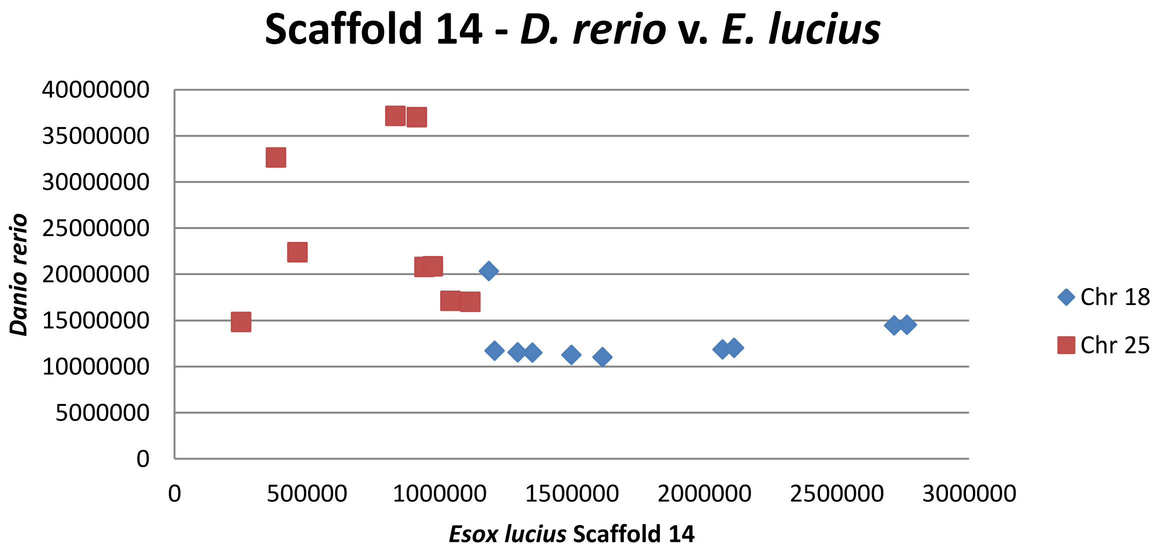

Scaffold 14 - *D. rerio* v. *E. lucius*
Danio rerio
40000000
35000000
30000000
25000000
20000000
15000000
10000000
5000000
0
0
500000
1000000
1500000
2000000
2500000
3000000
Esox lucius Scaffold 14
Chr 18
Chr 25

Scaffold 15

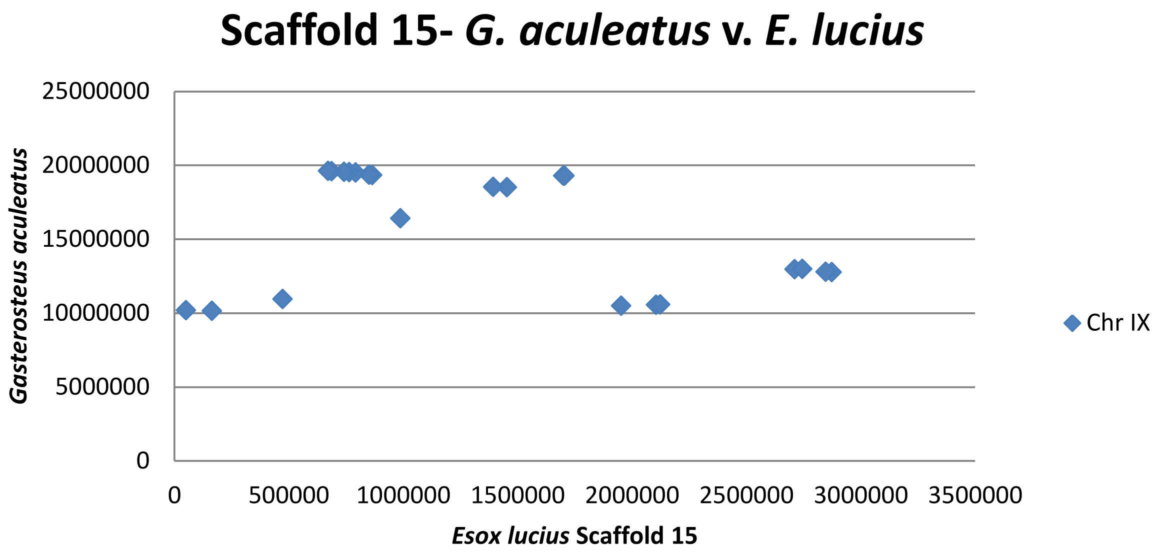
Scaffold 15- G. aculeatus v. E. lucius
Gasterosteus aculeatus
25000000
20000000
15000000
10000000
5000000
0
0
500000
1000000
1500000
2000000
2500000
3000000
3500000
Esox lucius Scaffold 15
Chr IX

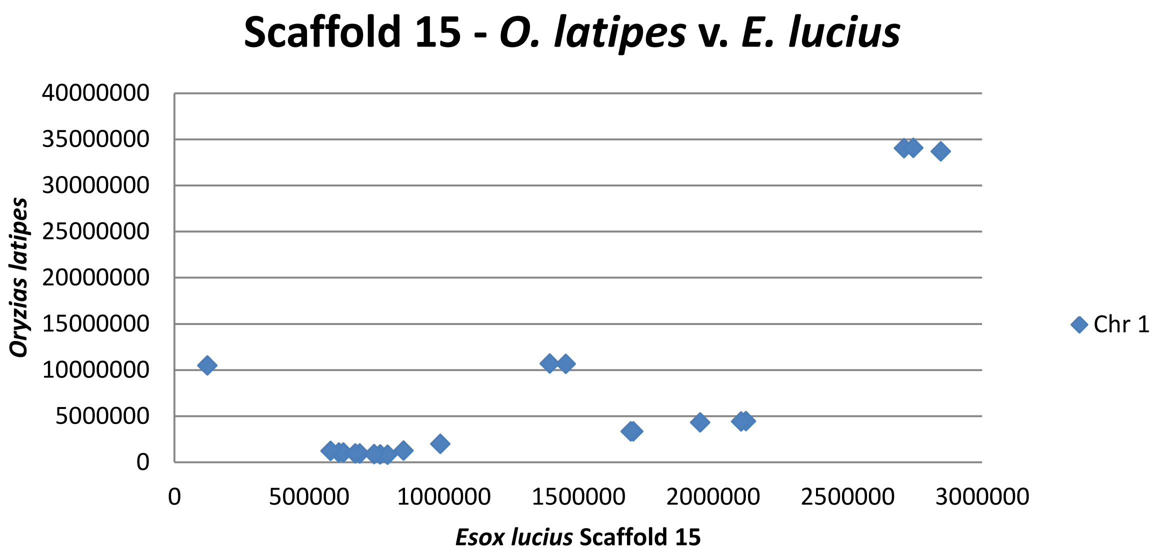
Scaffold 15 - O. latipes v. E. lucius
Oryzias latipes
40000000
35000000
30000000
25000000
20000000
15000000
10000000
5000000
0
0
500000
1000000
1500000
2000000
2500000
3000000
Esox lucius Scaffold 15
Chr 1

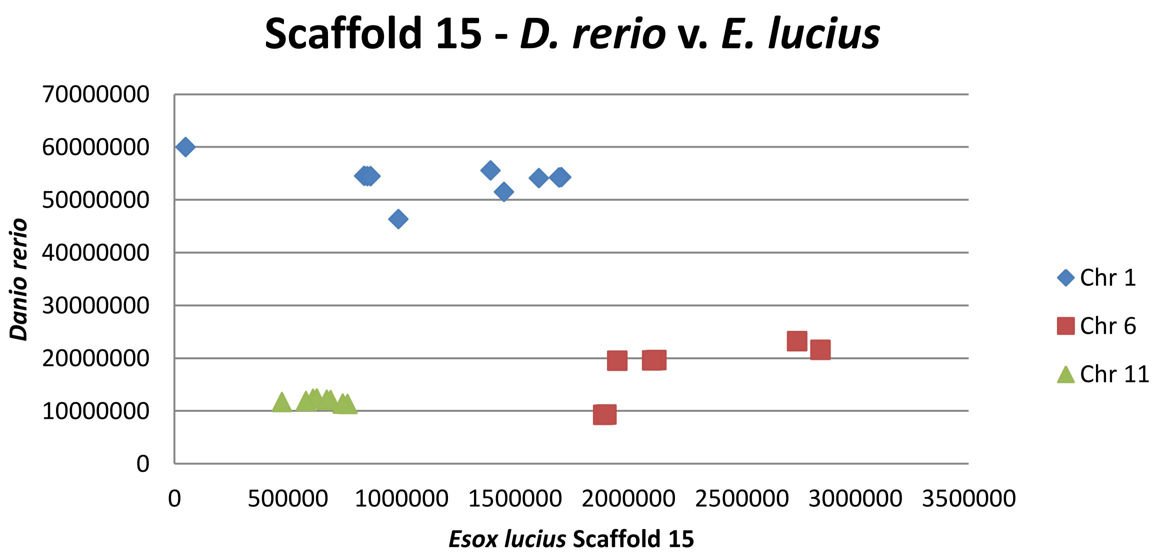
Scaffold 15 - D. rerio v. E. lucius
Danio rerio
70000000
60000000
50000000
40000000
30000000
20000000
10000000
0
0
500000
1000000
1500000
2000000
2500000
3000000
3500000
Esox lucius Scaffold 15
Chr 1
Chr 6
Chr 11

Scaffold 16

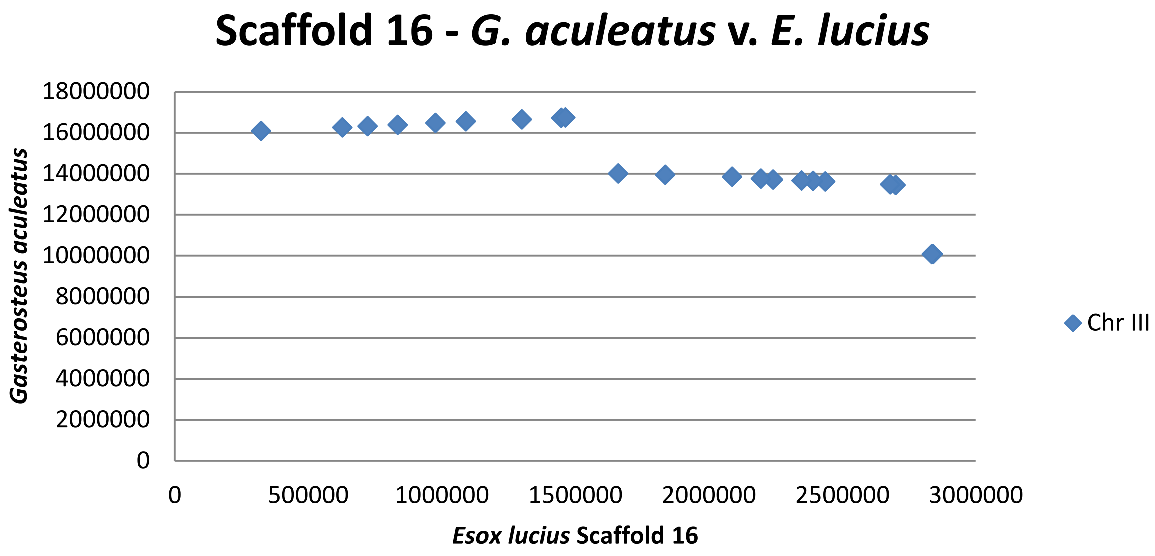
Scaffold 16 - *G. aculeatus* v. *E. lucius*
18000000
16000000
14000000
12000000
10000000
8000000
6000000
4000000
2000000
0
0
500000
1000000
1500000
2000000
2500000
3000000
Gasterosteus aculeatus
Esox lucius Scaffold 16
Chr III

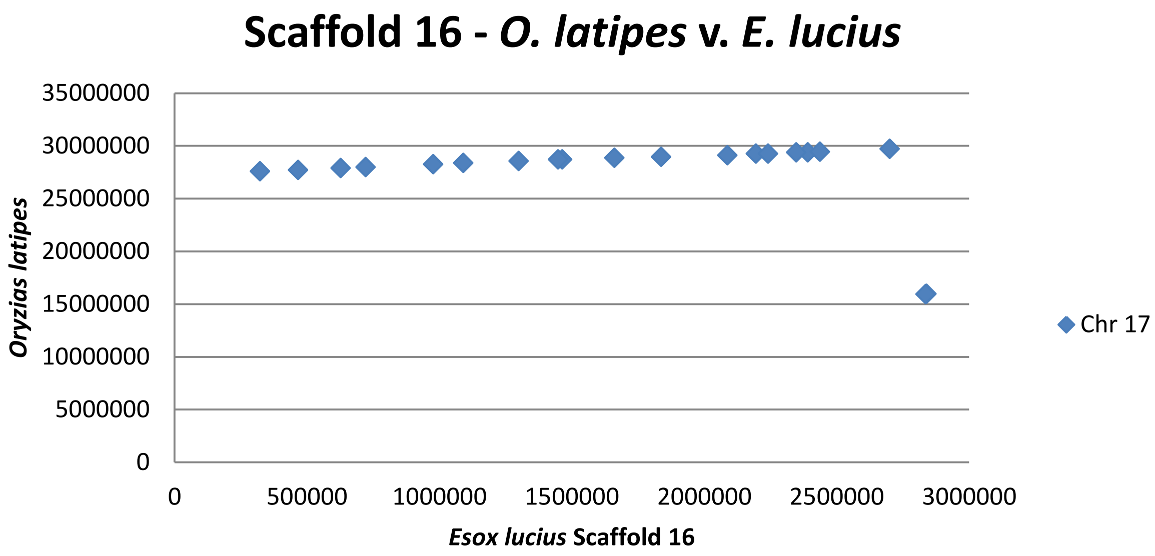
Scaffold 16 - *O. latipes* v. *E. lucius*
35000000
30000000
25000000
20000000
15000000
10000000
5000000
0
0
500000
1000000
1500000
2000000
2500000
3000000
Oryzias latipes
Esox lucius Scaffold 16
Chr 17

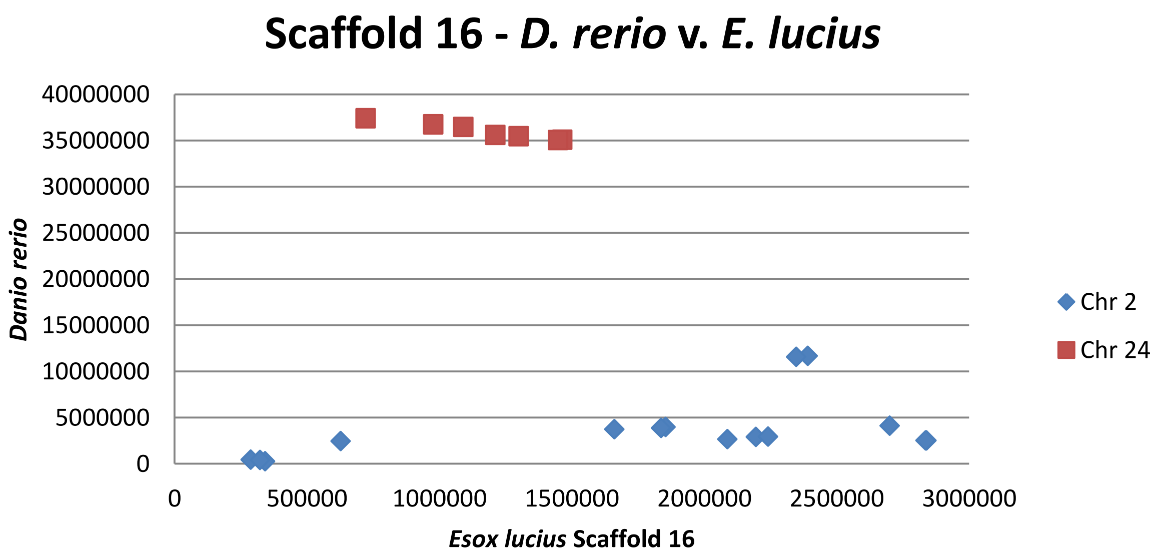
Scaffold 16 - *D. rerio* v. *E. lucius*
40000000
35000000
30000000
25000000
20000000
15000000
10000000
5000000
0
0
500000
1000000
1500000
2000000
2500000
3000000
Danio rerio
Esox lucius Scaffold 16
Chr 2
Chr 24

Scaffold 17

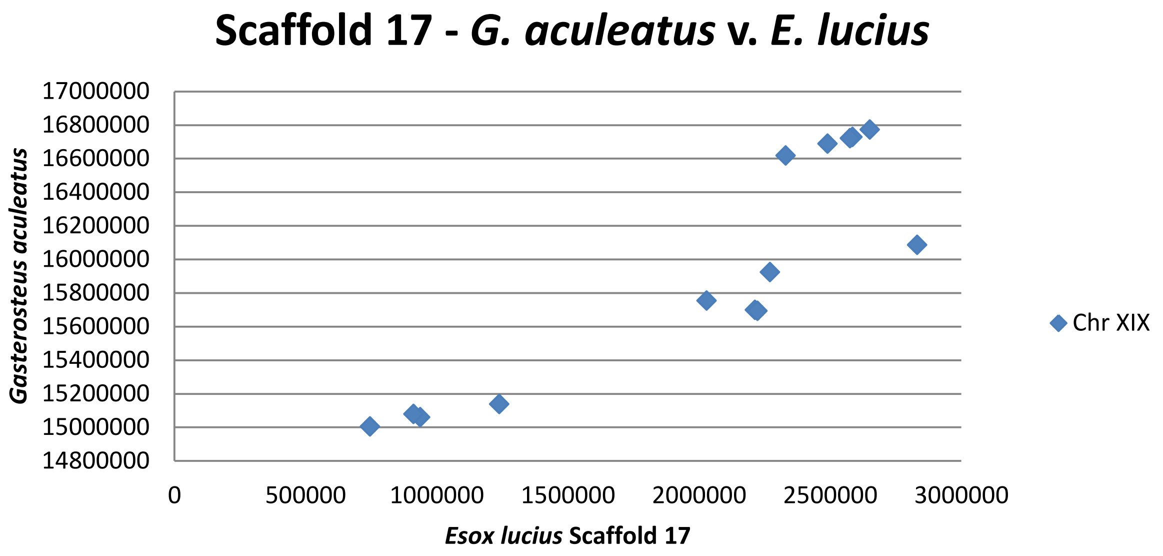
Scaffold 17 - *G. aculeatus* v. *E. lucius*
17000000
16800000
16600000
16400000
16200000
16000000
15800000
15600000
15400000
15200000
15000000
14800000
0
500000
1000000
1500000
2000000
2500000
3000000
Gasterosteus aculeatus
Esox lucius Scaffold 17
Chr XIX

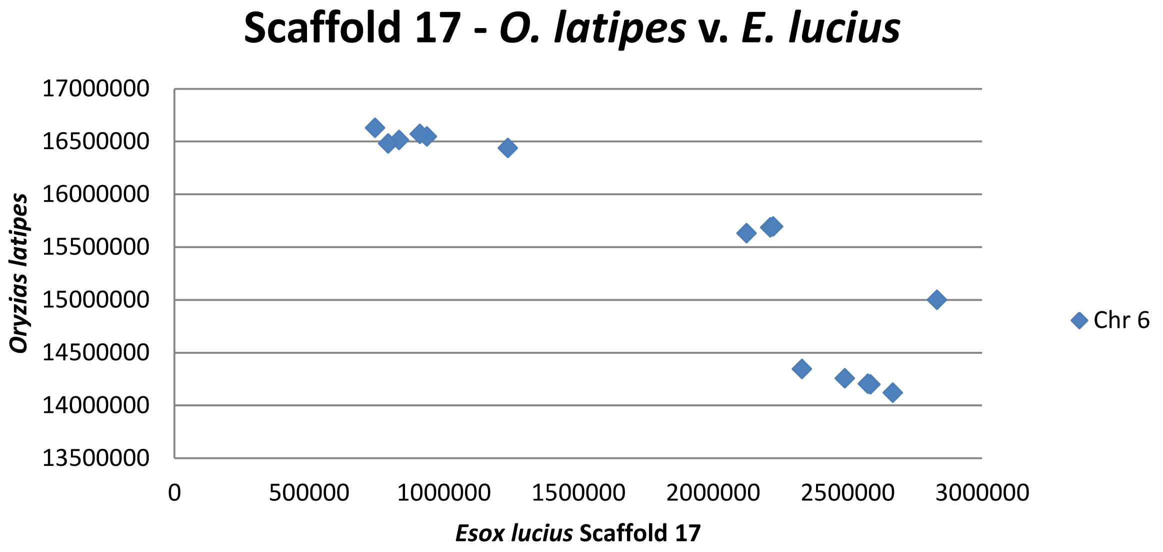
Scaffold 17 - *O. latipes* v. *E. lucius*
17000000
16500000
16000000
15500000
15000000
14500000
14000000
13500000
0
500000
1000000
1500000
2000000
2500000
3000000
Oryzias latipes
Esox lucius Scaffold 17
Chr 6

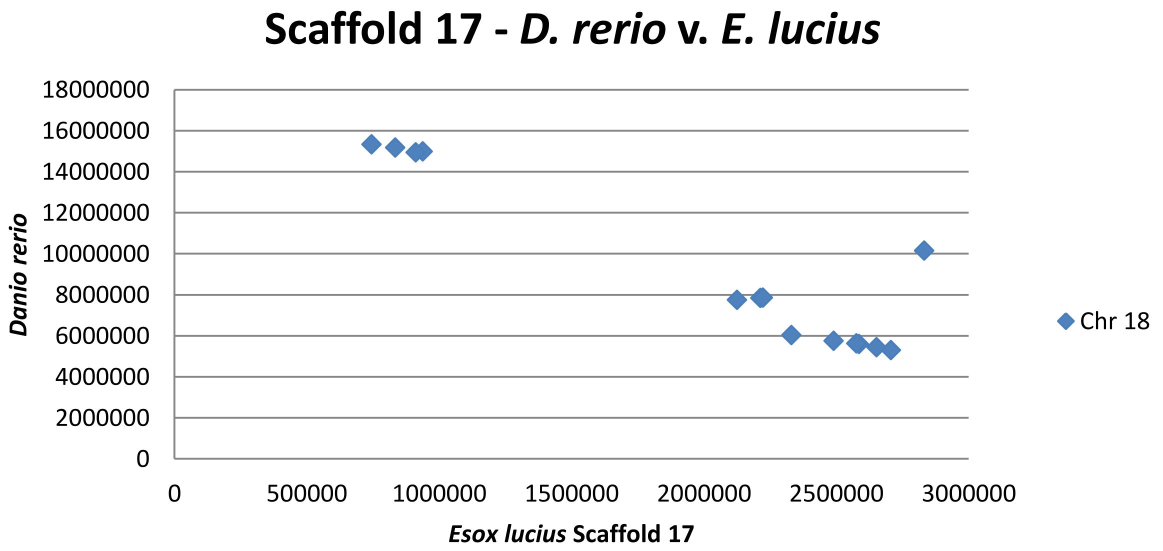
Scaffold 17 - *D. rerio* v. *E. lucius*
18000000
16000000
14000000
12000000
10000000
8000000
6000000
4000000
2000000
0
0
500000
1000000
1500000
2000000
2500000
3000000
Danio rerio
Esox lucius Scaffold 17
Chr 18

Scaffold 18

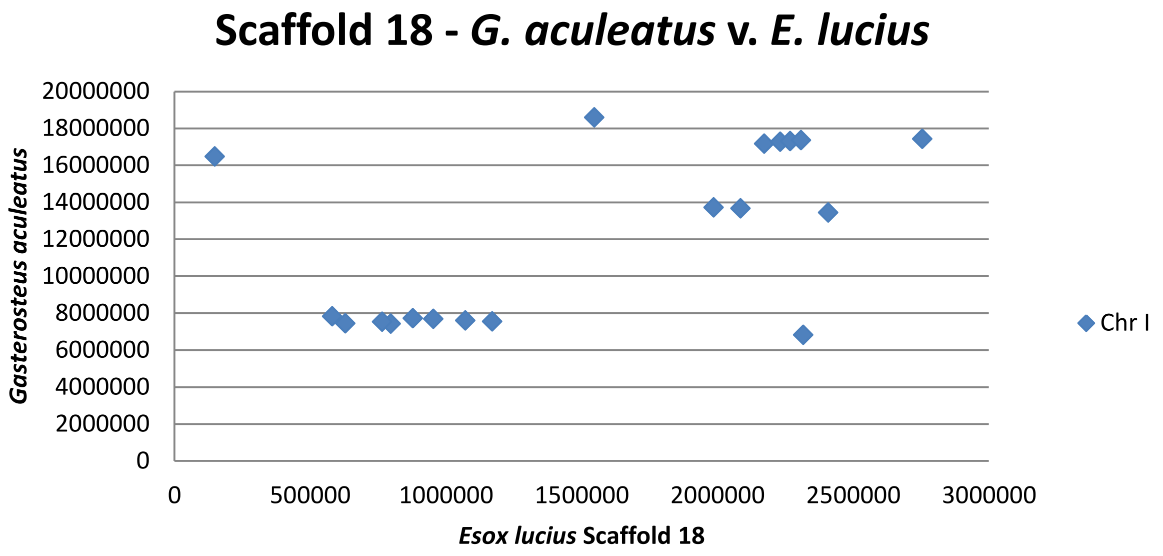
Scaffold 18 - *G. aculeatus* v. *E. lucius*
Gasterosteus aculeatus
20000000
18000000
16000000
14000000
12000000
10000000
8000000
6000000
4000000
2000000
0
0
500000
1000000
1500000
2000000
2500000
3000000
Esox lucius Scaffold 18
Chr I

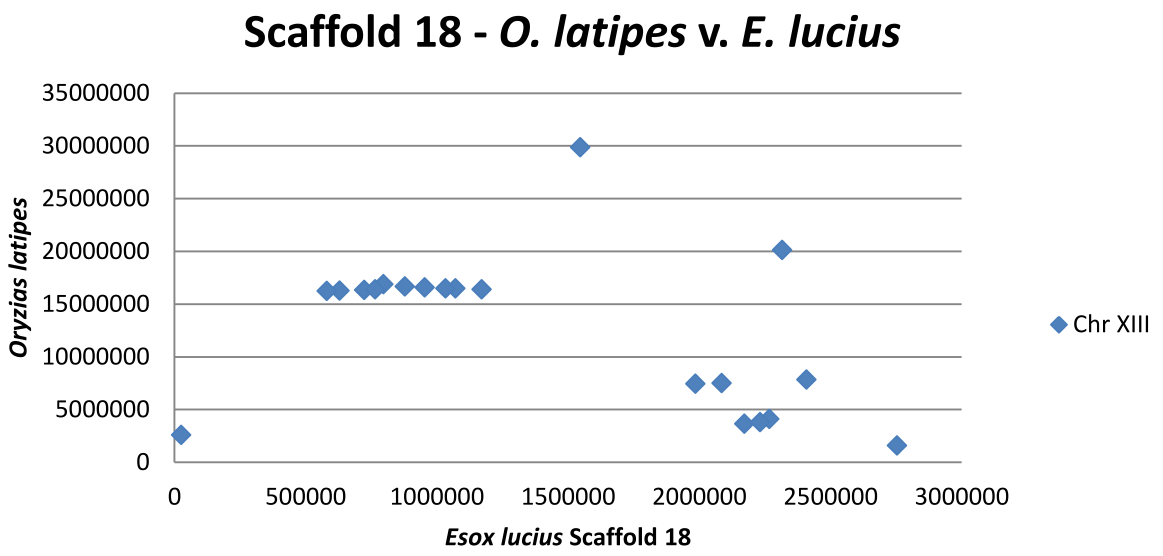
Scaffold 18 - *O. latipes* v. *E. lucius*
Oryzias latipes
35000000
30000000
25000000
20000000
15000000
10000000
5000000
0
0
500000
1000000
1500000
2000000
2500000
3000000
Esox lucius Scaffold 18
Chr XIII

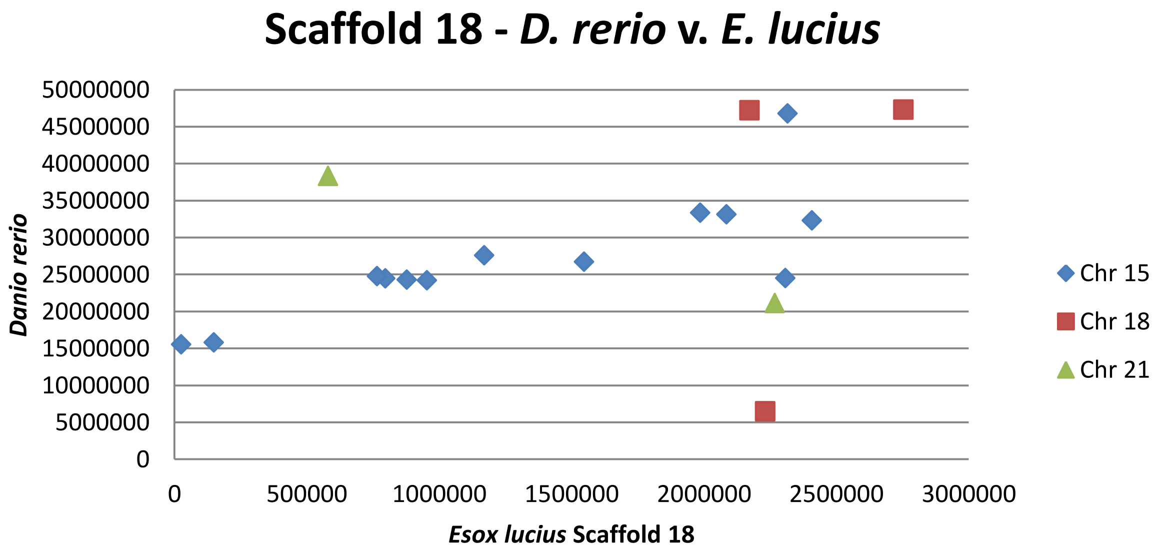
Scaffold 18 - *D. rerio* v. *E. lucius*
Danio rerio
50000000
45000000
40000000
35000000
30000000
25000000
20000000
15000000
10000000
5000000
0
0
500000
1000000
1500000
2000000
2500000
3000000
Esox lucius Scaffold 18
Chr 15
Chr 18
Chr 21

Scaffold 19

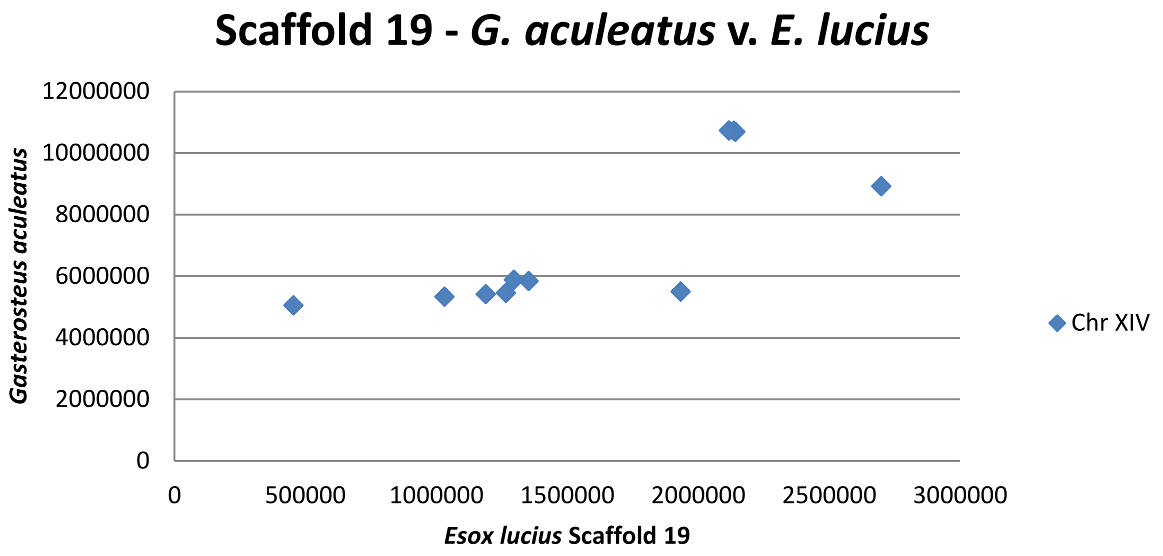

Scaffold 19 - *G. aculeatus* v. *E. lucius*
12000000
10000000
8000000
6000000
4000000
2000000
0
Gasterosteus aculeatus
0
500000
1000000
1500000
2000000
2500000
3000000
Esox lucius Scaffold 19
Chr XIV

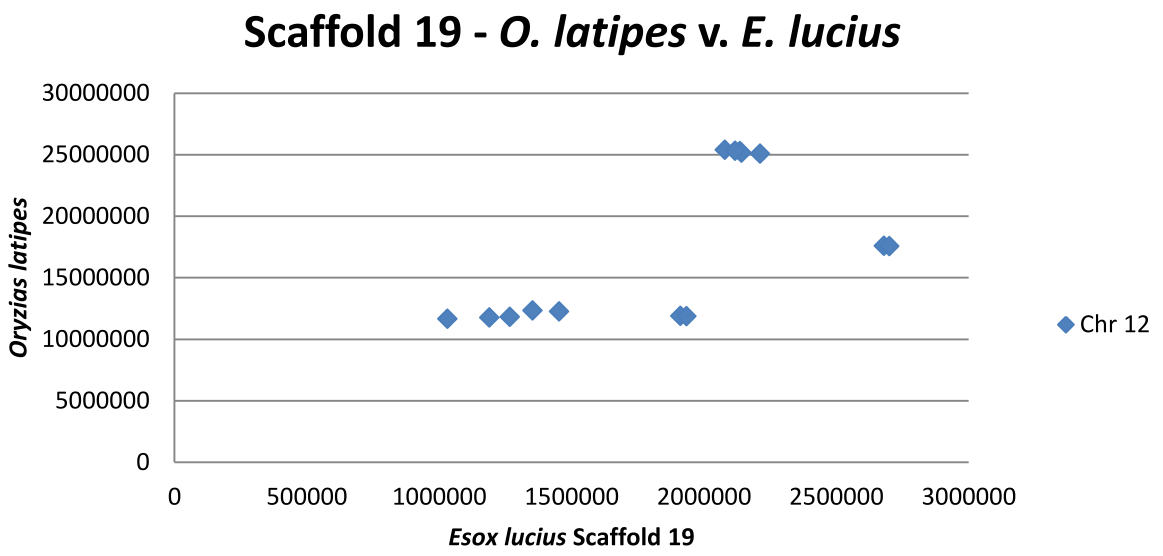

Scaffold 19 - *O. latipes* v. *E. lucius*
30000000
25000000
20000000
15000000
10000000
5000000
0
Oryzias latipes
0
500000
1000000
1500000
2000000
2500000
3000000
Esox lucius Scaffold 19
Chr 12

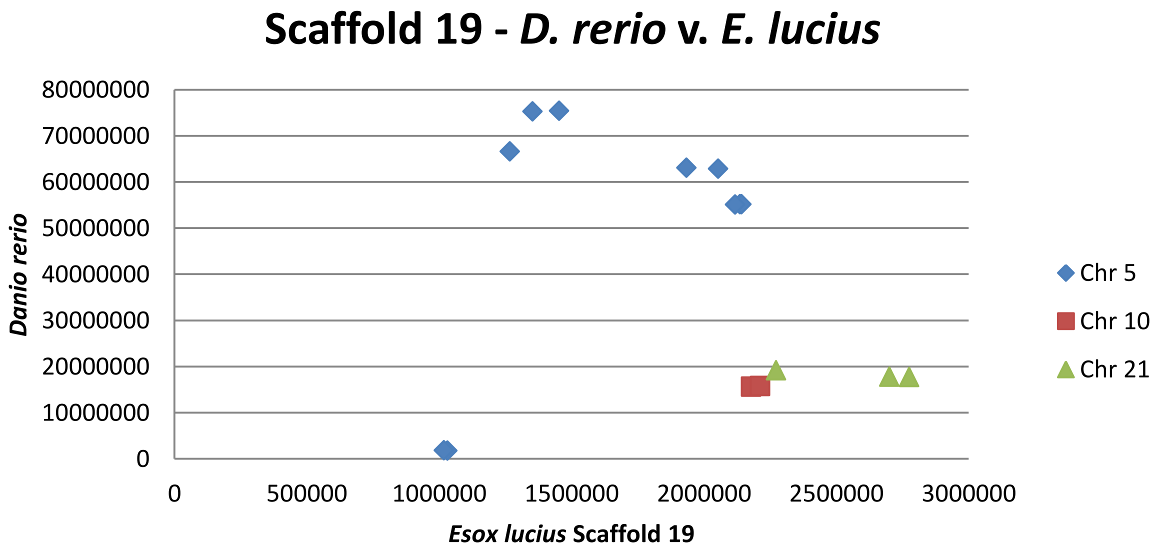

Scaffold 19 - *D. rerio* v. *E. lucius*
80000000
70000000
60000000
50000000
40000000
30000000
20000000
10000000
0
Danio rerio
0
500000
1000000
1500000
2000000
2500000
3000000
Esox lucius Scaffold 19
Chr 5
Chr 10
Chr 21

Scaffold 20

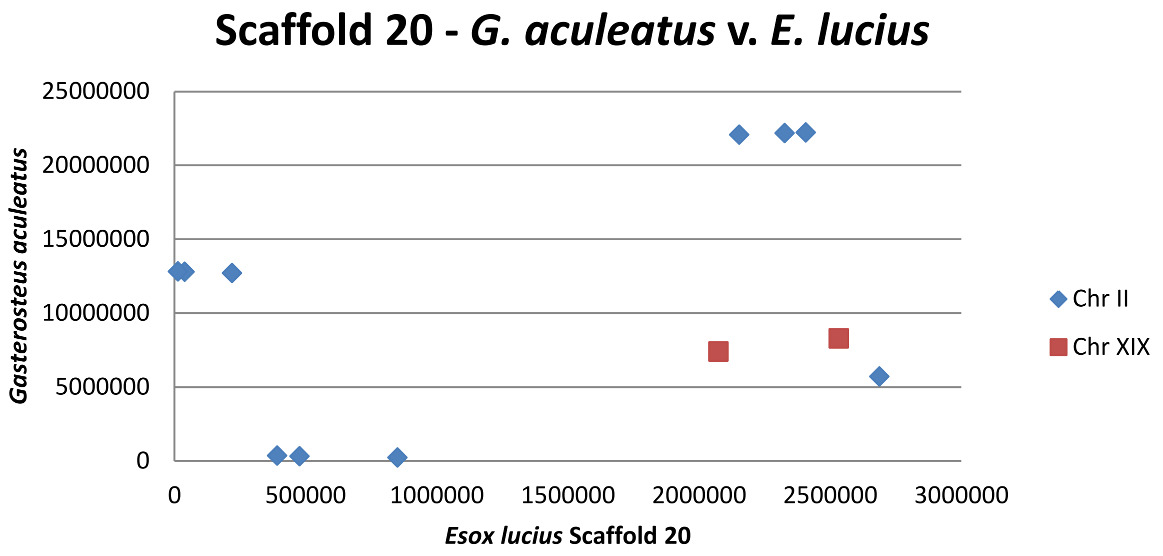
Scaffold 20 - *G. aculeatus* v. *E. lucius*
Gasterosteus aculeatus
25000000
20000000
15000000
10000000
5000000
0
0
500000
1000000
1500000
2000000
2500000
3000000
Esox lucius Scaffold 20
Chr II
Chr XIX

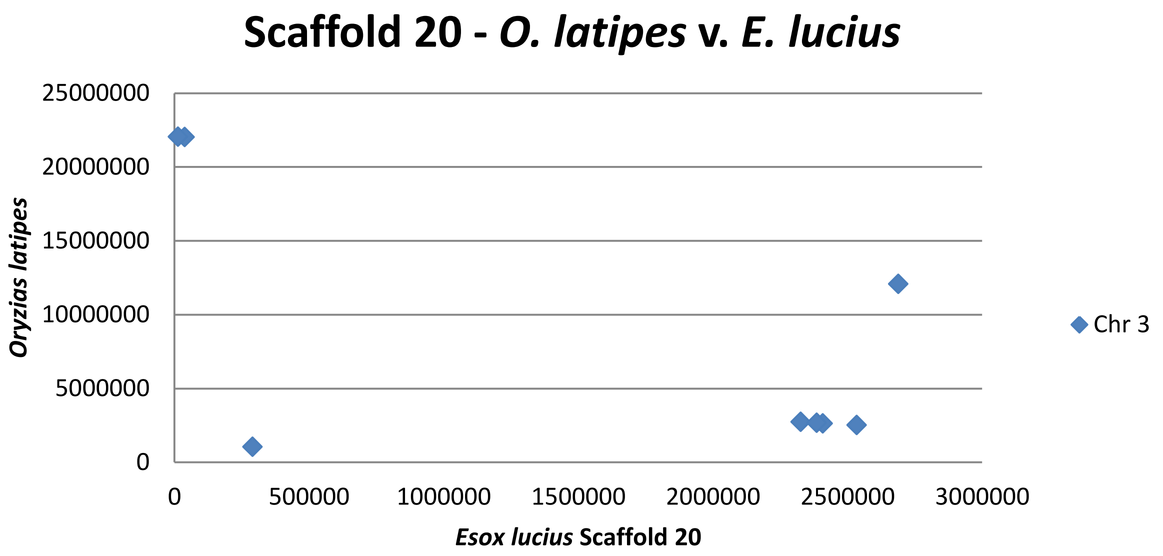
Scaffold 20 - *O. latipes* v. *E. lucius*
Oryzias latipes
25000000
20000000
15000000
10000000
5000000
0
0
500000
1000000
1500000
2000000
2500000
3000000
Esox lucius Scaffold 20
Chr 3

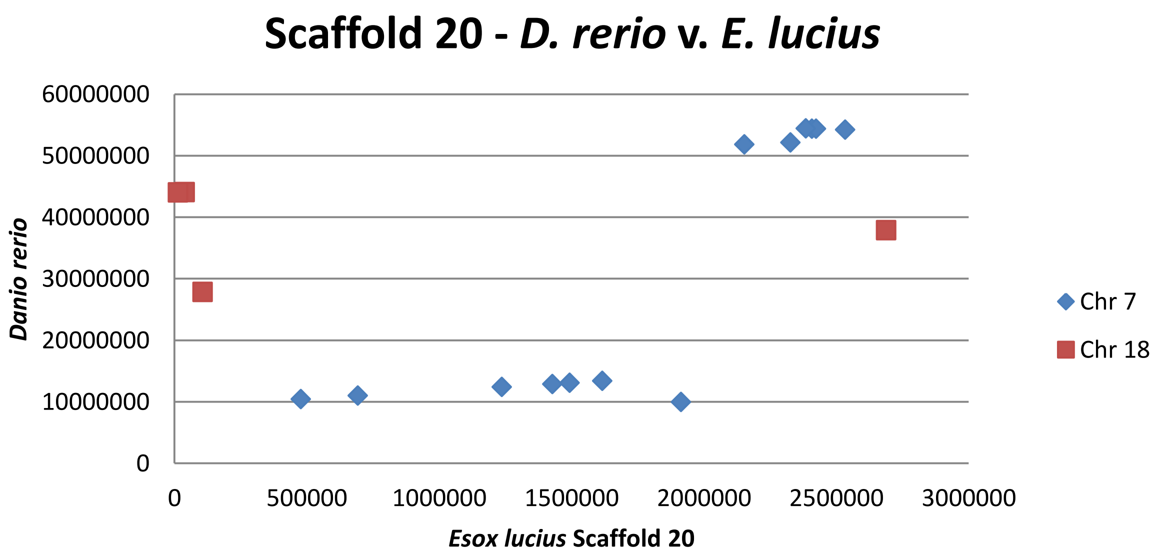
Scaffold 20 - *D. rerio* v. *E. lucius*
Danio rerio
60000000
50000000
40000000
30000000
20000000
10000000
0
0
500000
1000000
1500000
2000000
2500000
3000000
Esox lucius Scaffold 20
Chr 7
Chr 18

Scaffold 21

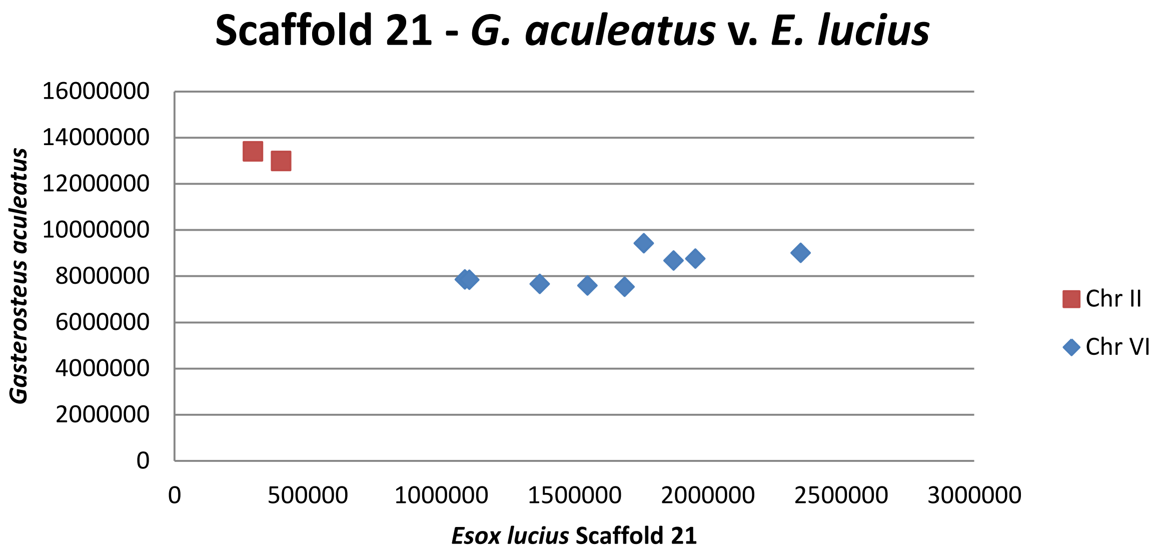
Scaffold 21 - G. aculeatus v. E. lucius
Gasterosteus aculeatus
16000000
14000000
12000000
10000000
8000000
6000000
4000000
2000000
0
0
500000
1000000
1500000
2000000
2500000
3000000
Esox lucius Scaffold 21
Chr II
Chr VI

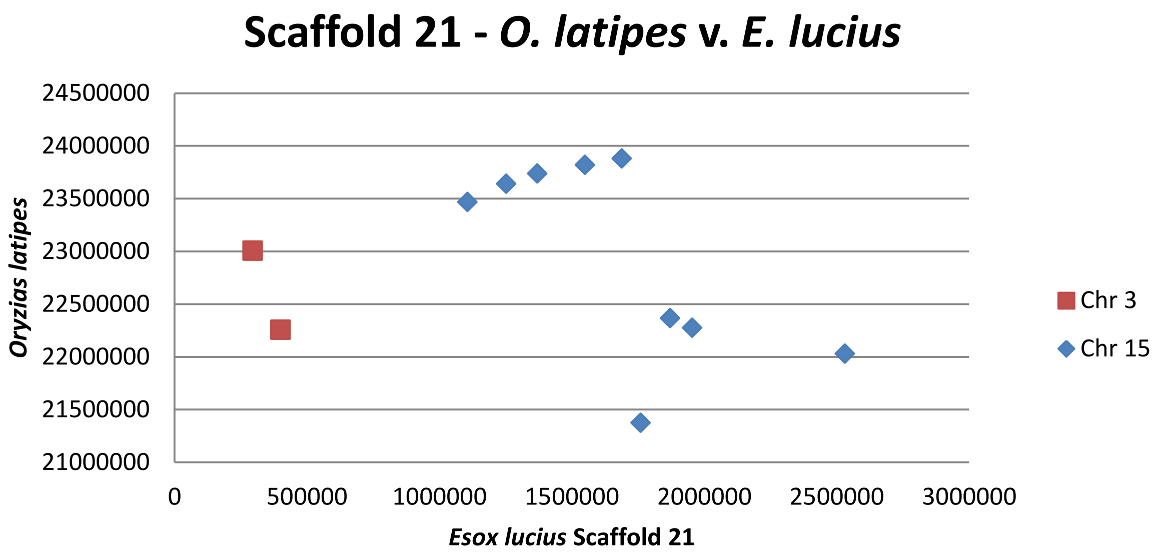
Scaffold 21 - O. latipes v. E. lucius
Oryzias latipes
24500000
24000000
23500000
23000000
22500000
22000000
21500000
21000000
0
500000
1000000
1500000
2000000
2500000
3000000
Esox lucius Scaffold 21
Chr 3
Chr 15

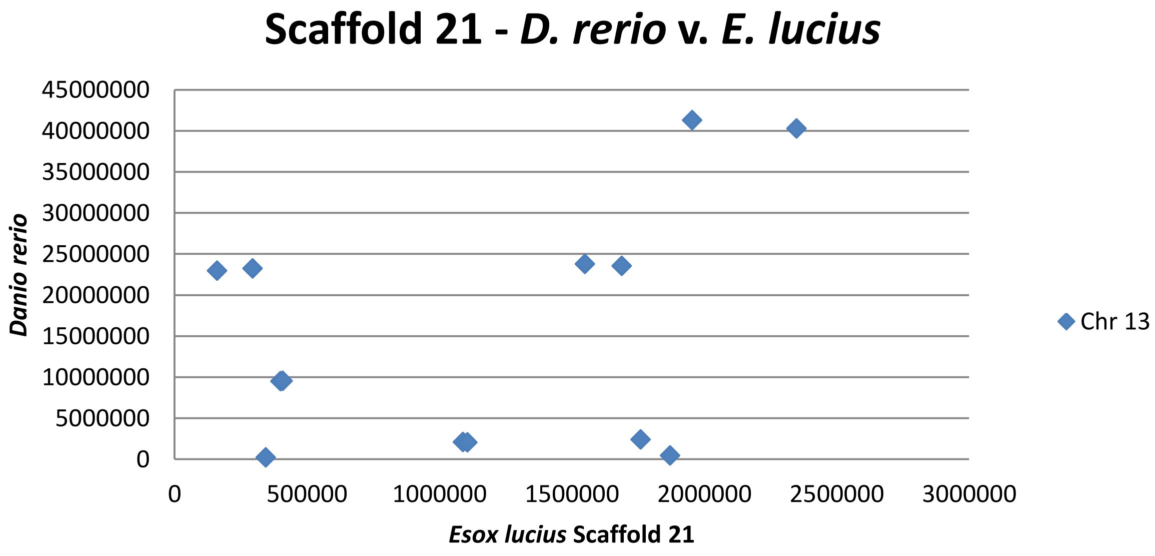
Scaffold 21 - D. rerio v. E. lucius
Danio rerio
45000000
40000000
35000000
30000000
25000000
20000000
15000000
10000000
5000000
0
0
500000
1000000
1500000
2000000
2500000
3000000
Esox lucius Scaffold 21
Chr 13

Scaffold 22

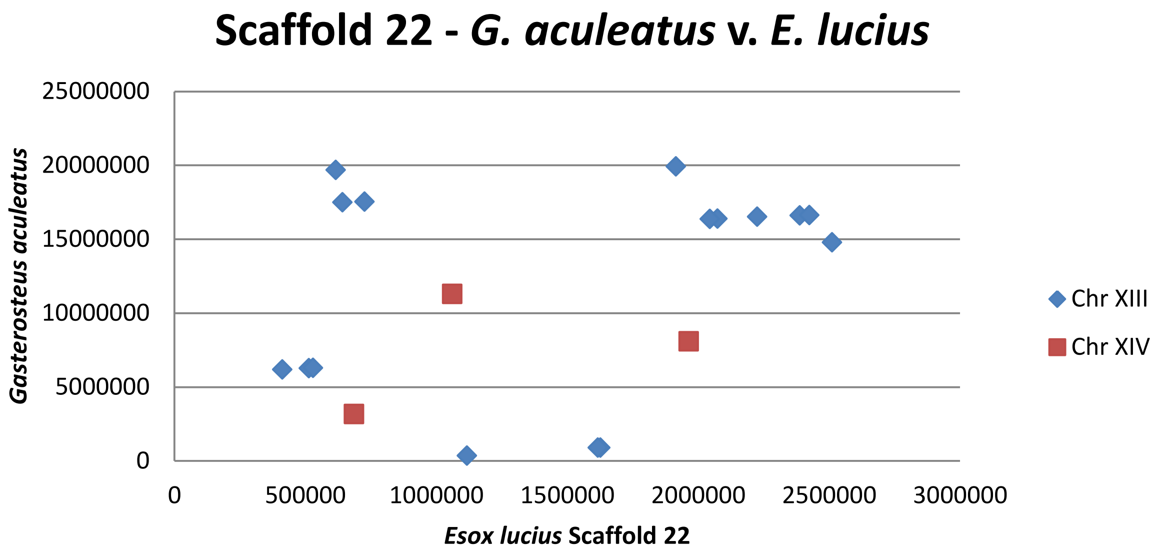
Scaffold 22 - G. aculeatus v. E. lucius
Gasterosteus aculeatus
25000000
20000000
15000000
10000000
5000000
0
0
500000
1000000
1500000
2000000
2500000
3000000
Esox lucius Scaffold 22
Chr XIII
Chr XIV

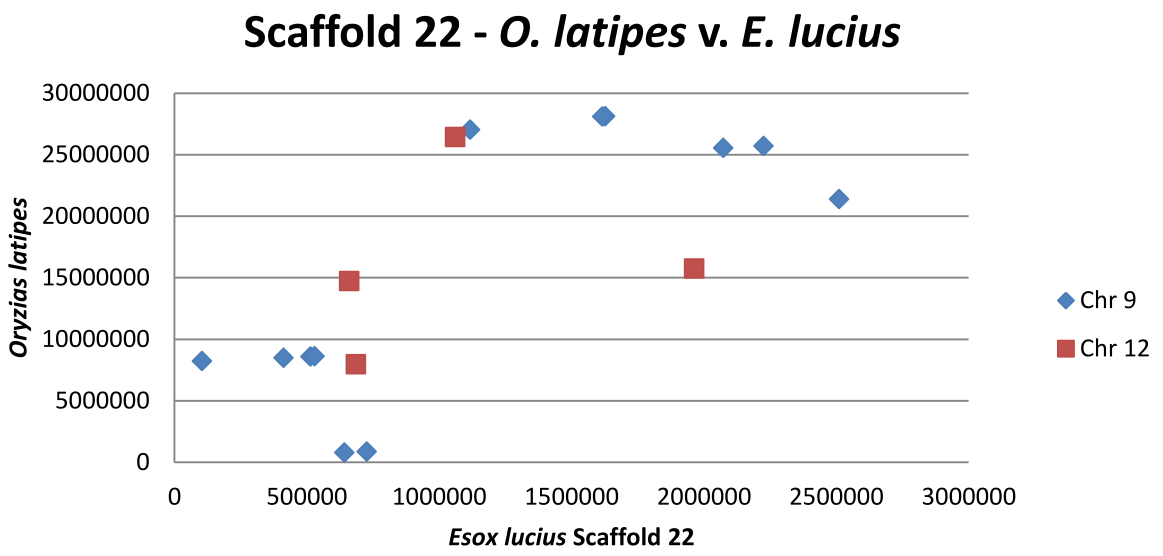
Scaffold 22 - O. latipes v. E. lucius
Oryzias latipes
30000000
25000000
20000000
15000000
10000000
5000000
0
0
500000
1000000
1500000
2000000
2500000
3000000
Esox lucius Scaffold 22
Chr 9
Chr 12

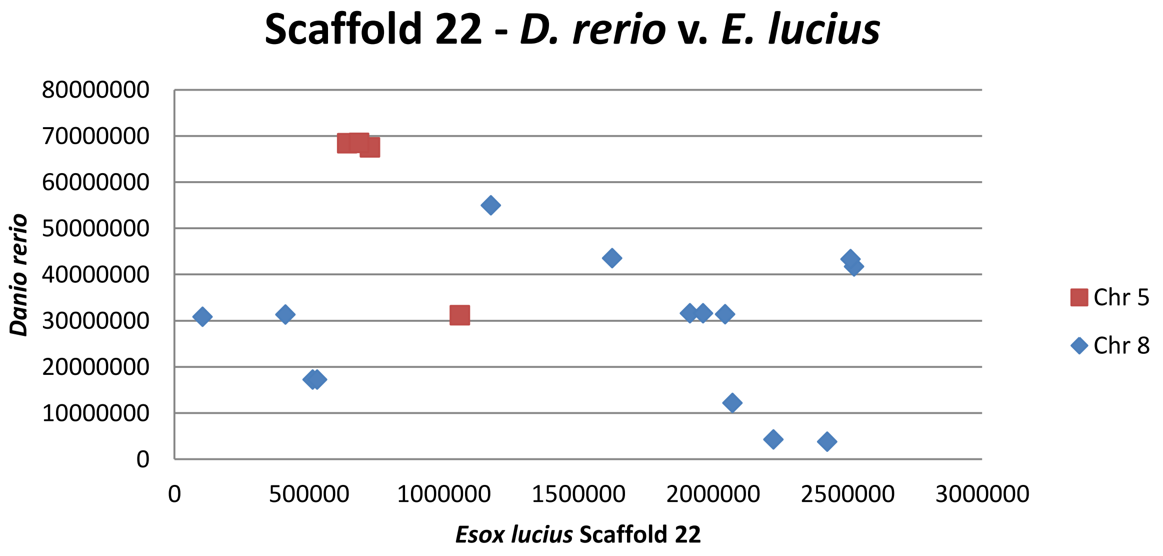
Scaffold 22 - D. rerio v. E. lucius
Danio rerio
80000000
70000000
60000000
50000000
40000000
30000000
20000000
10000000
0
0
500000
1000000
1500000
2000000
2500000
3000000
Esox lucius Scaffold 22
Chr 5
Chr 8

Scaffold 23

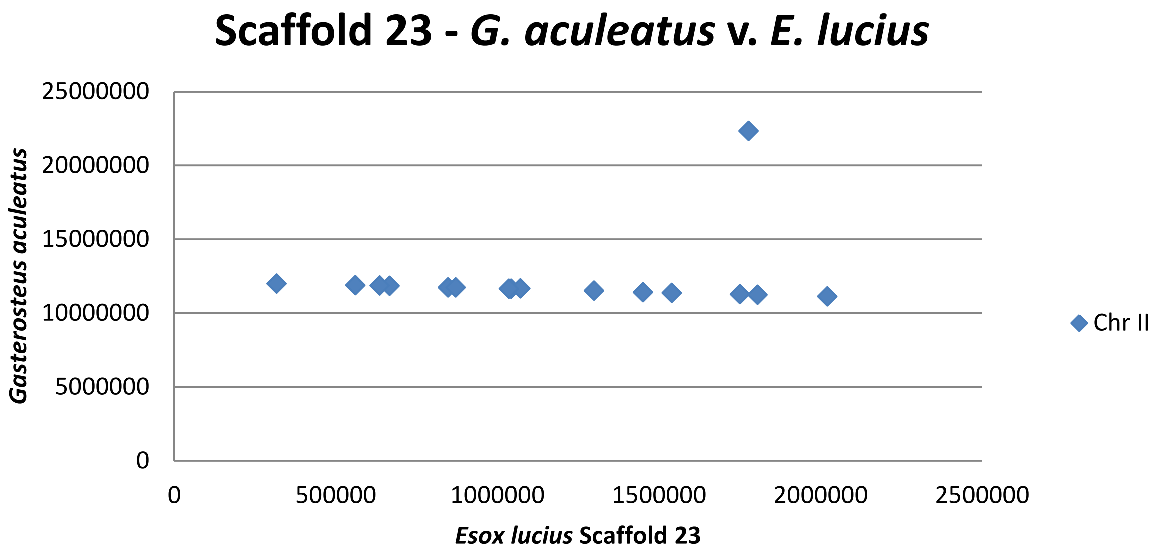

Scaffold 23 - *G. aculeatus* v. *E. lucius*
Gasterosteus aculeatus
25000000
20000000
15000000
10000000
5000000
0
0
500000
1000000
1500000
2000000
2500000
Esox lucius Scaffold 23
Chr II

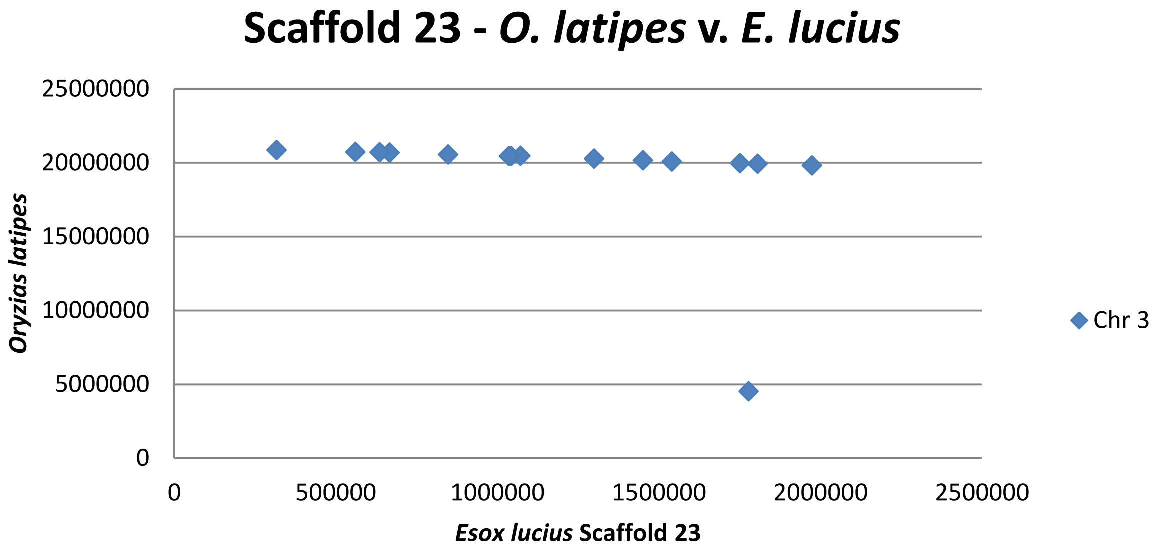

Scaffold 23 - *O. latipes* v. *E. lucius*
Oryzias latipes
25000000
20000000
15000000
10000000
5000000
0
0
500000
1000000
1500000
2000000
2500000
Esox lucius Scaffold 23
Chr 3

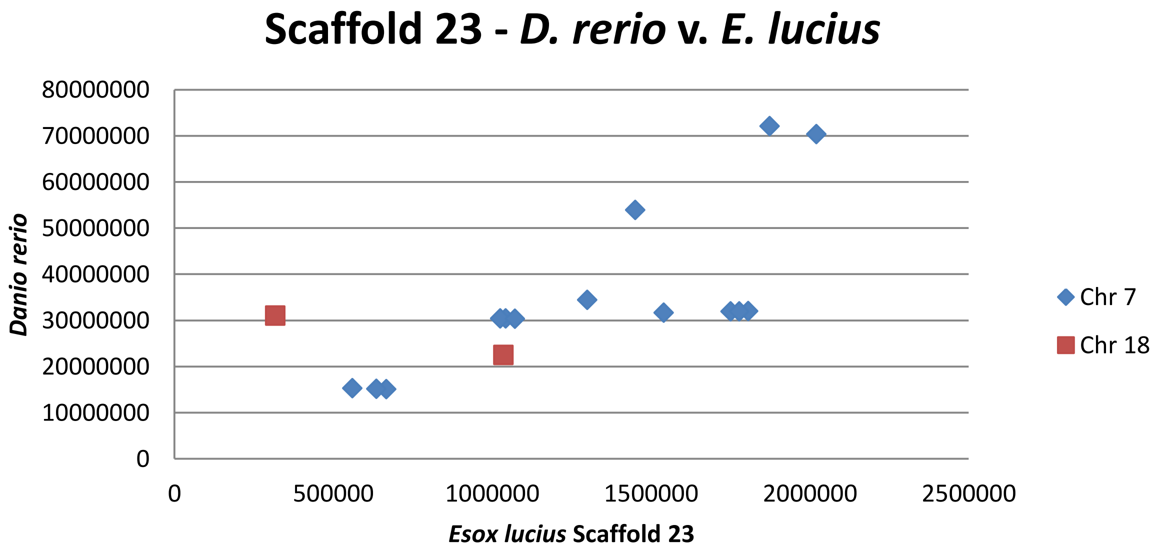

Scaffold 23 - *D. rerio* v. *E. lucius*
Danio rerio
80000000
70000000
60000000
50000000
40000000
30000000
20000000
10000000
0
0
500000
1000000
1500000
2000000
2500000
Esox lucius Scaffold 23
Chr 7
Chr 18

Scaffold 24

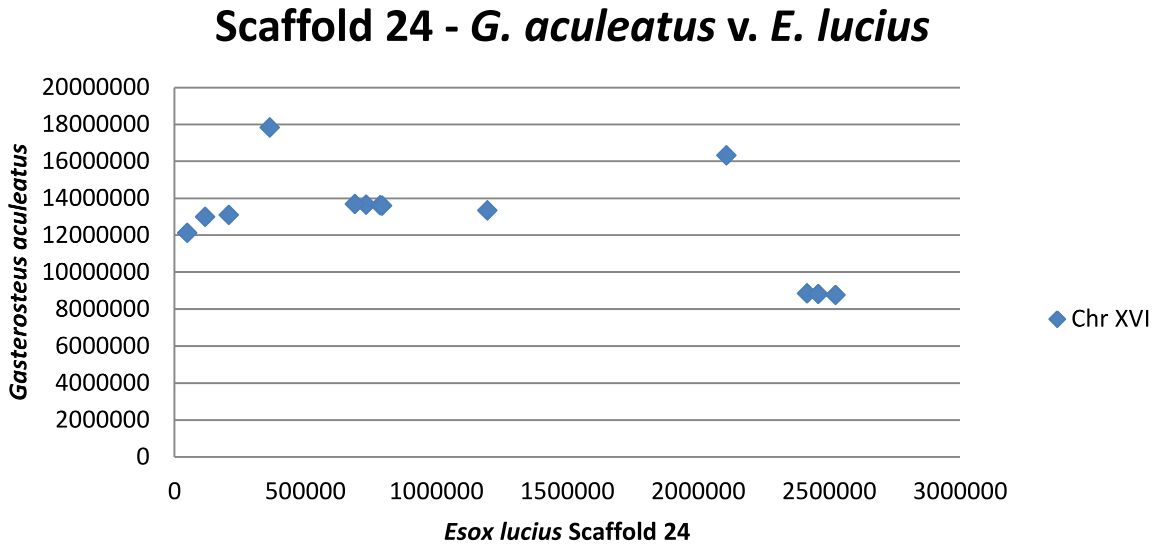

Scaffold 24 - *G. aculeatus* v. *E. lucius*
20000000
18000000
16000000
14000000
12000000
10000000
8000000
6000000
4000000
2000000
0
0
500000
1000000
1500000
2000000
2500000
3000000
Gasterosteus aculeatus
Esox lucius Scaffold 24
Chr XVI

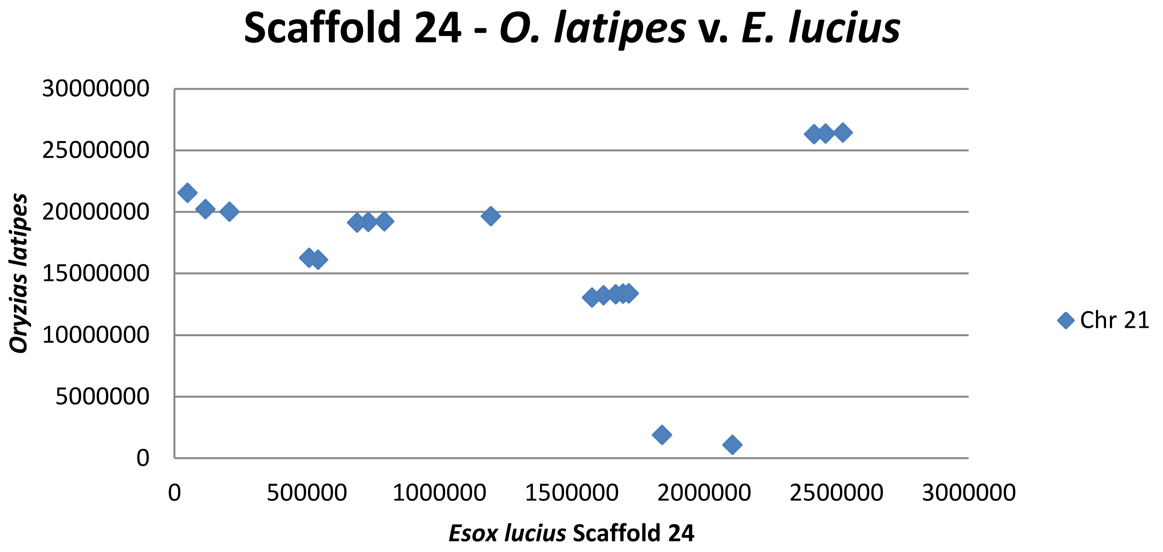

Scaffold 24 - *O. latipes* v. *E. lucius*
30000000
25000000
20000000
15000000
10000000
5000000
0
0
500000
1000000
1500000
2000000
2500000
3000000
Oryzias latipes
Esox lucius Scaffold 24
Chr 21

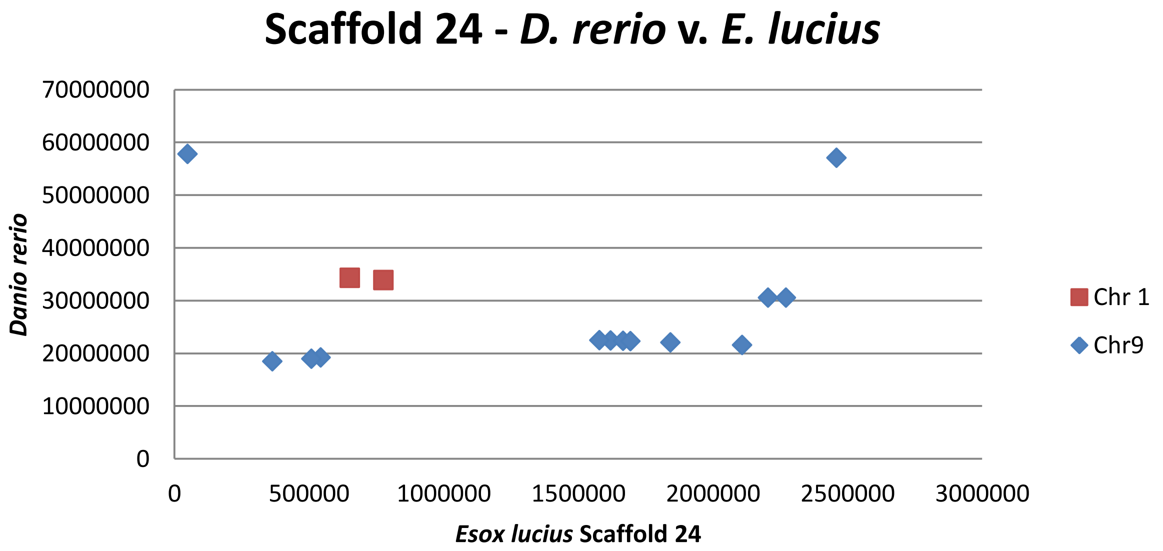

Scaffold 24 - *D. rerio* v. *E. lucius*
70000000
60000000
50000000
40000000
30000000
20000000
10000000
0
0
500000
1000000
1500000
2000000
2500000
3000000
Danio rerio
Esox lucius Scaffold 24
Chr 1
Chr9

Scaffold 25

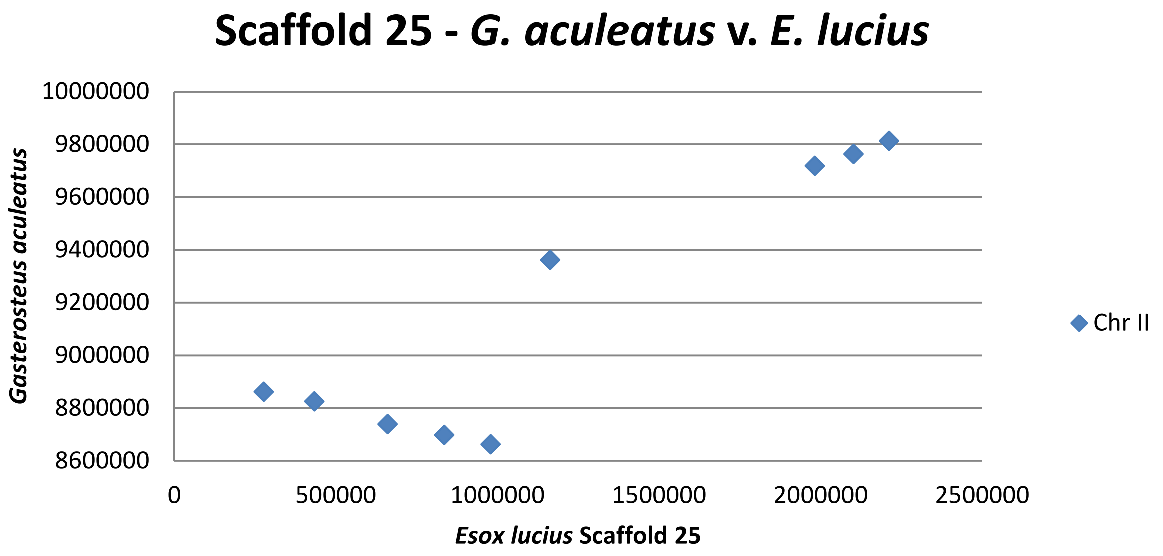
Scaffold 25 - G. aculeatus v. E. lucius
Gasterosteus aculeatus
10000000
9800000
9600000
9400000
9200000
9000000
8800000
8600000
0
500000
1000000
1500000
2000000
2500000
Esox lucius Scaffold 25
Chr II

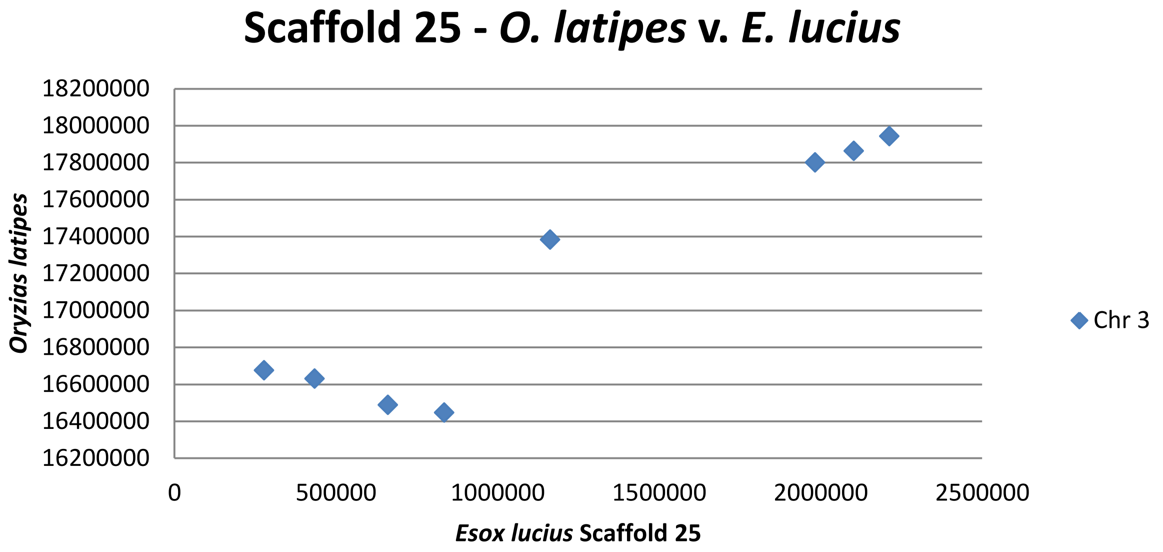
Scaffold 25 - O. latipes v. E. lucius
Oryzias latipes
18200000
18000000
17800000
17600000
17400000
17200000
17000000
16800000
16600000
16400000
16200000
0
500000
1000000
1500000
2000000
2500000
Esox lucius Scaffold 25
Chr 3

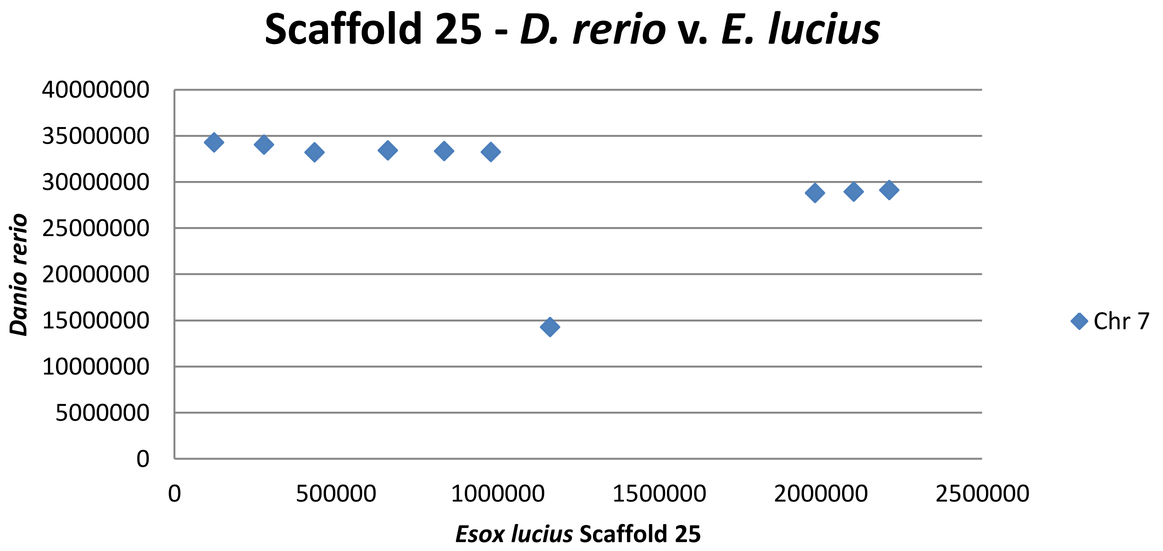
Scaffold 25 - D. rerio v. E. lucius
Danio rerio
40000000
35000000
30000000
25000000
20000000
15000000
10000000
5000000
0
0
500000
1000000
1500000
2000000
2500000
Esox lucius Scaffold 25
Chr 7

Scaffold 26

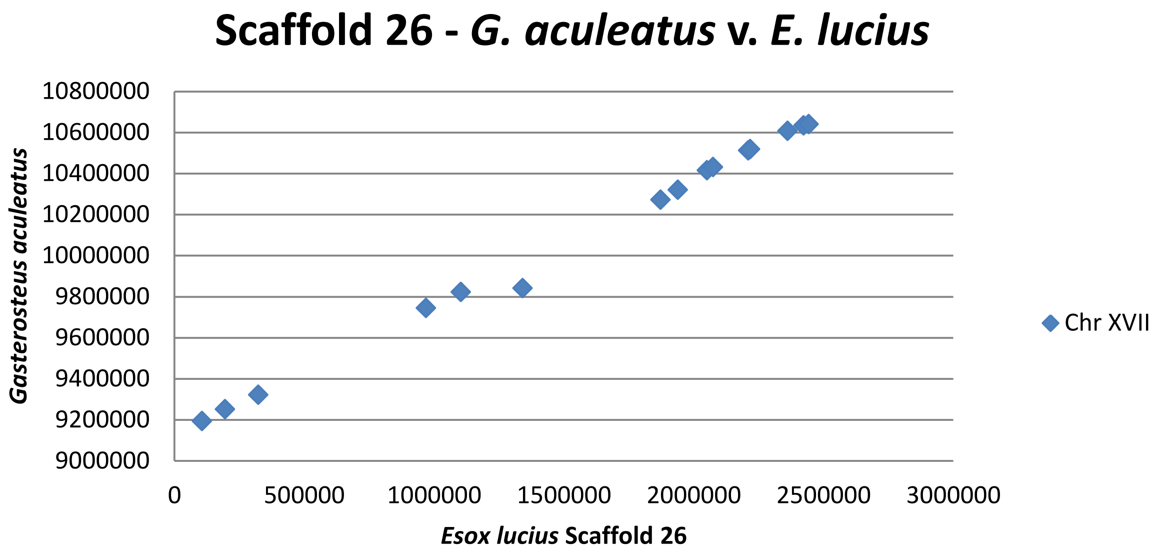
Scaffold 26 - G. aculeatus v. E. lucius
Gasterosteus aculeatus
10800000
10600000
10400000
10200000
10000000
9800000
9600000
9400000
9200000
9000000
0
500000
1000000
1500000
2000000
2500000
3000000
Esox lucius Scaffold 26
Chr XVII

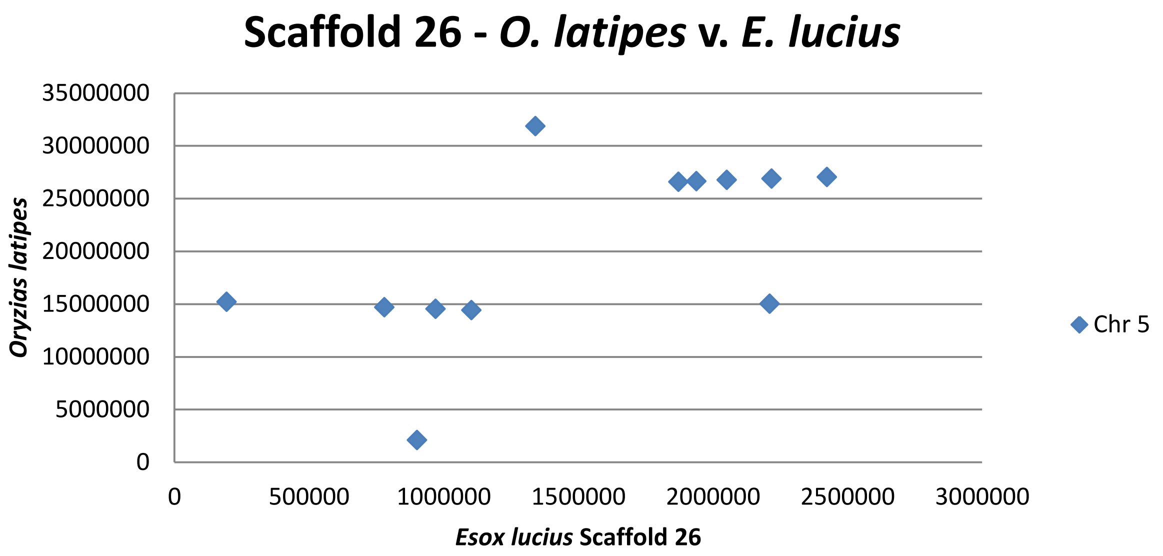
Scaffold 26 - O. latipes v. E. lucius
Oryzias latipes
35000000
30000000
25000000
20000000
15000000
10000000
5000000
0
500000
1000000
1500000
2000000
2500000
3000000
Esox lucius Scaffold 26
Chr 5

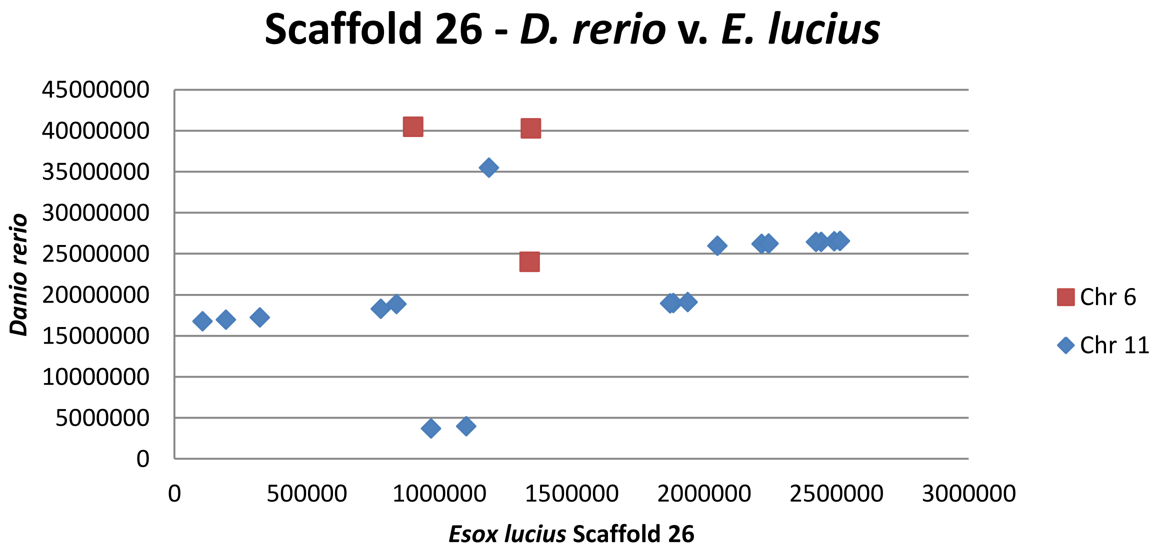
Scaffold 26 - D. rerio v. E. lucius
Danio rerio
45000000
40000000
35000000
30000000
25000000
20000000
15000000
10000000
5000000
0
500000
1000000
1500000
2000000
2500000
3000000
Esox lucius Scaffold 26
Chr 6
Chr 11

Scaffold 27

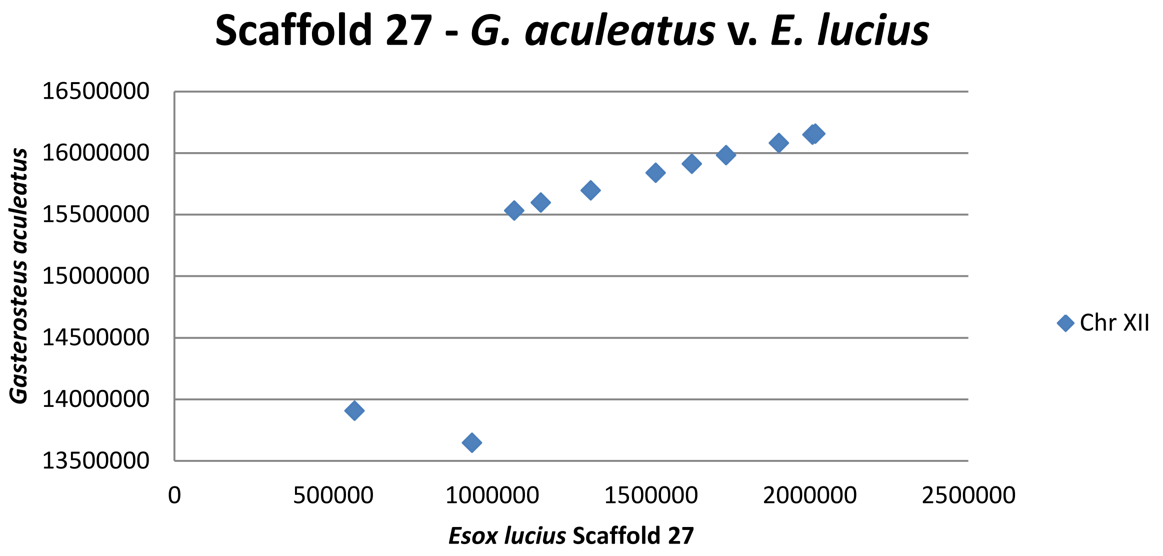
Scaffold 27 - G. aculeatus v. E. lucius
16500000
16000000
15500000
15000000
14500000
14000000
13500000
Gasterosteus aculeatus
0
500000
1000000
1500000
2000000
2500000
Esox lucius Scaffold 27
Chr XII

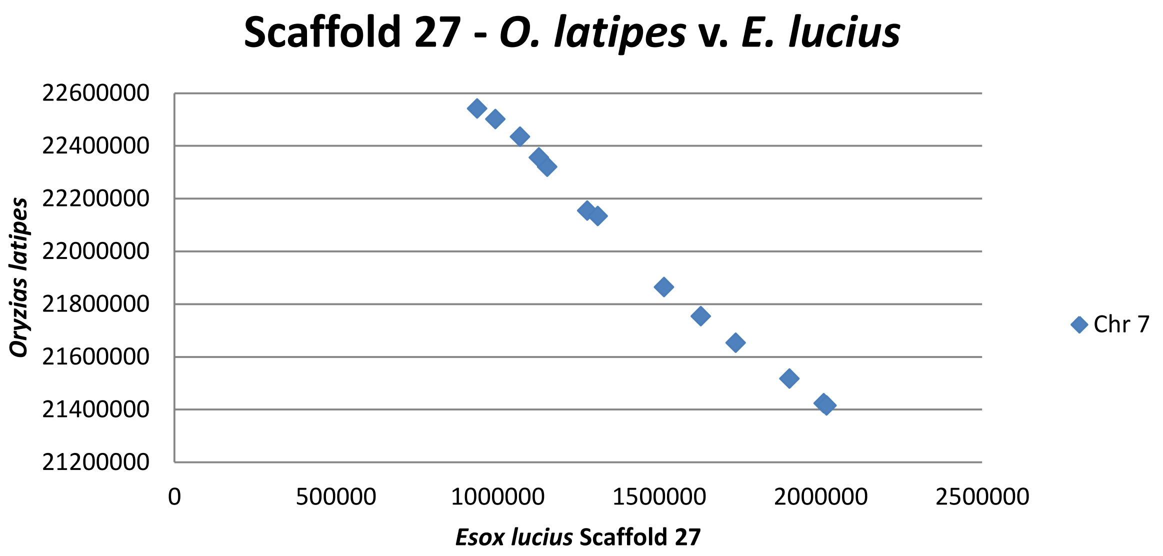
Scaffold 27 - O. latipes v. E. lucius
22600000
22400000
22200000
22000000
21800000
21600000
21400000
21200000
Oryzias latipes
0
500000
1000000
1500000
2000000
2500000
Esox lucius Scaffold 27
Chr 7

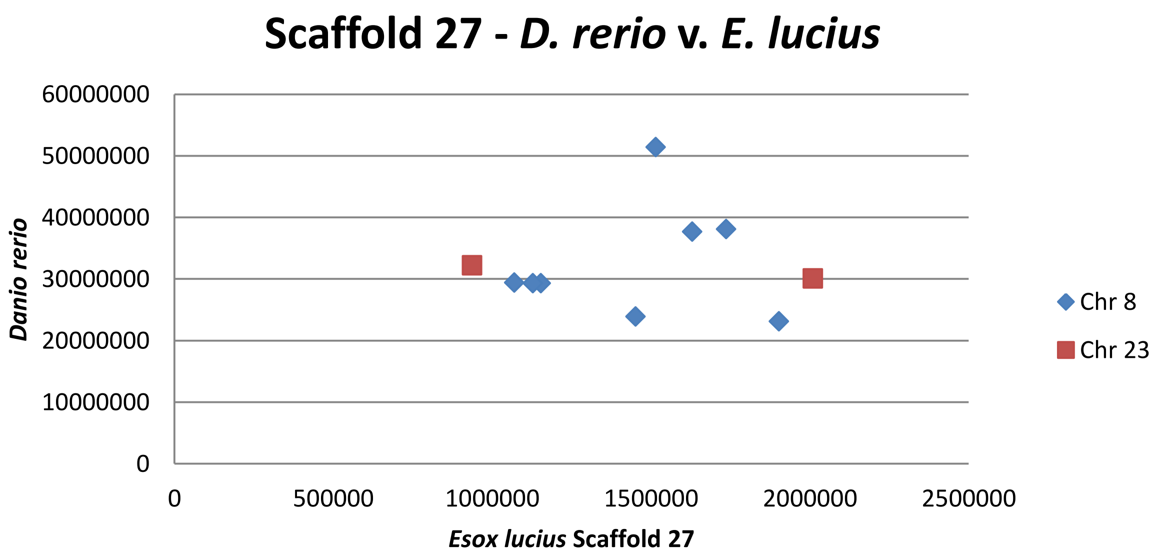
Scaffold 27 - D. rerio v. E. lucius
60000000
50000000
40000000
30000000
20000000
10000000
0
Danio rerio
0
500000
1000000
1500000
2000000
2500000
Esox lucius Scaffold 27
Chr 8
Chr 23

Scaffold 28

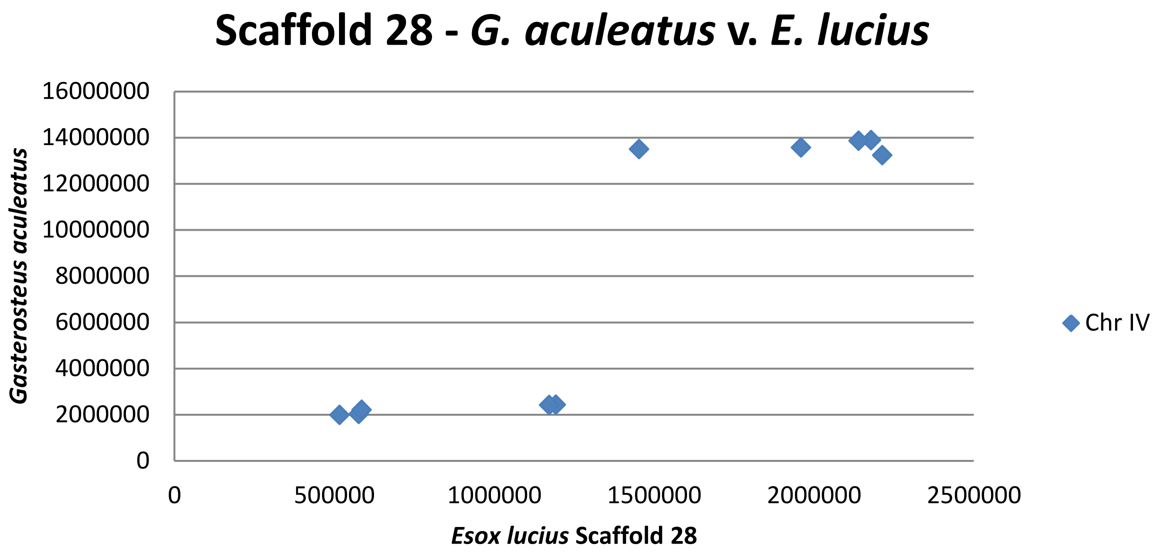

Scaffold 28 - G. aculeatus v. E. lucius
16000000
14000000
12000000
10000000
8000000
6000000
4000000
2000000
0
Gasterosteus aculeatus
0
500000
1000000
1500000
2000000
2500000
Esox lucius Scaffold 28
Chr IV

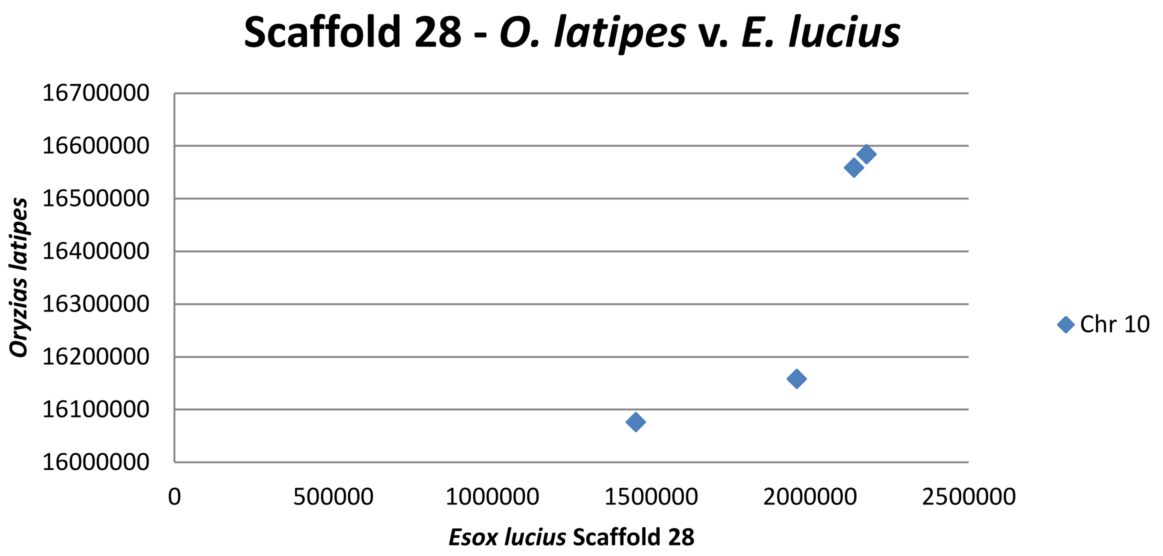

Scaffold 28 - O. latipes v. E. lucius
16700000
16600000
16500000
16400000
16300000
16200000
16100000
16000000
Oryzias latipes
0
500000
1000000
1500000
2000000
2500000
Esox lucius Scaffold 28
Chr 10

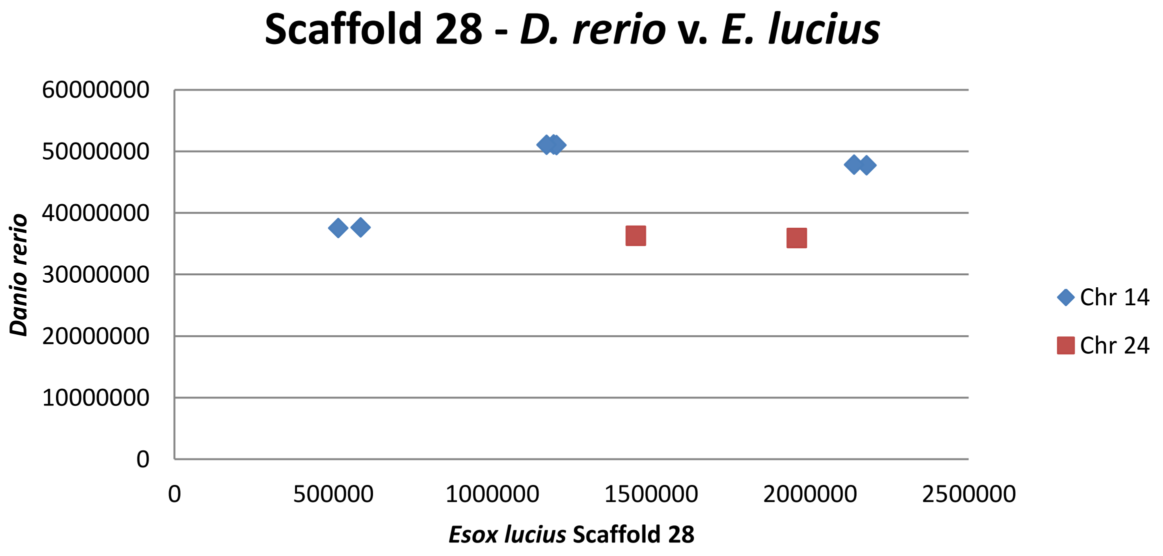

Scaffold 28 - D. rerio v. E. lucius
60000000
50000000
40000000
30000000
20000000
10000000
0
Danio rerio
0
500000
1000000
1500000
2000000
2500000
Esox lucius Scaffold 28
Chr 14
Chr 24

Scaffold 29

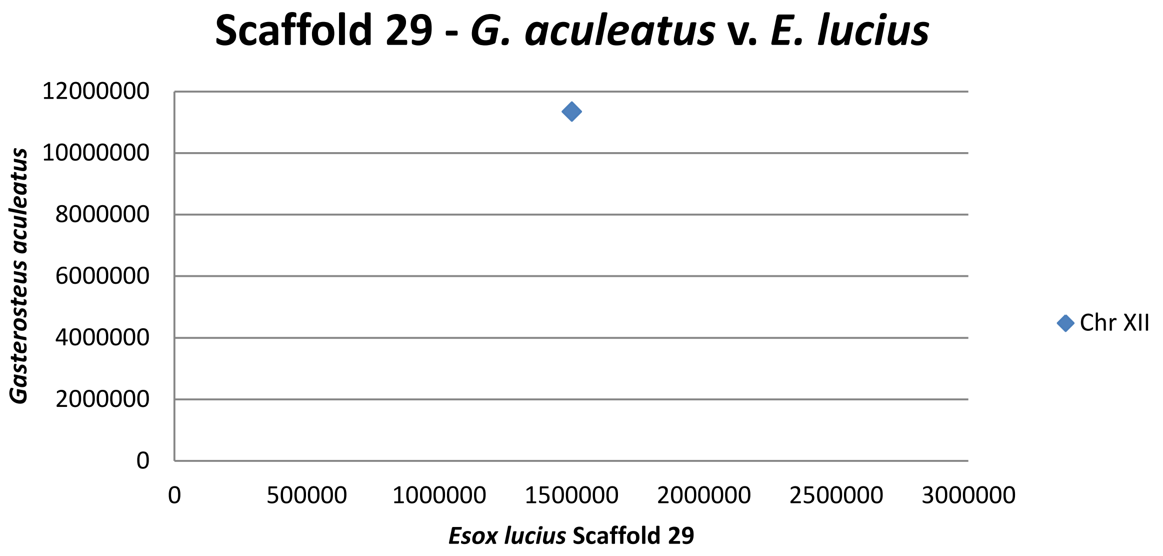

Scaffold 29 - G. aculeatus v. E. lucius
Gasterosteus aculeatus
12000000
10000000
8000000
6000000
4000000
2000000
0
0
500000
1000000
1500000
2000000
2500000
3000000
Esox lucius Scaffold 29
Chr XII

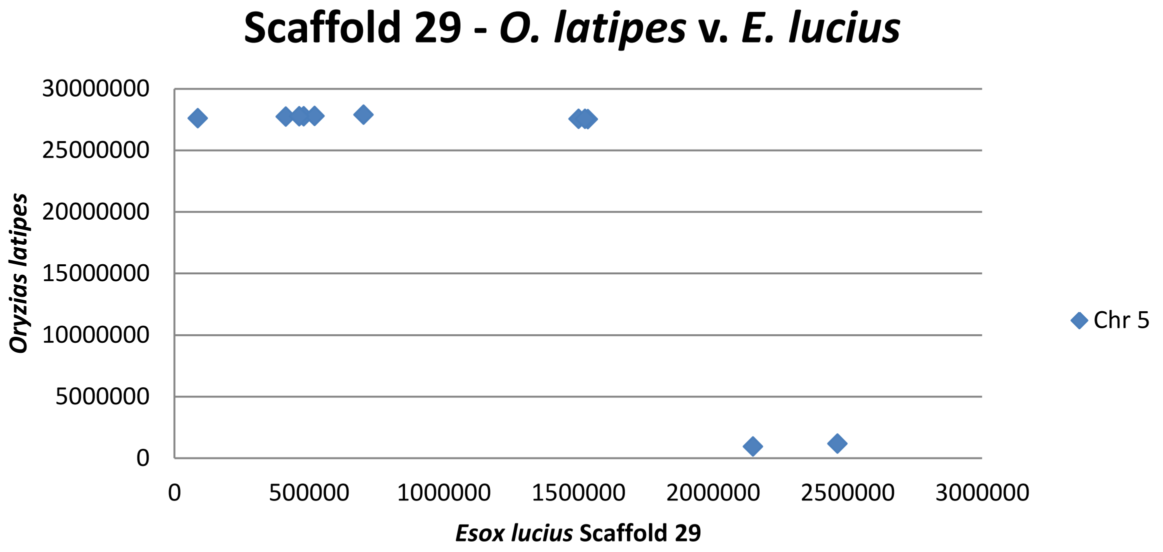

Scaffold 29 - O. latipes v. E. lucius
Oryzias latipes
30000000
25000000
20000000
15000000
10000000
5000000
0
0
500000
1000000
1500000
2000000
2500000
3000000
Esox lucius Scaffold 29
Chr 5

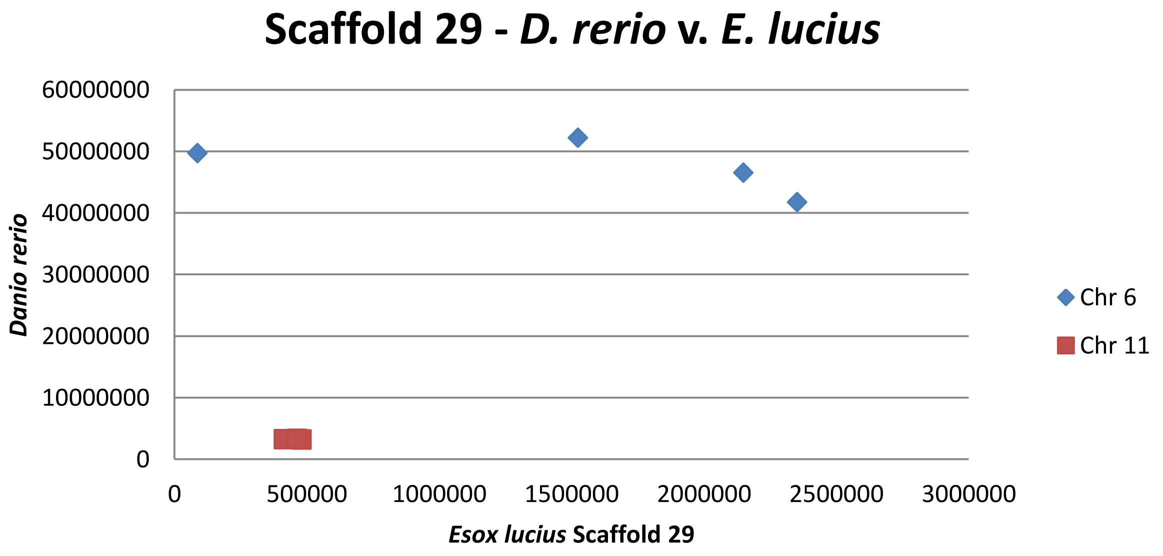

Scaffold 29 - D. rerio v. E. lucius
Danio rerio
60000000
50000000
40000000
30000000
20000000
10000000
0
0
500000
1000000
1500000
2000000
2500000
3000000
Esox lucius Scaffold 29
Chr 6
Chr 11

Scaffold 30

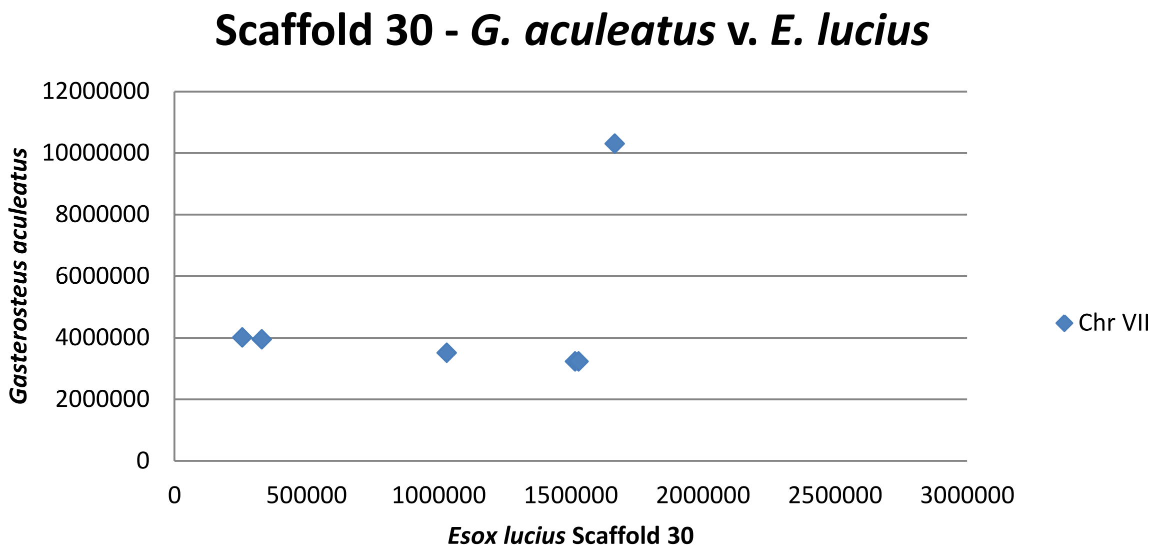

Scaffold 30 - G. aculeatus v. E. lucius
12000000
10000000
8000000
6000000
4000000
2000000
0
Gasterosteus aculeatus
0
500000
1000000
1500000
2000000
2500000
3000000
Esox lucius Scaffold 30
Chr VII

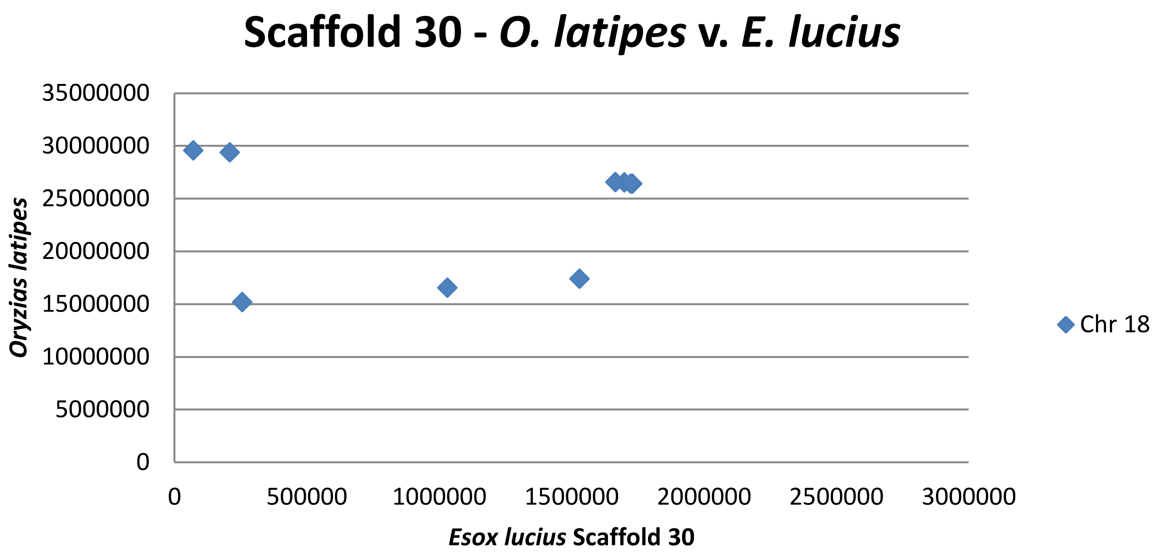

Scaffold 30 - O. latipes v. E. lucius
35000000
30000000
25000000
20000000
15000000
10000000
5000000
0
Oryzias latipes
0
500000
1000000
1500000
2000000
2500000
3000000
Esox lucius Scaffold 30
Chr 18

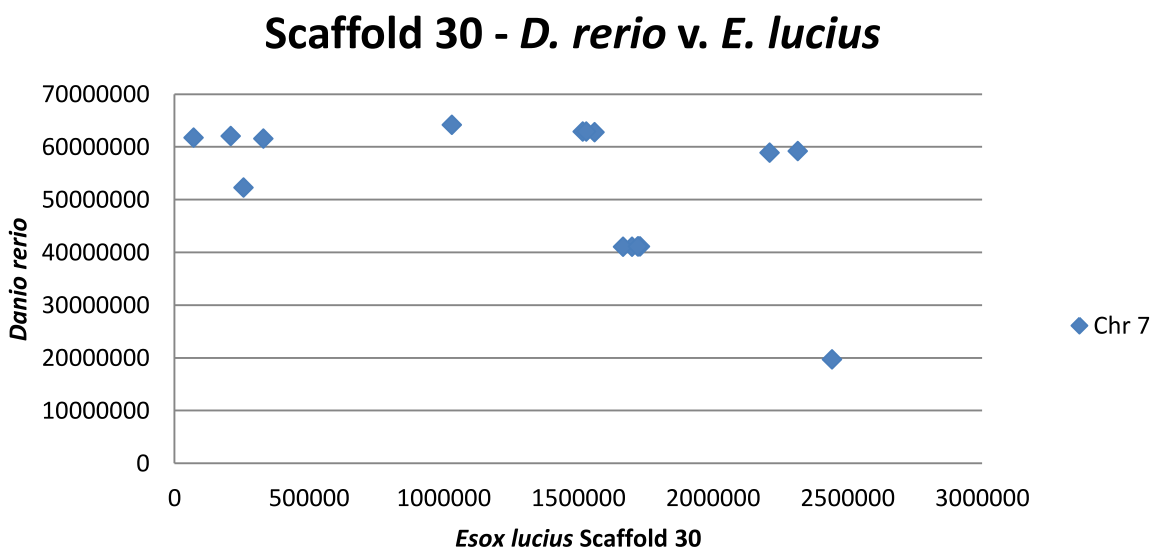

Scaffold 30 - D. rerio v. E. lucius
70000000
60000000
50000000
40000000
30000000
20000000
10000000
0
Danio rerio
0
500000
1000000
1500000
2000000
2500000
3000000
Esox lucius Scaffold 30
Chr 7

Scaffold 31

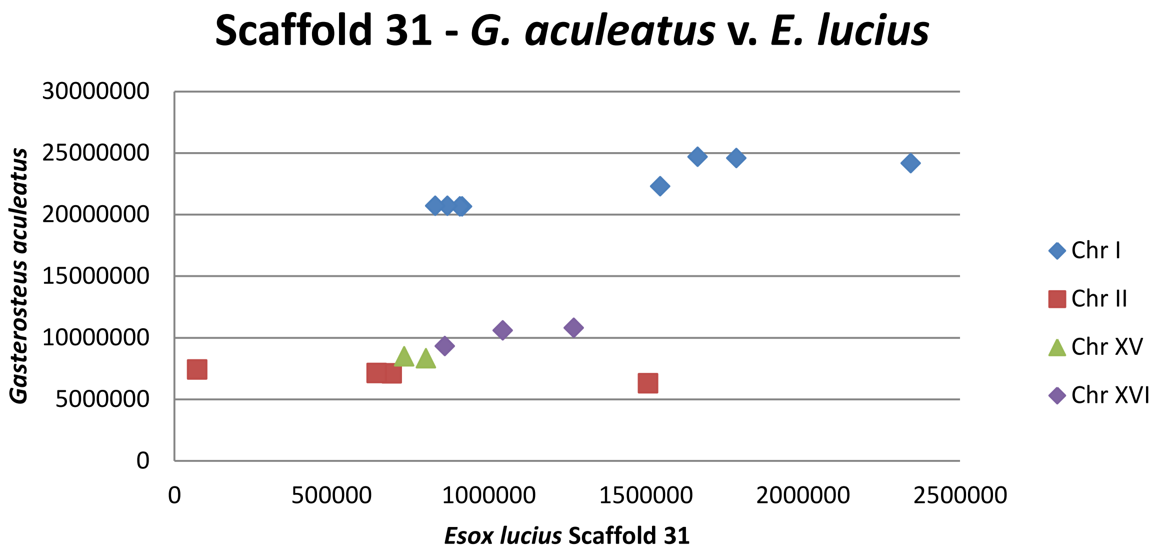

Scaffold 31 - G. aculeatus v. E. lucius
30000000
25000000
20000000
15000000
10000000
5000000
0
Gasterosteus aculeatus
0
500000
1000000
1500000
2000000
2500000
Esox lucius Scaffold 31
Chr I
Chr II
Chr XV
Chr XVI

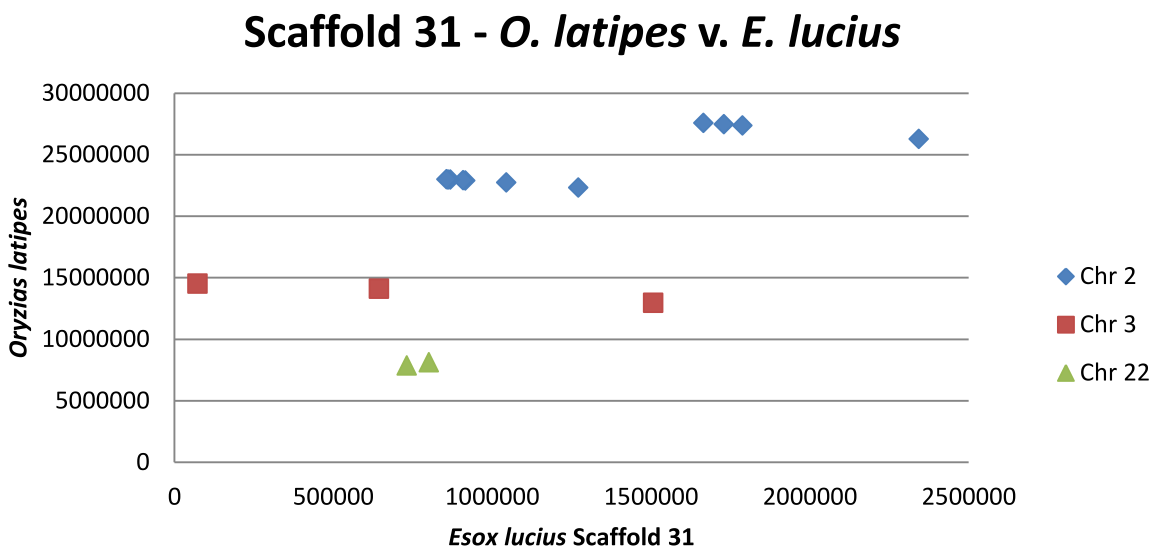

Scaffold 31 - O. latipes v. E. lucius
30000000
25000000
20000000
15000000
10000000
5000000
0
Oryzias latipes
0
500000
1000000
1500000
2000000
2500000
Esox lucius Scaffold 31
Chr 2
Chr 3
Chr 22

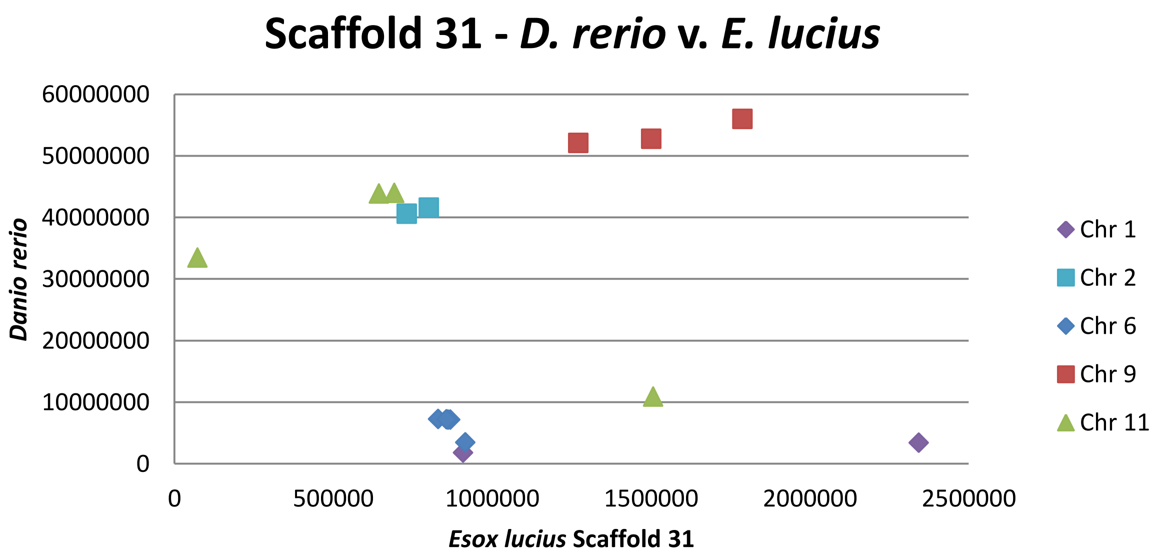

Scaffold 31 - D. rerio v. E. lucius
60000000
50000000
40000000
30000000
20000000
10000000
0
Danio rerio
0
500000
1000000
1500000
2000000
2500000
Esox lucius Scaffold 31
Chr 1
Chr 2
Chr 6
Chr 9
Chr 11

Scaffold 32

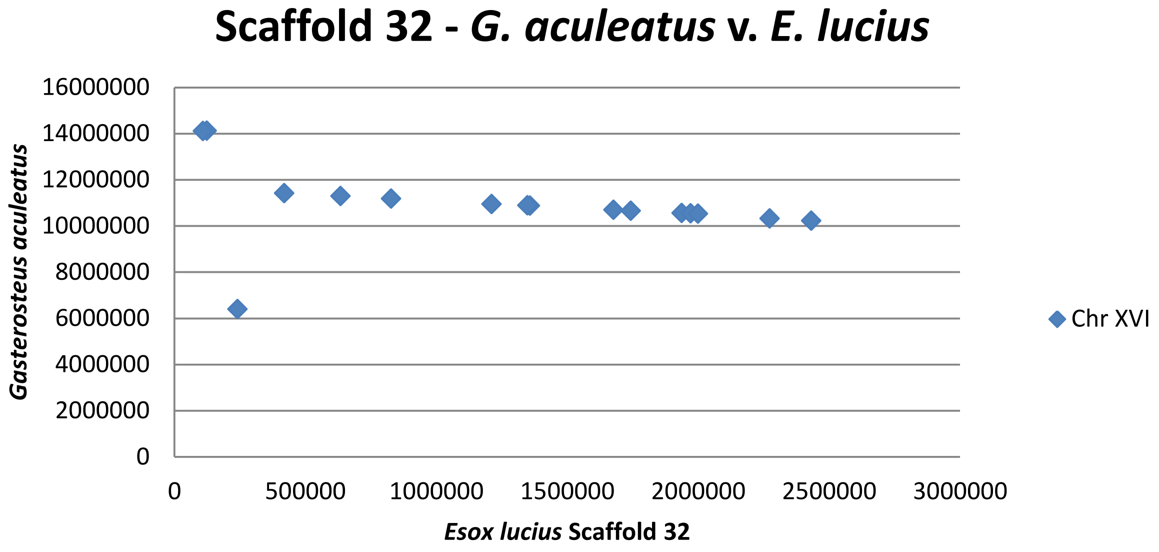
Scaffold 32 - G. aculeatus v. E. lucius
Gasterosteus aculeatus
16000000
14000000
12000000
10000000
8000000
6000000
4000000
2000000
0
0
500000
1000000
1500000
2000000
2500000
3000000
Esox lucius Scaffold 32
Chr XVI

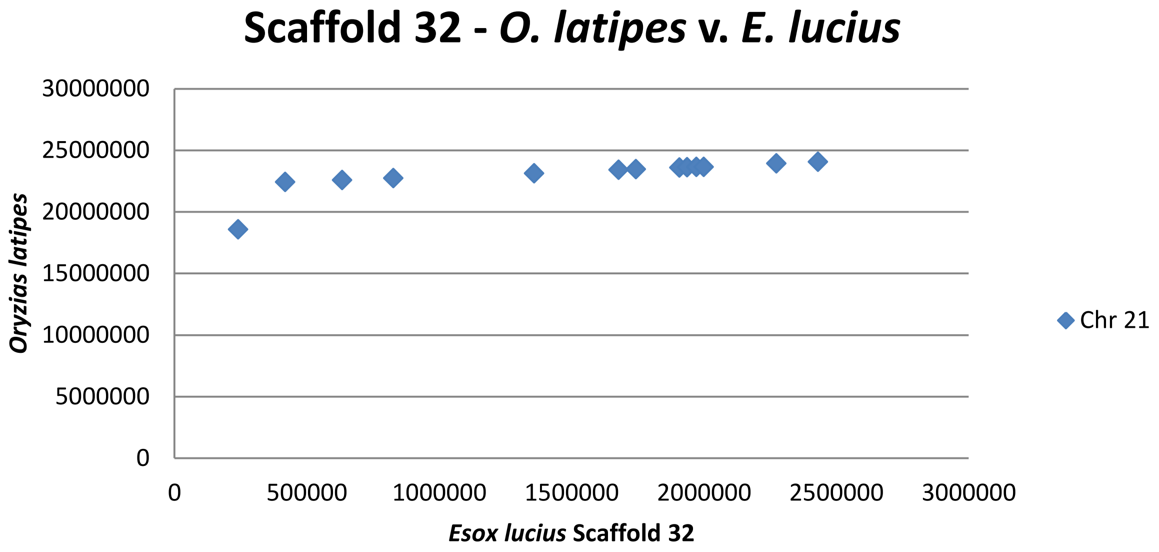
Scaffold 32 - O. latipes v. E. lucius
Oryzias latipes
30000000
25000000
20000000
15000000
10000000
5000000
0
0
500000
1000000
1500000
2000000
2500000
3000000
Esox lucius Scaffold 32
Chr 21

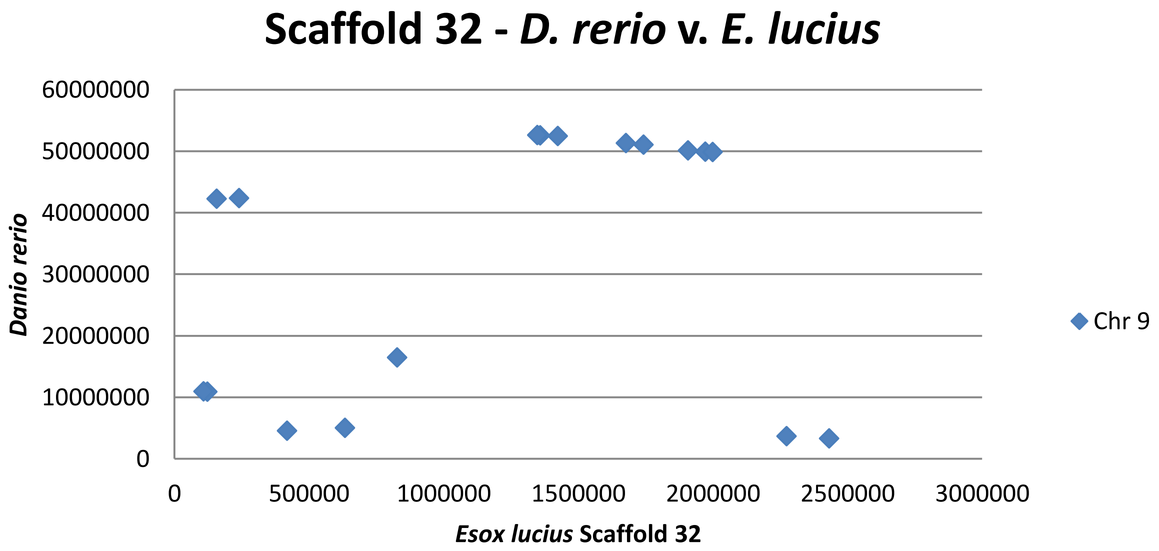
Scaffold 32 - D. rerio v. E. lucius
Danio rerio
60000000
50000000
40000000
30000000
20000000
10000000
0
0
500000
1000000
1500000
2000000
2500000
3000000
Esox lucius Scaffold 32
Chr 9

Scaffold 33

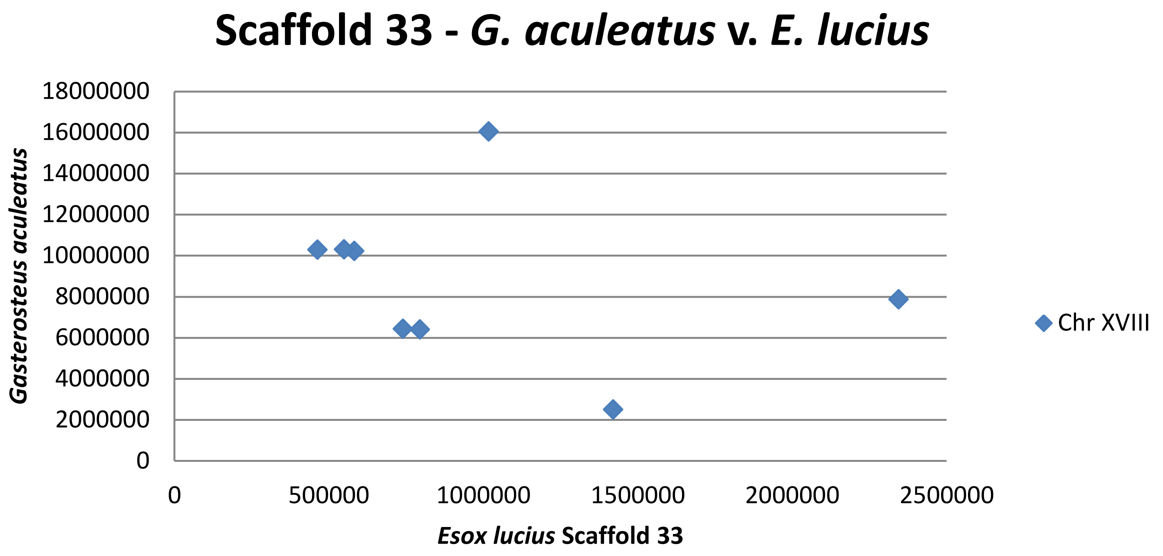

Scaffold 33 - *G. aculeatus* v. *E. lucius*
Gasterosteus aculeatus
18000000
16000000
14000000
12000000
10000000
8000000
6000000
4000000
2000000
0
0
500000
1000000
1500000
2000000
2500000
Esox lucius Scaffold 33
Chr XVIII

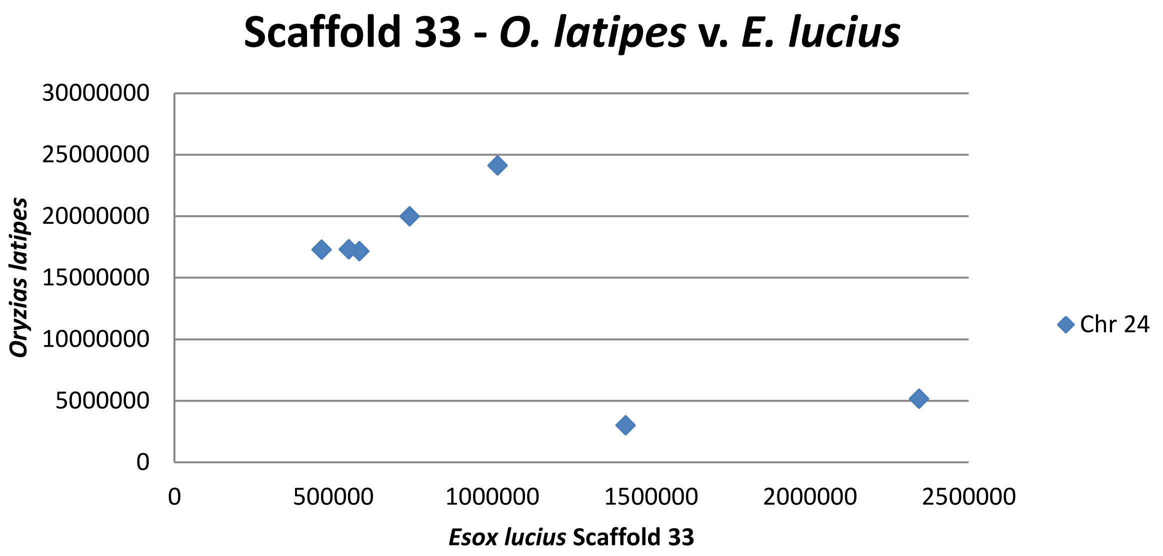

Scaffold 33 - *O. latipes* v. *E. lucius*
Oryzias latipes
30000000
25000000
20000000
15000000
10000000
5000000
0
0
500000
1000000
1500000
2000000
2500000
Esox lucius Scaffold 33
Chr 24

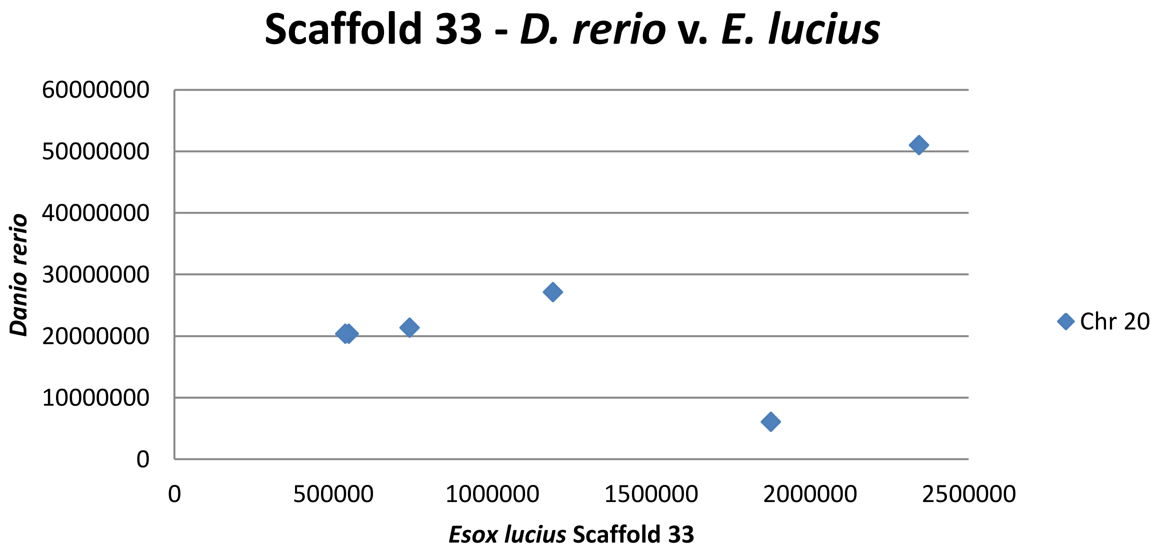

Scaffold 33 - *D. rerio* v. *E. lucius*
Danio rerio
60000000
50000000
40000000
30000000
20000000
10000000
0
0
500000
1000000
1500000
2000000
2500000
Esox lucius Scaffold 33
Chr 20

Scaffold 34

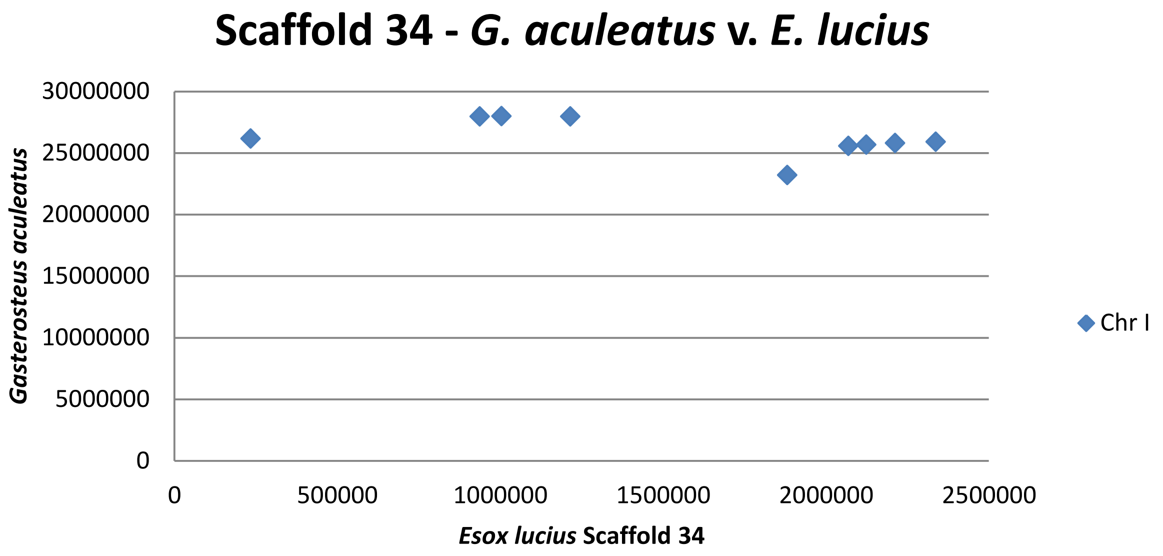

Scaffold 34 - G. aculeatus v. E. lucius
30000000
25000000
20000000
15000000
10000000
5000000
0
Gasterosteus aculeatus
0
500000
1000000
1500000
2000000
2500000
Esox lucius Scaffold 34
Chr I

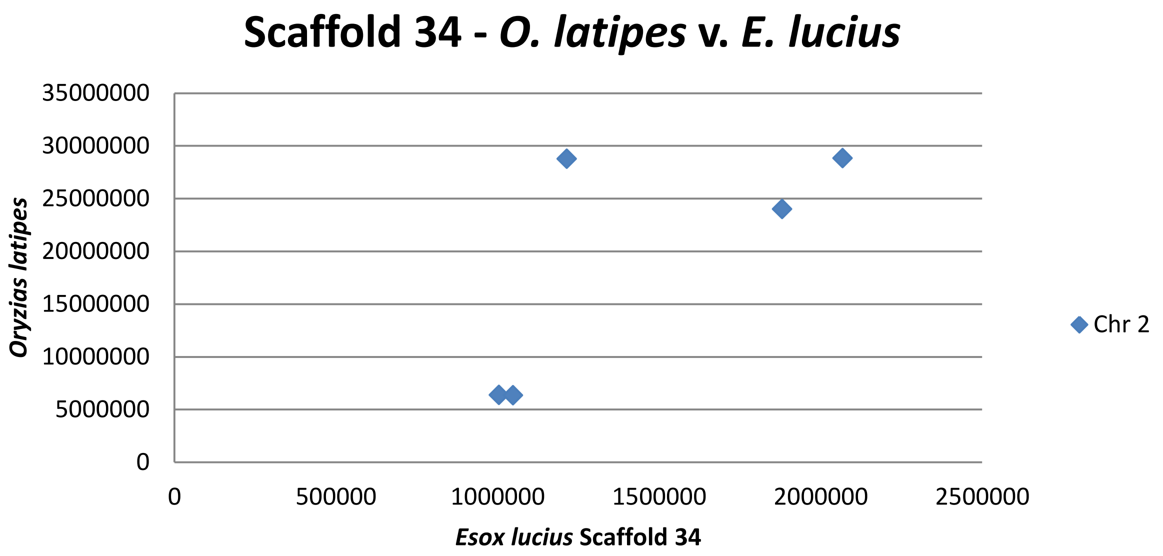

Scaffold 34 - O. latipes v. E. lucius
35000000
30000000
25000000
20000000
15000000
10000000
5000000
0
Oryzias latipes
0
500000
1000000
1500000
2000000
2500000
Esox lucius Scaffold 34
Chr 2

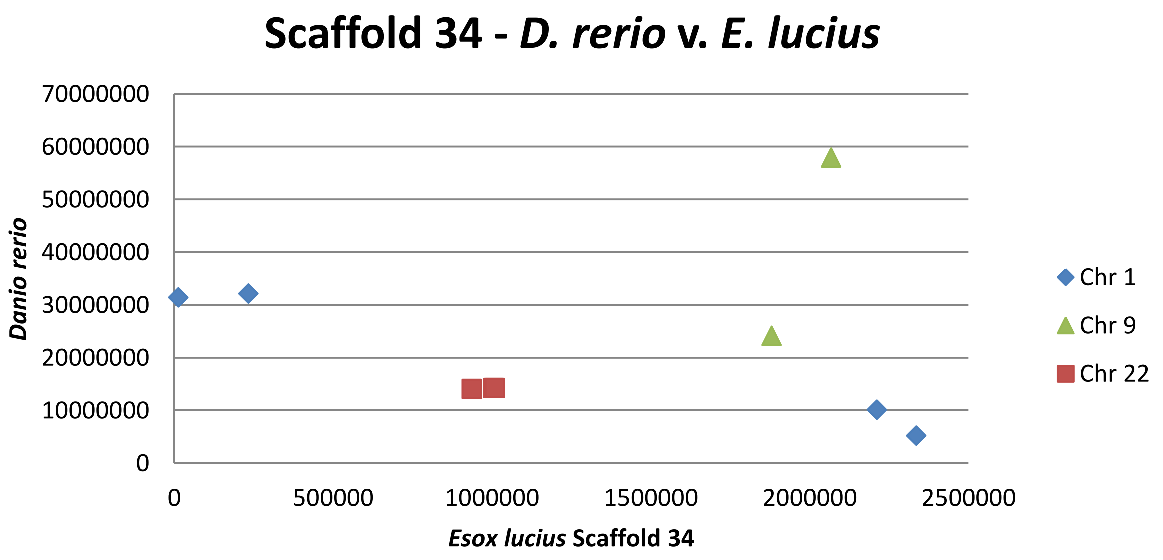

Scaffold 34 - D. rerio v. E. lucius
70000000
60000000
50000000
40000000
30000000
20000000
10000000
0
Danio rerio
0
500000
1000000
1500000
2000000
2500000
Esox lucius Scaffold 34
Chr 1
Chr 9
Chr 22

Scaffold 35

Scaffold 35 - *G. aculeatus* v. *E. lucius*

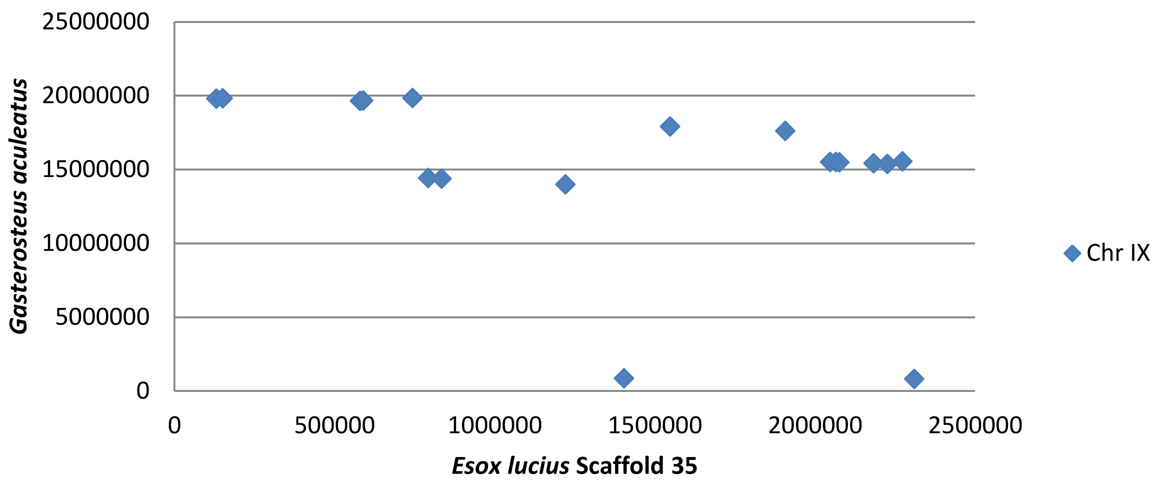

Scaffold 35 - *O. latipes* v. *E. lucius*

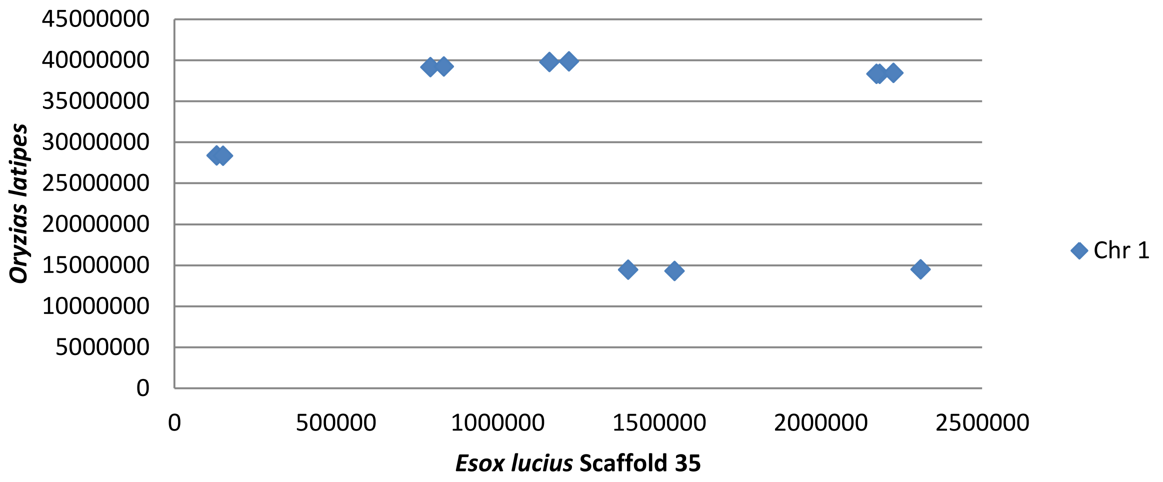

Scaffold 35 - *D. rerio* v. *E. lucius*

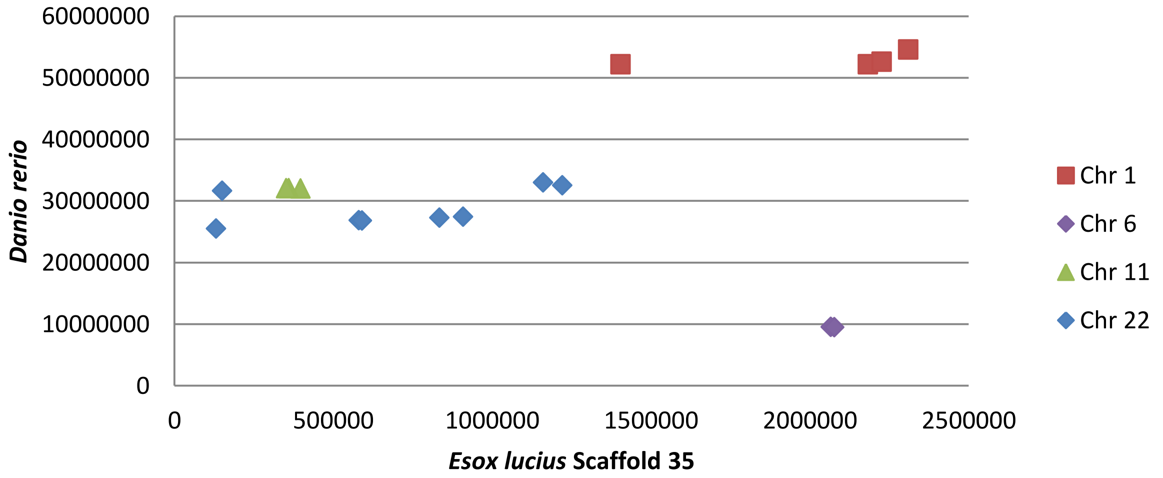

Scaffold 36

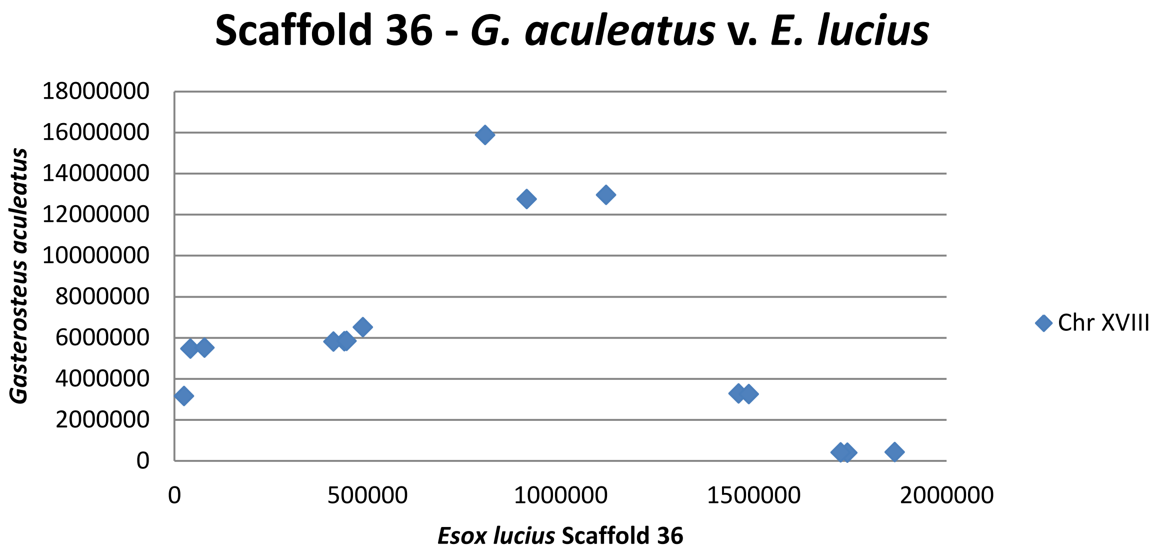
Scaffold 36 - *G. aculeatus* v. *E. lucius*
18000000
16000000
14000000
12000000
10000000
8000000
6000000
4000000
2000000
0
Gasterosteus aculeatus
0
500000
1000000
1500000
2000000
Esox lucius Scaffold 36
Chr XVIII

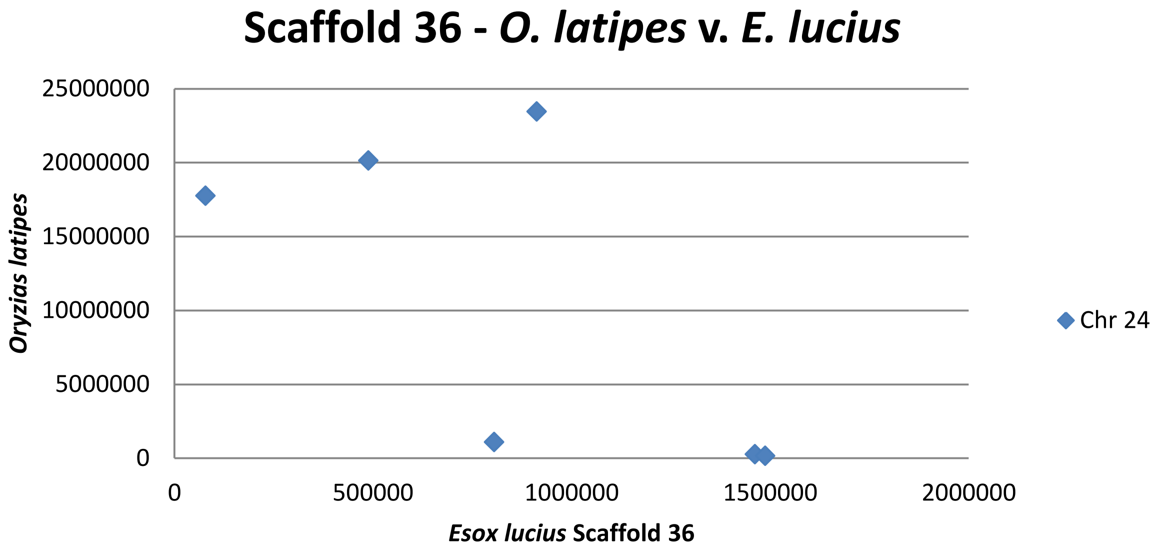
Scaffold 36 - *O. latipes* v. *E. lucius*
25000000
20000000
15000000
10000000
5000000
0
Oryzias latipes
0
500000
1000000
1500000
2000000
Esox lucius Scaffold 36
Chr 24

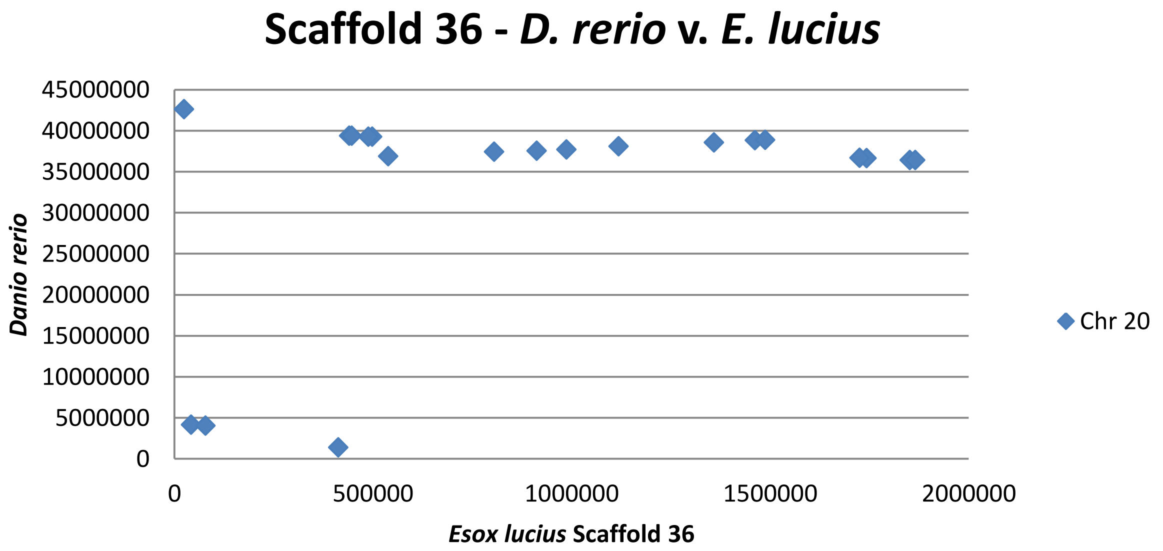
Scaffold 36 - *D. rerio* v. *E. lucius*
45000000
40000000
35000000
30000000
25000000
20000000
15000000
10000000
5000000
0
Danio rerio
0
500000
1000000
1500000
2000000
Esox lucius Scaffold 36
Chr 20

Scaffold 37

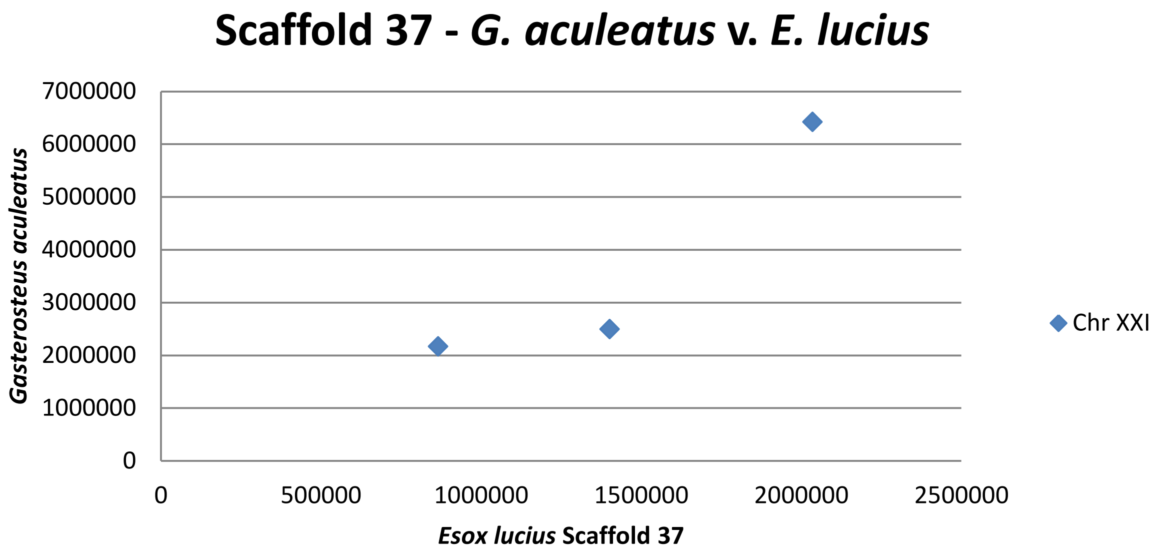
Scaffold 37 - G. aculeatus v. E. lucius
Gasterosteus aculeatus
7000000
6000000
5000000
4000000
3000000
2000000
1000000
0
0
500000
1000000
1500000
2000000
2500000
Esox lucius Scaffold 37
Chr XXI

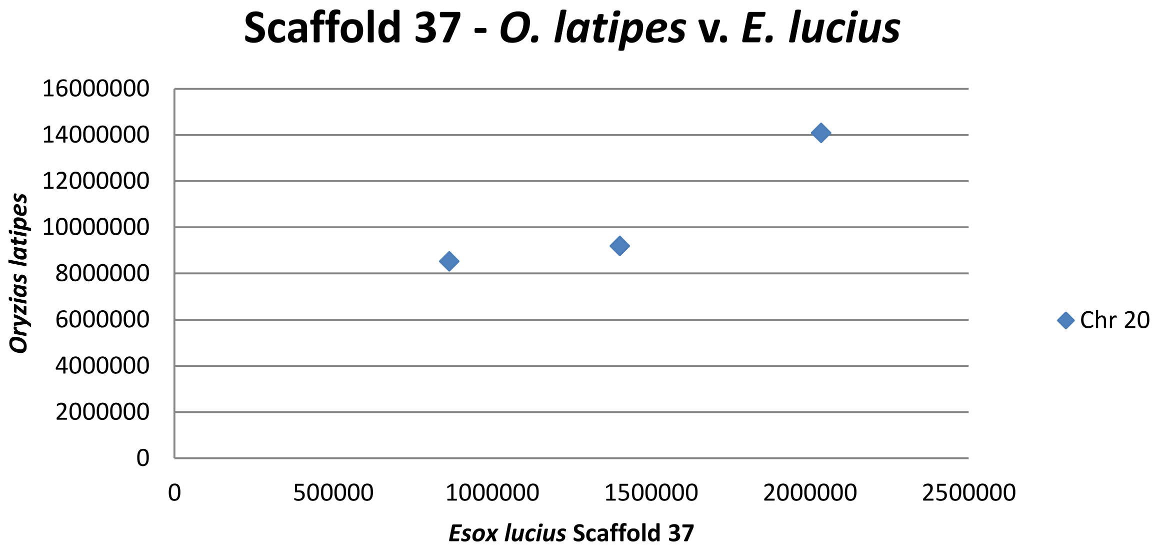
Scaffold 37 - O. latipes v. E. lucius
Oryzias latipes
16000000
14000000
12000000
10000000
8000000
6000000
4000000
2000000
0
0
500000
1000000
1500000
2000000
2500000
Esox lucius Scaffold 37
Chr 20

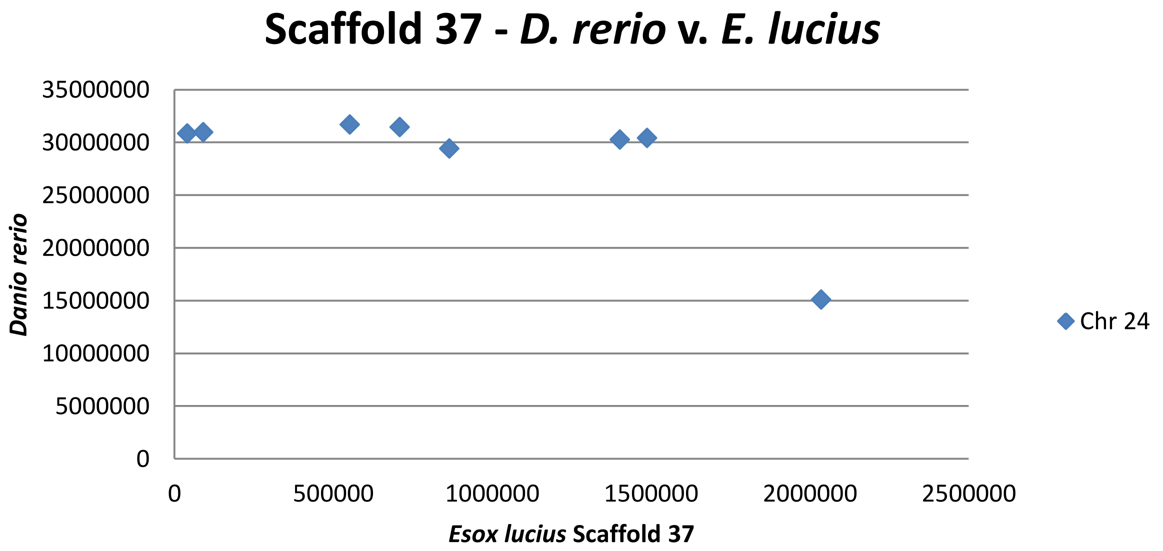
Scaffold 37 - D. rerio v. E. lucius
Danio rerio
35000000
30000000
25000000
20000000
15000000
10000000
5000000
0
0
500000
1000000
1500000
2000000
2500000
Esox lucius Scaffold 37
Chr 24

Scaffold 38

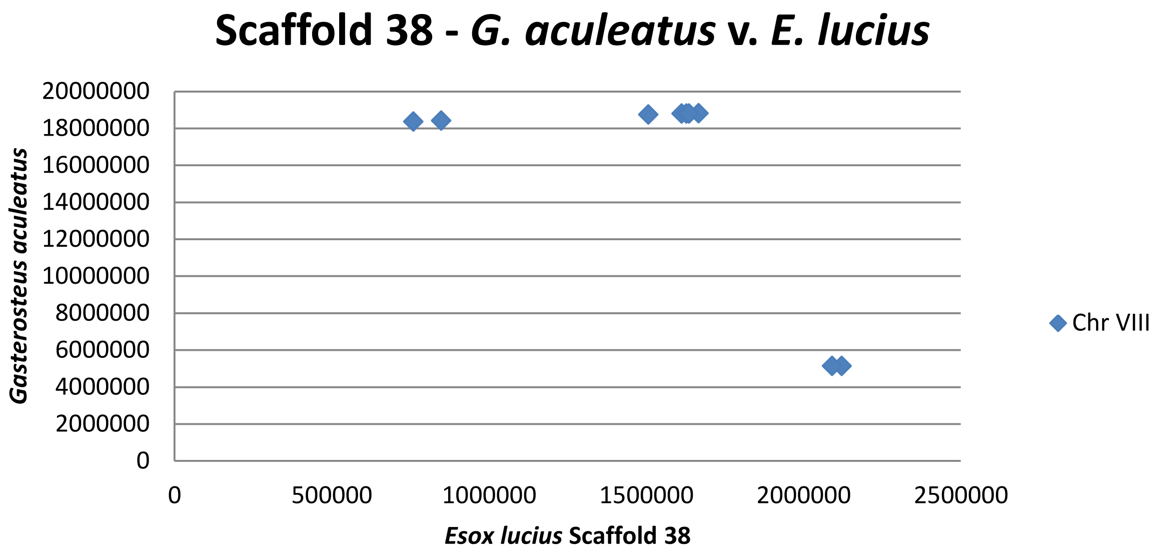

Scaffold 38 - G. aculeatus v. E. lucius
Gasterosteus aculeatus
20000000
18000000
16000000
14000000
12000000
10000000
8000000
6000000
4000000
2000000
0
0
500000
1000000
1500000
2000000
2500000
Esox lucius Scaffold 38
Chr VIII

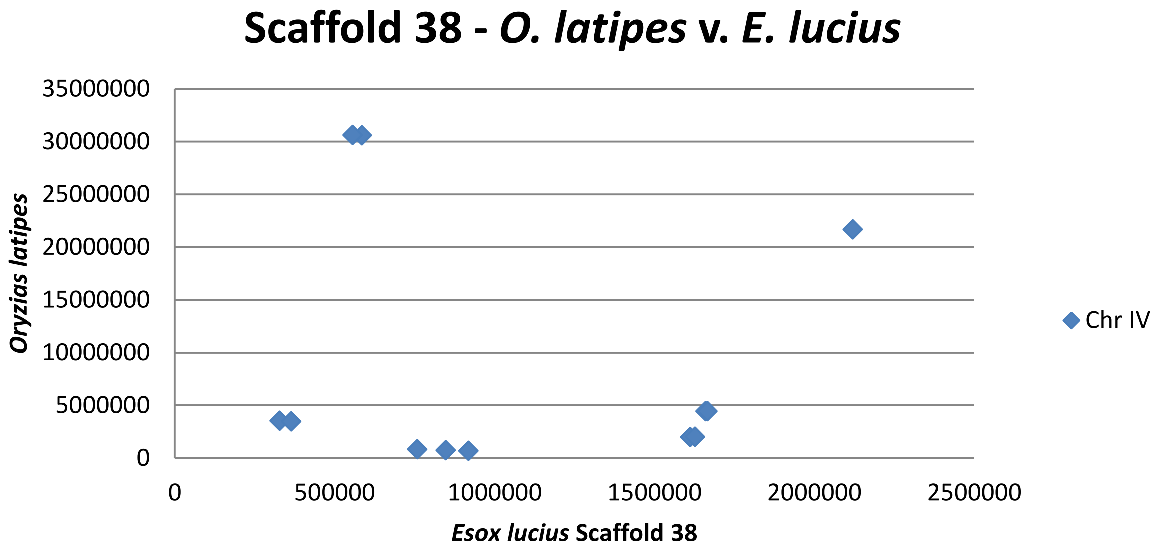

Scaffold 38 - O. latipes v. E. lucius
Oryzias latipes
35000000
30000000
25000000
20000000
15000000
10000000
5000000
0
0
500000
1000000
1500000
2000000
2500000
Esox lucius Scaffold 38
Chr IV

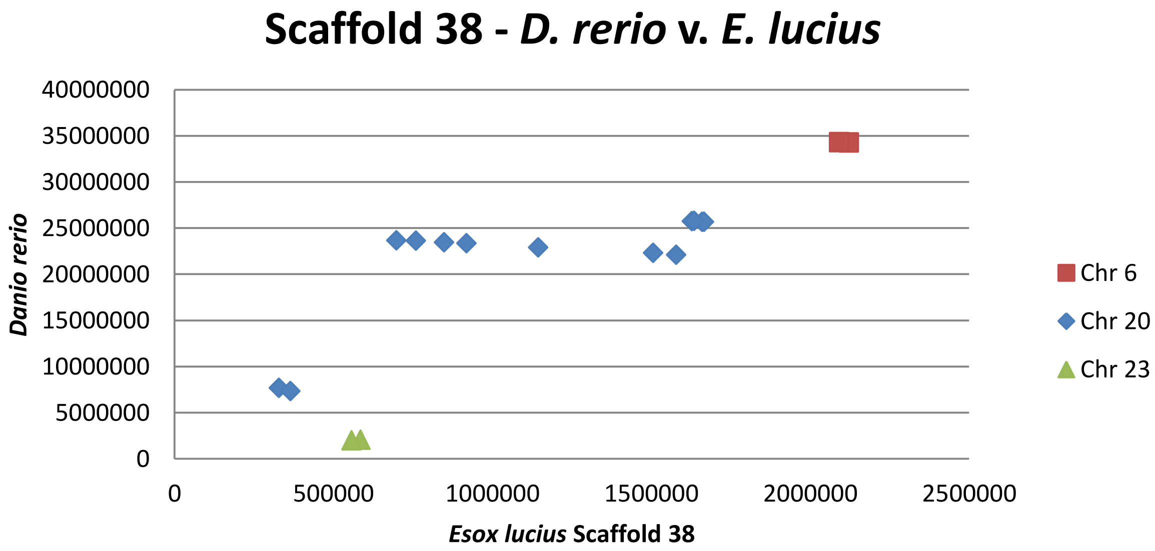

Scaffold 38 - D. rerio v. E. lucius
Danio rerio
40000000
35000000
30000000
25000000
20000000
15000000
10000000
5000000
0
0
500000
1000000
1500000
2000000
2500000
Esox lucius Scaffold 38
Chr 6
Chr 20
Chr 23

Scaffold 39

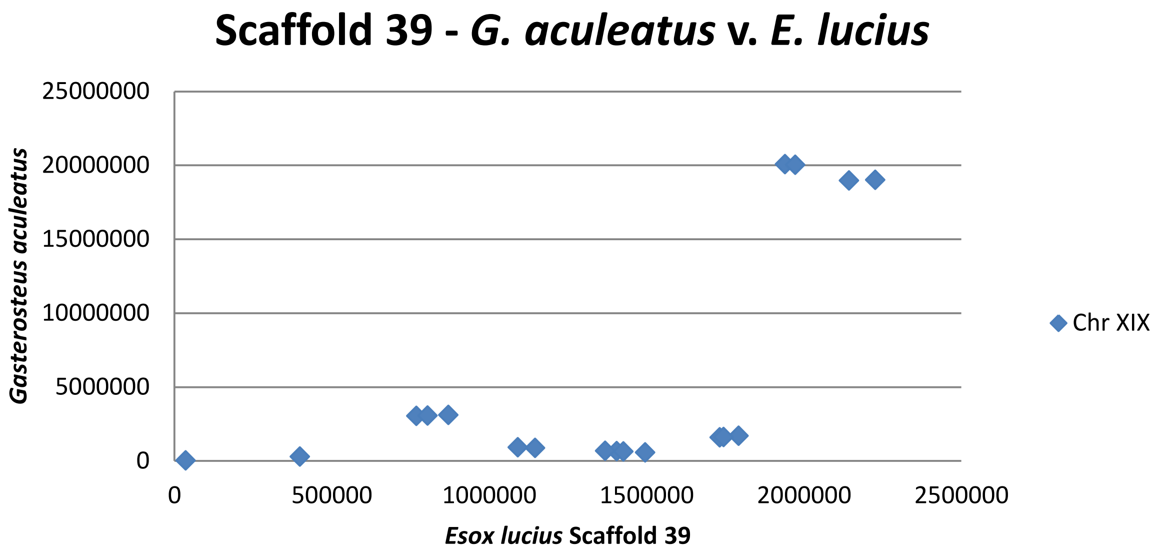
Scaffold 39 - *G. aculeatus* v. *E. lucius*
25000000
20000000
15000000
10000000
5000000
0
Gasterosteus aculeatus
0
500000
1000000
1500000
2000000
2500000
Esox lucius Scaffold 39
Chr XIX

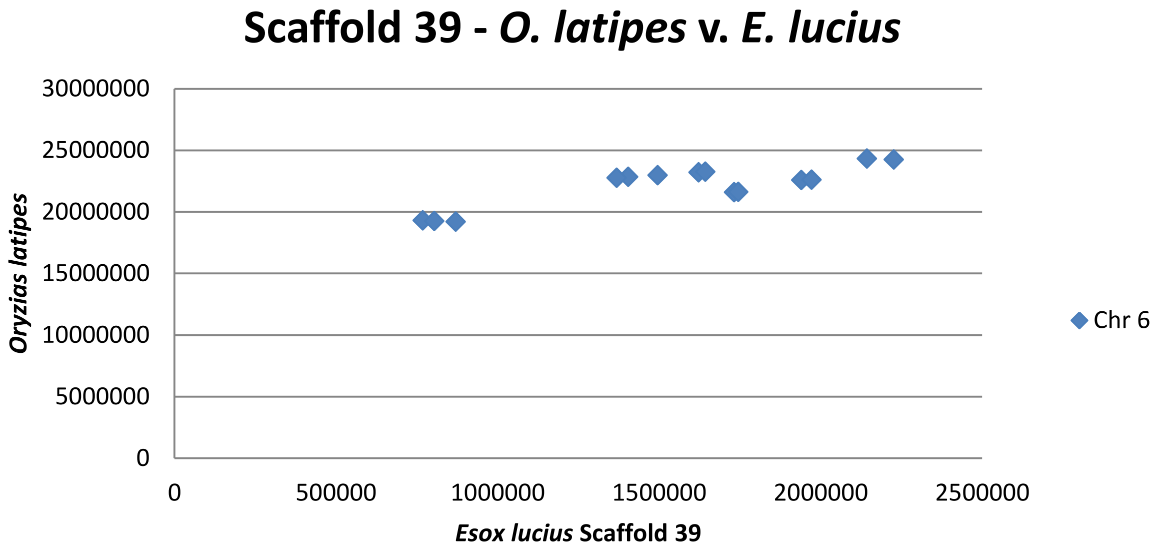
Scaffold 39 - *O. latipes* v. *E. lucius*
30000000
25000000
20000000
15000000
10000000
5000000
0
Oryzias latipes
0
500000
1000000
1500000
2000000
2500000
Esox lucius Scaffold 39
Chr 6

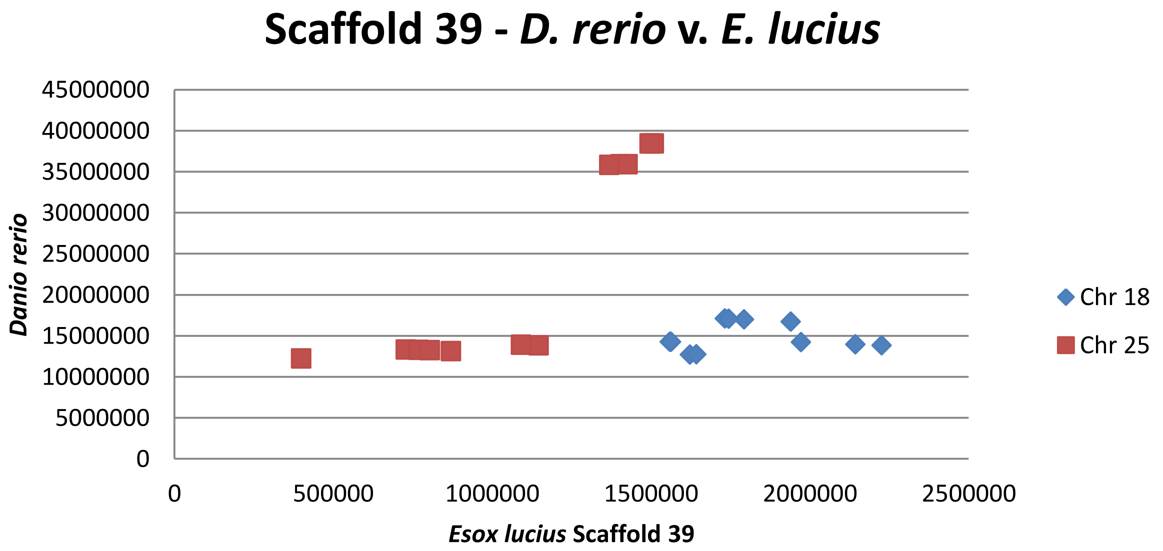
Scaffold 39 - *D. rerio* v. *E. lucius*
45000000
40000000
35000000
30000000
25000000
20000000
15000000
10000000
5000000
0
Danio rerio
0
500000
1000000
1500000
2000000
2500000
Esox lucius Scaffold 39
Chr 18
Chr 25

Scaffold 40

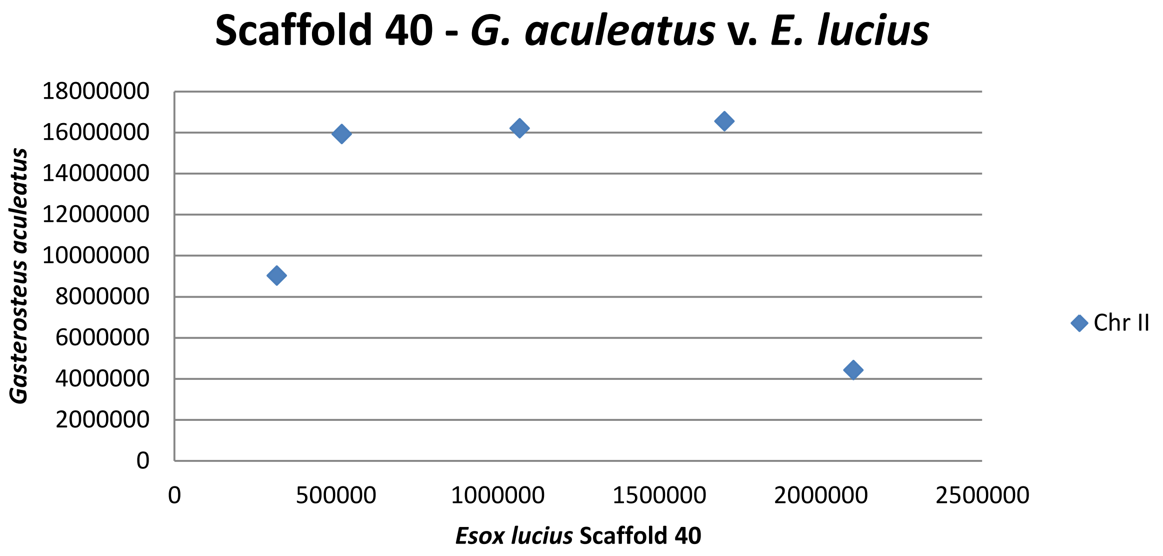
Scaffold 40 - G. aculeatus v. E. lucius
18000000
16000000
14000000
12000000
10000000
8000000
6000000
4000000
2000000
0
Gasterosteus aculeatus
0
500000
1000000
1500000
2000000
2500000
Esox lucius Scaffold 40
Chr II

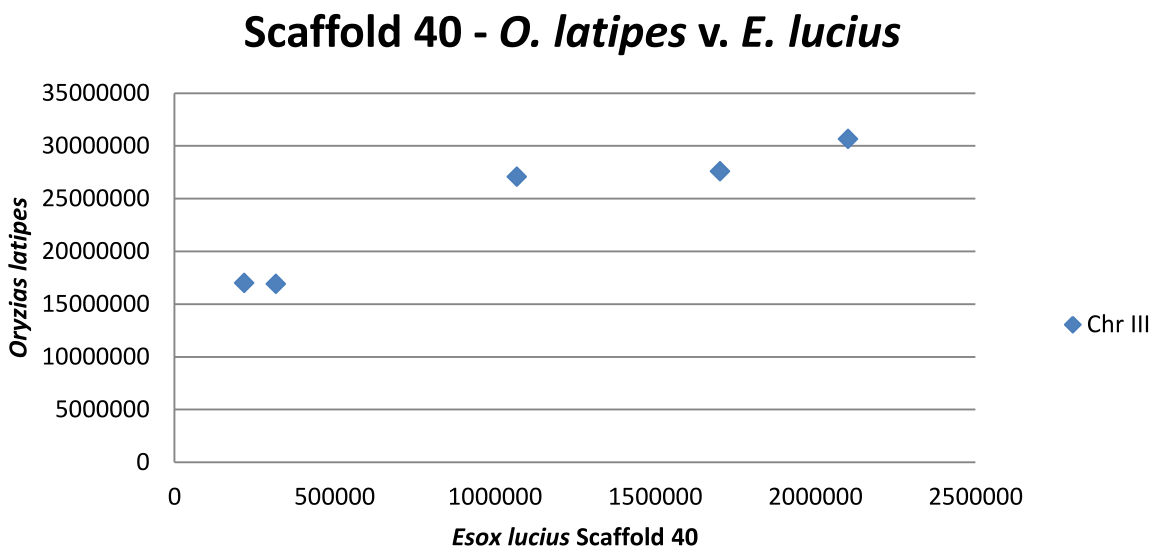
Scaffold 40 - O. latipes v. E. lucius
35000000
30000000
25000000
20000000
15000000
10000000
5000000
0
Oryzias latipes
0
500000
1000000
1500000
2000000
2500000
Esox lucius Scaffold 40
Chr III

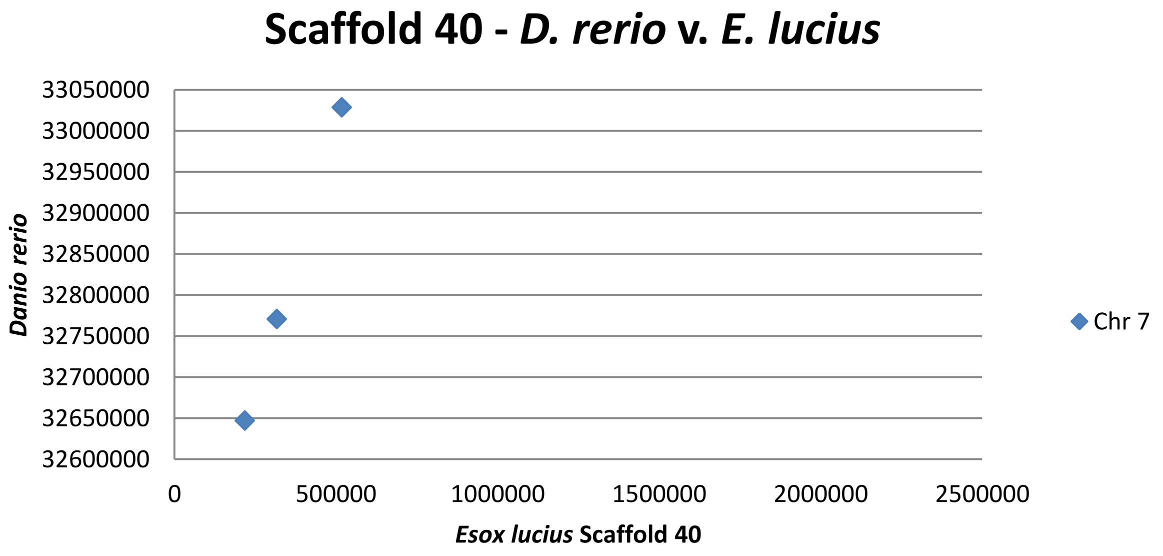
Scaffold 40 - D. rerio v. E. lucius
33050000
33000000
32950000
32900000
32850000
32800000
32750000
32700000
32650000
32600000
Danio rerio
0
500000
1000000
1500000
2000000
2500000
Esox lucius Scaffold 40
Chr 7

Scaffold 41

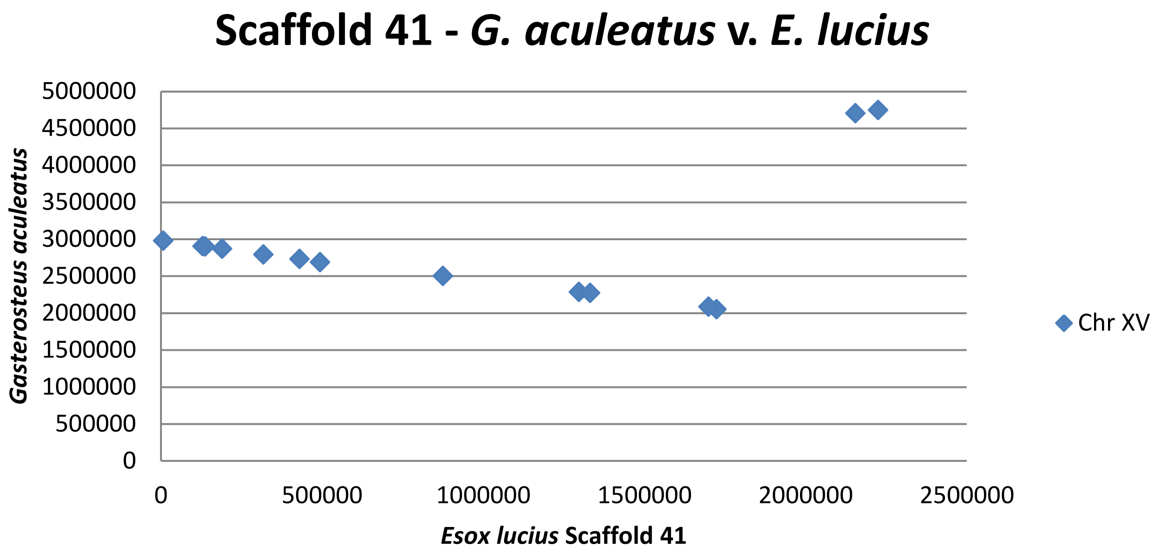

Scaffold 41 - G. aculeatus v. E. lucius
Gasterosteus aculeatus
5000000
4500000
4000000
3500000
3000000
2500000
2000000
1500000
1000000
500000
0
0
500000
1000000
1500000
2000000
2500000
Esox lucius Scaffold 41
Chr XV

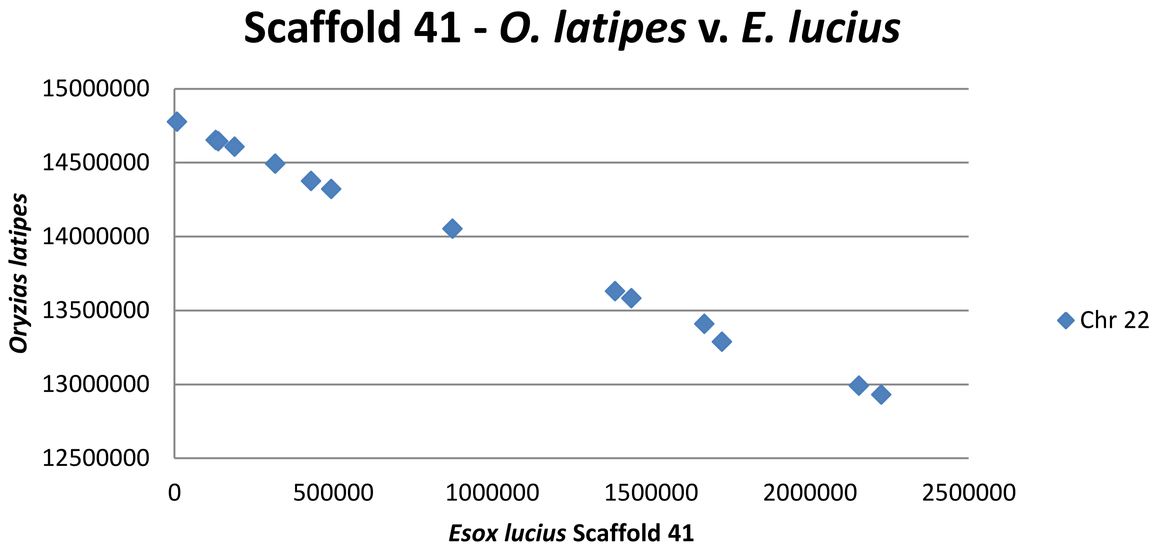

Scaffold 41 - O. latipes v. E. lucius
Oryzias latipes
15000000
14500000
14000000
13500000
13000000
12500000
0
500000
1000000
1500000
2000000
2500000
Esox lucius Scaffold 41
Chr 22

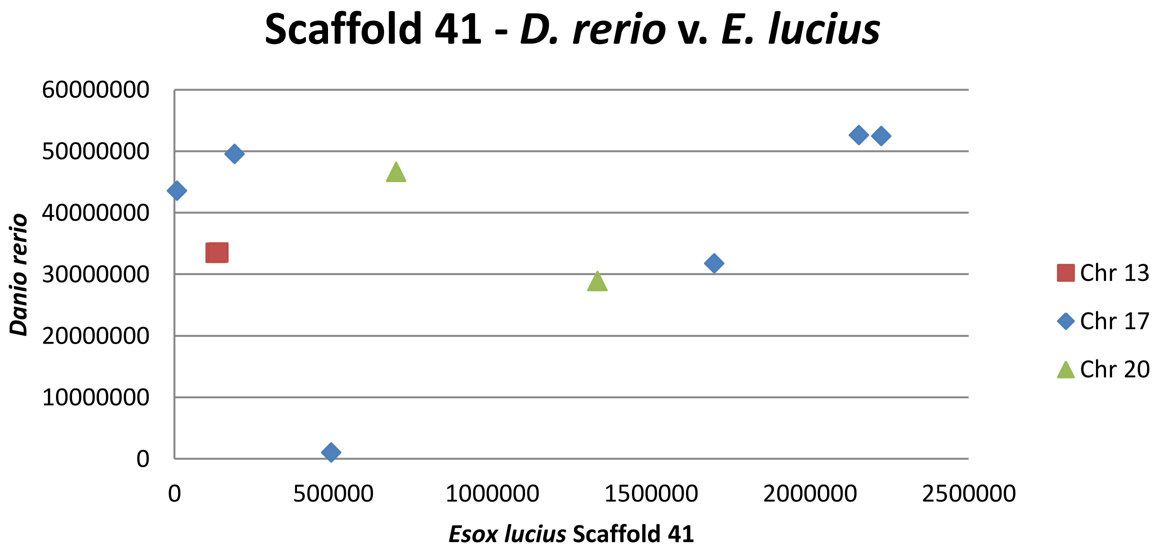

Scaffold 41 - D. rerio v. E. lucius
Danio rerio
60000000
50000000
40000000
30000000
20000000
10000000
0
0
500000
1000000
1500000
2000000
2500000
Esox lucius Scaffold 41
Chr 13
Chr 17
Chr 20

Scaffold 42

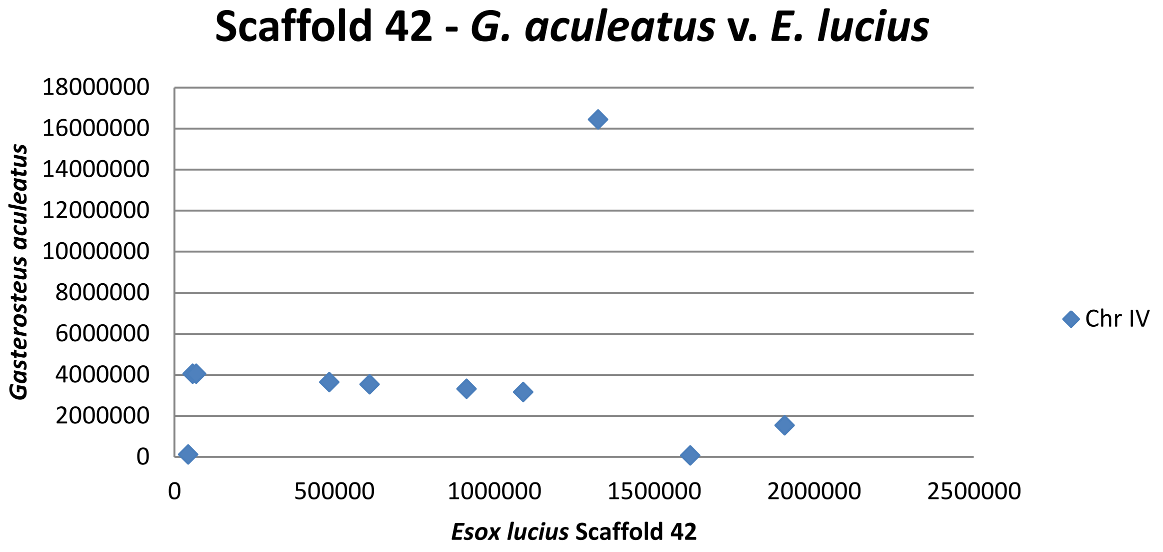
Scaffold 42 - G. aculeatus v. E. lucius
Gasterosteus aculeatus
Esox lucius Scaffold 42
Chr IV
0
2000000
4000000
6000000
8000000
10000000
12000000
14000000
16000000
18000000
500000
1000000
1500000
2000000
2500000

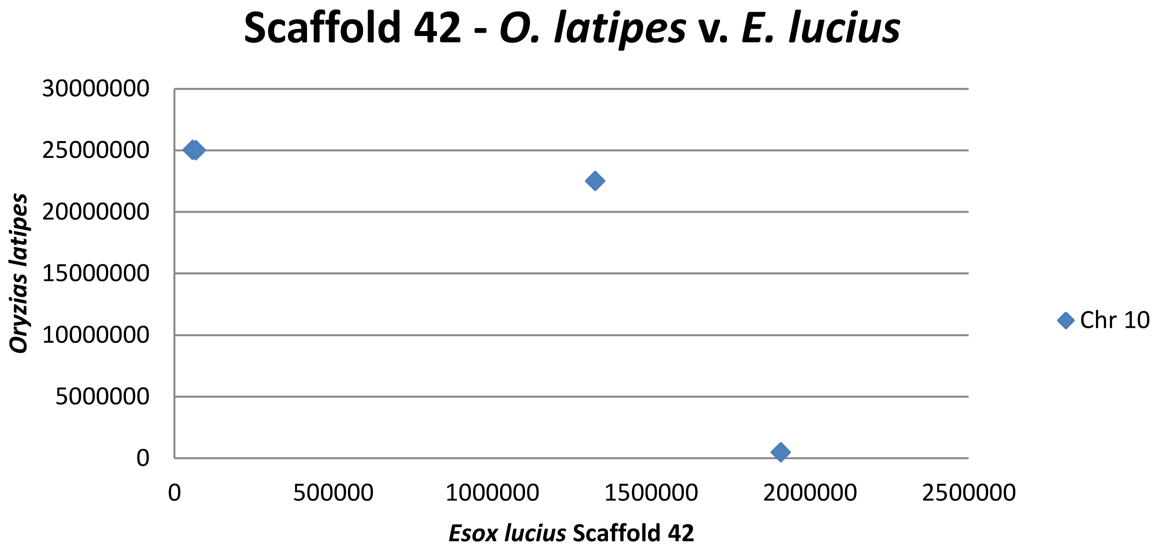
Scaffold 42 - O. latipes v. E. lucius
Oryzias latipes
Esox lucius Scaffold 42
Chr 10
0
5000000
10000000
15000000
20000000
25000000
30000000
500000
1000000
1500000
2000000
2500000

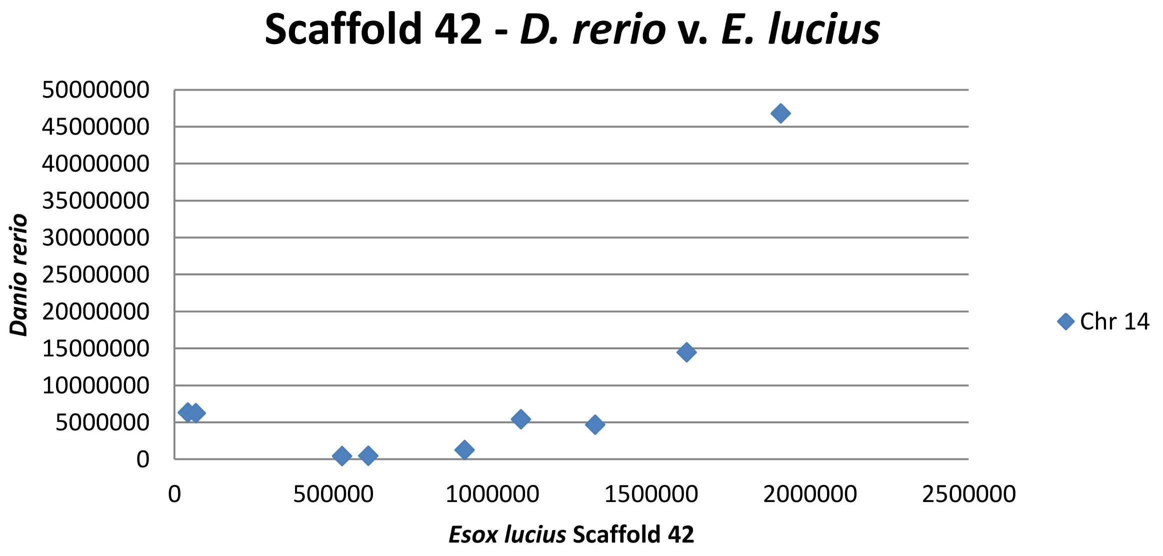
Scaffold 42 - D. rerio v. E. lucius
Danio rerio
Esox lucius Scaffold 42
Chr 14
0
5000000
10000000
15000000
20000000
25000000
30000000
35000000
40000000
45000000
50000000
500000
1000000
1500000
2000000
2500000

Scaffold 43

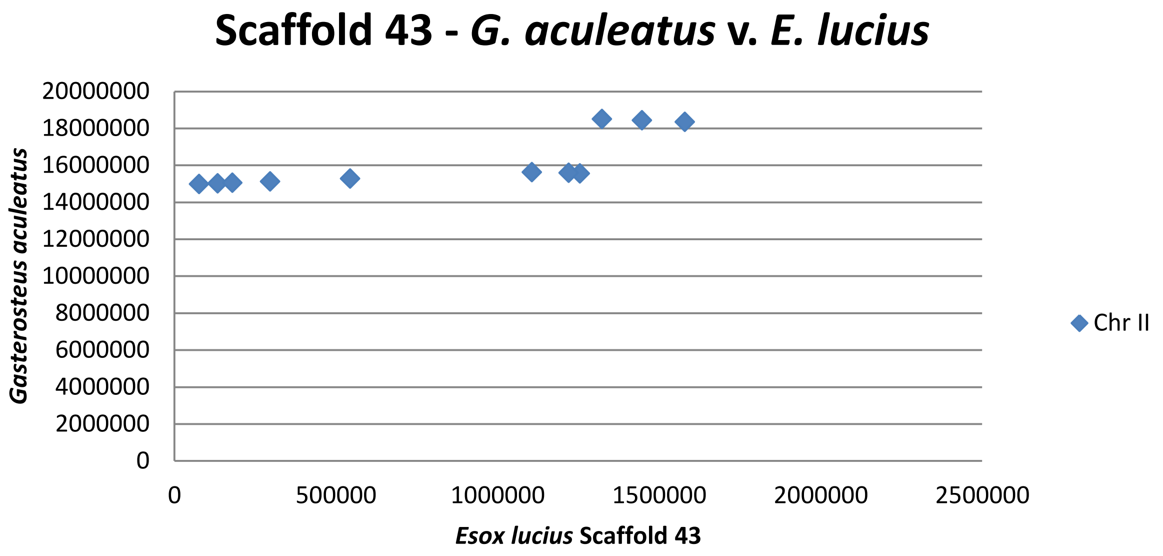

Scaffold 43 - G. aculeatus v. E. lucius
Gasterosteus aculeatus
20000000
18000000
16000000
14000000
12000000
10000000
8000000
6000000
4000000
2000000
0
0
500000
1000000
1500000
2000000
2500000
Esox lucius Scaffold 43
Chr II

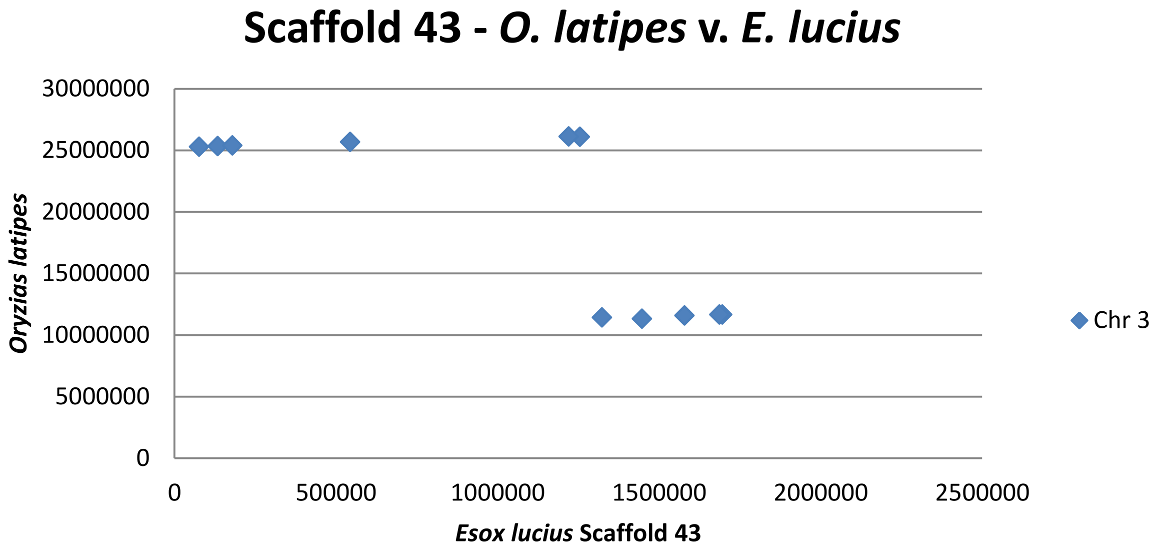

Scaffold 43 - O. latipes v. E. lucius
Oryzias latipes
30000000
25000000
20000000
15000000
10000000
5000000
0
0
500000
1000000
1500000
2000000
2500000
Esox lucius Scaffold 43
Chr 3

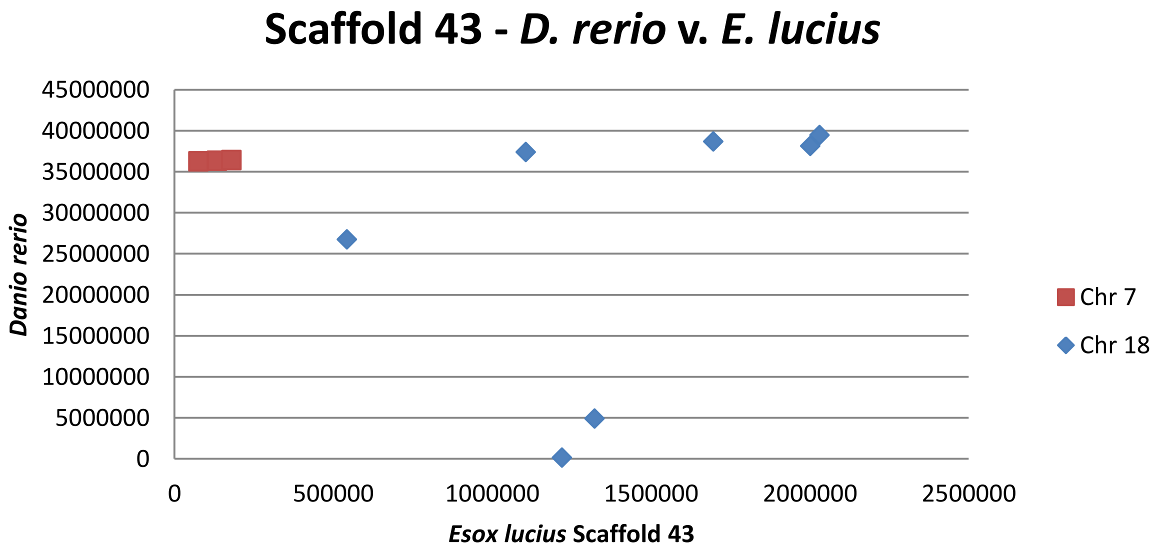

Scaffold 43 - D. rerio v. E. lucius
Danio rerio
45000000
40000000
35000000
30000000
25000000
20000000
15000000
10000000
5000000
0
0
500000
1000000
1500000
2000000
2500000
Esox lucius Scaffold 43
Chr 7
Chr 18

Scaffold 44

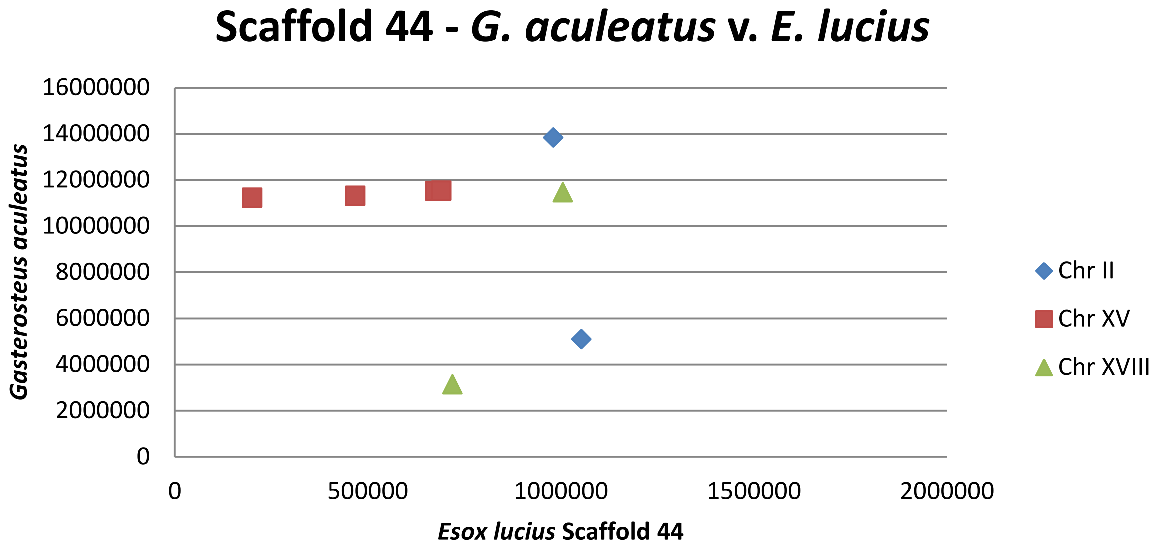
Scaffold 44 - G. aculeatus v. E. lucius
Gasterosteus aculeatus
16000000
14000000
12000000
10000000
8000000
6000000
4000000
2000000
0
0
500000
1000000
1500000
2000000
Esox lucius Scaffold 44
Chr II
Chr XV
Chr XVIII

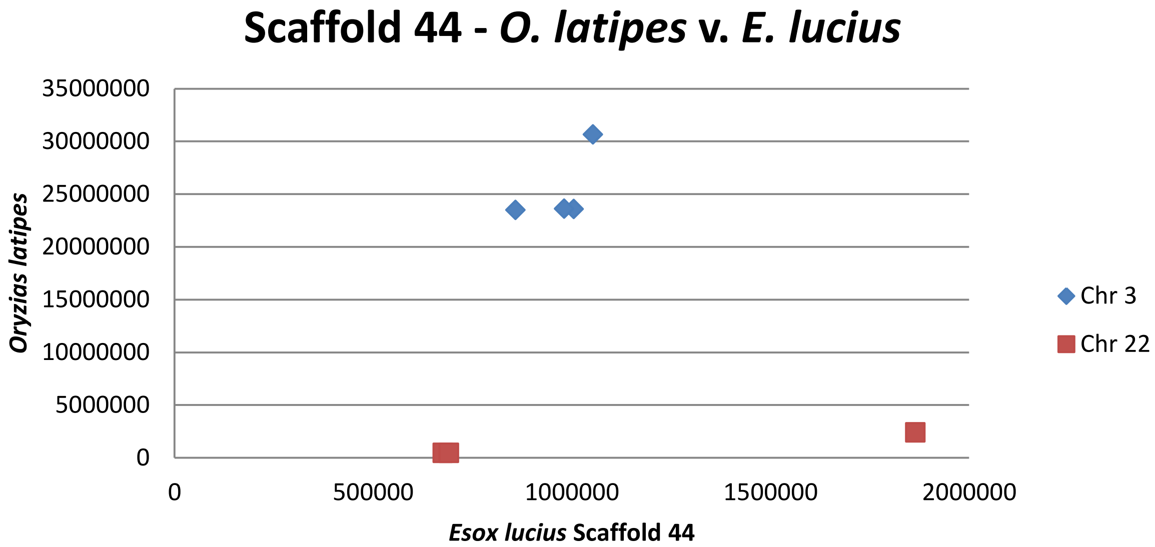
Scaffold 44 - O. latipes v. E. lucius
Oryzias latipes
35000000
30000000
25000000
20000000
15000000
10000000
5000000
0
0
500000
1000000
1500000
2000000
Esox lucius Scaffold 44
Chr 3
Chr 22

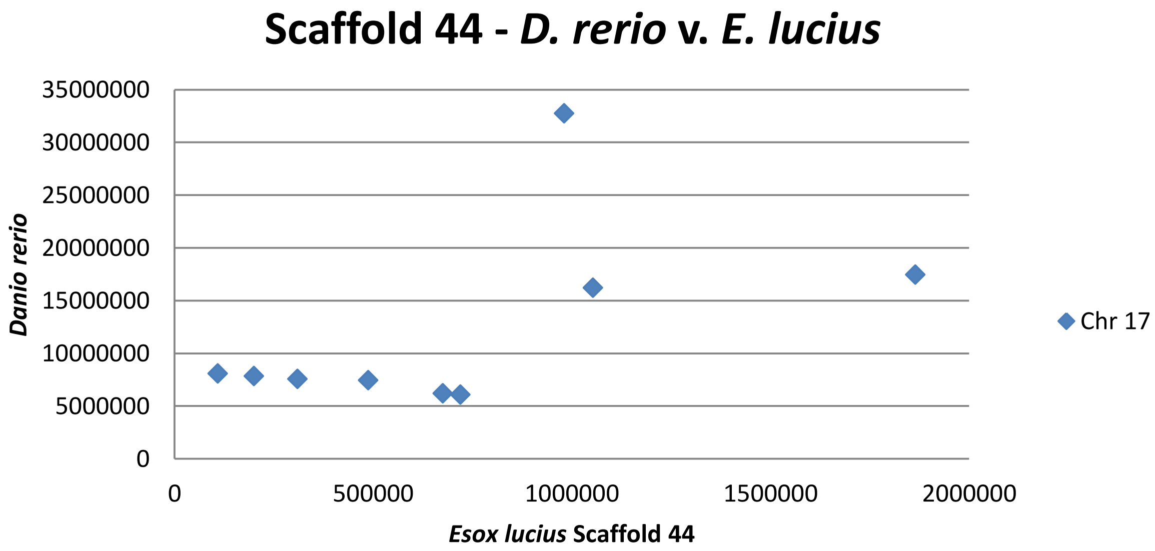
Scaffold 44 - D. rerio v. E. lucius
Danio rerio
35000000
30000000
25000000
20000000
15000000
10000000
5000000
0
0
500000
1000000
1500000
2000000
Esox lucius Scaffold 44
Chr 17

Scaffold 45

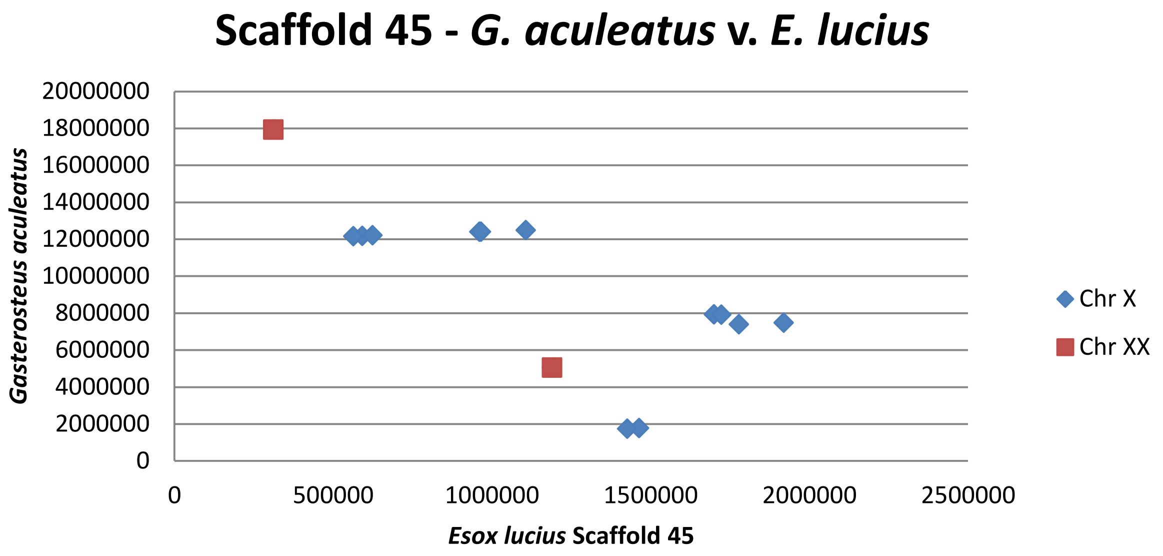

Scaffold 45 - *G. aculeatus* v. *E. lucius*
Gasterosteus aculeatus
20000000
18000000
16000000
14000000
12000000
10000000
8000000
6000000
4000000
2000000
0
0
500000
1000000
1500000
2000000
2500000
Esox lucius Scaffold 45
Chr X
Chr XX

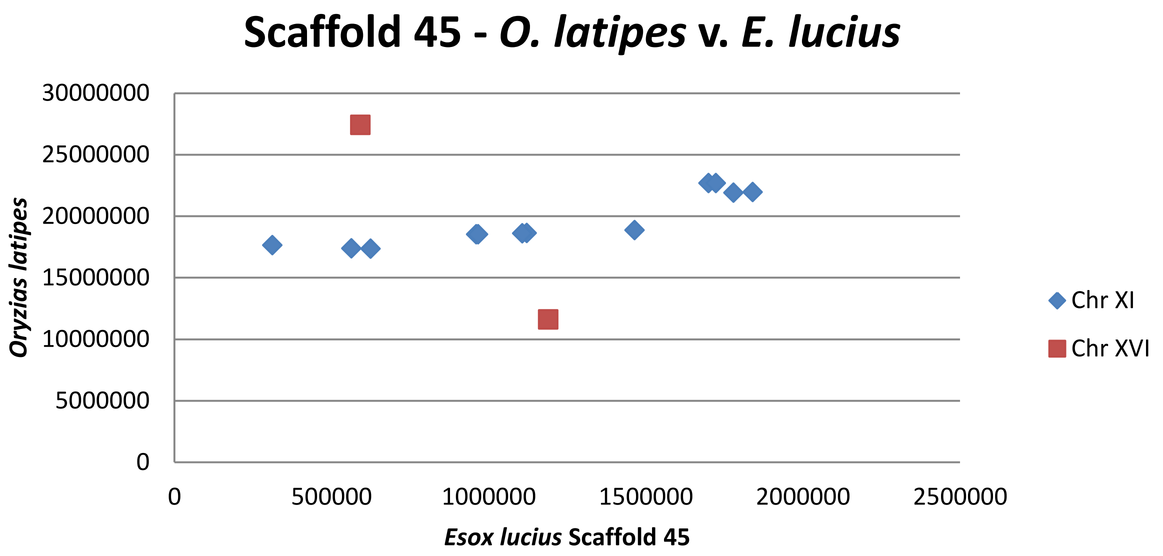

Scaffold 45 - *O. latipes* v. *E. lucius*
Oryzias latipes
30000000
25000000
20000000
15000000
10000000
5000000
0
0
500000
1000000
1500000
2000000
2500000
Esox lucius Scaffold 45
Chr XI
Chr XVI

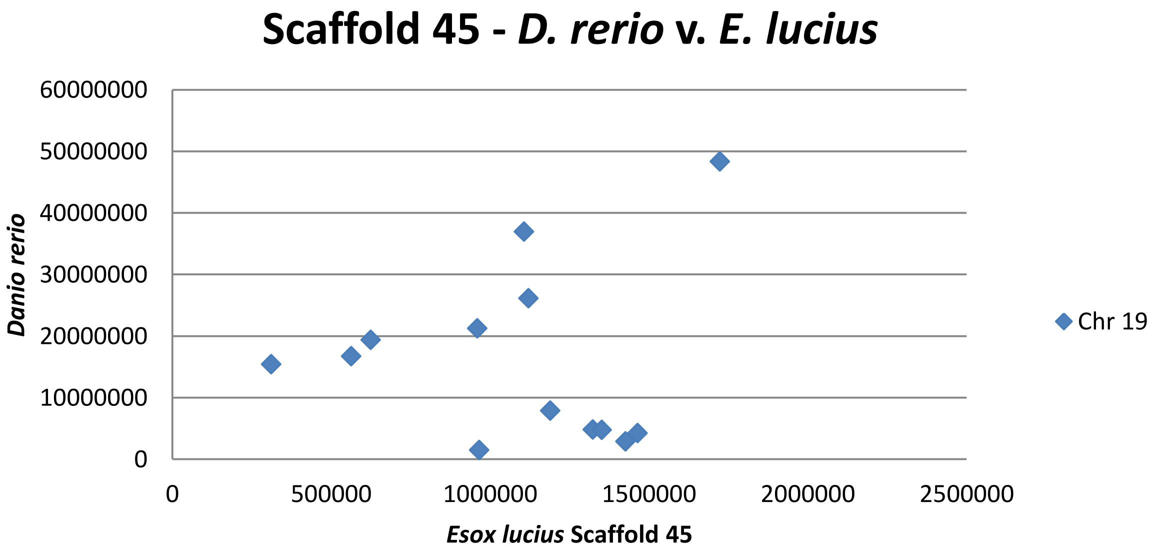

Scaffold 45 - *D. rerio* v. *E. lucius*
Danio rerio
60000000
50000000
40000000
30000000
20000000
10000000
0
0
500000
1000000
1500000
2000000
2500000
Esox lucius Scaffold 45
Chr 19

Scaffold 46

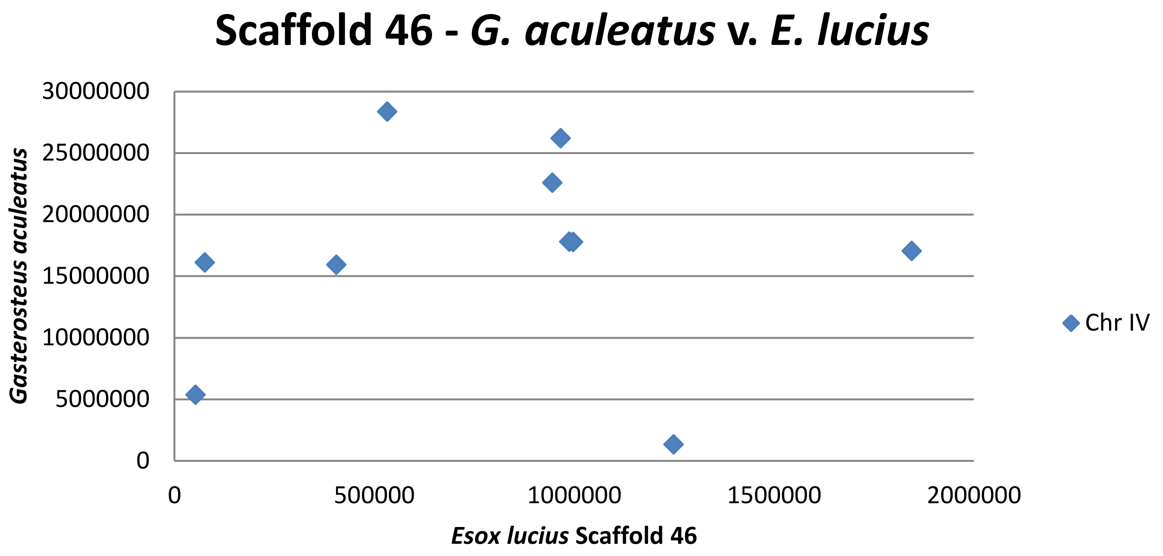

Scaffold 46 - G. aculeatus v. E. lucius
Gasterosteus aculeatus
30000000
25000000
20000000
15000000
10000000
5000000
0
0
500000
1000000
1500000
2000000
Esox lucius Scaffold 46
Chr IV

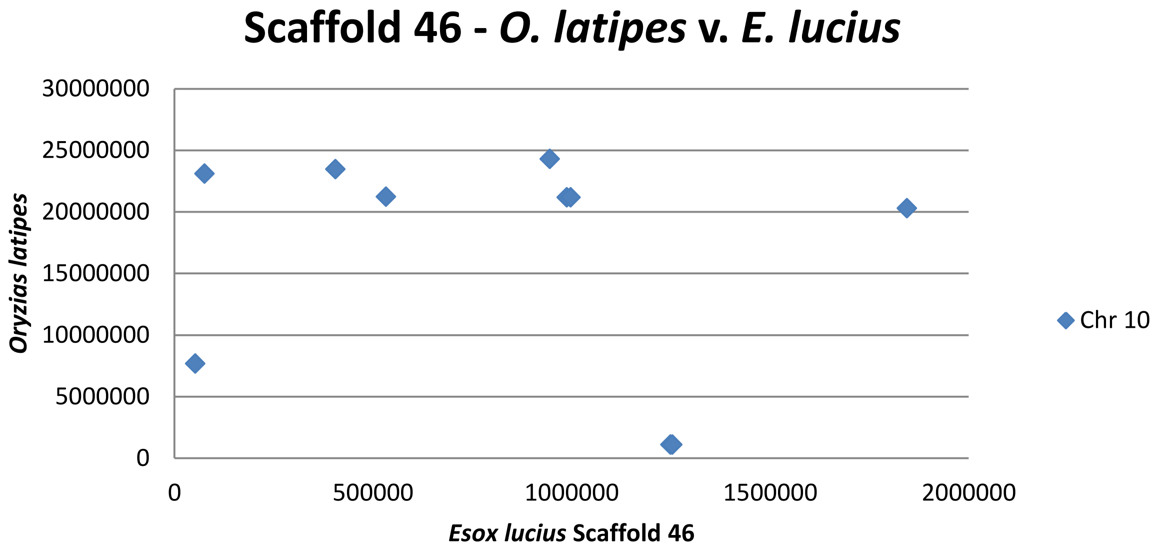

Scaffold 46 - O. latipes v. E. lucius
Oryzias latipes
30000000
25000000
20000000
15000000
10000000
5000000
0
0
500000
1000000
1500000
2000000
Esox lucius Scaffold 46
Chr 10

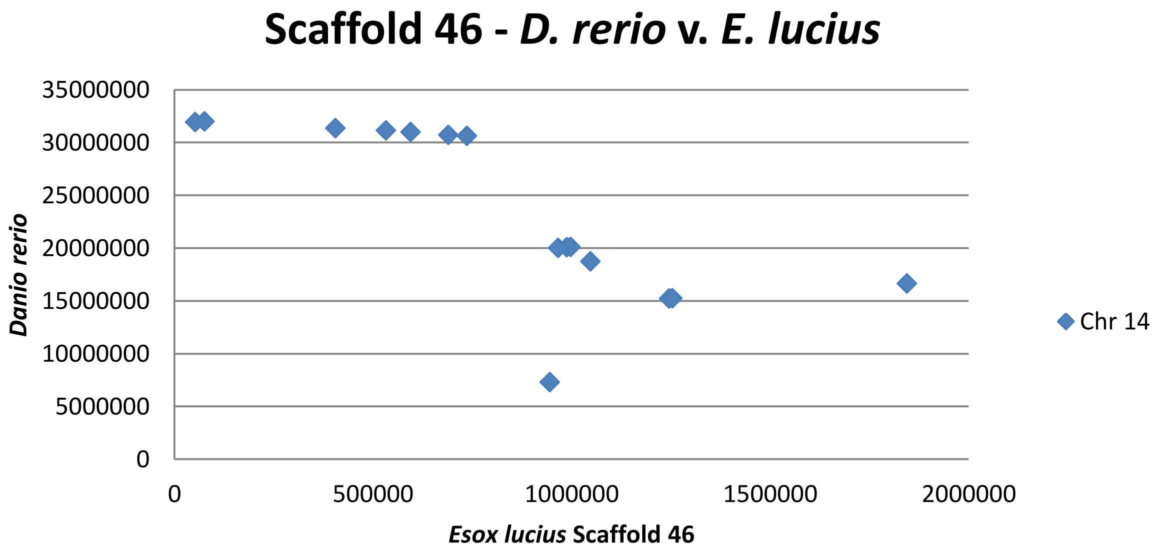

Scaffold 46 - D. rerio v. E. lucius
Danio rerio
35000000
30000000
25000000
20000000
15000000
10000000
5000000
0
0
500000
1000000
1500000
2000000
Esox lucius Scaffold 46
Chr 14

Scaffold 47

Scaffold 47 - *G. aculeatus* v. *E. lucius*

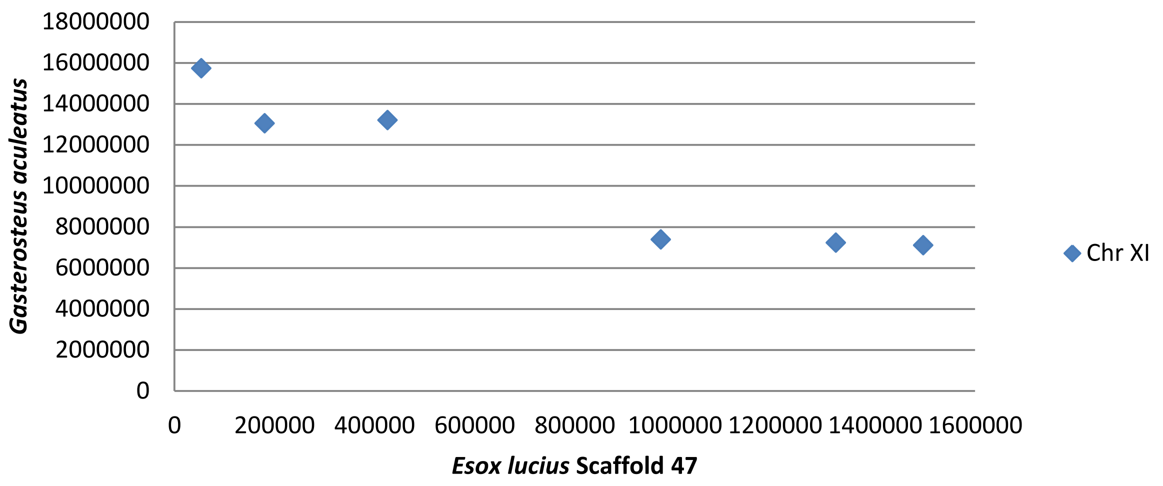

Scaffold 47 - *O. latipes* v. *E. lucius*

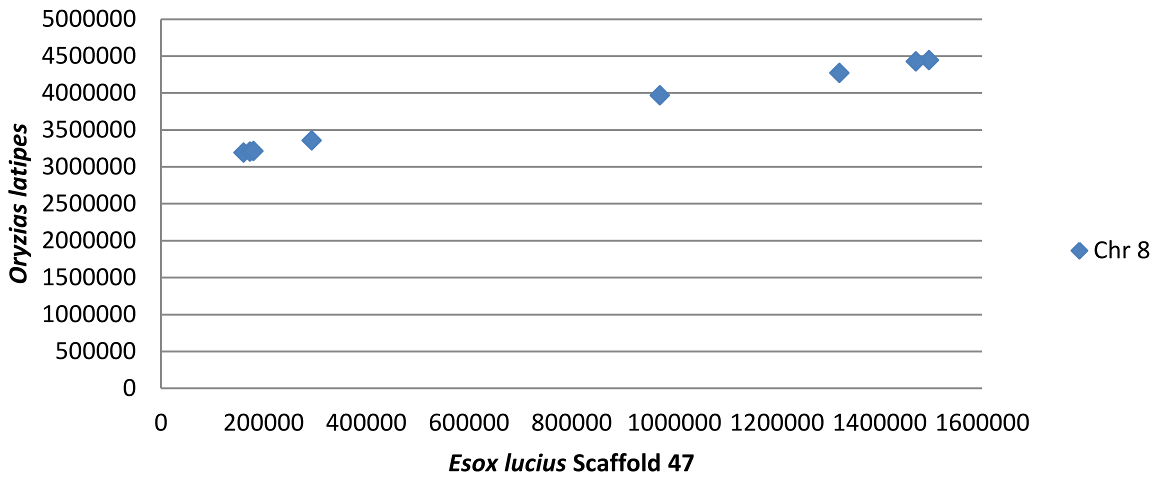

Scaffold 47 - *D. rerio* v. *E. lucius*

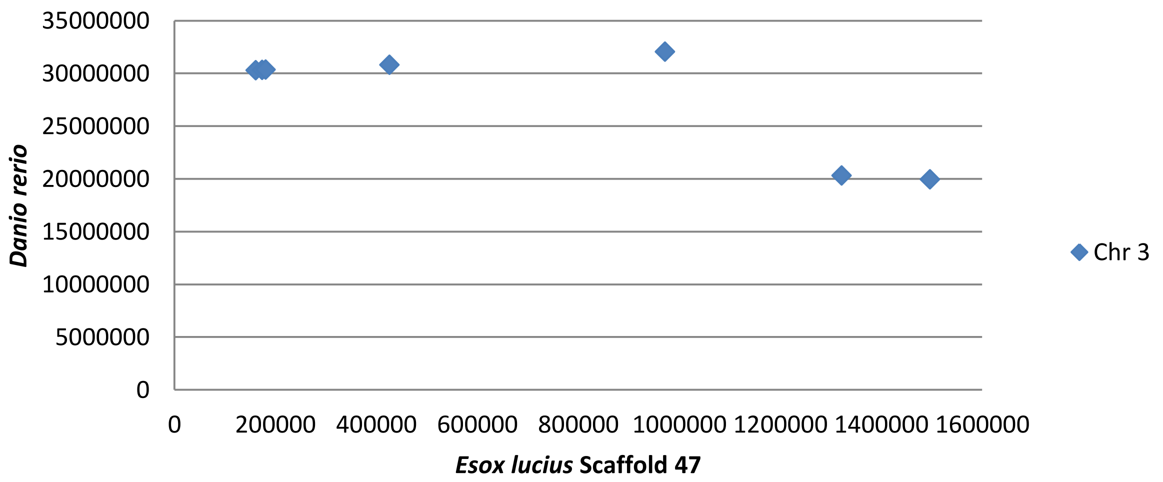

Scaffold 48

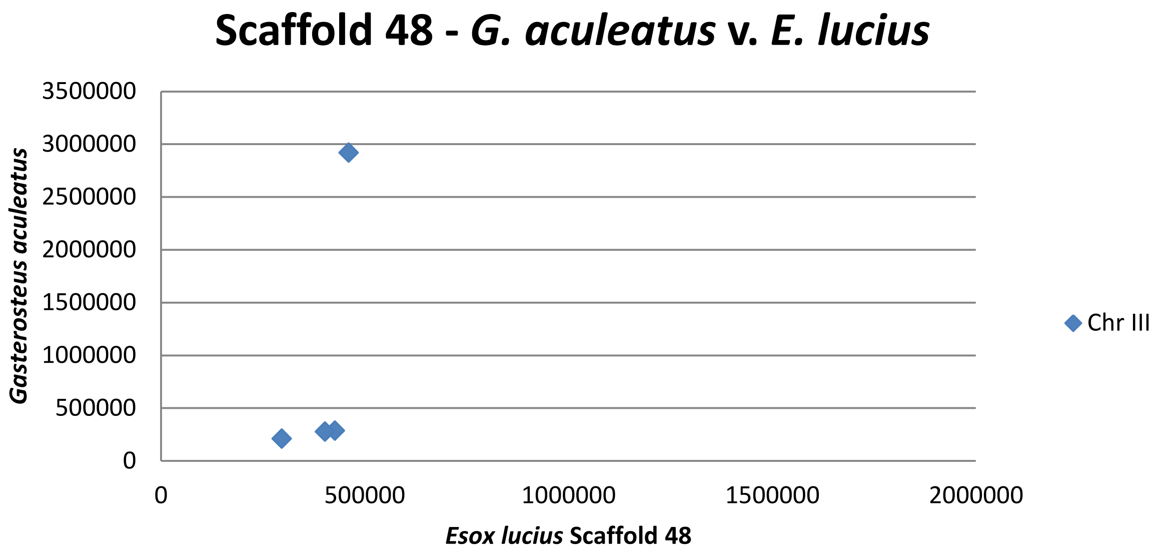
Scaffold 48 - *G. aculeatus* v. *E. lucius*
3500000
3000000
2500000
2000000
1500000
1000000
500000
0
0
500000
1000000
1500000
2000000
Gasterosteus aculeatus
Esox lucius Scaffold 48
Chr III

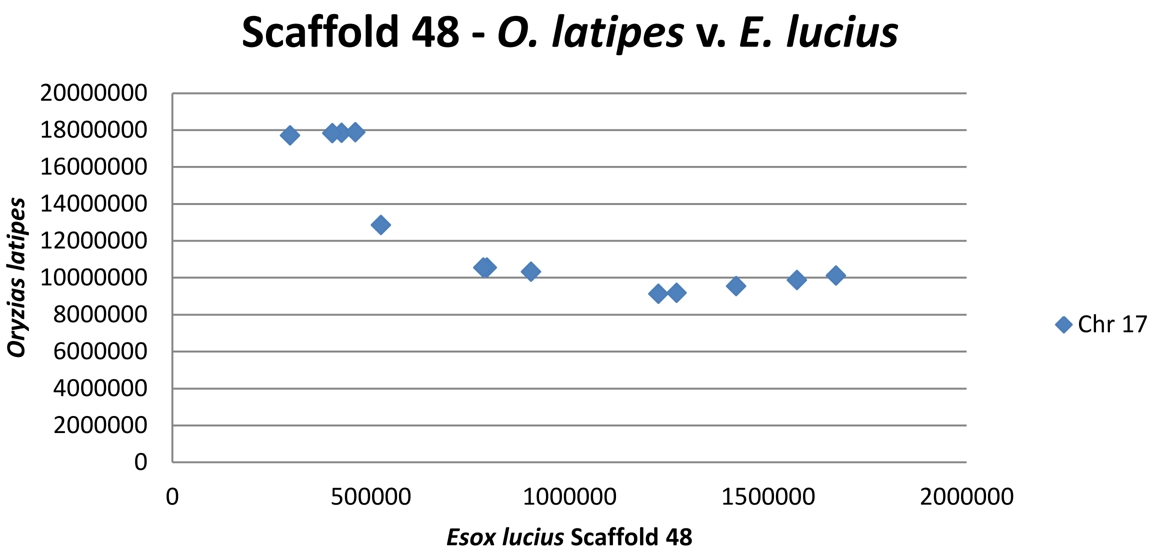
Scaffold 48 - *O. latipes* v. *E. lucius*
20000000
18000000
16000000
14000000
12000000
10000000
8000000
6000000
4000000
2000000
0
0
500000
1000000
1500000
2000000
Oryzias latipes
Esox lucius Scaffold 48
Chr 17

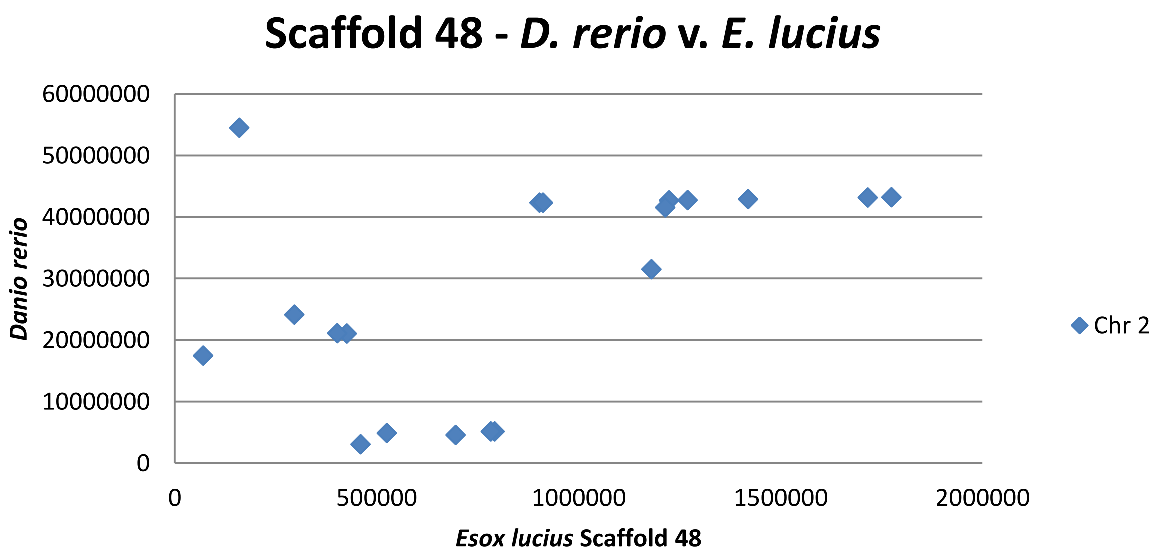
Scaffold 48 - *D. rerio* v. *E. lucius*
60000000
50000000
40000000
30000000
20000000
10000000
0
0
500000
1000000
1500000
2000000
Danio rerio
Esox lucius Scaffold 48
Chr 2

Scaffold 49

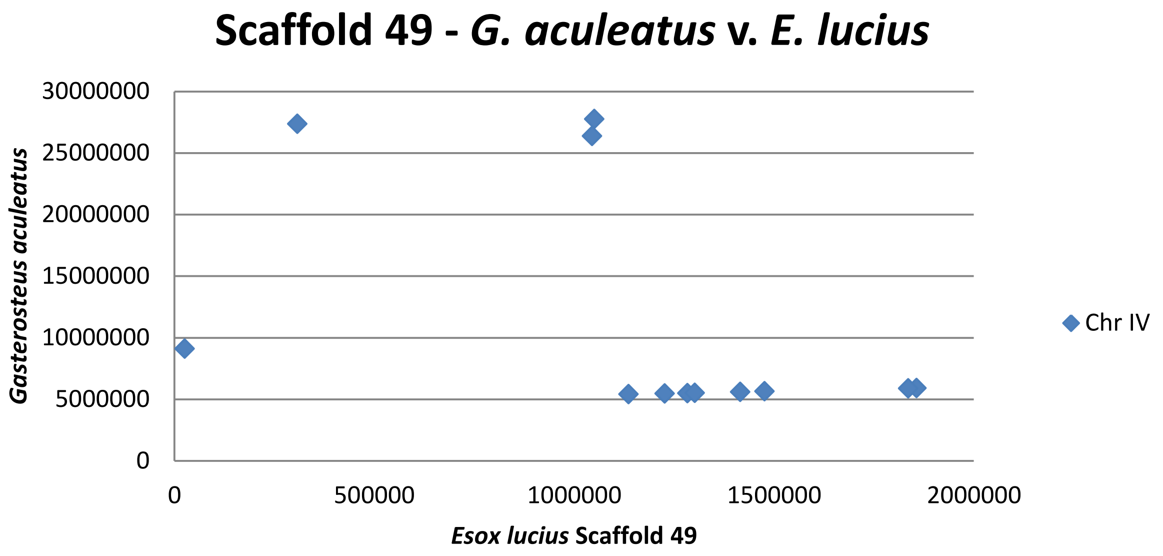

Scaffold 49 - *G. aculeatus* v. *E. lucius*
30000000
25000000
20000000
15000000
10000000
5000000
0
Gasterosteus aculeatus
0
500000
1000000
1500000
2000000
Esox lucius Scaffold 49
Chr IV

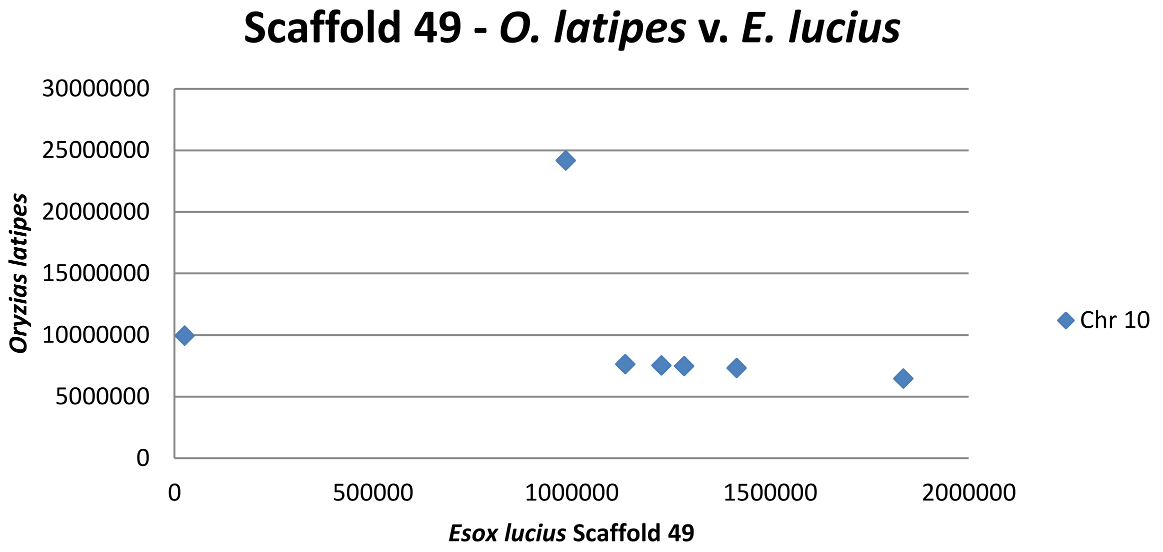

Scaffold 49 - *O. latipes* v. *E. lucius*
30000000
25000000
20000000
15000000
10000000
5000000
0
Oryzias latipes
0
500000
1000000
1500000
2000000
Esox lucius Scaffold 49
Chr 10

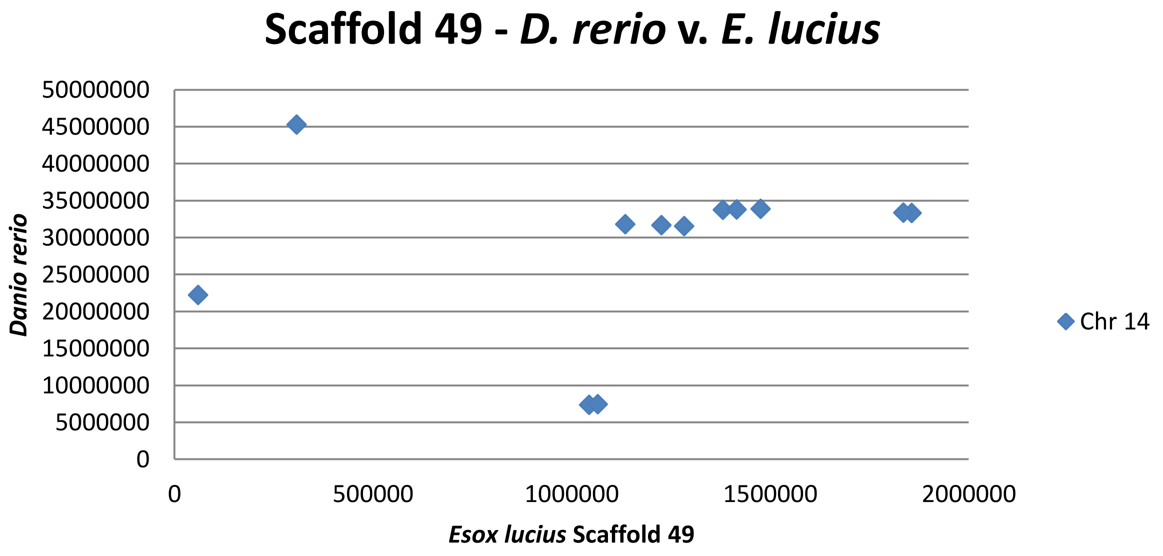

Scaffold 49 - *D. rerio* v. *E. lucius*
50000000
45000000
40000000
35000000
30000000
25000000
20000000
15000000
10000000
5000000
0
Danio rerio
0
500000
1000000
1500000
2000000
Esox lucius Scaffold 49
Chr 14

Scaffold 50

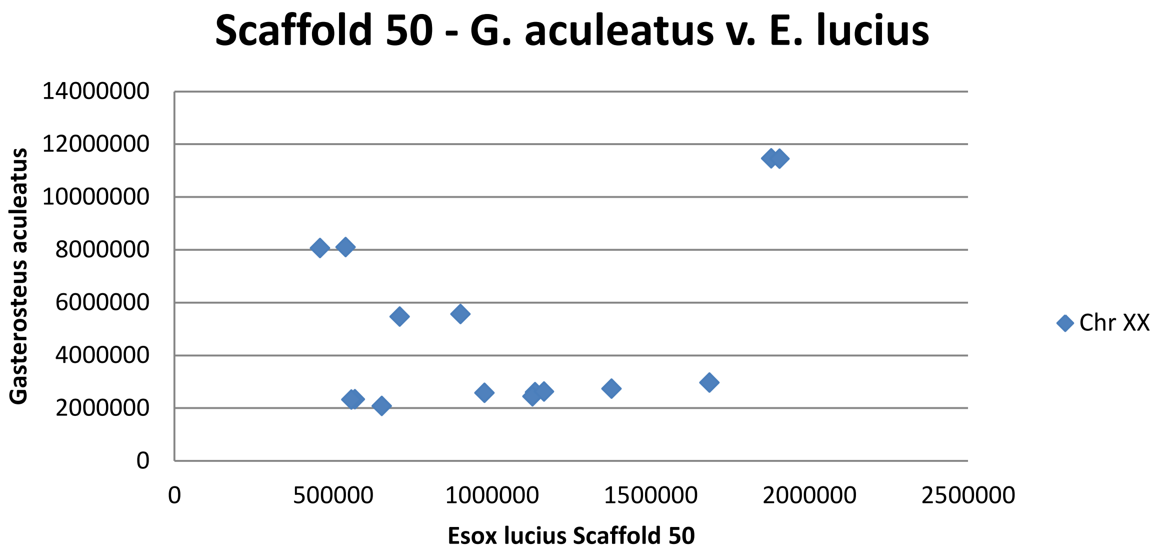
Scaffold 50 - G. aculeatus v. E. lucius
Gasterosteus aculeatus
14000000
12000000
10000000
8000000
6000000
4000000
2000000
0
0
500000
1000000
1500000
2000000
2500000
Esox lucius Scaffold 50
Chr XX

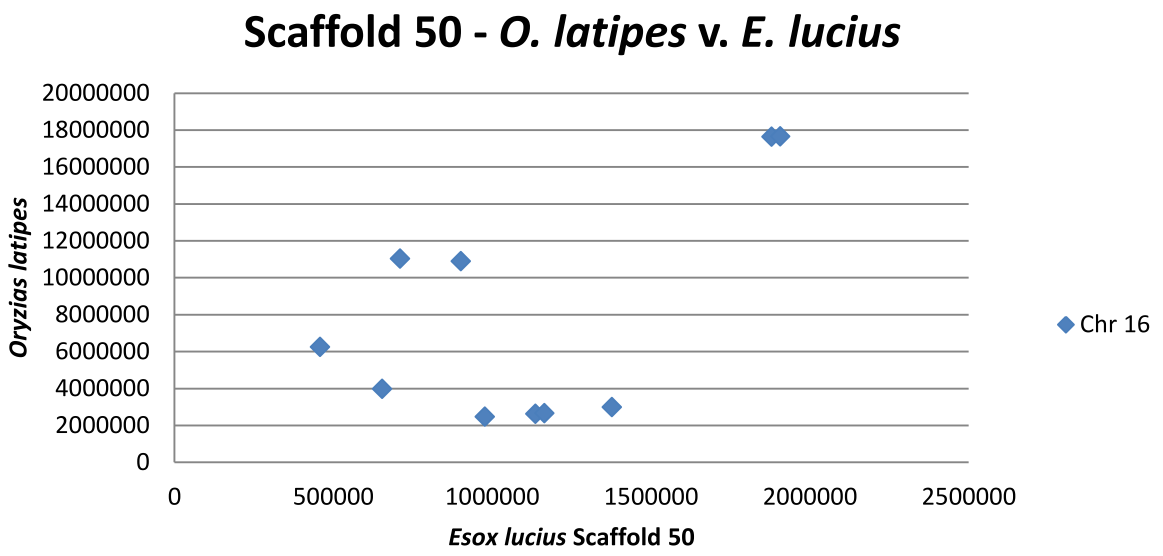
Scaffold 50 - O. latipes v. E. lucius
Oryzias latipes
20000000
18000000
16000000
14000000
12000000
10000000
8000000
6000000
4000000
2000000
0
0
500000
1000000
1500000
2000000
2500000
Esox lucius Scaffold 50
Chr 16

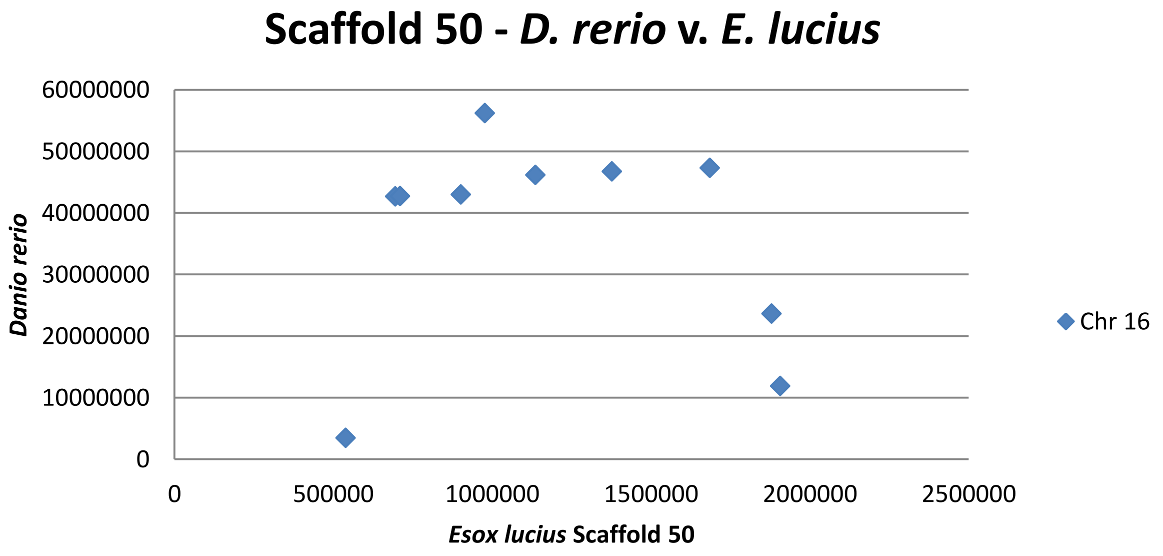
Scaffold 50 - D. rerio v. E. lucius
Danio rerio
60000000
50000000
40000000
30000000
20000000
10000000
0
0
500000
1000000
1500000
2000000
2500000
Esox lucius Scaffold 50
Chr 16
